# Hydrogenolysis triggered formation of zero-valent metals and metal selenides at room temperature from divalent metal aminoxide complexes facilitated by metal-ligand cooperativity

**DOI:** 10.1039/d6sc07403k

**Published:** 2026-10-27

**Authors:** Lina M. Zarnitsa, Oliver E. Garcia, Martin-Louis Y. Riu, Allison Lorber, Miguel Medina Lopez, Anneke Talke, Tyler A. Kerr, Matthew Nava

**Affiliations:** a Department of Chemistry and Biochemistry, University of California Los Angeles California 90095-1569 USA mjnava@ucla.edu

## Abstract

The synergistic interaction between a metal center and ligand to activate a substrate, known as metal-ligand cooperativity (MLC), has served as an effective strategy for small molecule activation and catalysis, but MLC has seen limited application in the synthesis of inorganic materials, in part, because of an incomplete understanding of molecular parameters required to facilitate such processes. Herein, we demonstrate the synthesis of zero-valent metals and metal selenides facilitated by MLC. Divalent metal aminoxide complexes (metal = Mg, Ca, Sr, Zn, Cd, Sn, and Pb; aminoxide = 2,2,6,6-tetramethylpiperidinyl-1-oxide (TEMPO^−^)) were systematically synthesized and structurally characterized. Critically, Gutmann-Beckett analysis of the compounds showed that hydrogenolysis reactivity trended with Lewis acidity of the metal center, and gas-phase proton affinity calculations showed no correlation between metal ion identity and nitrogen basicity. When treated with H_2_, complexes [Cd(TEMPO)_2_]_2_·2THF ([Cd]) and [Pb(TEMPO)_2_]_2_ ([Pb]) were reduced to elemental Cd^0^ and Pb^0^, respectively, as assessed by powder X-ray diffraction. Furthermore, treatment of complexes [Cd] or [Pb] with H_2_ in the presence of Ph_3_PSe led to the formation of CdSe and PbSe, respectively, along with Ph_3_P and 2,2,6,6-tetramethylpiperidin-1-ol (TEMPOH). Our studies suggest that an MLC-mediated hydrogenolysis reactivity pathway can be leveraged to furnish putative transient metal-hydride species, and such species could serve as zero-valent metal synthons for binary material synthesis.

## Abstract 2

Metal–ligand cooperativity (MLC) enables small-molecule activation and catalysis but remains underexplored in inorganic materials synthesis. Here, we explore molecular parameters governing materials syntheses from MLC mediated processes.

## Graphical abstract

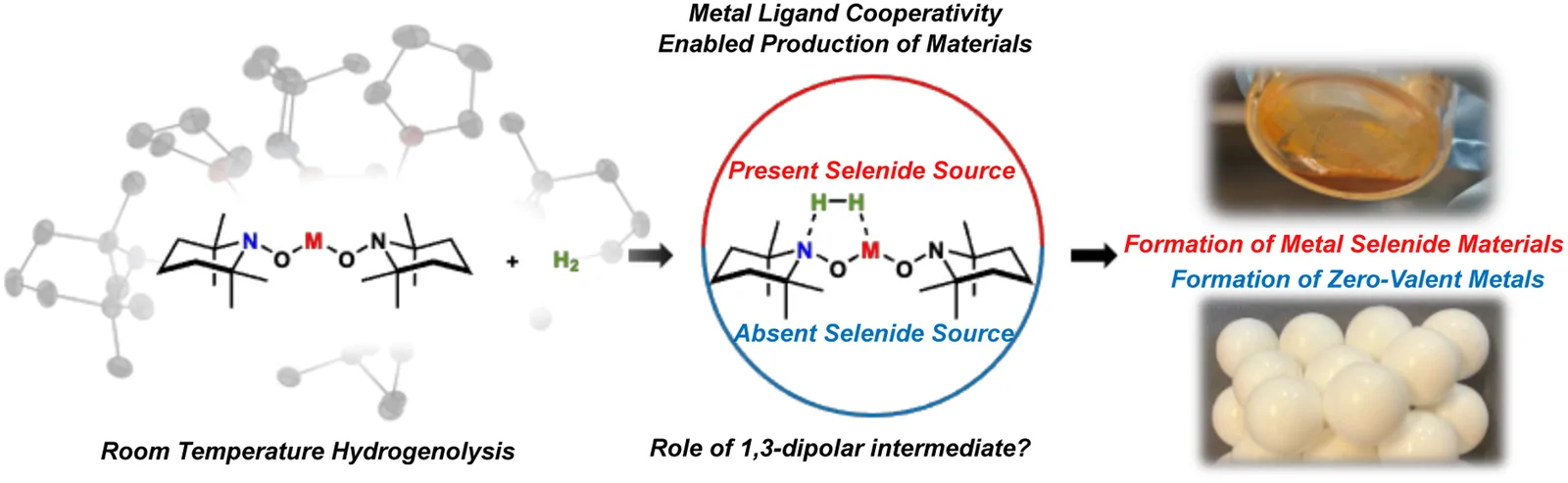

## Introduction

Methods to synthesize materials under mild conditions have garnered interest in recent years to mitigate the potential degradation pathways commonly encountered in high-temperature synthetic procedures or to access metastable phases.^1–5^ Several strategies have been developed to access binary materials that do not proceed through a redox mechanism, including metathesis and elimination reactions, some of which can be conducted at or near room temperature to afford materials.^6,7^ Complementary to these non-redox approaches, the reduction of metal ions to their zero-valent state offers an alternative strategy to access binary materials *via* subsequent redox reaction with a chalcogenide source. Reducing metals to their zero-valent state can be achieved by hydrogenolysis, as exemplified by the treatment of metal salts or oxides with H_2_, but this method must typically be conducted at temperatures greater than 500 °C (Scheme 1(a)).^8–10^ To enable low-temperature materials syntheses, chemists have tailored reaction conditions or engineered precursors that react in a controlled manner to furnish a zero-valent metal or its synthetic equivalent to form a binary material through a subsequent redox reaction.^11–19^ Processes that enable the generation and isolation of reactive intermediates, such as metal hydrides, have additionally facilitated the formation of nanomaterials.^20^ Building on these approaches, the design of precursors that selectively transform into reactive intermediates, particularly those which produce transient subvalent metal species, under mild conditions may provide a strategic route to the controlled synthesis of binary materials. In this regard, metal-ligand cooperativity (MLC) provides a useful framework for precursor design, wherein the ligand and metal engage in bond formation and cleavage, enabling the controlled generation of reactive intermediates.^5,21–23^ This versatile synthetic strategy has commonly been employed for processes such as small molecule activation and catalysis,^22,24,25^ yet has seen limited use in materials synthesis.^2^

**Scheme 1:**
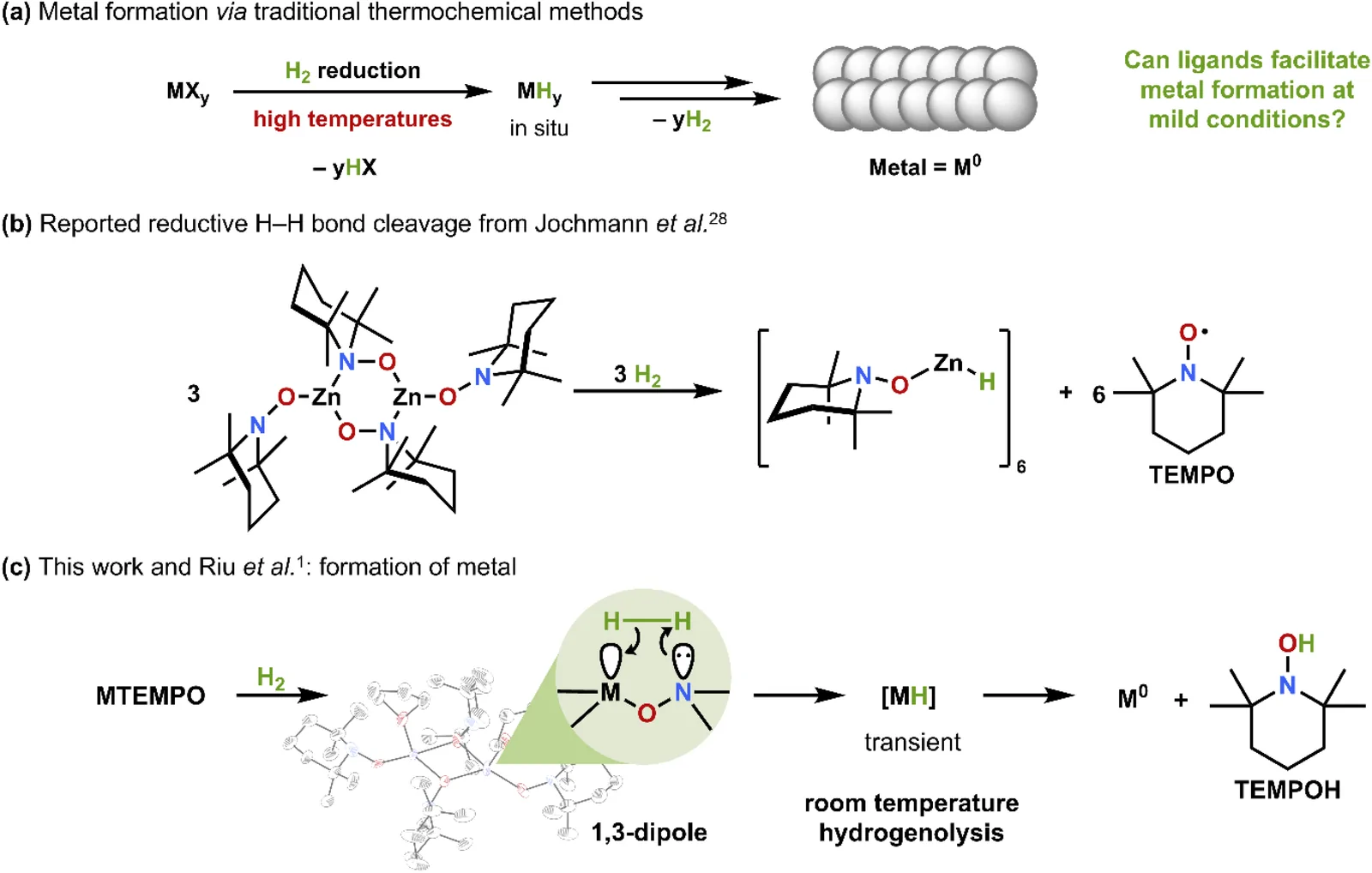
(a) Canonical treatment of metal halides with hydrogen gas to form metals involving high temperatures. (b) Reductive H–H bond cleavage from Jochmann *et al.*^28^ (c) This work and Riu *et al.*:^1^ formation of metals by hydrogenolysis of metal aminoxide complexes at room temperature.

Group 13 aminoxide complexes, Ga(TEMPO)_3_·THF and In(TEMPO)_3_·Py (TEMPO = 2,2,6,6-tetramethylpiperidinyl-1-oxide, THF = tetrahydrofuran, and Py = pyridine) have been shown to heterolytically activate H_2_*via* a 1,3-dipole generated across the ligand and metal center.^1^ Notably, reduction of the indium complex to elemental indium was observed and was attributed to the known thermal instability of indium hydrides (Scheme 1(c)).^26,27^ When the reaction was repeated in the presence of Ph_3_PSe, abstraction of selenium from Ph_3_PSe led to the formation of amorphous InSe. Similarly, the zinc aminoxide complex, [Zn(TEMPO)_2_]_2_, has been reported to react with dihydrogen to afford a zinc hydride aminoxide complex, hinting at the ability of aminoxides to support hydrogenolysis reactivity (Scheme 1(b)).^28^

Building on these earlier findings, we have sought to understand the explicit roles of the metal and supporting ligand in bond activation processes conducted by metal aminoxide complexes. Herein, we describe our investigation into the reactivity of a series of divalent metal aminoxide complexes, of Mg, Ca, Sr, Zn, Cd, Sn, and Pb, to determine the origin and generality of reactivity with dihydrogen. The activation of H_2_ by aminoxide complexes of Zn, Cd, and Pb was observed, and in the case of Cd and Pb, reduction to Cd^0^ and Pb^0^ was identified (Scheme 1(c)). Furthermore, when aminoxide complexes of Cd and Pb were treated with H_2_ in the presence of Ph_3_PSe, binary CdSe and PbSe formed, demonstrating the ability to access reductive redox state changes at room temperature facilitated by ligand design to form inorganic materials.

## Results and discussion

### Synthesis of divalent aminoxide complexes

Our group has previously synthesized group XIII aminoxide complexes using a modular synthetic procedure based on salt metathesis of NaTEMPO and metal halide.^1^ A similar procedure was also used by Kayser *et al.* to prepare chromium and molybdenum complexes bearing aminoxide ligands.^29^ In this work, treatment of the alkaline earth metal halides MgCl_2_, CaCl_2_, and SrBr_2_ with NaTEMPO (2 equiv.) in tetrahydrofuran (THF) afforded [Mg(TEMPO)_2_]_2_·2THF ([Mg]), Na_2_[Ca(TEMPO)_4_]·2THF ([Ca·Na_2_]), and Sr_2_Na_2_(TEMPO)_6_·2THF ([Sr·Na]_2_) in 87%, 70%, and 71% yield, respectively, with respect to NaTEMPO. The identities of these products were confirmed by ^1^H and ^13^C nuclear magnetic resonance (NMR) spectroscopy, quantitative ^1^H NMR spectroscopy, inductively coupled plasma mass spectrometry (ICP-MS), and single crystal X-ray diffraction (SCXRD) studies (Fig. 1).

**Fig. 1:**
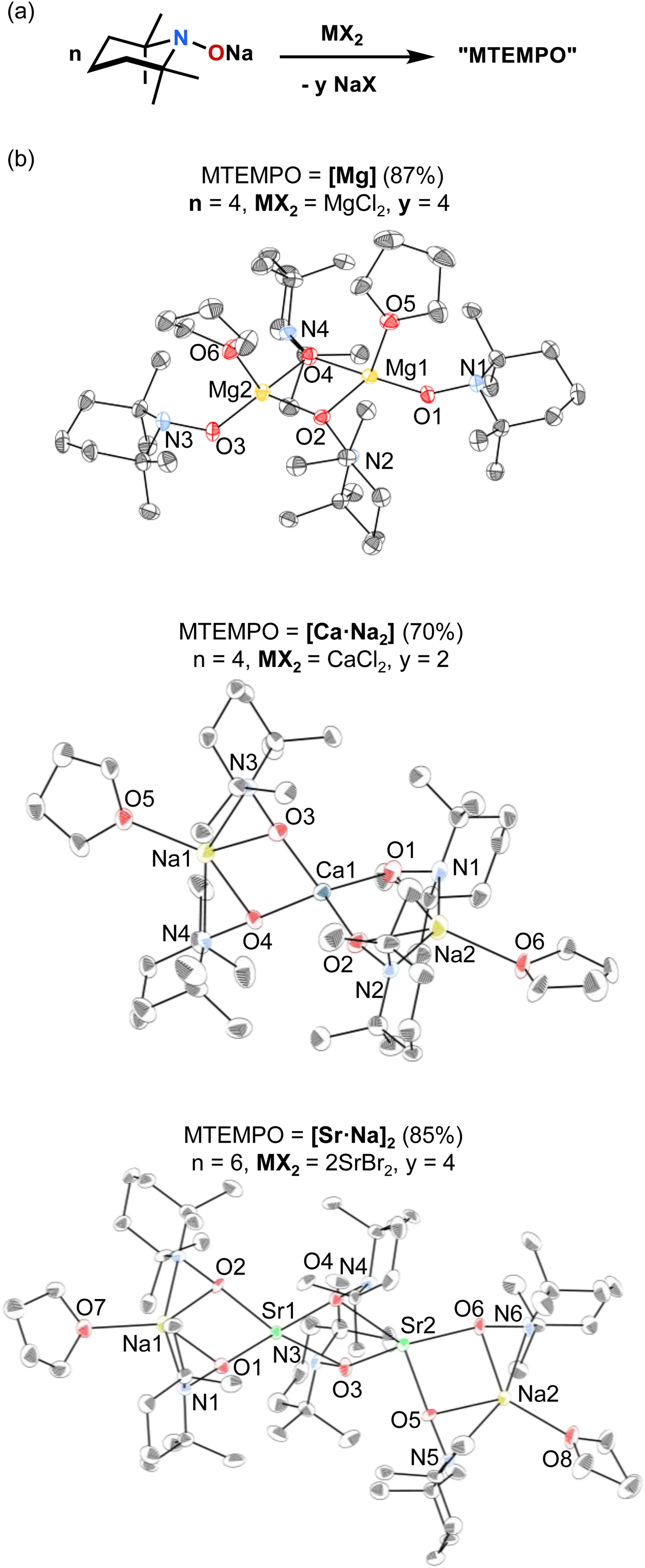
(a) Synthesis and (b) single-crystal X-ray diffraction structures of aminoxide complexes [Mg] (top), [Ca·Na_2_] (middle), and [Sr·Na]_2_ (bottom) with thermal ellipsoids shown at the 50% probability level. Hydrogen atoms are omitted for clarity.

Treatment of ZnCl_2_ with NaTEMPO (2 equiv.) in THF afforded the previously reported complex [Zn(TEMPO)_2_]_2_ ([Zn]) in 84% yield with respect to NaTEMPO and ^1^H NMR spectra are consistent with the previous report.^28^ In contrast, treatment of SnCl_2_ and NaTEMPO (2 equiv.) in THF resulted in a complex mixture of products according to ^1^H NMR spectroscopy. However, treatment of SnCl_2_ with NaTEMPO (4 equiv.) in pyridine furnished Sn(TEMPO)_2_·pyridine ([Sn]) in 85% isolated yield with respect to NaTEMPO according to ^1^H NMR spectroscopy (Fig. S18–S21).

Treatment of CdCl_2_ with NaTEMPO in THF at −35 °C for 48 h, followed by filtration of the reaction mixture with a precooled frit with a pad of Celite, afforded a solution that furnished crystals upon concentration at −35 °C. The crystals were identified as [Cd(TEMPO)_2_]_2_·2THF ([Cd]), by ^1^H and ^13^C NMR spectroscopy, ICP-MS, and SCXRD studies (Fig. 2, S22–S26, S75, Tables S5 and S16). Once crystallized, [Cd] was stable at room temperature in neat benzene-*d*_6_ for several days. In contrast, the salt metathesis of CdCl_2_ with NaTEMPO in THF at room temperature directly formed Cd^0^ as assessed by PXRD, and the absorbance spectrum of the reaction mixture revealed the presence of TEMPO (Fig. S27).

**Fig. 2:**
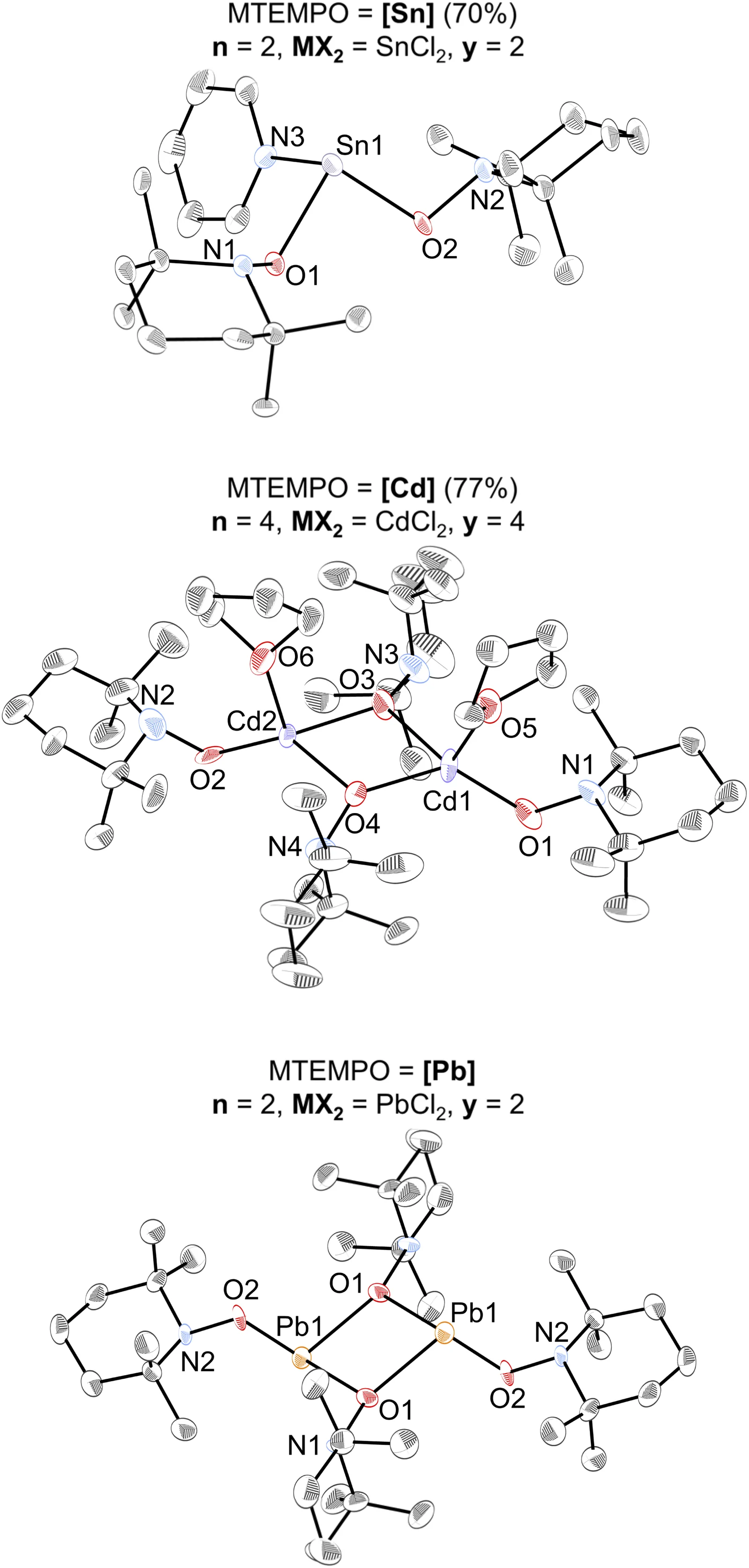
Single-crystal X-ray diffraction structures of aminoxide complexes [Sn] (top) with thermal ellipsoids shown at 50% level of probability level, [Cd] (middle) with thermal ellipsoids shown at the 50% level of probability, and [Pb] (bottom) with thermal ellipsoids shown at the 90% level of probability. Hydrogen atoms are omitted for clarity.

Similarly, treatment of PbCl_2_ with NaTEMPO in THF at −35 °C for 5 h, followed by filtration with a precooled frit with a pad of Celite afforded a solution that furnished crystals upon concentration at −35 °C. The crystals were identified as [Pb(TEMPO)_2_]_2_ ([Pb]) according to ICP-MS, and SCXRD studies (Tables S6, S17 and Fig. 2, S75). In contrast to [Cd], crystallized samples of [Pb] demonstrated poor thermal stability, degrading to black solids when redissolved in benzene-*d*_6_. Attempts to reproducibly isolate [Pb] for further analysis of the degradation products were unsuccessful. Treatment of PbCl_2_ with NaTEMPO in THF at room temperature afforded a mixture of Pb^0^, NaCl, and PbO_2_ as assessed by PXRD studies and TEMPO was observed in the UV-vis absorbance spectra of the reaction mixture within an hour (Fig. S28). We hypothesize that selection of aminoxide ligands with anodically shifted reduction potentials relative to TEMPO may combat spontaneous reduction of comparatively oxidizing metal ions such as Pb^2+^and is an active area under investigation.

The divalent ions examined here are sampled from across the periodic table with different covalent radii, metal-ligand bonding covalency, and canonical coordination numbers.^30^ SCXRD studies of [Mg], [Zn], [Cd], and [Pb] showed that dinuclear complexes are formed in the solid state and the metal centers have a coordination number of 4. The structure of [Mg] closely resembles that of [Mg(TEMPO)_2_]_2_·2THF previously prepared *via* treatment of [Mg{N(SiMe_3_)_2_}_2_(THF)_2_] or (*n*-Bu)_2_Mg with TEMPO and PhSiH_3_ in benzene-*d*_6_, with the primary difference being the presence of benzene in the unit cell of the reported structure.^31^ Solid-state structures of [Ca·Na_2_] and [Sr·Na]_2_ obtained by SCXRD experiments showed that metalate complexes were formed and the metal centers retain a coordination number of 4. [Sn] formed a mononuclear structure with a coordination number of 3 in the solid state as determined by SCXRD studies. Despite the varying ionic radii of the metals, the coordination number of the metals in the aminoxide complexes is 3 or 4, which we attribute to the large steric profile of the TEMPO ligand. Furthermore, the SCXRD structures of the compounds [Mg], [Ca·Na_2_], [Sr·Na]_2_, [Zn],^28^[Cd], [Sn], and [Pb] exhibit an average N–O bond length of 1.450 (2) Å, supporting the anionic assignment of the ligands.^32^

The longer than expected M–O(THF) bond lengths observed in the SCXRD structures of [Mg], [Ca·Na_2_], [Sr·Na]_2_, and [Cd] alluded to lability of the THF ligands, leading us to assess the structures of the compounds in solution.^33,34^ The ^1^H NMR spectra of [Mg], [Ca·Na_2_], [Sr·Na]_2_, and [Cd] in benzene-*d*_6_ displayed inequivalent TEMPO resonances and in compounds that coordinated THF, the downfield THF resonance was shifted from the expected value of 3.58 ppm to 3.63 ppm (Fig. S3, S8, S13, S14, and S22). ^1^H and ^13^C NMR spectroscopy coupled with HMBC and HSQC experiments showed that the asymmetry of the TEMPO ligands observed in the solid-state structures was maintained in benzene-*d*_6_, indicating that [Mg], [Ca·Na_2_], [Sr·Na]_2_, and [Cd] retain their dimeric structure in solution (Fig. S3–S17 and S22–S26). In contrast, spectra collected in pyridine-*d*_5_ displayed equivalent TEMPO resonances and the expected ^1^H chemical shift of THF for this solvent system, suggesting that the complexes are mononuclear in the coordinating pyridine solvent (Fig. S5, S10, and S23).^35^ UV-vis absorbance spectra of THF solutions of the metal aminoxide compounds did not exhibit the n → π* absorption of TEMPO at 455 nm, consistent with the absence of free TEMPO in the isolated products and validating the anionic assignment of the ligands (Fig. S29–S35).^36^

### Investigation of hydrogenolysis reactivity

A summary of the hydrogenolysis reactions of the divalent aminoxide complexes can be found in Scheme 2. Exposure of [Mg] (8 atm H_2_), [Ca·Na_2_] (3 atm H_2_), and [Sr·Na]_2_ (3 atm H_2_) to H_2_ in benzene-*d*_6_ at room temperature (rt) resulted in no observable reaction according to ^1^H NMR spectroscopy (Fig. S40–S42). Similarly, treatment of [Sn] with H_2_ (3 atm, rt) in benzene yielded predominantly starting material alongside trace 2,2,6,6-tetramethylpiperidin-1-ol (TEMPOH) in a 1 : 0.1 molar ratio. Analogous reaction with D_2_ formed TEMPOH rather than TEMPOD by ^1^H and ^2^H NMR spectroscopy, confirming that TEMPOH formation arose from adventitious water rather than H_2_ activation (Fig. S46 and S61–S62). In contrast, reaction of [Zn] with H_2_ (8 atm) in benzene-*d*_6_ at room temperature led to the complete consumption of [Zn] after 48 h, forming TEMPOH in 58% yield as assessed by ^1^H NMR spectroscopy with no detectable TEMPO radical formation by UV-vis spectroscopy (Fig. S43 and S44). Additionally, treatment of [Zn] with H_2_ (3 atm) in benzene-*d*_6_ at room temperature did not generate TEMPO as assessed by EPR spectroscopy as only trace TEMPO could be detected which could be ascribed to TEMPO (>5%) present in starting [Zn] (Fig. S45). Unit cell determination of crystals isolated from the reaction mixture matched the unit cell of the reported hexameric hydride [Zn(*µ*-H)(*µ*^2^-*η*^1^:*η*^1^-TEMPO)]_6_.^28^ Isotopic substitution of D_2_ for H_2_ under identical reaction conditions yielded TEMPOD (^2^H NMR: 3.49 ppm, O-D) and minor TEMPOH, again attributed to trace water, in a 9 : 1 molar ratio, implicating molecular hydrogen as the source of the proton in TEMPOH in the reaction (Fig. S55 and S56). Furthermore, [Zn] exhibited no reactivity with D_2_ in pyridine-*d*_5_ (3 atm, rt) (Fig. S57 and S58). While Jochmann *et al.* previously reported TEMPO radical formation upon hydrogenolysis of [Zn], our identification of TEMPOH may either indicate incomplete product characterization in the earlier report or be due to the differing synthetic protocols used to access [Zn].^28^

**Scheme 2:**
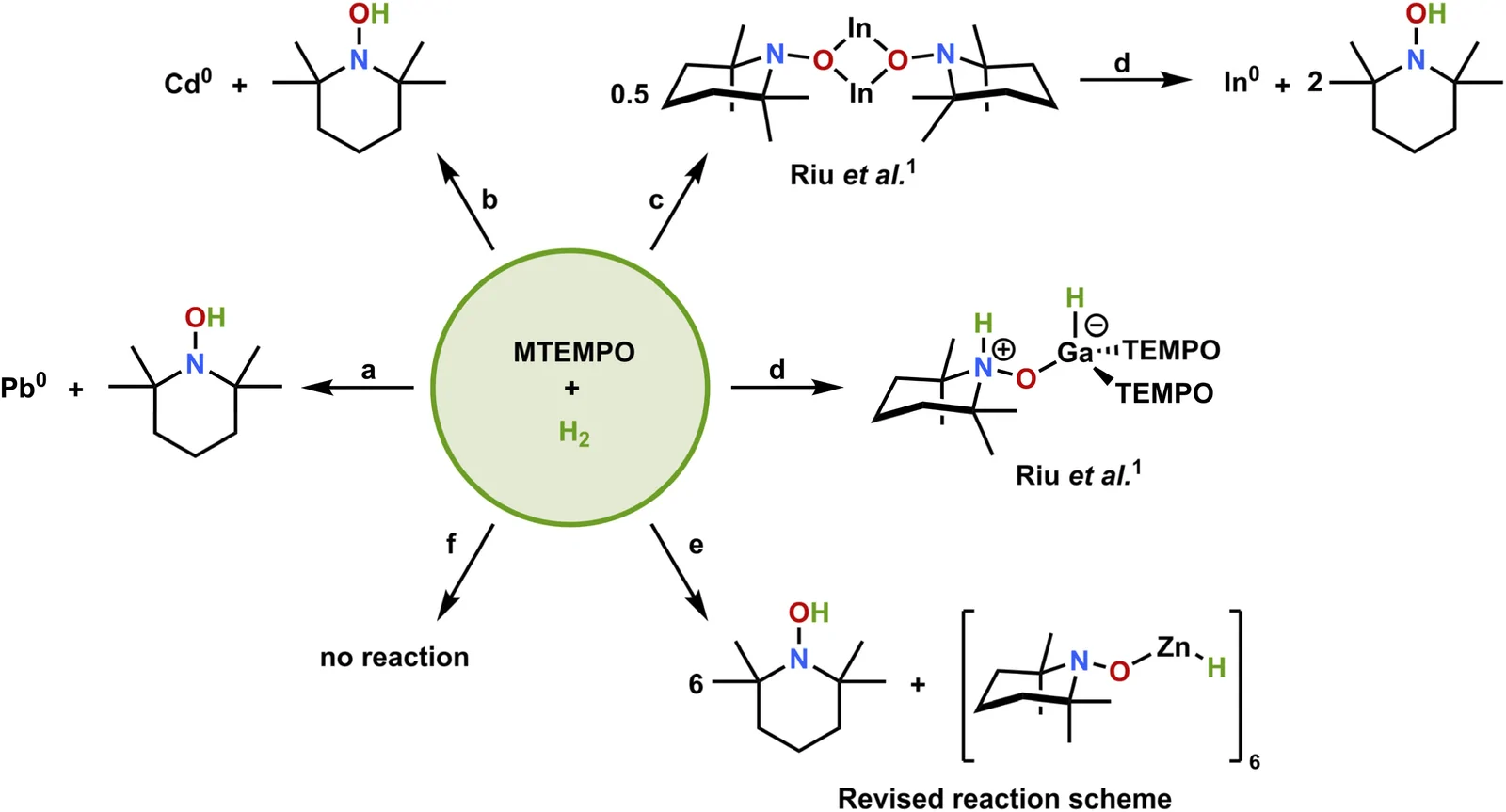
Hydrogenolysis reactions of divalent aminoxide complexes. Reaction conditions: (a) NaTEMPO, PbCl_2_, pyridine, rt. (b) [Cd], benzene-*d*_6_, rt. (c) In(TEMPO)_3_·Py, benzene-*d*_6_, rt. producing 2 equiv. TEMPOH, 1 equiv. H_2_, and [In(TEMPO)]_2_ through In(iii)-hydride intermediates, (d) Ga(TEMPO)_3_·THF, benzene-*d*_6_, rt. (e) [Zn], benzene-*d*_6_, rt. The reaction scheme has been revised from the original report by Jochmann *et al.* to include TEMPOH as the product rather than TEMPO.^28^ (f) [Mg], [Ca·Na_2_], [Sr·Na]_2_, or [Sn], benzene-*d*_6_, rt.

Hydrogenolysis of [Cd] with H_2_ (3 atm) in benzene-*d*_6_ afforded TEMPOH in 60% yield as determined by ^1^H NMR spectroscopy alongside dark solids identified as Cd^0^ by powder X-ray diffraction (PXRD) studies (Fig. S47 and S48).^37^ Substitution of H_2_ with D_2_ exclusively afforded TEMPOD according to ^1^H and ^2^H NMR spectroscopy (Fig. S59 and S60). Due to its thermal instability, [Pb] was generated *in situ* from PbCl_2_ and NaTEMPO in pyridine at −32 °C prior to H_2_ exposure (3 atm). Upon warming to room temperature, the reaction produced TEMPOH in 74% yield according to ^1^H NMR spectroscopy (Fig. S49 and S50), alongside Pb^0^ and NaCl precipitate according to PXRD analysis (Fig. S51).^38,39^ A control experiment in which PbCl_2_ was treated with H_2_ in pyridine (3 atm, rt) without NaTEMPO did not form Pb^0^ as assessed by PXRD, confirming that the aminoxide and metal ion together are required to promote H_2_ activation (Fig. S52).

The formation of zero-valent cadmium and lead deposits reflects our previous observations for In(TEMPO)_3_·Py, where hydrogenolysis yielded elemental metal and TEMPOH *via* unstable metal hydride intermediates.^25^ We propose that H–H bond activation across Cd and Pb aminoxides proceeds *via* a metal-ligand cooperative pathway, forming transient Cd–H or Pb–H species that rapidly undergo decomposition to M^0^ and H_2_.^40,41^ Similarly, our results suggest a heterolytic MLC pathway for the hydrogenolysis of [Zn].

### Evaluation of Lewis acidity *via* the Gutmann-Beckett method

To investigate whether Lewis acidity governs H_2_ reactivity of metal aminoxides, the relative Lewis acidities of the complexes were quantified using the Gutmann-Beckett method *via*^31^P{^1^H} NMR spectroscopy of Et_3_PO adducts in benzene-*d*_6_. Addition of Et_3_PO to [Mg], [Ca·Na_2_], [Sr·Na]_2_, [Zn], and [Sn] induced downfield ^31^P chemical shift differences (Δ*δ*) ranging from 4.8 to 21.1 ppm relative to free Et_3_PO, shown in Fig. 3(b). Complexes [Ca·Na_2_] and [Sr·Na]_2_ exhibited smaller shifts, which we attribute to their anionic metalate structures. For [Zn], two distinct ^31^P resonances were observed (Δ*δ* = 19.4 and 8.4 ppm); while multiple adducts typically undergo fast dynamic exchange to yield an averaged signal,^42^ discrete shifts within the same sample have been reported for divalent cations.^43^ In contrast, treatment of [Cd] with Et_3_PO resulted in the rapid formation of black solids and no observable ^31^P NMR signals, which we attribute to spontaneous coordination polymer formation. As a cautionary note, comparison of Gutmann-Beckett shifts of metal ions with widely varying ionic radii may be sensitive to solvation or steric effects, leading to complications in the direct comparison of Gutmann-Beckett shift values.^44,45^

**Fig. 3:**
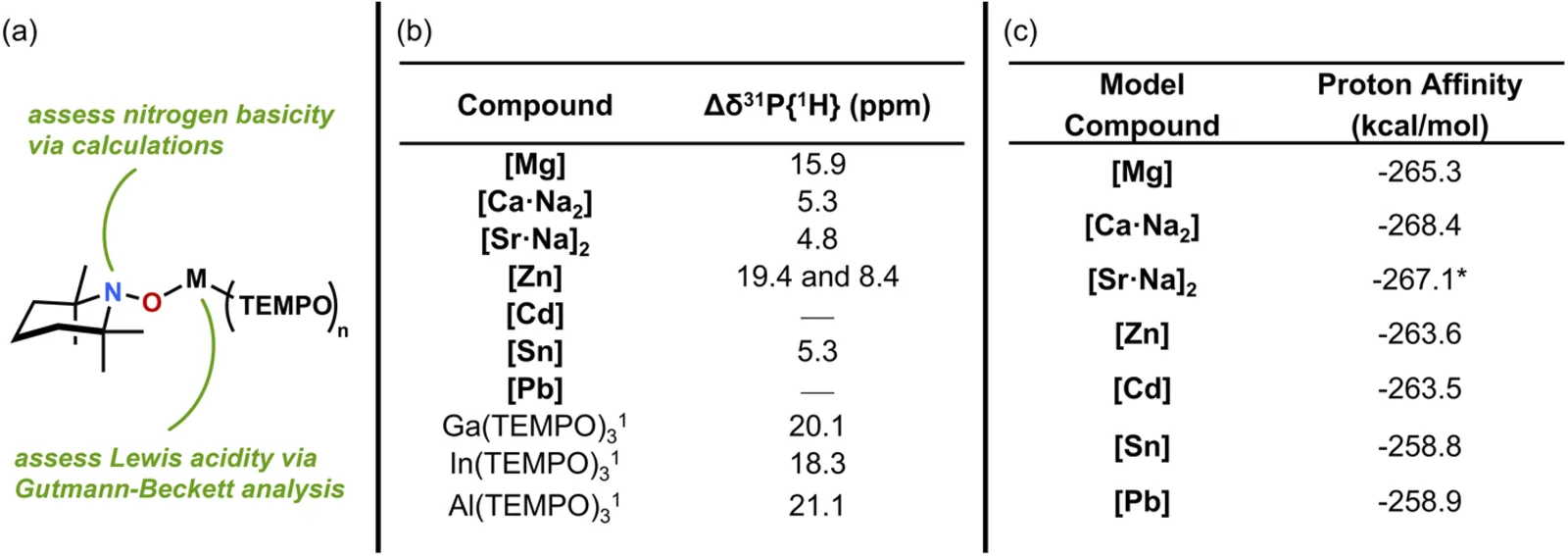
(a) Scheme of a general metal TEMPO complex. (b) Assessment of Lewis acidity, reported as the ^31^P{^1^H} NMR shift, in ppm, from Et_3_PO, using the Gutmann-Beckett method (Δ*δ*^31^P{^1^H}). (c) Assessment of the nitrogen basicity according to gas-phase proton affinity calculations. B3LYP-D4/def2-SVP//B3LYP-D4/def2-TZVPP level of theory. *Denotes a computed value based on optimized structures which displayed a minor imaginary frequency.

Across the series, a clear threshold emerges: complexes displaying a Gutmann-Beckett shift greater than approximately 16 ppm undergo hydrogenolysis, indicating that a baseline level of Lewis acidity is necessary to activate H_2_. A notable exception is the previously reported Al(TEMPO)_3_·THF, which exhibits a Δ*δ* of 21.1 ppm yet remains unreactive toward H_2_.^25^ We attribute this discrepancy to the greater electropositivity of aluminum;^46–48^ the resulting ionic Al–H bond would undergo rapid back reaction with the acidic TEMPOH (*N*-protonated) ligand, rendering net H_2_ activation thermodynamically unfavorable and highlighting the importance of bond covalency on net reactivity.

### Evaluation of nitrogen basicity *via* computations

According to calculations of the group XIII aminoxide compounds, the reaction with H_2_ proceeded through the interaction of H_2_ with the 1,3-dipole of the complex in which the metal serves as a Lewis acid and the nitrogen of the aminoxide ligand as a Lewis base.^1^ We hypothesized that differences in reactivity of the metal aminoxide complexes reported herein with H_2_ are due to a confluence of factors, including but not limited to: the Lewis acidity of the metal centers, the basicity of the respective nitrogens of the complexes, solution speciation, and coordinating ability of the solvent.

To assess the nitrogen basicity of the complexes, gas-phase proton affinity calculations were performed using coordinates derived from the SCXRD structures at the B3LYP-D4/def2-SVP//B3LYP-D4/def2-TVPP level of theory. The results extracted from computational analysis are presented in Fig. 3(c).

The proton affinity values of [Mg], [Zn], and [Cd], which exhibit metal ions in similar coordination environments, are within 2 kcal mol^−1^ of each other, indicating the nitrogens have similar basicity values irrespective of the metal center. Despite the nearly identical calculated proton affinity values, only [Zn] and [Cd] are observed to react with H_2_. Based on the computed proton affinity values, we must conclude that either (a) there is not a major role in the basicity of the nitrogen for the dipolar cleavage of H_2_, as complexes with high ([Zn] and [Cd]) and low proton affinity values ([Pb]) experimentally react with H_2_, or (b) the proton affinity values of the complexes are similar between all experimental complexes and, while nitrogen basicity is crucial for dipolar reactivity, the metal identity plays a larger role in the observed differences in reactivity. We favor the latter assumption as the oxygen atom of the M–O–N dipole may serve to attenuate electronic effects of the metal center on the nitrogen of the aminoxide and hence not substantially modulate the basicity between complexes. The combined experimental and computational results points to metal Lewis acidity, metal-hydride bond covalency, and structural differences in solution as playing a predominant role in the differing reactivity of the divalent metal aminoxide complexes with H_2_.

### Formation of metal selenides mediated by hydrogenolysis reactions

Metal hydrides of cadmium and lead are known to rapidly decompose at room temperature to form bulk metal and H_2_, making isolation and utilization of these species difficult.^40,41,49–51^ To intercept transient low-valent species prior to bulk metal precipitation, [Cd] (1 equiv.) and triphenylphosphine selenide (Ph_3_PSe) (1 equiv.) were treated with H_2_ (3 atm) in benzene-*d*_6_ at room temperature. After 24 h, ^31^P{^1^H} NMR spectroscopy indicated a 10 : 1 ratio of PPh_3_ to residual Ph_3_PSe, alongside TEMPOH formation (Fig. S64 and S65). Energy-dispersive-X-ray spectroscopy (EDS) of the resulting yellow solids formed from the reaction mixture confirmed a 1 : 1 Cd : Se ratio, while PXRD analysis showed a broad featureless pattern characteristic of amorphous material (Table S11 and Fig. 4(b)). Annealing this powder at 400 °C in a quartz tube under a flow of argon for 4 hours afforded black crystalline solids identified as CdSe by PXRD analysis (Fig. 4(c)).^52^

**Fig. 4:**
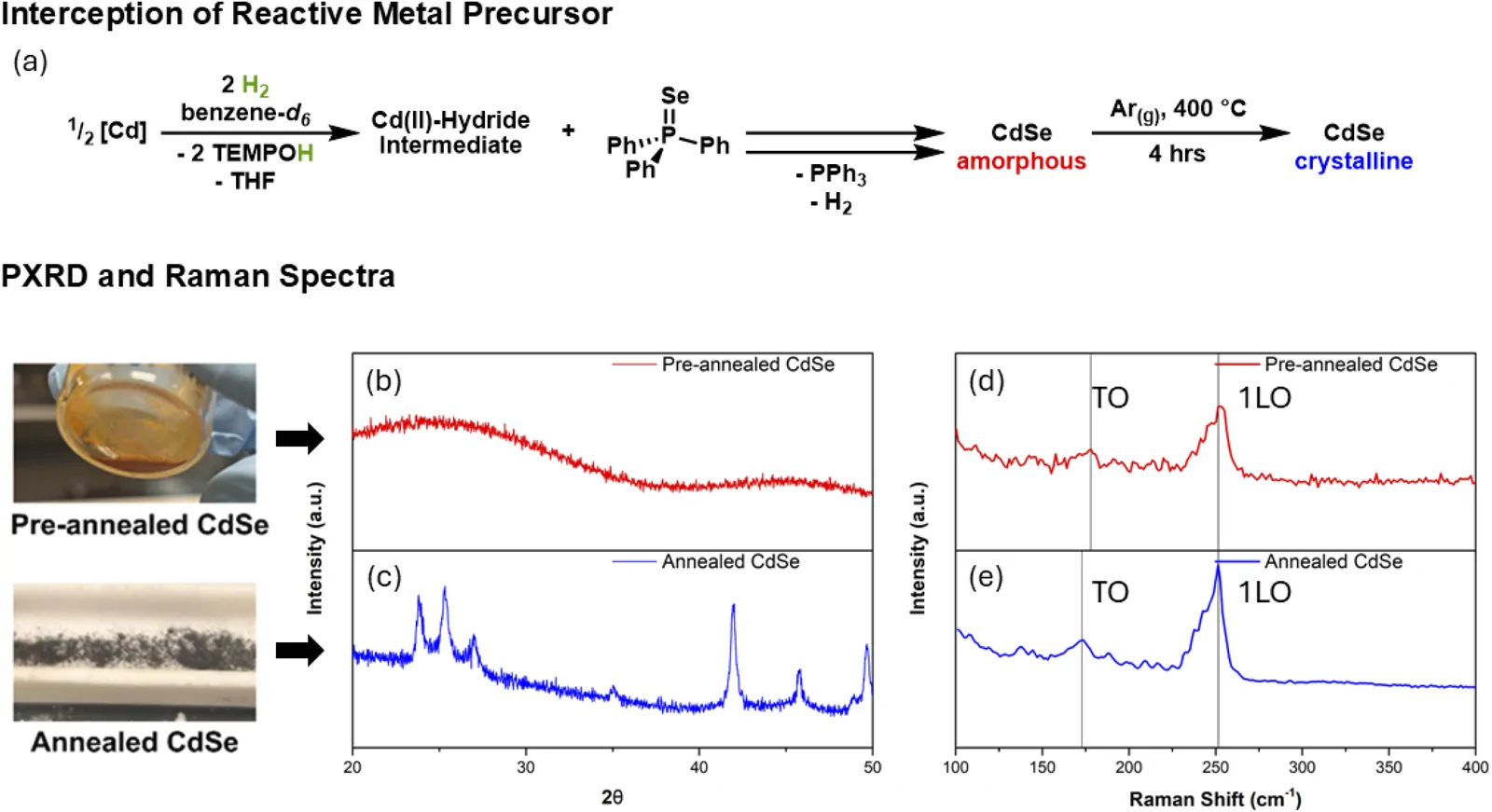
(a) Hydrogenolysis of [Cd] in the presence of Ph_3_PSe, generating amorphous CdSe before annealing, and crystalline CdSe after annealing. (b) The PXRD pattern of CdSe before annealing (c) the PXRD pattern of CdSe after annealing, (d) the Raman spectrum of CdSe before annealing, (e) the Raman spectrum of CdSe after annealing. The transverse optical (TO) and longitudinal optical (1LO) phonon modes are marked with lines for comparison.

Raman spectra of the unannealed solids displayed transverse optical (TO) and longitudinal optical (1LO) phonon modes at 178 and 250 cm^−1^, respectively (Fig. 4(d)).^53^ Upon annealing, the 1LO mode remained unchanged at 250 cm^−1^, whereas the TO mode shifted to 173 cm^−1^ (Fig. 4(e)). The 250 cm^−1^ 1LO feature is frequently attributed to lattice disorder within wurtzite-zinc blende transition zones in nanoscale CdSe;^53^ however, its retention in our crystallographically pure bulk sample suggests this spectral feature may not strictly correlate with phase disorder in bulk materials. In a control experiment, treatment of freshly prepared bulk cadmium powder with Ph_3_PSe and H_2_ (3 atm) produced no PPh_3_ after 24 h (Fig. S65), demonstrating that hydrogenolysis of [Cd] accesses a reactive synthon capable of bypassing the kinetic inertness of bulk Cd^0^.

Due to the thermal sensitivity of [Pb], the complex was generated *in situ* in pyridine. Treatment of a pyridine solution of PbCl_2_ (1 equiv.), NaTEMPO (2 equiv.), and Ph_3_PSe (1 equiv.) with H_2_ (3 atm) was conducted at 0 °C. After 24 h, ^1^H and ^31^P{^1^H} NMR spectroscopy confirmed TEMPOH formation and a 1 : 2 ratio of PPh_3_ to Ph_3_PSe, indicating the reduction of P(v) to P(iii) (Fig. S68 and S69). PXRD analysis of the isolated solids established the presence of Pb^0^, NaCl, and crystalline PbSe, with Scherrer analysis indicating an average PbSe domain size of 18 nm (Fig. S70).^38,39,54^ A control reaction combining bulk lead powder, Ph_3_PSe, and H_2_ (3 atm) in pyridine showed no reaction after 24 h by ^1^H and ^31^P{^1^H} NMR spectroscopy (Fig. S68 and S69).

Thermolysis or photolysis of molecular Cd and Pb precursors has been reported to afford putative zero-valent intermediates that react with phosphine chalcogenides.^55,56^ In contrast, this work generates such *via* MLC-mediated hydrogenolysis at room temperature. Collectively, these results suggest that H_2_-mediated reduction of divalent aminoxides generates transient zero-valent metal synthons, or reactive metal hydrides, that undergo chalcogenide transfer faster than they aggregate into bulk metal. This strategy provides a room-temperature route to metal chalcogenides using H_2_ as a clean, mild reductant and permits a broader range of co-reagents in low-temperature materials-forming reactions.

## Conclusion

We have prepared a series of divalent metal aminoxide complexes using a facile synthetic procedure. Rich structural variation is observed, with metals with smaller covalent radii forming multimetallic complexes with bridging ligands, whereas larger metals formed ionic metalates.^30^ We find that the Lewis acidity of the metal ion of the aminoxide complexes influences reactivity with H_2_, and that the metal ion does not strongly modulate the basicity of the aminoxide ligand, which provides predictive descriptors for hydrogenolysis reactivity. A key finding is that potent Lewis acid–base pairs are not strictly required for H_2_ activation. Instead, comparatively weak acidic and basic sites placed strategically within a single metal-ligand framework can access remarkable reactivity. Accordingly, activation of H_2_ at room temperature is achieved by the 1,3-dipolar intermediate generated within the metal-ligand framework, as demonstrated for [Zn], [Cd], and [Pb]. In the case of [Pb], thermal degradation pathways can be circumvented through *in situ* generation under H_2_, leading to the formation of the corresponding zero-valent metal. Amorphous CdSe and crystalline PbSe were successfully prepared at room temperature by treatment of the corresponding aminoxide complexes with H_2_ in the presence of Ph_3_PSe, demonstrating that transient metal-hydride species can be intercepted in solution by a chalcogenide source for the formation of binary materials. Although complementary routes to binary materials exist, the present work establishes a hydrogenolysis-gated, redox-based synthetic strategy in which MLC governs precursor reactivity, offering a mechanistically distinct and generalizable route to bottom-up materials synthesis under mild conditions.

## Author contributions

L. M. Zarnitsa performed all experiments with [Mg], [Ca·Na_2_], [Sr·Na]_2_, and [Zn]. O. E. Garcia performed experiments with [Sn], [Cd] and [Pb]. A. Lorber and M. M. Lopez assisted with the preparation of the compounds. All EDS experiments were performed by M.-L. Riu. DFT calculations were conducted by L. M. Zarnitsa, M.-L. Riu, and A. Talke. L. M. Zarnitsa, M.-L. Riu, and T. A. Kerr collected and interpreted the X-ray structure data. L. M. Zarnitsa and O. E. Garcia prepared the manuscript and SI. M. Nava directed the project and revised the manuscript. All authors have given approval to the original version of the manuscript.

## Conflicts of interest

There are no conflicts to declare.

## Supplementary Material

SC-OLF-D6SC07403K-s001

## Data Availability

Supplementary information (SI): full synthetic and computational details, including preparative procedures and spectroscopic data for characterization of compounds, are available in the SI. See DOI: https://doi.org/10.1039/d6sc07403k. CCDC 2504184, 2514574, 2514576, 2514633, 2514638 and 2542829 contain the supplementary crystallographic data for this paper.^57*a*–*f*^
