# Supplementary material for: Hydrogenolysis triggered formation of zero-valent metals and metal selenides at room temperature from divalent metal aminoxide complexes facilitated by metal-ligand cooperativity
Source: Chem Sci. 2026 Oct 27. Online ahead of print. doi: 10.1039/d6sc07403k (PMC13726100; doi:10.1039/d6sc07403k)
Supplement: Supplementary file 1 [file SC-OLF-D6SC07403K-s001.pdf]

Supplementary Information (SI) for Chemical Science.
This journal is © The Royal Society of Chemistry

Divalent Metal Aminoxide Complexes in Hydrogenolysis Reactions and Room-Temperature Formation of Binary Materials

Lina M. Zarnitsa,† Oliver Garcia,† Martin-Louis Y. Riu, Allison Lorber, Miguel Medina Lopez, Anneke Talke, Tyler A. Kerr, Matthew Nava*

Department of Chemistry and Biochemistry, University of California, Los Angeles, Los Angeles, California 90095, USA. † These authors contributed equally. E-mail: mjnava@ucla.edu

Table of Contents

1. General Considerations S.3
2. Preparation of Compounds S.5
   2.1 Synthesis and Characterization of NaTEMPO S.5
   2.2 Synthesis and Characterization of $[Mg(TEMPO)_2]_2 \cdot 2THF$ (**[Mg]**) S.8
   2.3 Synthesis and Characterization of $Na_2[Ca(TEMPO)_4] \cdot 2THF$ (**[Ca·Na$_2$]**) S.15
   2.4 Synthesis and Characterization of $(Na[Sr(TEMPO)_3])_2 \cdot 2THF$ (**[Sr·Na]$_2$**) S.23
   2.5 Synthesis and Characterization of $[Zn(TEMPO)_2]_2$ (**[Zn]**) S.29
   2.6 Synthesis and Characterization of $Sn(TEMPO)_2 \cdot Py$ (**[Sn]**) S.30
   2.7 Synthesis and Characterization of $[Cd(TEMPO)_2]_2 \cdot 2THF$ (**[Cd]**) S.36
   2.8 Synthesis of $[Pb(TEMPO)_2]_2$ (**[Pb]**) S.44
3. UV-Vis Absorbance Spectra S.47
4. Energy dispersive X-ray spectroscopy (EDS) data S.54
5. Reactivity with H_2 .... S.57
   5.1 Treatment of NaTEMPO with H_2: S.57
   5.2 Treatment of **[Mg]** with H_2 .... S.58
   5.3 Treatment of **[Ca·Na$_2$]** with H_2 .... S.59
   5.4 Treatment of **[Sr·Na]$_2$** with H_2 .... S.60
   5.5 Treatment of **[Zn]** with H_2 .... S.61
   5.6 Treatment of **[Sn]** with H_2 .... S.65
   5.7 Treatment of **[Cd]** with H_2 .... S.66
   5.8 Treatment of $PbCl_2$ and NaTEMPO with H_2 .... S.69
   5.9 Treatment of $PbCl_2$ with H_2 .... S.73
6. Reactivity with D_2 .... S.74
   6.1 Treatment of **[Mg]** with D_2 .... S.74

6.2 Treatment of **[Zn]** with D_2 .... S.77
7. Gutmann-Beckett Studies S.85
8. Reactivity with Ph_3PSe S.87
8.1 Treatment of **[Cd]** with Ph_3PSe, followed by H_2 .... S.87
8.2 Treatment of Cd Powder with Ph_3PSe, followed by H_2 .... S.87
8.3 Treatment of NaTEMPO and $PbCl_2$ and Ph_3PSe, followed by H_2 .... S.93
8.4 Treatment of Pb Powder with Ph_3PSe, followed by H_2 .... S.93
9. X-ray Diffraction Structures and Refinement Details S.97
10. Computational Studies S.116
10.1 General Information S.116
10.2 Cartesian Coordinates of def2-TZVPP Optimized Structures Relevant to Proton Affinity Calculations S.117
References S.160

1. General Considerations

All procedures and syntheses were performed under a nitrogen atmosphere in a Vigor glovebox unless otherwise noted. Tetrahydrofuran (THF), *n*-hexane, and hexanes were also purified by passage through columns of alumina using a solvent purification system (Pure Process Technology, Nashua, NH) and stored over 4 Å molecular sieves for at least 24 hours prior to use. Toluene and pentanes were degassed by being placed under dynamic vacuum for >15 min with stirring, then stored over 4 Å molecular sieves for at least 24 hours prior to use. Benzene-d_6 (C_6D_6) and pyridine-d_5 were degassed using the freeze-pump-thaw method and dried over 4 Å molecular sieves for at least 24 hours prior to use. Celite and 4 Å molecular sieves were dried above 200 °C under dynamic vacuum for a minimum of 36 hours prior to use. All glassware, glass microfiber filter paper, and magnetic Teflon stir bars were dried at 150 °C for a minimum of 3 hours prior to use. Unless otherwise noted, all materials are of analytical grade and purchased and used as received without further purification from Sigma Aldrich, Fisher Scientific, Strem Chemicals Inc., and Oakwood Chemical. 2,2,6,6-Tetramethyl-1-piperidinyloxy (TEMPO) was obtained from Oakwood Chemical. Ultra-high purity hydrogen gas (impurities <0.1 ppm) was purchased from Airgas and used as received. All metal halides were dried at 110 °C under dynamic vacuum for a minimum of 12 hours prior to use.

Nuclear magnetic resonance (NMR) spectra were recorded on Bruker Avance 300 MHz, Bruker Avance 400 MHz, Bruker Avance 500 MHz (equipped with a nitrogen-cooled cryoprobe), or Bruker Avance NEO 600 MHz (equipped with a nitrogen-cooled cryoprobe) spectrometers. Spectra in benzene-d_6 and pyridine-d_5 are reported in δ (parts per million) relative to residual protio-solvent signals for ^{1}H NMR spectra unless otherwise noted. Deuterated solvents (Cambridge Isotope Laboratories) were used for NMR spectroscopic analyses.

UV-Vis absorbance spectra were obtained on the Varian 5000 UV-Vis-NIR Spectrophotometer. Solutions were prepared in the glovebox in a quartz cuvette (Starna Cells) before being sealed and taken out of the glovebox for measurement.

Inductively coupled plasma mass spectrometry (ICP-MS, NexION 2000, PerkinElmer) analysis was performed to detect specified element concentrations of crystalline samples. Each sample was transferred to a clean Teflon vessel for acid digestion. Digestion was carried out with 4 mL of concentrated HNO_3 (65-70%, Trace Metal Grade, Fisher Scientific) and 1 mL H_2O_2 (30%, Certified ACS, Fisher Scientific) at 190 °C for 20 min in a microwave digestion system (Titan MPS, PerkinElmer). Once the sample was completely digested, it was subsequently diluted to make a final volume of 10 mL by adding filtered DI water for analysis. The calibration curve was established using a standard solution while the dwell time was 50 ms with thirty sweeps and three replicates with background correction.

Energy dispersive X-ray spectroscopy (EDS) experiments were performed using a Nova 230 Nano SEM.

Powder X-Ray Diffraction (PXRD) data were collected using a Panalytical X'Pert Pro X-ray Powder Diffractometer using a Cu*Kα* X-ray beam (λ = 1.5418 Å) with a step size of 0.017°, and a 0.5° slit.

Continuous-wave X-band EPR spectra were recorded on a Bruker EMXplus X-band CW EPR spectrometer operating at approximately 9.36 GHz. Samples were prepared inside a nitrogen-filled glovebox and transferred to standard quartz EPR tubes and sealed with electrical. Spectra were acquired at 298 K.

Raman data were collected using a Horiba LabRAM HR Evolution spectrometer equipped with a CVI Melles Griot laser emitting at $\lambda = 633$ nm.

2. Preparation of Compounds

2.1 Synthesis and Characterization of NaTEMPO

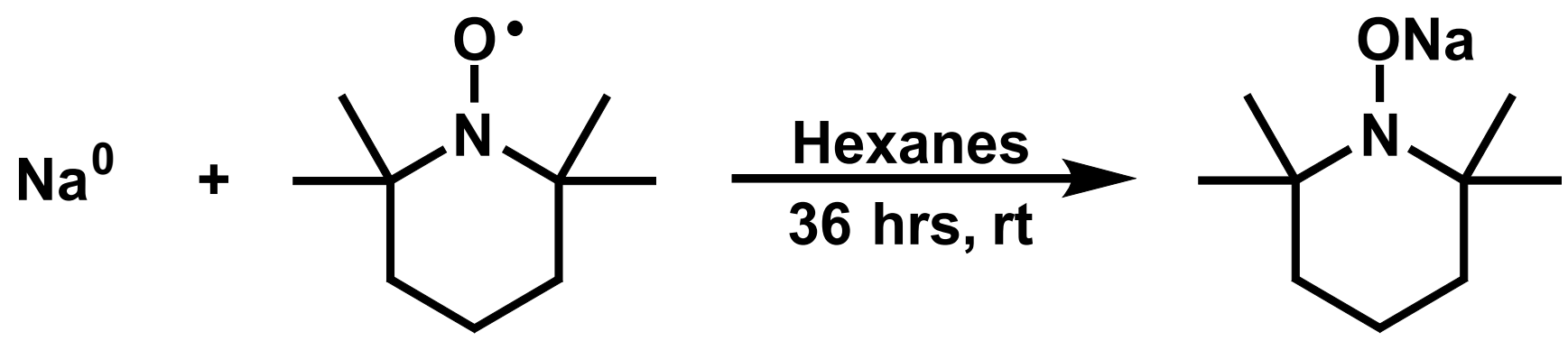

This compound was prepared according to a previously reported study with slight modifications.[1] Hexanes (250 mL) were added to sodium (1.567 g, 68.17 mmol, 1.000 equiv.), cut in approximately 0.5 cm^3 chunks, and TEMPO (10.656 g, 68.198 mmol, 1.000 equiv.). After stirring for ca. 36 hours, the light pink suspension was passed through a 60 mL medium sintered frit. Volatile materials were removed from the collected solids under dynamic vacuum to provide NaTEMPO (10.888 g, 89%) as a light pink solid. ^{1}H NMR (400 MHz, benzene-d_6, Fig. S1) δ 1.57, 1.30 ppm. ^{1}H NMR (400 MHz, pyridine-d_5, Fig. S2) δ 1.67, 1.42 ppm. The UV-vis absorbance spectrum of NaTEMPO in THF was collected and is shown in Fig. S30, and for comparison, the UV-vis absorbance spectrum of TEMPO in THF was collected and is shown in Fig. S29.

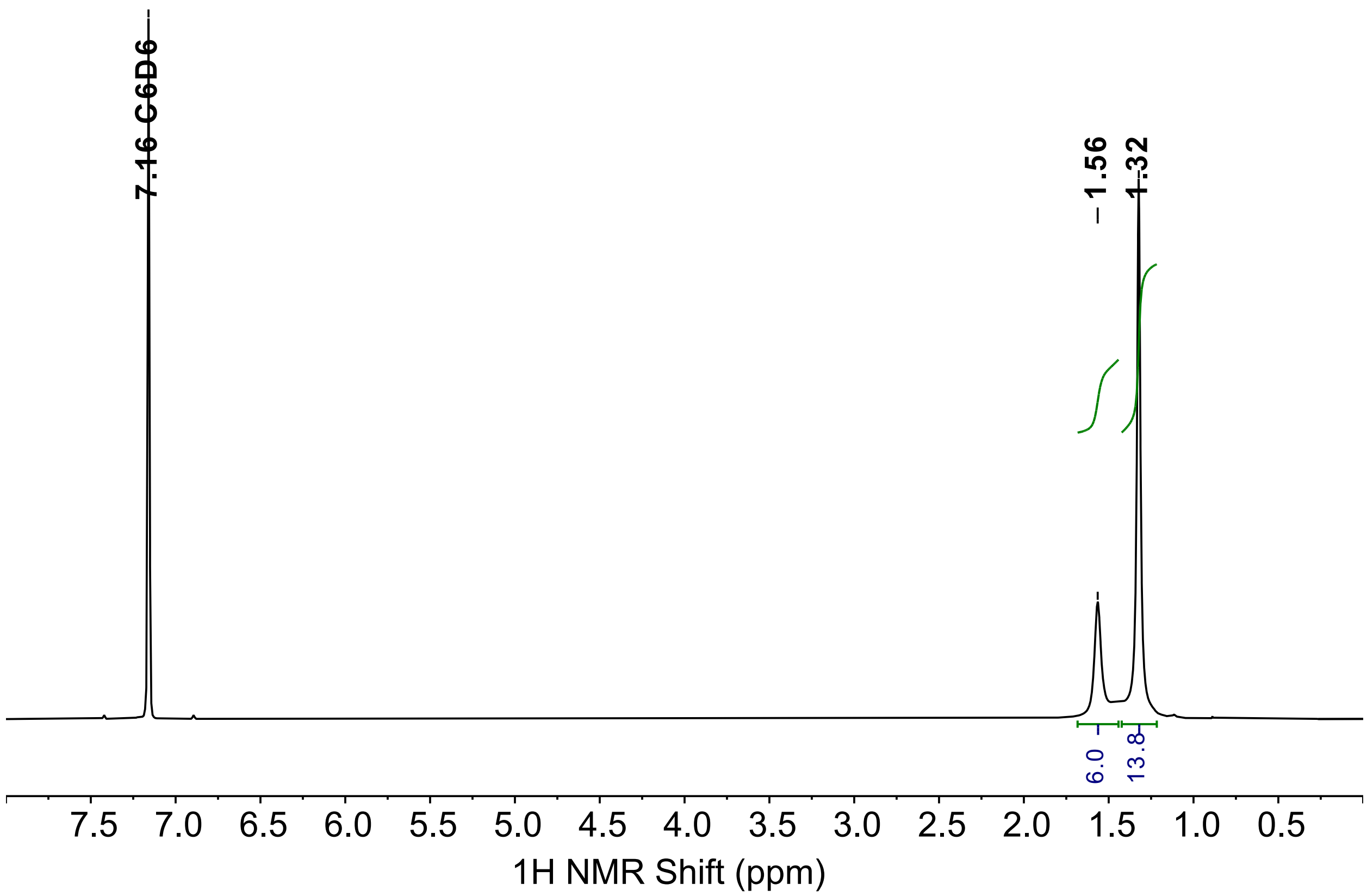

Figure S1. ^{1}H NMR (400 MHz, benzene-d_6, 25 °C) spectrum of NaTEMPO.

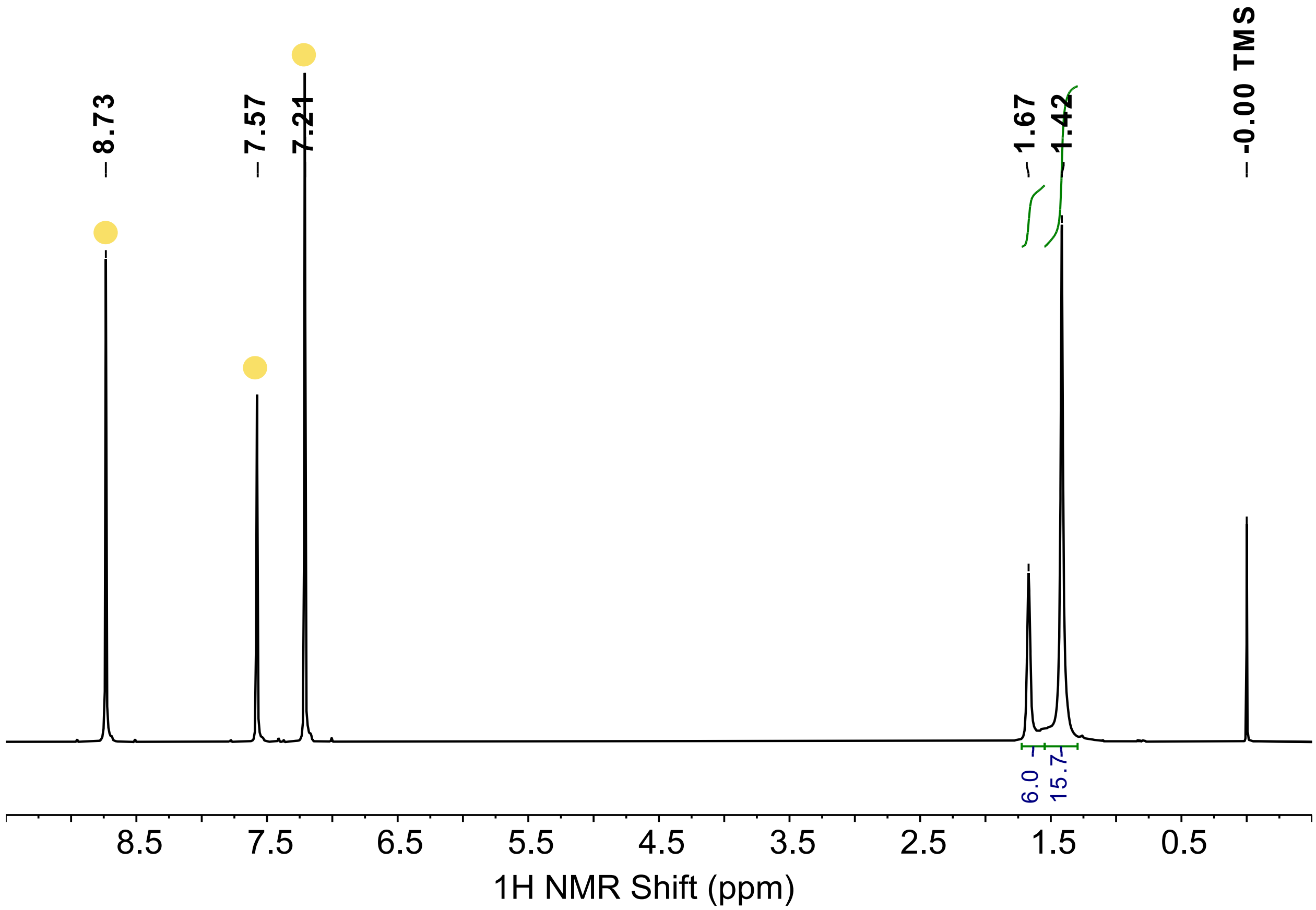

Figure S2. ^{1}H NMR (400 MHz, pyridine-d_5, 25 °C) spectrum of NaTEMPO referenced to tetramethylsilane (TMS). The yellow dots represent resonances assigned to pyridine.

2.2 Synthesis and Characterization of $[Mg(TEMPO)_2]_2 \cdot 2THF$ ([Mg])

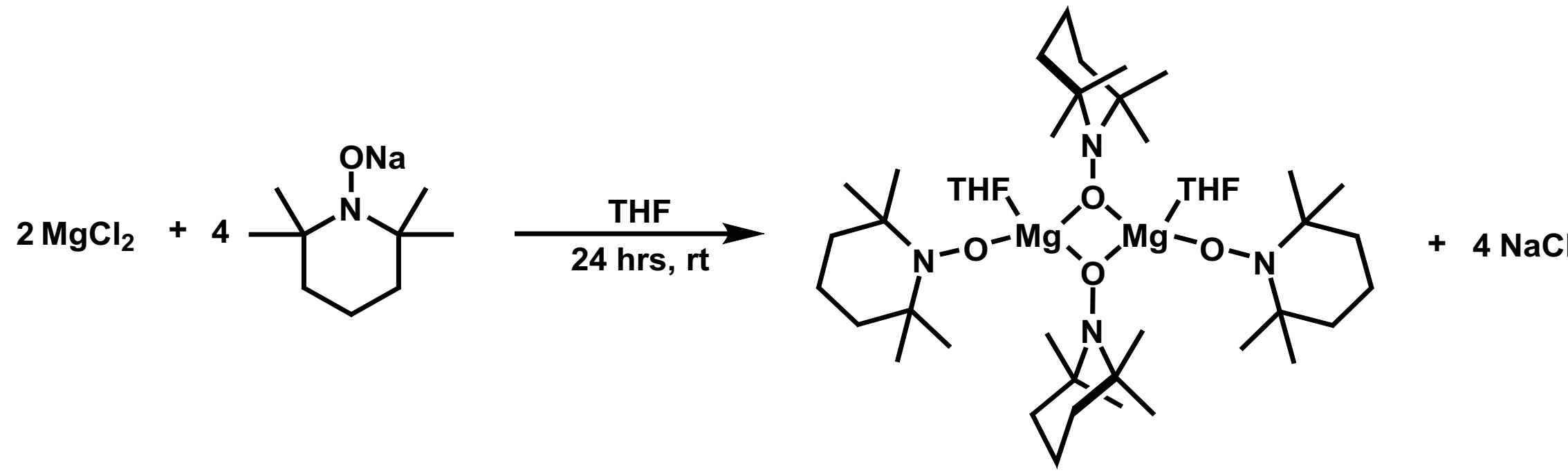

THF (100 mL) was added to NaTEMPO (2.000 g, 11.16 mmol, 2.00 equiv.) and $MgCl_2$ (0.531 g, 5.58 mmol, 1.00 equiv.). After stirring for ca. 24 hours, the suspension was passed through a 30 mL fine sintered frit containing a half-inch plug of Celite. Volatile materials were removed from the collected filtrate under dynamic vacuum to yield **[Mg]** (1.982 g, 87%). The compound can be crystallized by dissolving the solids in ca. 10 mL THF in a 20 mL scintillation vial, then placing the vial in a 50 mL vial containing ca. 20 mL toluene. This vapor diffusion setup provided **[Mg]** as a colorless crystalline solid with crystals suitable for single crystal X-ray diffraction (SCXRD). NMR spectroscopic analysis shows the complex retains a dimeric structure in benzene-d_6. $^{13}C\{^1H\}$ NMR spectroscopic assignments are shown in Fig. S4 and are supported by ^1H, ^{13}C-HMBC and ^1H, ^{13}C-HSQC experiments shown in Fig. S6-S7. While we cannot assign pairs of resonances to the bridging or terminal TEMPO, we use the color scheme to denote pairs of assignments in Fig. S4. ^1H NMR (400 MHz, benzene-d_6, Fig. S3) δ 3.64, 2.25, 1.74, 1.62, 1.46, 1.41, 1.22 ppm. $^{13}C\{^1H\}$ NMR (101 MHz, benzene-d_6, Fig. S4) δ 68.3, 60.5, 58.9, 41.2, 38.9, 35.9 (br), 33.3 (br), 25.7, 19.8 (br), 18.5 (br), 17.2 ppm. ^1H NMR (500 MHz, pyridine-d_5, Fig. S5) δ 3.66, 1.70, 1.61, 1.54, 1.44, 1.33 ppm. Repeated attempts to obtain elemental analysis data of **[Mg]** were unsuccessful, likely due to the oxygen sensitivity of the compound and the volatility of the decomposition products. ICP-MS results are reported in Table S1.

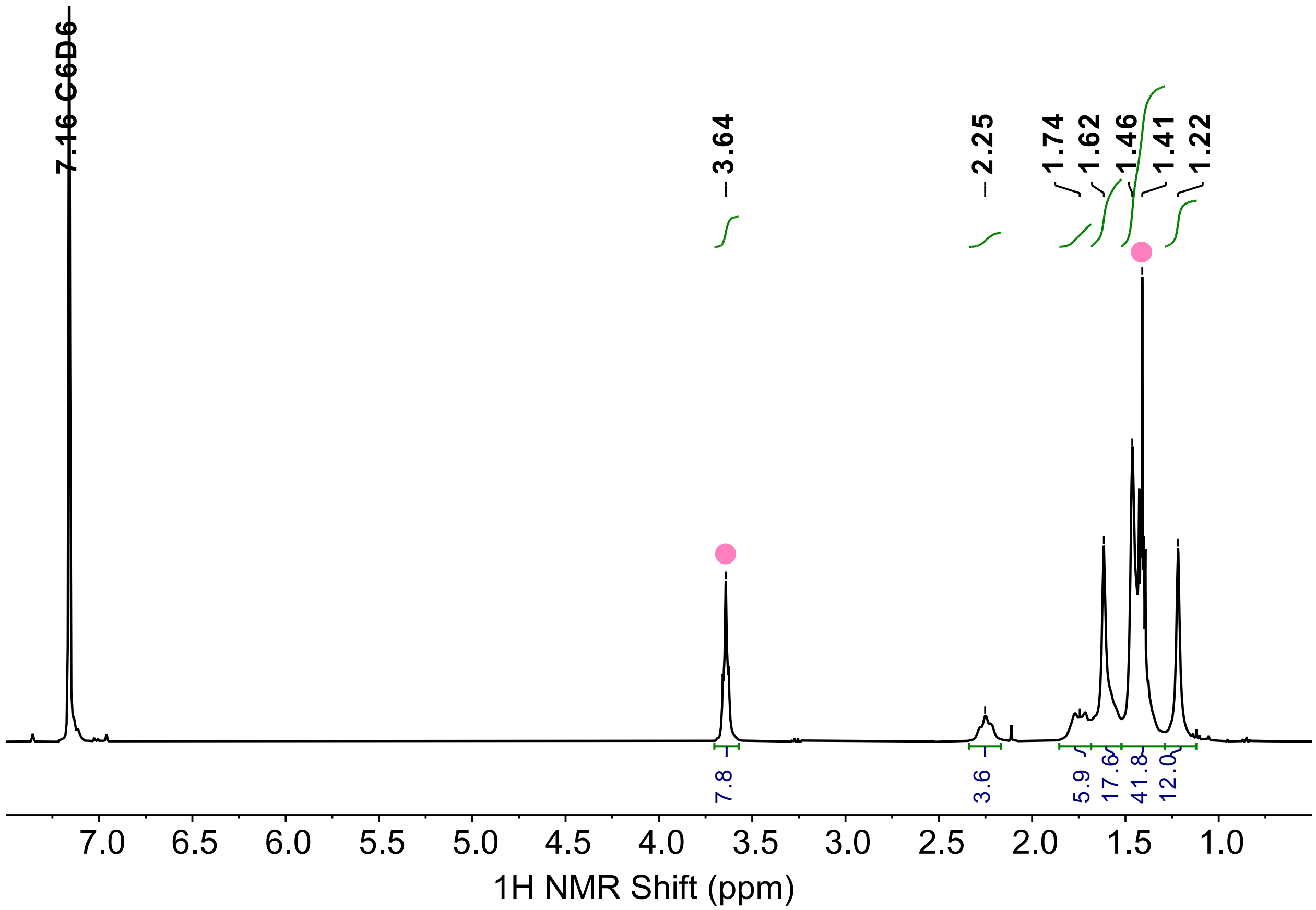

Figure S3. ^{1}H NMR (400 MHz, benzene-d_6, 25 °C) spectrum of **[Mg]**. The pink dots indicate resonances assigned to bound THF.

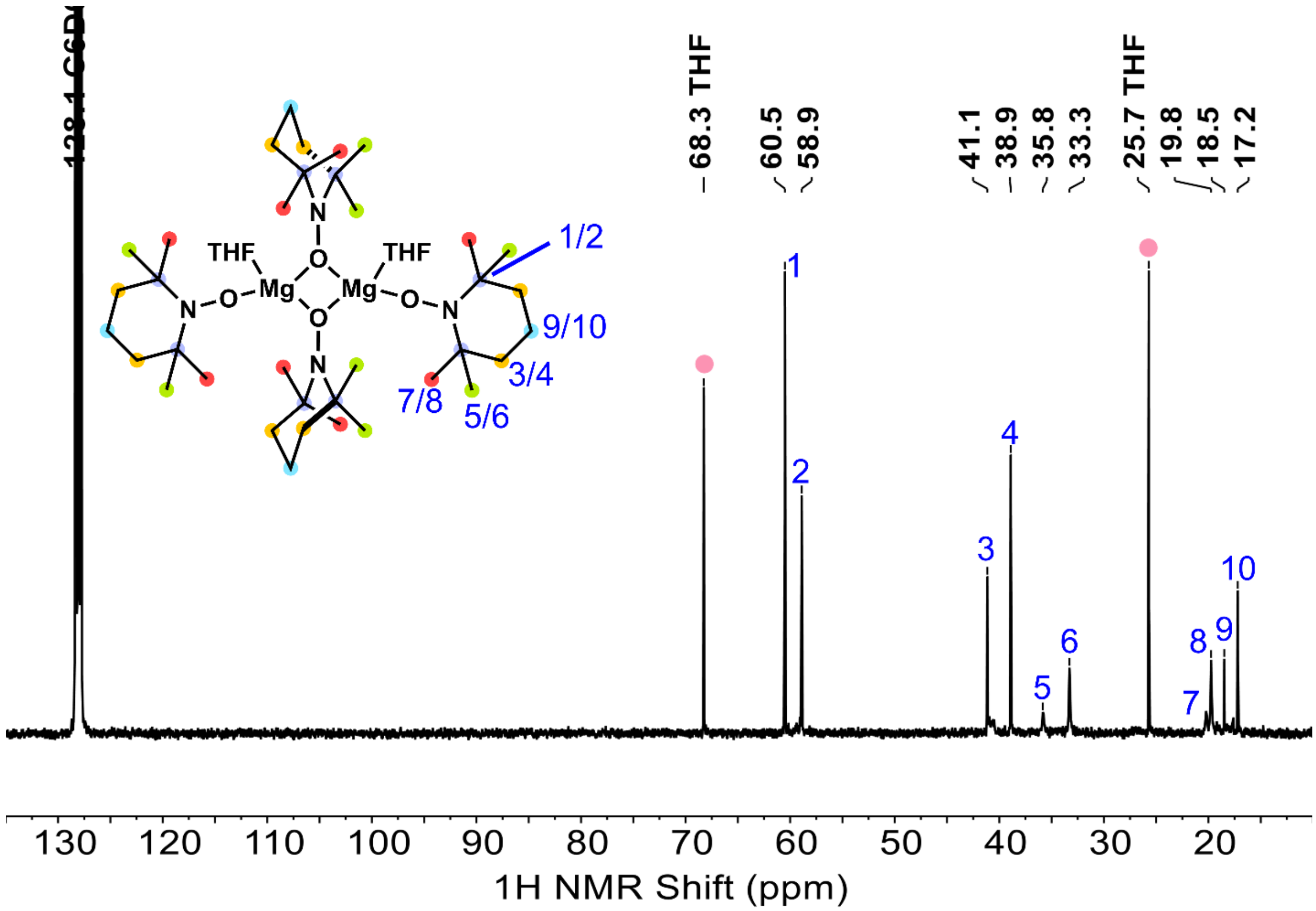

Figure S4. $^{13}C\{^1H\}$ NMR (101 MHz, benzene-d_6) spectrum of **[Mg]**. The pink dots indicate resonances assigned to bound THF. $^{13}C\{^1H\}$ NMR spectroscopic assignments are shown and are supported by ^1H, ^{13}C-HMBC and ^1H, ^{13}C-HSQC experiments shown in Fig. S6-S7. While we cannot assign pairs of resonances to the bridging or terminal TEMPO, we use the color scheme to denote pairs of assignments.

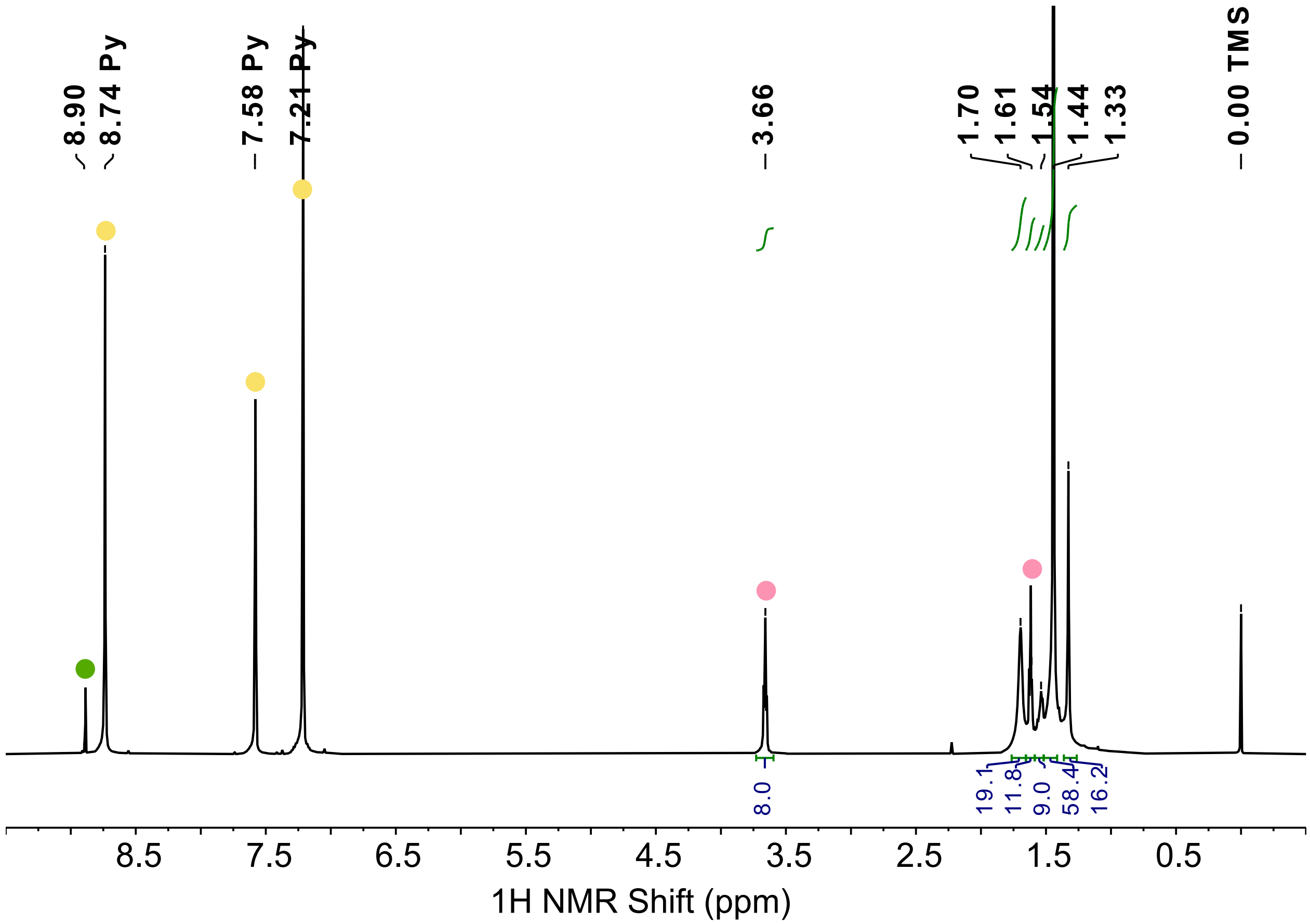

Figure S5. ^{1}H NMR (500 MHz, pyridine-d_5) spectrum of **[Mg]** referenced to tetramethylsilane (TMS). The yellow dots indicate resonances assigned to pyridine, the pink dots indicate resonances assigned to free THF, the green dot indicates a resonance assigned to unknown trace impurity.

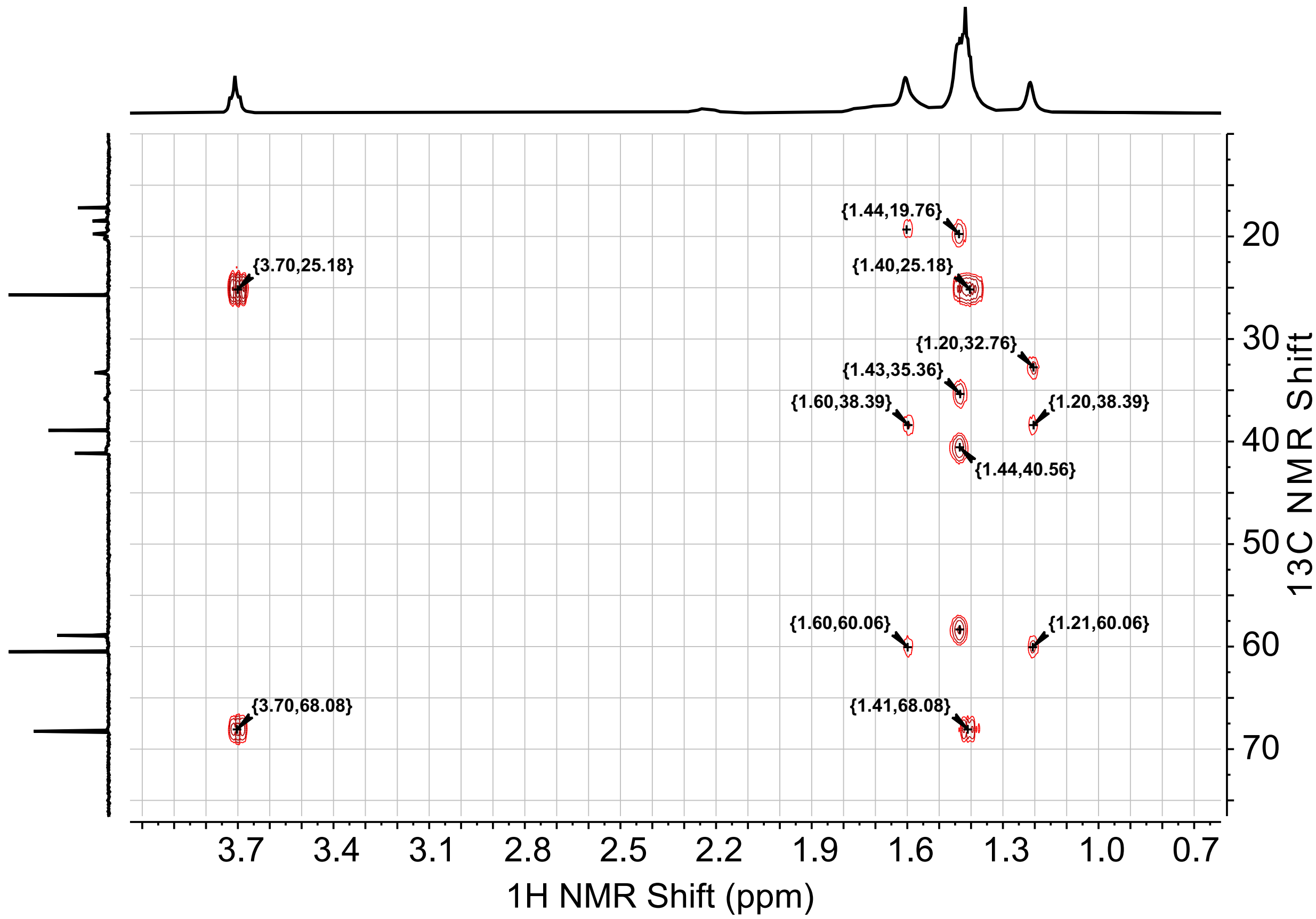

Figure S6. Multiplicity-edited ^{1}H, ^{13}C- HMBC NMR (400 MHz, benzene-d_6) spectrum of **[Mg]**.

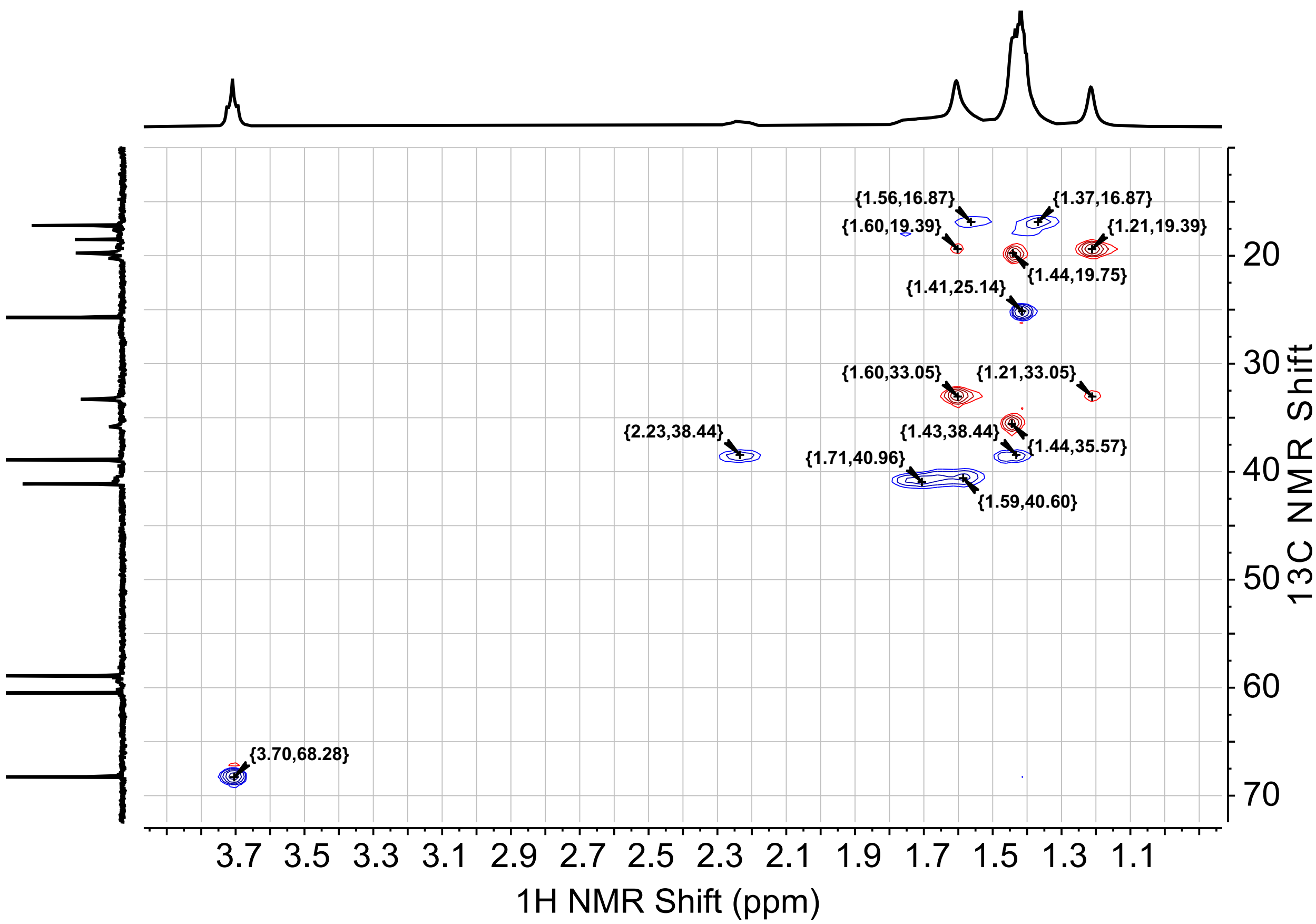

Figure S7. Multiplicity-edited ^{1}H, ^{13}C- HSQC NMR (400 MHz, benzene-d_6) spectrum of **[Mg]**.

Table S1. ICP-MS data of crystalline **[Mg]**. The expected molar ratio of the sample is 0:1 sodium to magnesium. The observed molar ratio of the sample is 0:1 sodium to magnesium. The elemental analysis by EDS suggests a molar ratio of 0:1 sodium to magnesium and showed no chloride (Fig. S36 and Table S7). ICP-MS analysis for a heteroleptic alkaline earth metal aminoxide complex has been previously used to confirm the metal ratio present in a sample.[2]

	**Sodium Concentration (μg element/mg sample)**	**Magnesium Concentration (μg element/mg sample)**
Experimental **[Mg]**	Below detection limit	146.490
Experimental standard deviation	n/a	2.974

2.3 Synthesis and Characterization of $Na_2[Ca(TEMPO)_4]\cdot 2THF$ ([Ca·Na₂])

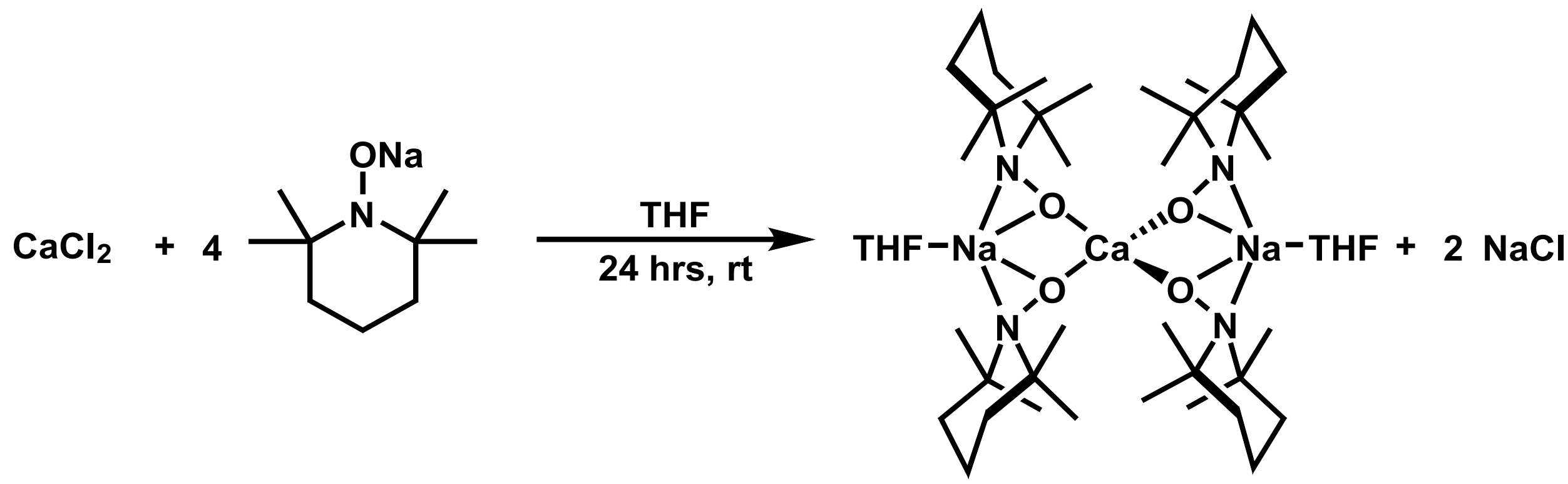

THF (100 mL) was added to NaTEMPO (2.000 g, 11.16 mmol, 2.00 equiv.) and $CaCl_2$ (0.619 g, 5.58 mmol, 1.00 equiv.)). After stirring for ca. 24 hours, the suspension was passed through a 30 mL fine sintered frit containing a half-inch plug of Celite. Volatile materials were removed from the collected filtrate under dynamic vacuum to yield **[Ca·Na$_2$]** (1.367 g, 70%). The compound can be crystallized by dissolving the solids in ca. 10 mL THF in a 20 mL scintillation vial, then placing the vial in a 50 mL vial containing ca. 20 mL toluene. This vapor diffusion setup provided **[Ca·Na$_2$]** as a colorless crystalline solid with crystals suitable for single crystal X-ray diffraction (SCXRD). NMR spectroscopic analysis shows the complex retains a dimeric structure in benzene-*d*$_6$. $^{13}C\{^1H\}$ NMR spectroscopic assignments are shown in Fig. S9 and are supported by ^1H, ^{13}C-HMBC and ^1H, ^{13}C-HSQC experiments shown in Fig. S11-S12. While we cannot assign pairs of resonances to the bridging or terminal TEMPO, we use the color scheme to denote pairs of assignments in Fig. S9. ^1H NMR (400 MHz, benzene-*d*$_6$, Fig. S8) δ 3.61, 1.64, 1.43, 1.39 1.34 ppm. $^{13}C\{^1H\}$ NMR (101 MHz, benzene-*d*$_6$, Fig. S9) δ 68.2, 58.5, 40.5, 34.3 (br) 25.7, 18.1 (br) ppm. ^1H NMR (500 MHz, pyridine-*d*$_5$, Fig. S10) δ 3.66, 1.75, 1.62, 1.52, 1.45, 1.37 ppm. To collect a quantitative ^1H NMR spectrum of **[Ca·Na$_2$]** (Fig. S13), 1,3,5-trimethoxybenzene (0.6 mL, 0.0198 M in benzene-*d*$_6$) was added to solid **[Ca·Na$_2$]** (0.030 g) and the solution was transferred to an NMR tube and data was collected with relaxation delay (D1) time of 30 s. The quantitative ^1H NMR (500 MHz, benzene-*d*$_6$, Fig. S13) spectrum of **[Ca·Na$_2$]** confirms 83% purity of the crystallized compound. Repeated attempts to obtain elemental analysis data of **[Ca·Na$_2$]** were unsuccessful, likely due to the oxygen sensitivity of the compound and the volatility of the decomposition products. ICP-MS data is shown in Table S2.

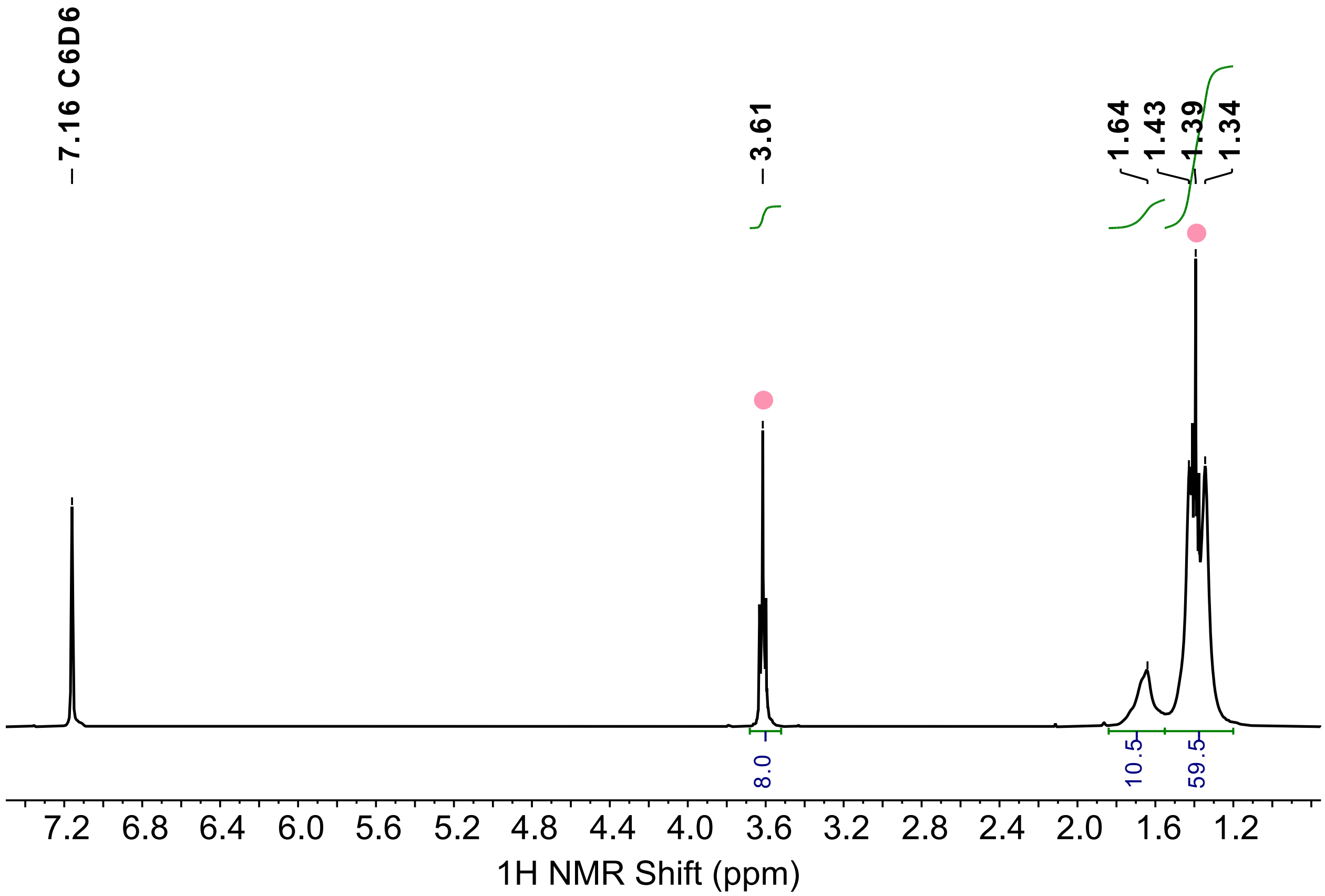

Figure S8. ^{1}H NMR (400 MHz, benzene-d_6) spectrum of **[Ca·Na$_2$]**. The pink dots indicate resonances assigned to THF.

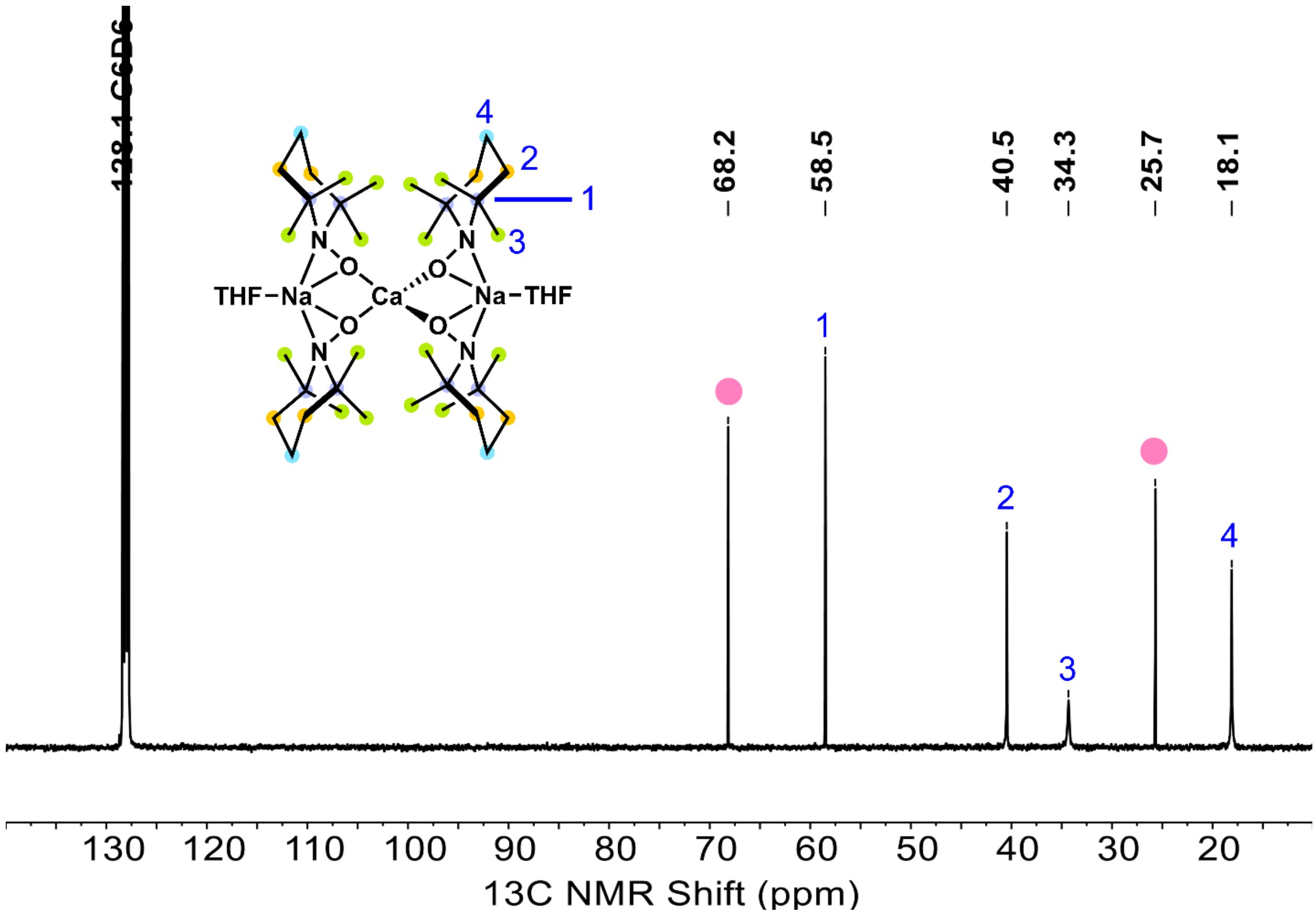

Figure S9. $^{13}C\{^1H\}$ NMR (101 MHz, benzene-d_6) spectrum of **[Ca·Na$_2$]**. The pink dots indicate resonances assigned to THF. $^{13}C\{^1H\}$ NMR spectroscopic assignments are shown and are supported by ^1H, ^{13}C-HMBC and ^1H, ^{13}C-HSQC experiments shown in Fig. S11-S12. While we cannot assign pairs of resonances to the bridging or terminal TEMPO, we use the color scheme to denote pairs of assignments.

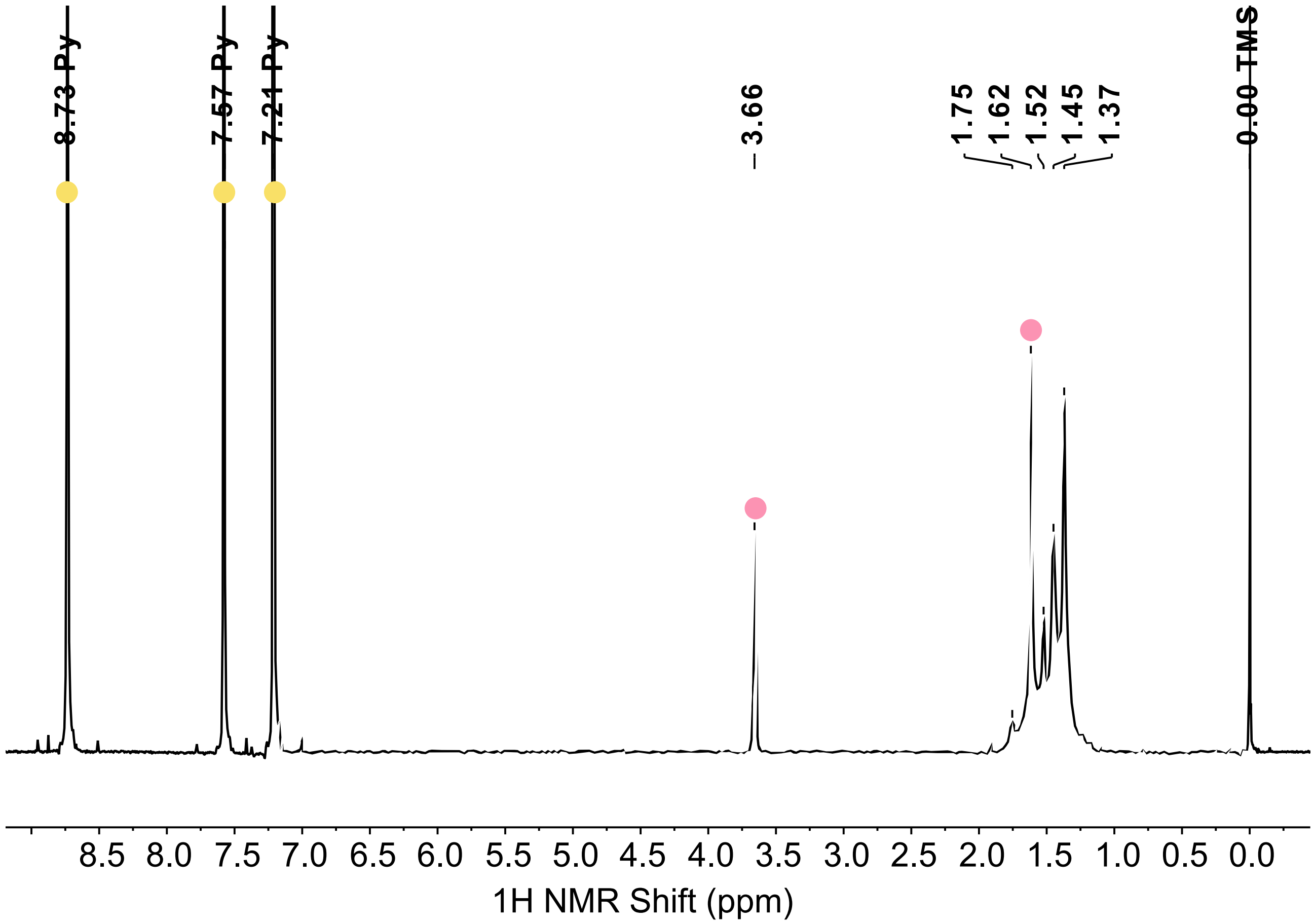

Figure S10. ^{1}H NMR (400 MHz, pyridine-d_5) spectrum of **[Ca·Na$_2$]** referenced to tetramethylsilane (TMS) present in the NMR solvent. The yellow dots indicate resonances assigned to pyridine, the pink dots indicate resonances assigned to THF.

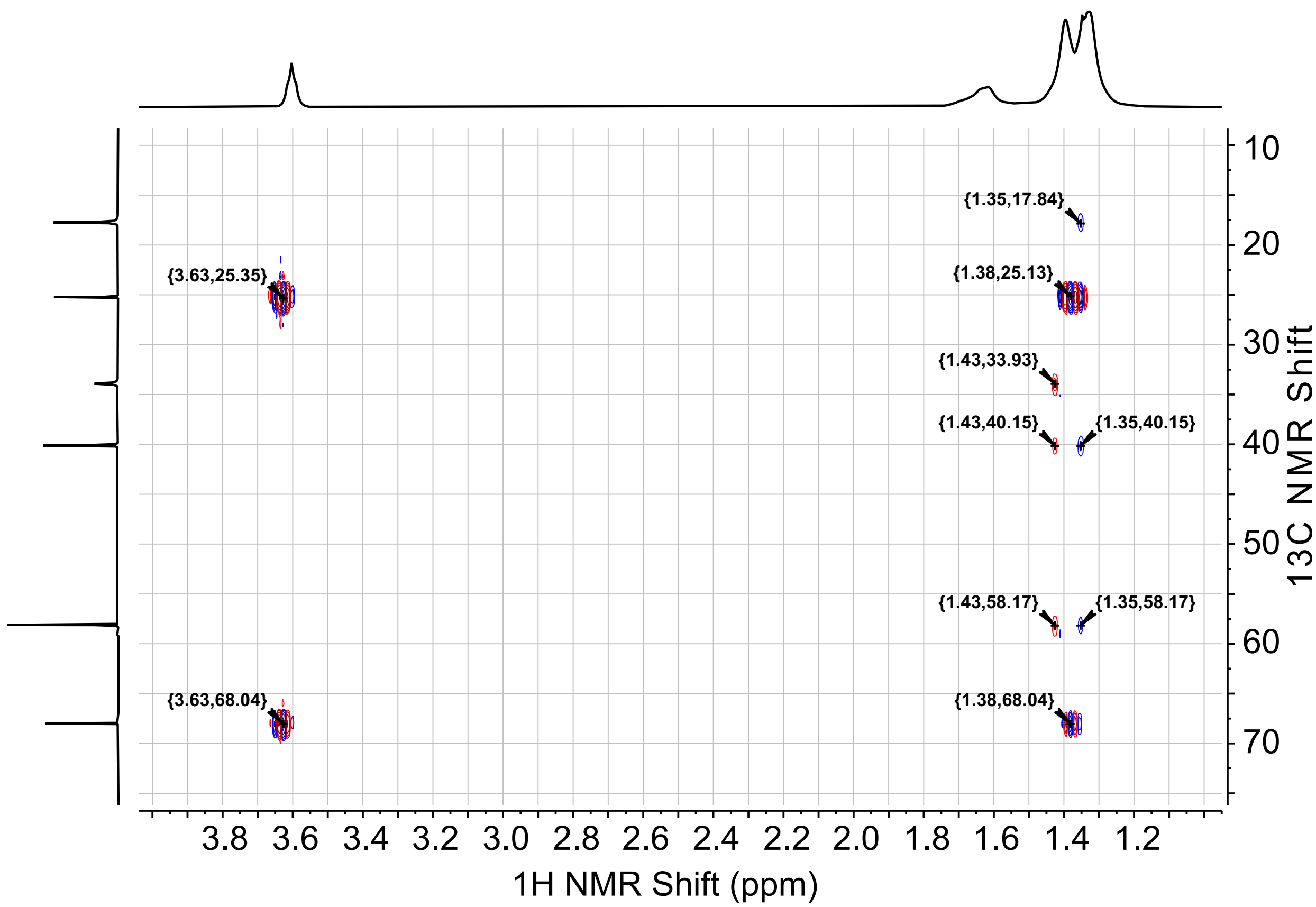

Figure S11. Multiplicity-edited ^{1}H, ^{13}C- HSQC NMR (400 MHz, benzene-d_6) spectrum of **[Ca·Na₂]**.

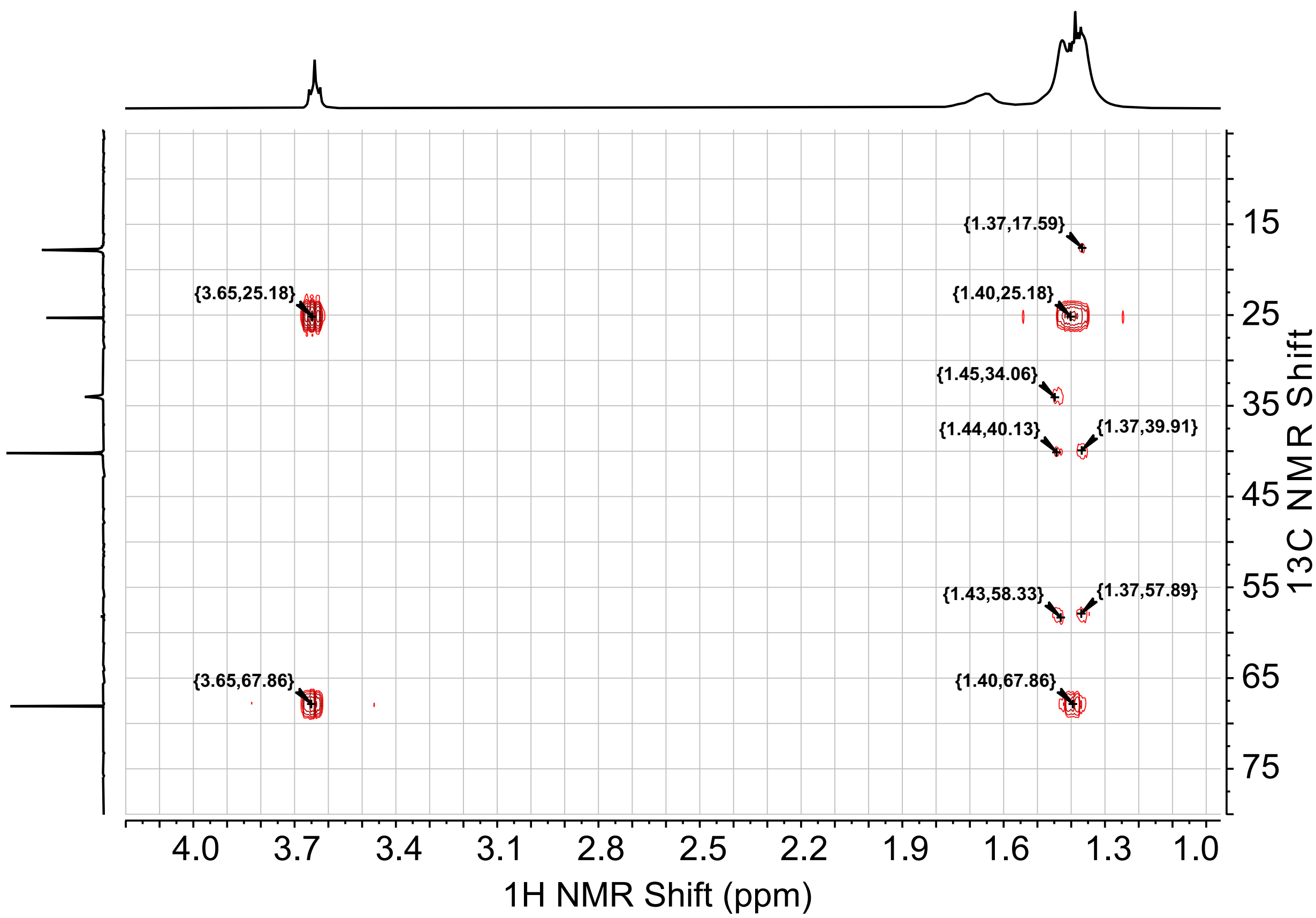

Figure S12. ^{1}H, ^{13}C-HMBC NMR (400 MHz, benzene-d_6) spectrum of **[Ca·Na$_2$]**.

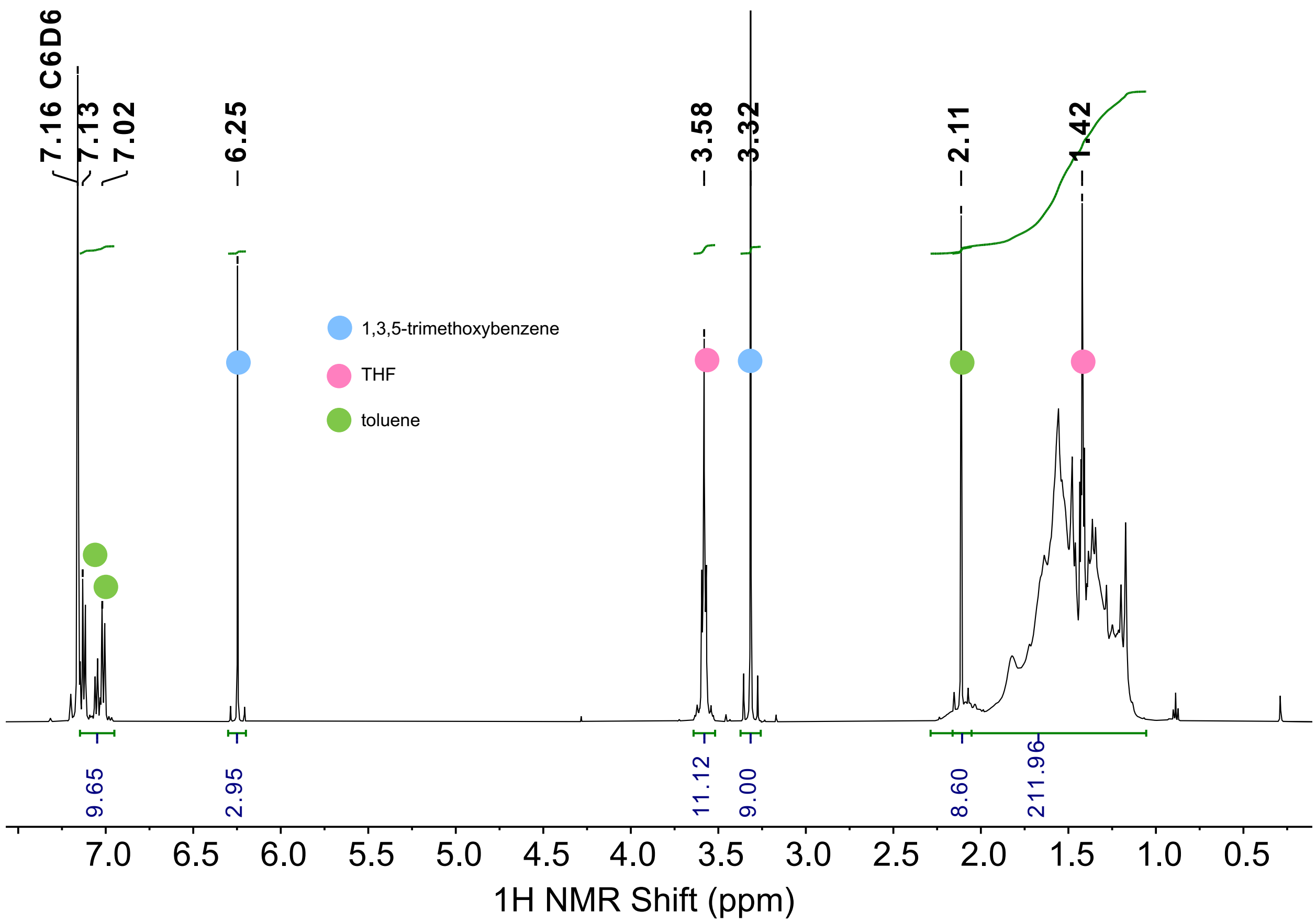

Figure S13. Quantitative ^{1}H NMR (500 MHz, benzene-d_6) spectrum of **[Ca·Na$_2$]** and 1,3,5-trimethoxybenzene.

Table S2. ICP-MS data of crystalline **[Ca·Na$_2$].** The expected molar ratio of the sample is 2:1 sodium to calcium. The observed molar ratio of the sample is 3.6931:1.0000 sodium to calcium. The elemental analysis by EDS suggests a molar ratio of 2.9:1 sodium to calcium and showed no chloride (Fig. S37 and Table S8). ICP-MS analysis for a heteroleptic alkaline earth metal aminoxide complex has been previously used to confirm the metal ratio present in a sample.[2]

	**Sodium Concentration (µg element/mg sample)**	**Calcium Concentration (µg element/mg sample)**
Experimental **[Ca·Na$_2$]**	26.324	12.426
Experimental standard deviation	0.290	0.739

2.4 Synthesis and Characterization of $(Na[Sr(TEMPO)_3])_2 \cdot 2THF$ ($[Sr \cdot Na]_2$)

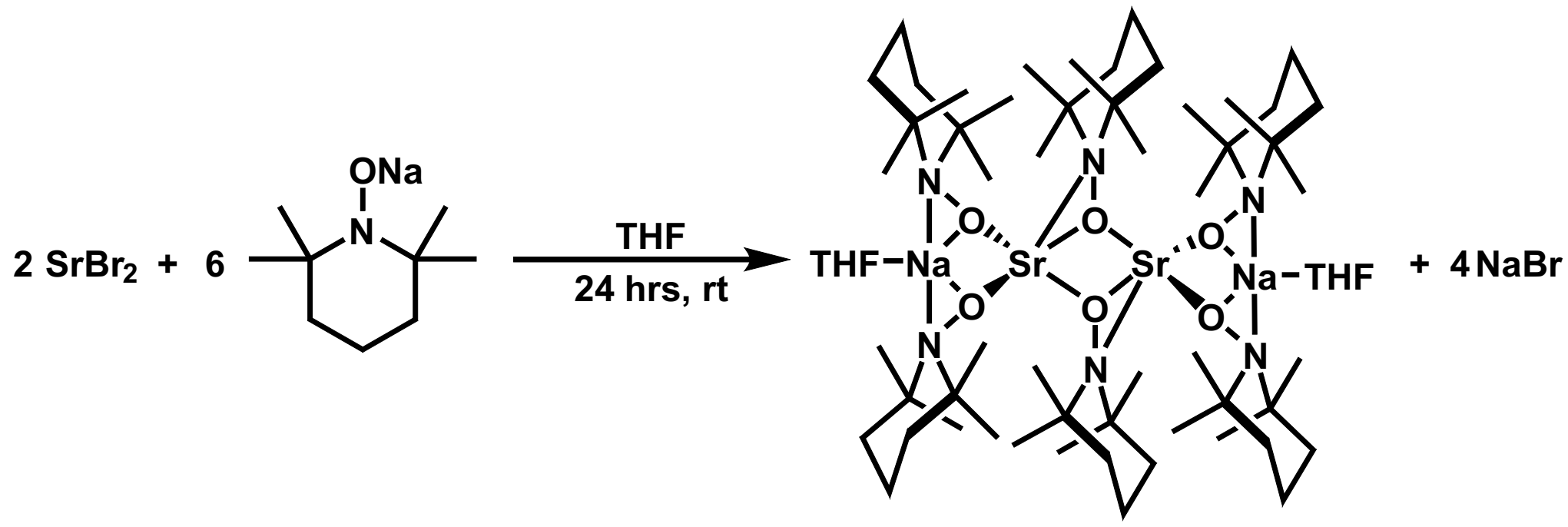

THF (100 mL) was added to NaTEMPO (0.498 g, 2.78 mmol, 2.99 equiv.) and $SrBr_2$ (0.230 g, 0.930 mmol, 1.00 equiv.). After stirring for ca. 24 hours, the suspension was passed through a 30 mL fine sintered frit containing a half-inch plug of Celite. Volatile materials were removed from the collected filtrate under dynamic vacuum to yield **$[Sr \cdot Na]_2$** (0.277 g, 71%). The compound can be crystallized by dissolving the solids in ca. 10 mL THF in a 20 mL scintillation vial, then placing the vial in a 50 mL vial containing ca. 20 mL toluene. This vapor diffusion setup provided **$[Sr \cdot Na]_2$** as a colorless crystalline solid with crystals suitable for single crystal X-ray diffraction (SCXRD). NMR spectroscopic analysis shows the complex retains a dimeric structure of $Na[Sr(TEMPO)_3] \cdot THF$ in benzene-d_6. $^{13}C\{^1H\}$ NMR spectroscopic assignments are shown in Fig. S15 and are supported by ^1H, ^{13}C-HMBC and ^1H, ^{13}C-HSQC experiments shown in Fig. S16-S17. While we cannot assign pairs of resonances to the bridging or terminal TEMPO, we use the color scheme to denote pairs of assignments in Fig. S15. ^1H NMR (400 MHz, benzene-d_6, Fig. S14) δ 3.60, 2.11, 1.80, 1.76, 1.68, 1.53, 1.41, 1.33 ppm. $^{13}C\{^1H\}$ NMR (101 MHz, benzene-d_6, Fig. S15) δ 59.2, 59.0, 58.3, 40.7, 40.6, 34.7 (br), 20.2 (br), 17.9 (br), 15.6 ppm. Repeated attempts to obtain elemental analysis data of **$[Sr \cdot Na]_2$** were unsuccessful, likely due to the volatility and oxygen sensitivity of the compound. ICP-MS data is shown in Table S3.

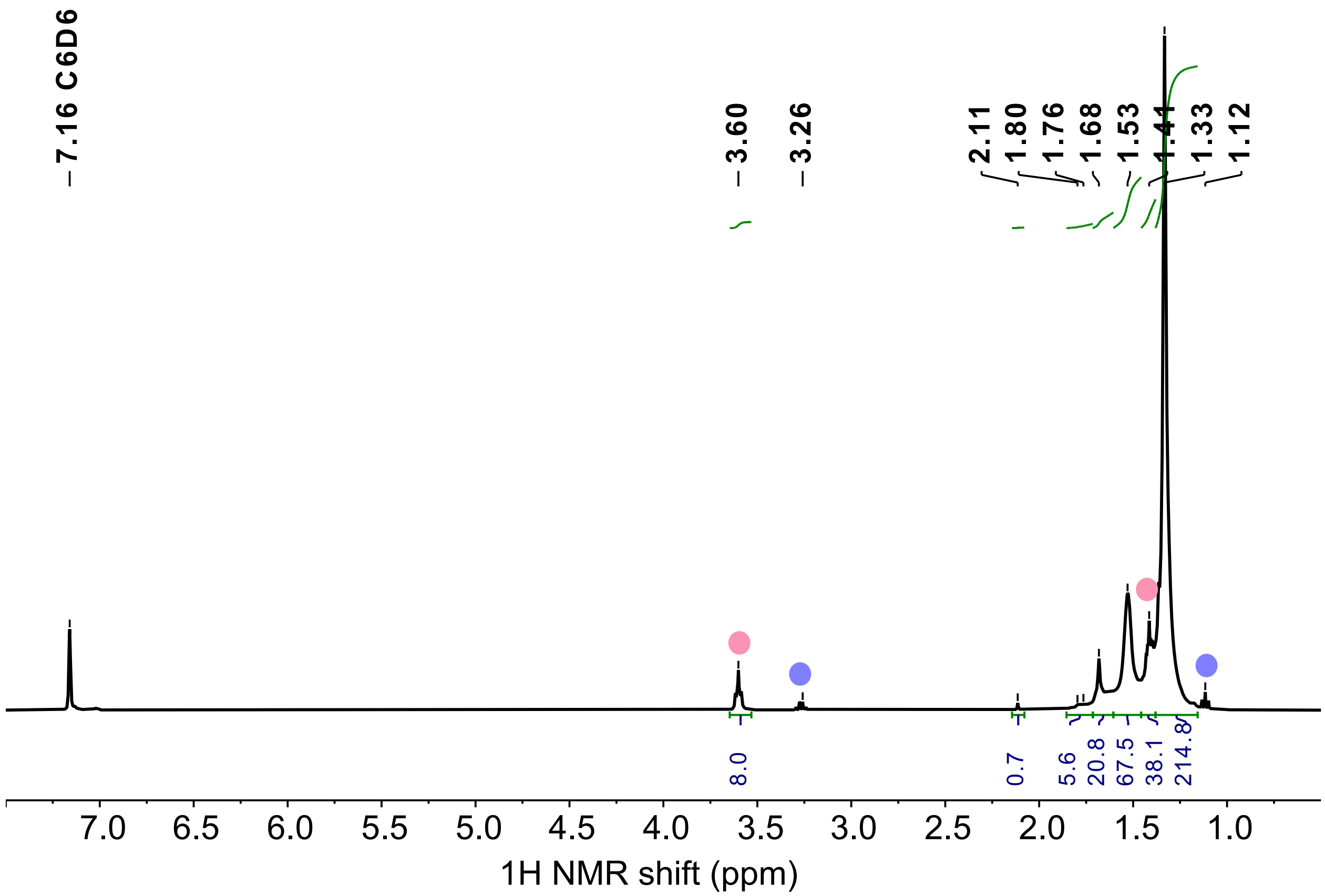

Figure S14. ^{1}H NMR (400 MHz, benzene-d_6, 25 °C) spectrum of **[Sr·Na]$_2$.** The pink dots indicate resonances assigned THF, the purple dots indicate resonances assigned to free diethyl ether which was present in the NMR solvent.

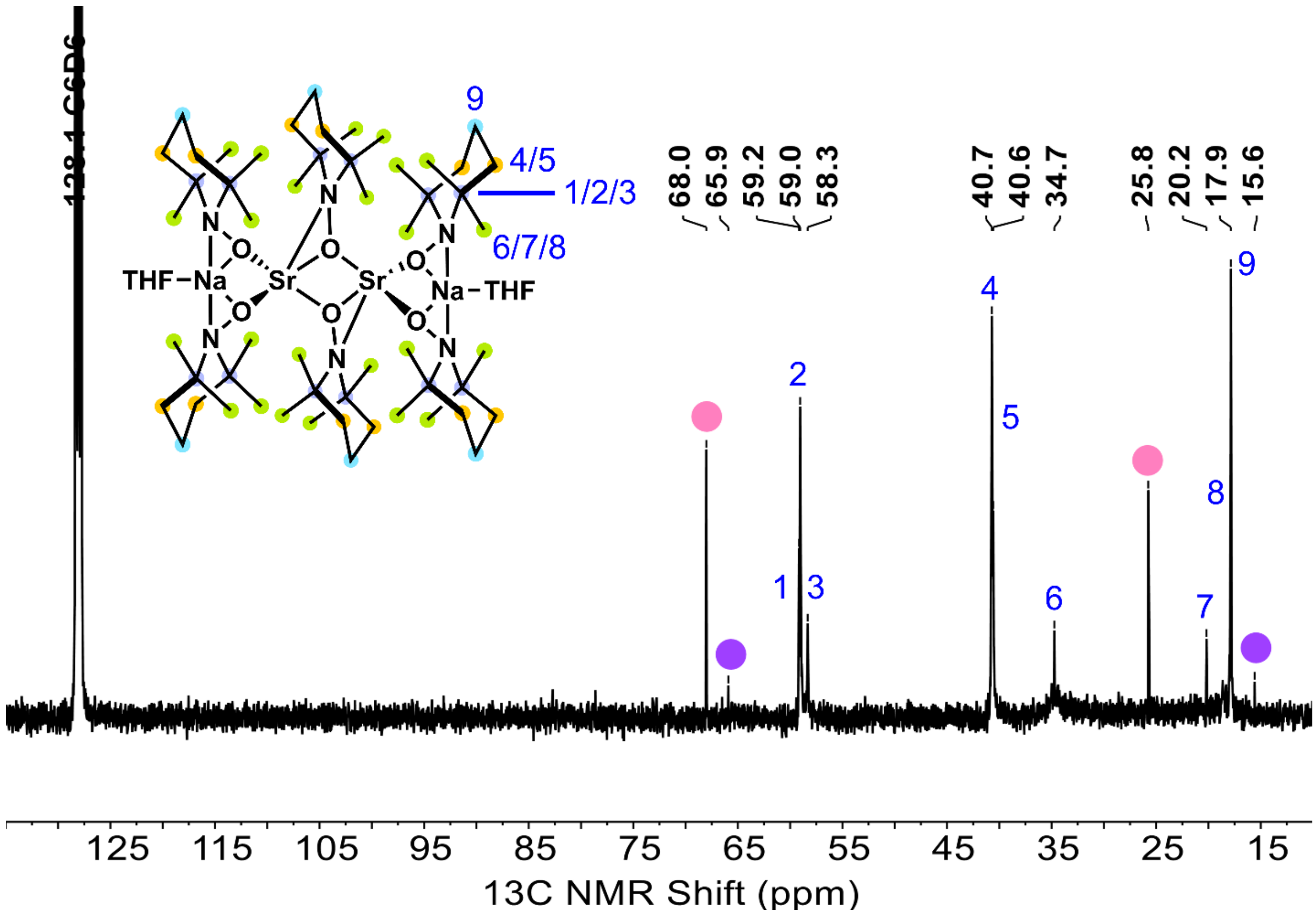

Figure S15. $^{13}C\{^{1}H\}$ NMR (101 MHz, benzene-d_6) spectrum of **[Sr·Na]₂**. The pink dots indicate resonances assigned to THF, the purple dots indicate resonances assigned to diethyl ether which was present in the NMR solvent. $^{13}C\{^{1}H\}$ NMR spectroscopic assignments are shown and are supported by ^{1}H, ^{13}C-HMBC and ^{1}H, ^{13}C-HSQC experiments shown in Fig. S16-S17. While we cannot assign pairs of resonances to the bridging or terminal TEMPO, we use the color scheme to denote pairs of assignments.

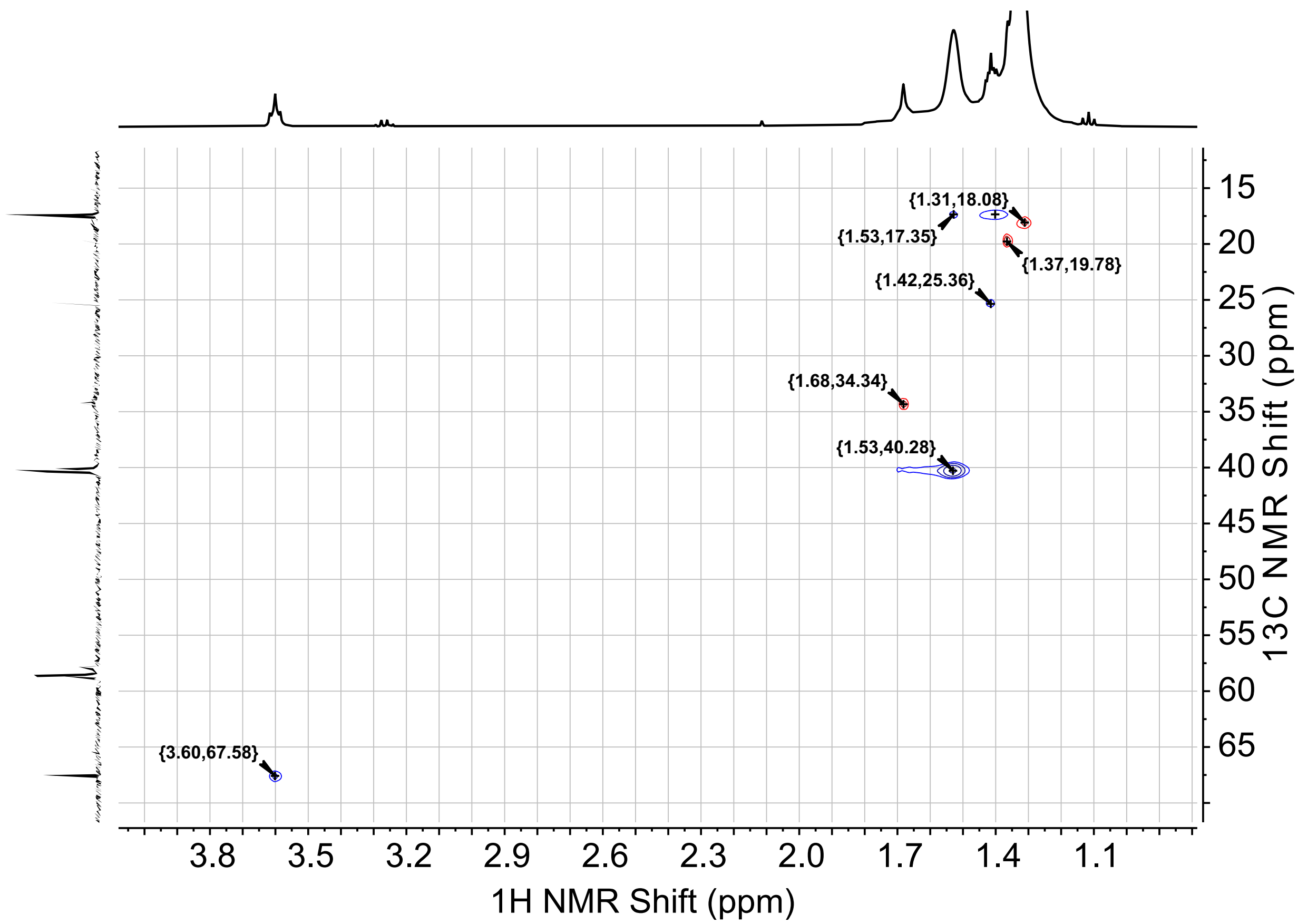

Figure S16. Multiplicity-edited ^{1}H, ^{13}C- HSQC NMR (400 MHz, benzene-d_6) spectrum of **[Sr·Na]$_2$**.

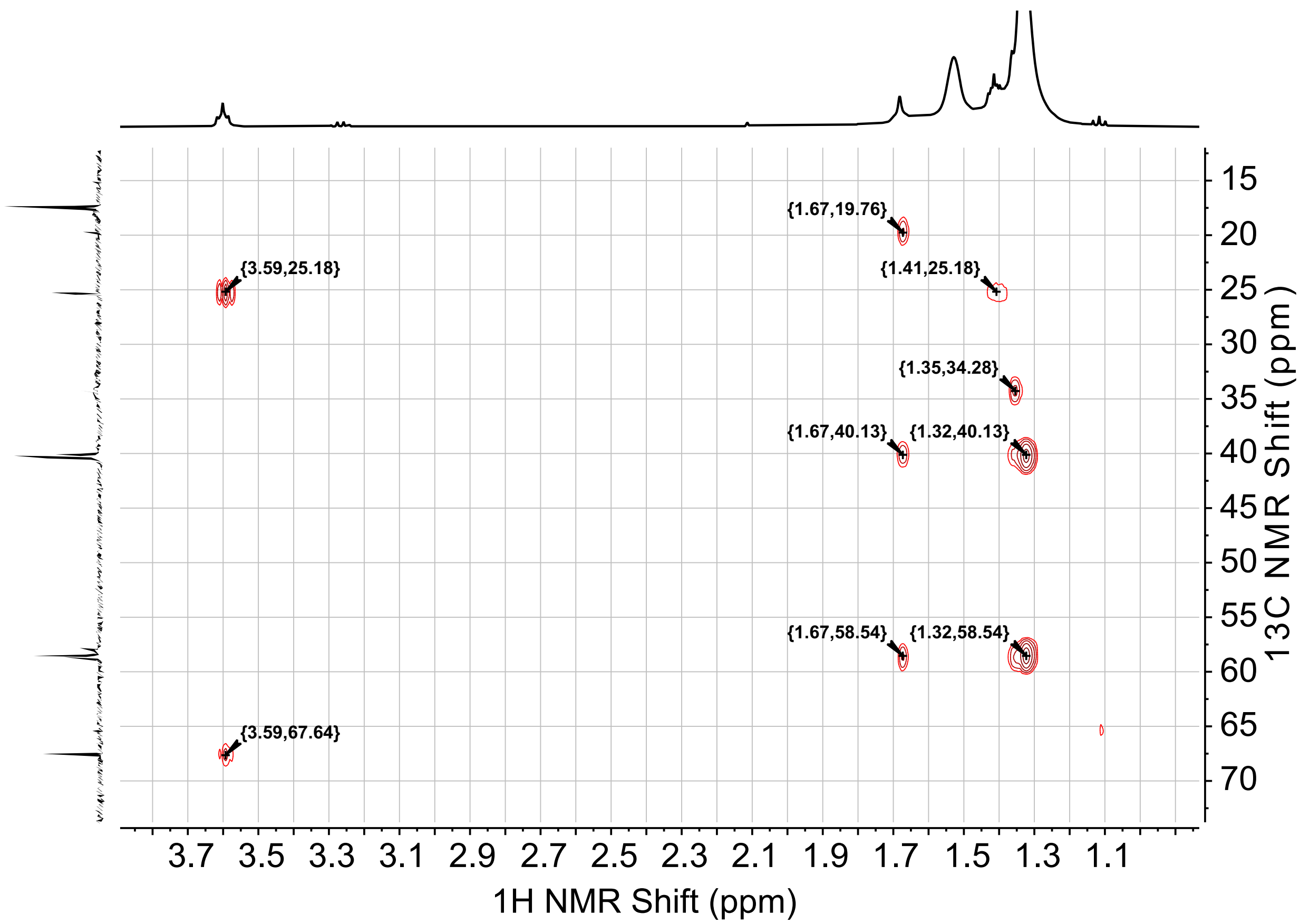

Figure S17. ^{1}H, ^{13}C-HMBC NMR (400 MHz, benzene-d_6) spectrum of **$[Sr{\cdot}Na]_2$**.

Table S3. ICP-MS data of crystalline **[Sr·Na]$_2$**. The expected molar ratio of the sample is 1:1 sodium to strontium. The observed molar ratio of the sample is 1.0000:0.94503 sodium to strontium. The elemental analysis by EDS suggests a molar ratio of 1:1.8 sodium to strontium and showed 2.5% bromide (Fig. S38 and Table S9). ICP-MS analysis for a heteroleptic alkaline earth metal aminoxide complex has been previously used to confirm the metal ratio present in a sample.[2]

	**Sodium Concentration (µg element/mg sample)**	**Strontium Concentration (µg element/mg sample)**
Experimental **[Sr·Na]$_2$**	33.066	119.095
Experimental standard deviation	0.528	0.781
Expected **[Sr·Na]$_2$**	34.4	131.2

2.5 Synthesis and Characterization of $[Zn(TEMPO)_2]_2$ ([Zn])

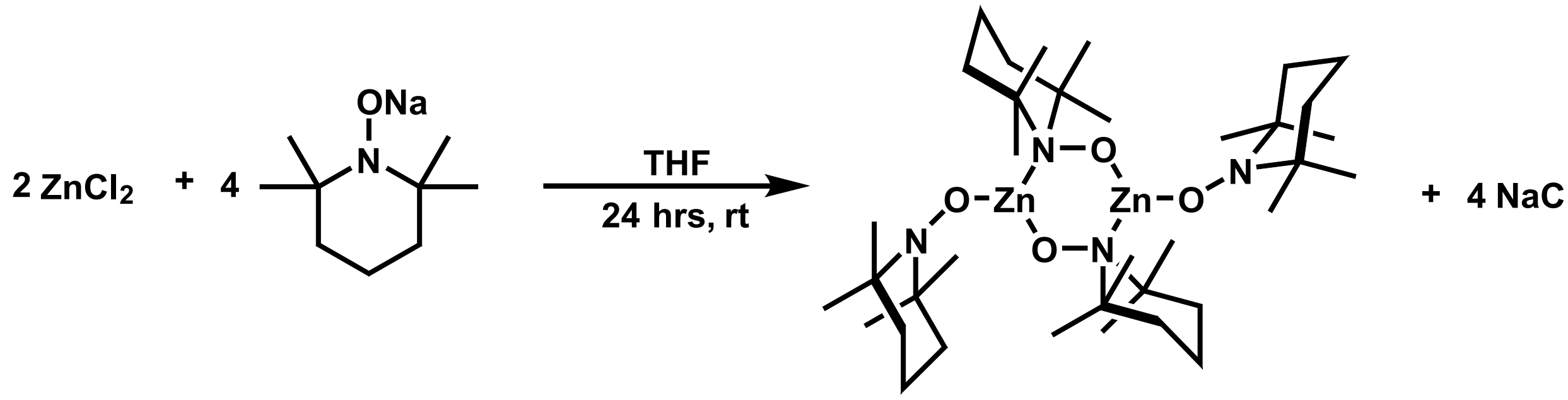

THF (100 mL) was added to NaTEMPO (0.550 g, 3.07 mmol, 1.74 equiv.) and $ZnCl_2$ (0.210 g, 1.76 mmol, 1.00 equiv.). After stirring for ca. 24 hours, the suspension was passed through a 30 mL fine sintered frit containing a half-inch plug of Celite. Volatile materials were removed from the collected filtrate under dynamic vacuum to yield **[Zn]** (0.464 g, 80%). The compound can be crystallized by dissolving the solids in ca. 5 mL of diethyl ether at room temperature and storing the solution at −35 °C, providing **[Zn]** as a colorless crystalline solid. The spectroscopic data of the compound matched the previous literature report.[3]

2.6 Synthesis and Characterization of $Sn(TEMPO)_2{\cdot}Py$ ([Sn])

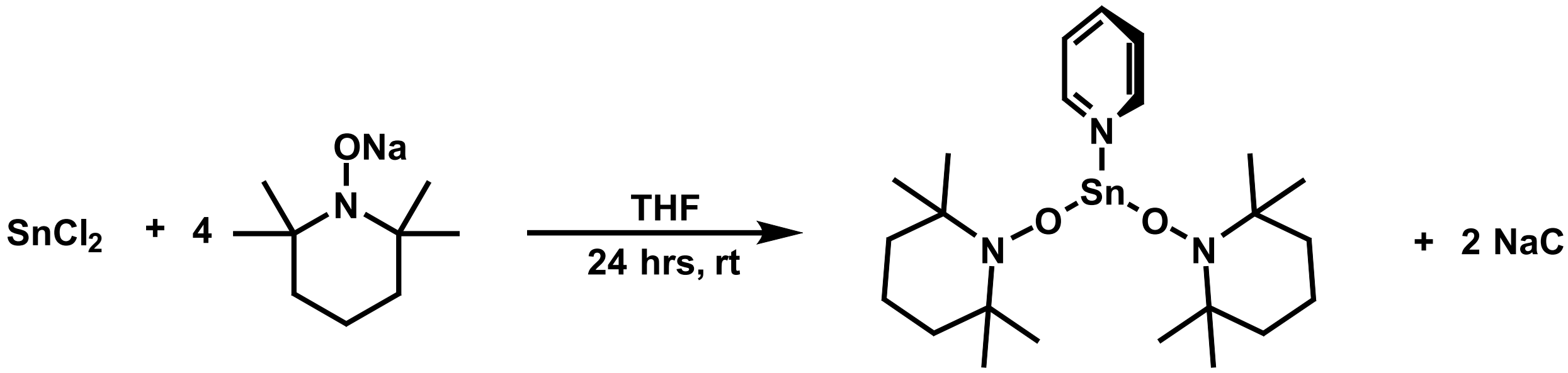

A solution of NaTEMPO (1.890 g, 10.5 mmol, 3.98 equiv.) in pyridine (10 mL) was added to a stirring solution of $SnCl_2$ (0.500 g, 2.64 mmol, 1.00 equiv.) in pyridine (10 mL). After stirring for ca. 24 hours, the suspension was passed through a 15 mL fine sintered frit containing a half-inch plug of Celite. Volatile materials were removed from the collected filtrate under dynamic vacuum. The solids were dissolved in ca. 5 mL of toluene at room temperature and stored at −35 °C, providing **[Sn]** as a colorless crystalline solid (1.150 g, 85%) with crystals suitable for SCXRD. ^{1}H NMR (400 MHz, benzene-d_6, Fig. S18) δ 8.54, 6.94, 6.63, 2.11, 1.61, 1.25 ppm. To collect a quantitative ^{1}H NMR spectrum of **[Sn]** (Fig. S21), a stock solution of 1,3,5-trimethoxybenzene (0.0084 g, 0.050 mmol) in benzene-d_6 (1.00 mL) prepared. From the 1,3,5-trimethoxybenzene stock solution, 0.218 mL was added to solid **[Sn]** (0.025 g) and the solution was transferred to an NMR tube. Additional benzene-d_6 (0.40 mL) was added to the NMR tube and data was collected with relaxation delay (D1) time of 40 s. The quantitative ^{1}H NMR (500 MHz, benzene-d_6, Fig. S21) spectrum of **[Sn]** confirms 95% purity of the crystallized compound. ^{13}C{^{1}H} NMR (101 MHz, benzene-d_6, Fig. S19) δ 58.9, 41.4, 28.8 (br), 18.2 ppm. ICP-MS data is shown in Table S4.

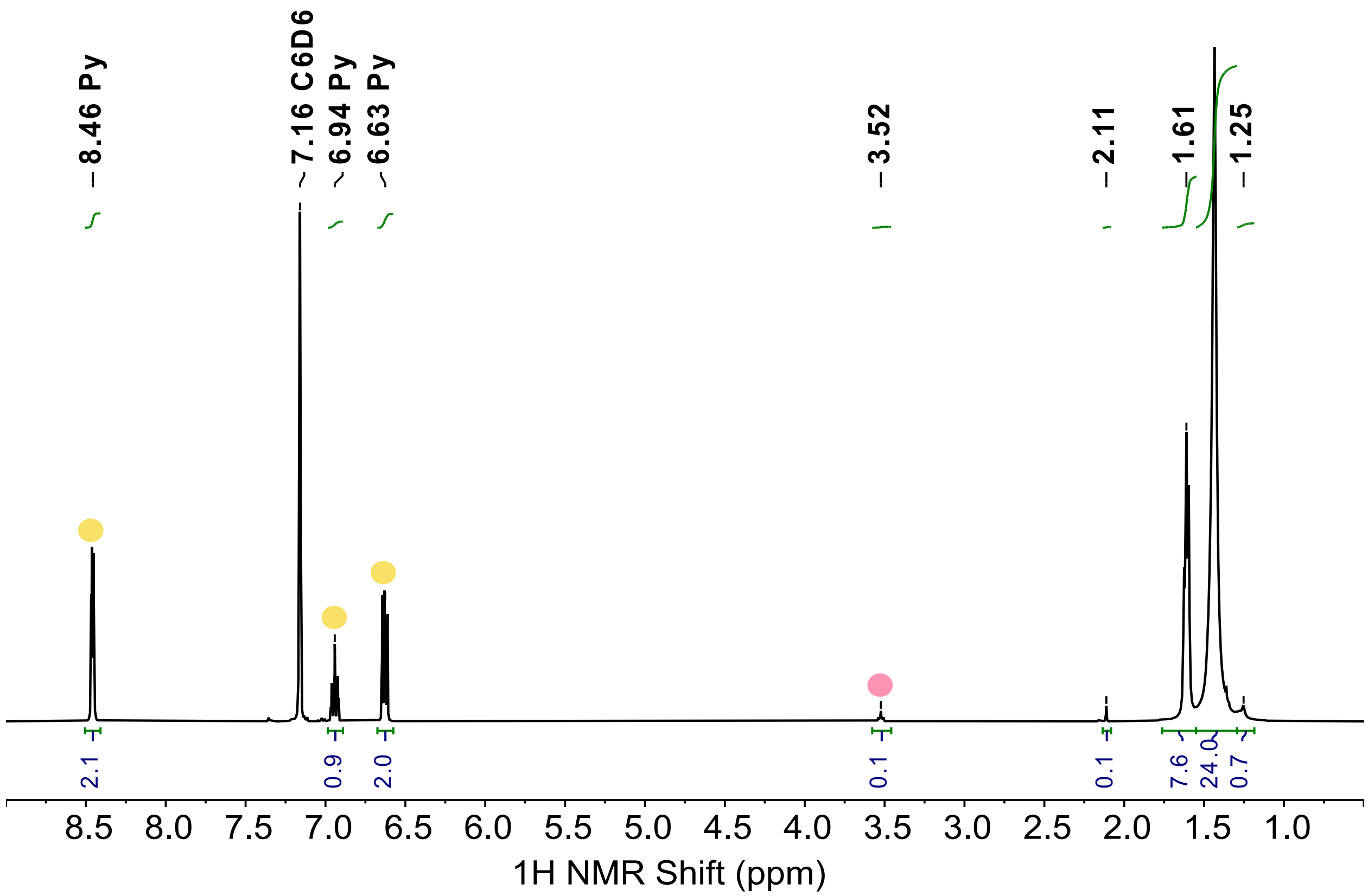

Figure S18. ^{1}H NMR (400 MHz, benzene-d_6, 25 °C) spectrum of **[Sn]**. The yellow dots indicate resonances assigned to pyridine coordinated to the Sn center, the pink dot indicates a resonance assigned to trace THF.

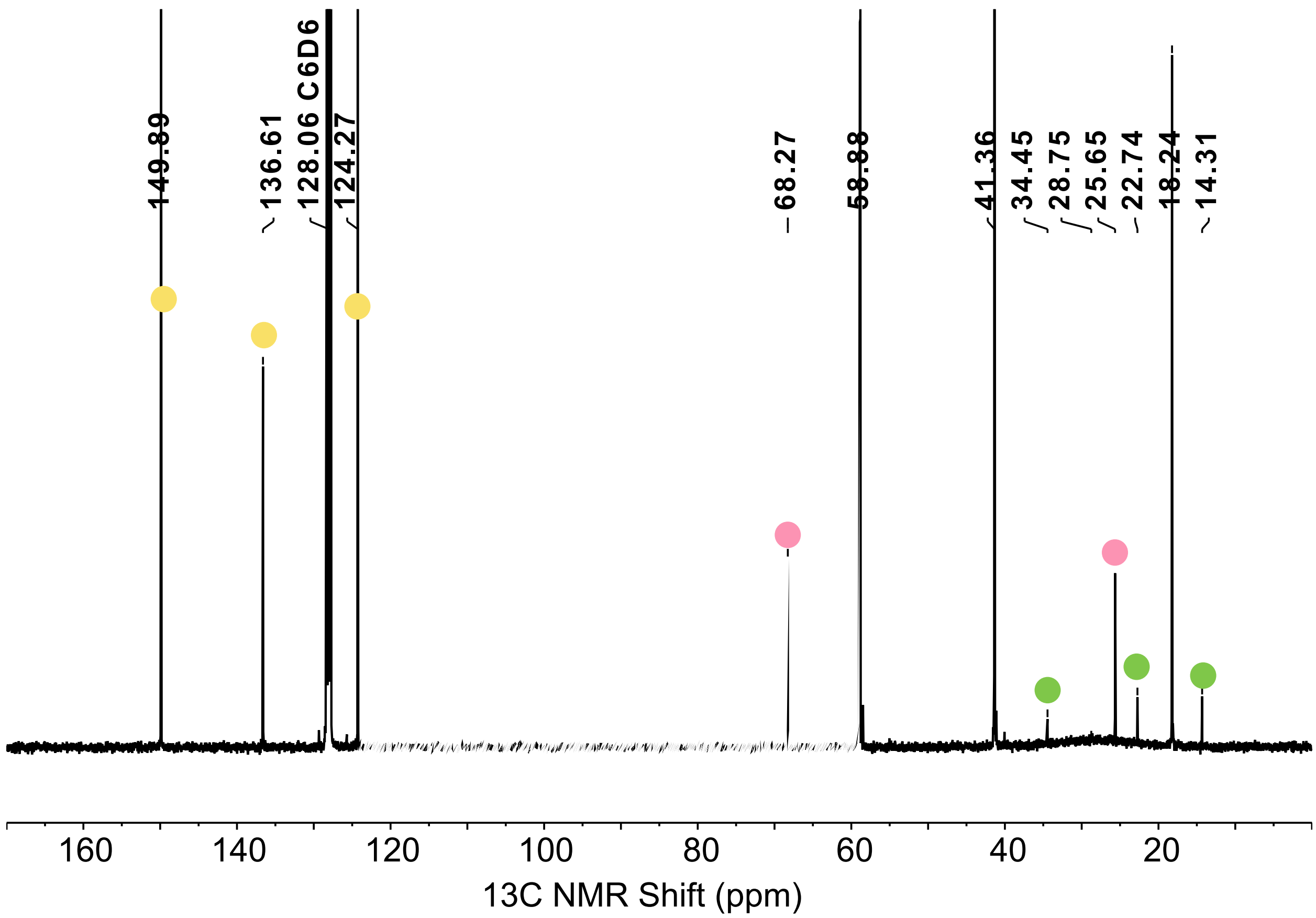

Figure S19. $^{13}C\{^{1}H\}$ NMR (101 MHz, benzene-d_6) spectrum of **[Sn]**. The yellow dots indicate resonances assigned to pyridine coordinated to the Sn center, the pink dots indicate resonances assigned to trace THF, the green dots indicate resonances assigned to *n*-hexane which was present in the solvent.

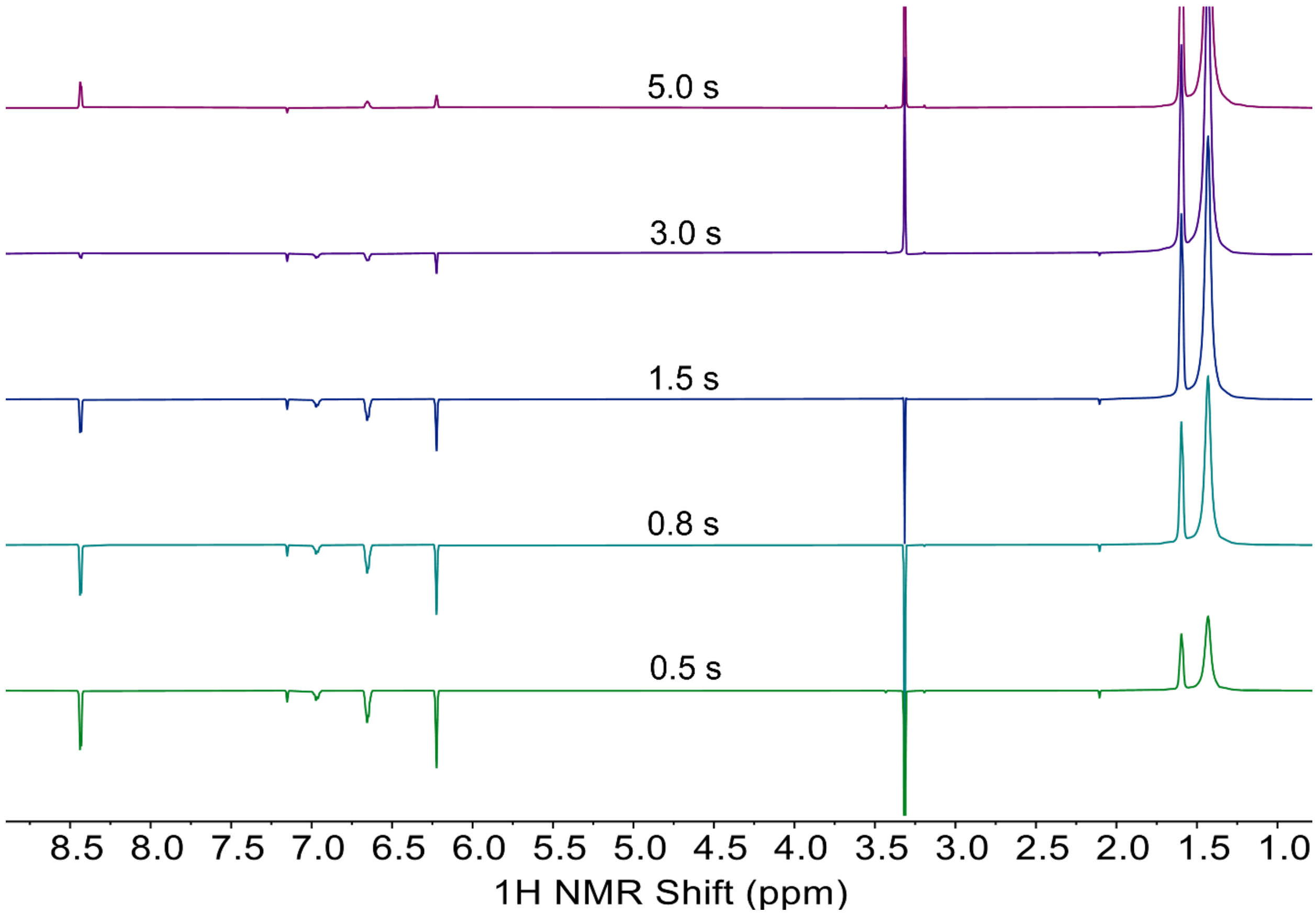

Figure S20. Stacked ¹H NMR (500 MHz, benzene-d_6, 25 °C) spectra **[Sn]** and 1,3,5-trimethoxybenzene and associated relaxation delay times (D1) from an inversion recovery experiment.

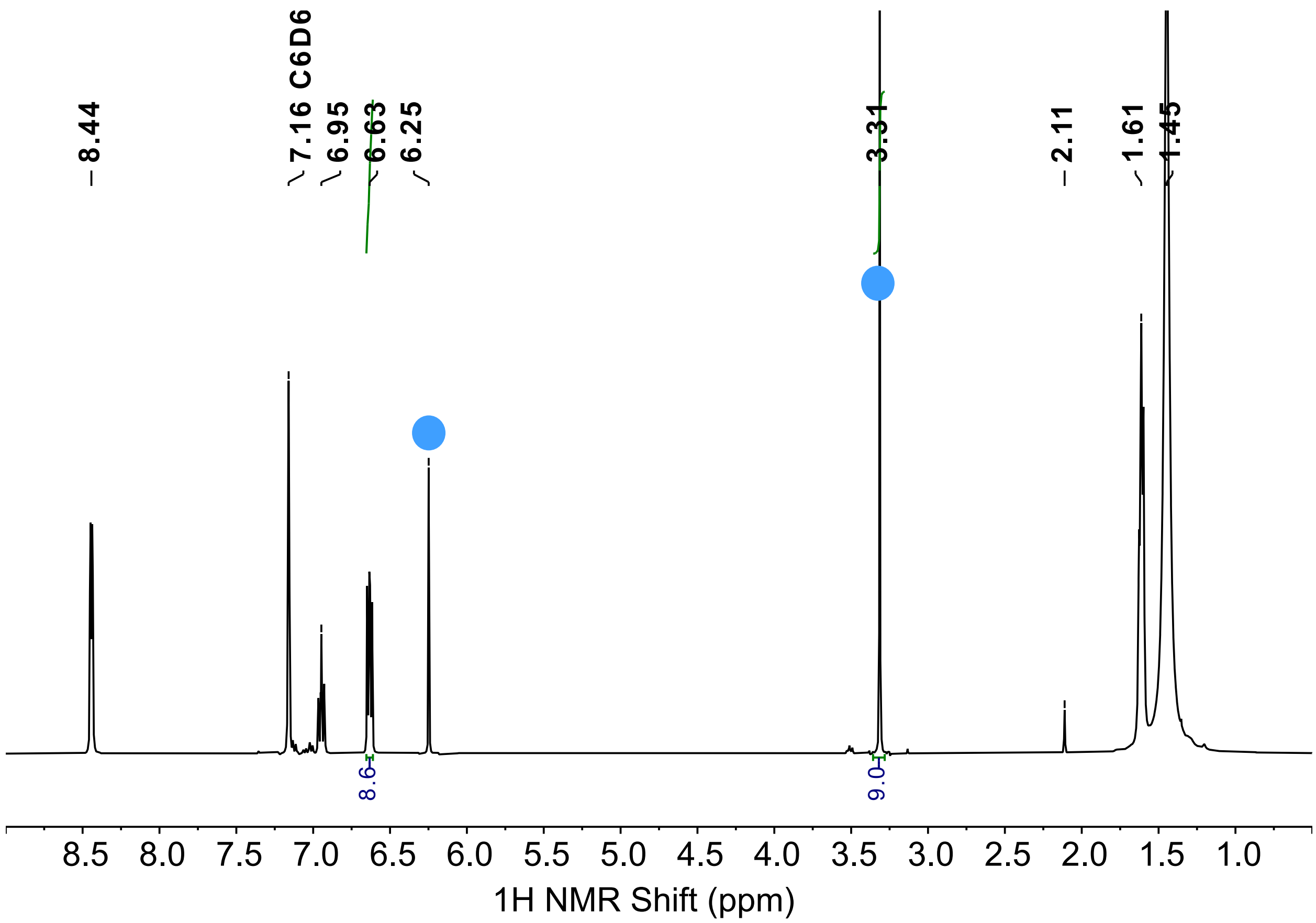

Figure S21. Quantitative ^{1}H NMR (500 MHz, benzene-d_6) spectrum of **[Sn]**, 1,3,5-trimethoxybenzene, and toluene which was present in the NMR solvent. The blue dots indicate resonances assigned to 1,3,5-trimethoxybenzene.

Table S4. ICP-MS data of crystalline **[Sn]**. The expected molar ratio of the sample is 0:1 sodium to tin. The observed molar ratio of the sample is 1.00:0.00393 sodium to tin. ICP-MS analysis for a heteroleptic alkaline earth metal aminoxide complex has been previously used to confirm the metal ratio present in a sample.[2]

	**Sodium Concentration (µg element/mg sample)**	**Tin Concentration (µg element/mg sample)**
Experimental **[Sn·Py]**	27.976	0.567
Experimental standard deviation	0.006	1.413
Expected **[Sn·Py]**	0	232.6

2.7 Synthesis and Characterization of $[Cd(TEMPO)_2]_2 \cdot 2THF$ ([Cd])

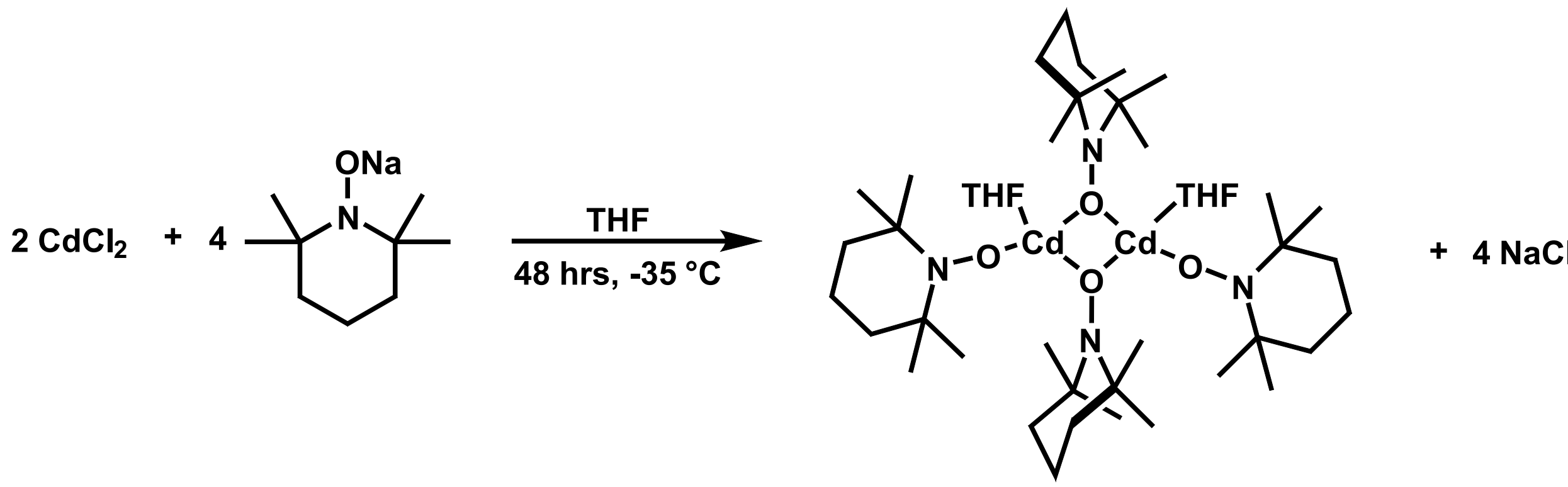

To a −35 °C stirring suspension of $CdCl_2$ (0.324g, 1.77 mmol, 2.00 equiv.) in THF (4.5 mL) was added a −35 °C solution of NaTEMPO (0.634 g, 3.54 mmol, 1.00 equiv.) in THF (4.5 mL). After stirring for ca. 48 hours at −35 °C, the suspension was passed through a 15 mL medium sintered frit (pre-cooled at −35 °C to avoid the formation of cadmium metal) containing a half-inch plug of Celite. Volatile materials were removed from the collected filtrate under dynamic vacuum. The resulting solids were suspended in pentanes (5 mL) and filtered through a 15 mL medium sintered frit. Volatile materials were removed from the collected filtrate under dynamic vacuum to yield **[Cd]** as a bright yellow solid (0.673 g, 77%). The compound can be crystallized by dissolving the solids in ca. 3 mL of hexanes at room temperature and storing the solution at −35 °C, providing **[Cd]** as a yellow crystalline solid. The ^{1}H NMR spectrum of **[Cd]** in benzene-d_6 (Fig. S22) exhibits a broad, complicated feature from approximately 2.25 ppm to 1.00 ppm, however, upon removing the benzene-d_6 under dynamic vacuum and redissolving the material in pyridine-d_5, the ^{1}H NMR spectrum (Fig. S23) exhibits sharp resonances. This behavior is indicative of fluxionality of the ligands on the NMR timescale. ^{1}H NMR (600 MHz, benzene-d_6, Fig. S22) δ 4.85, 4.78, 3.58, 1.98, 1.64, 1.44, 1.19, 1.07 ppm. ^{1}H NMR (600 MHz, pyridine-d_5, Fig. S23) δ 1.64, 1.51, 1.34, 1.21, 1.11, 0.82 ppm. ^{13}C{^{1}H} NMR (151 MHz, benzene-d_6, Fig. S24) δ 67.9, 61.1, 59.2 (br), 40.6, 40.0, 35.0 (br), 26.7, 25.8, 23.3, 18.3 (br), 17.4 (br) ppm. ICP-MS data is shown in Table S5. It is important to note that the addition of NaTEMPO solution in THF to the stirring suspension of $CdCl_2$ in THF is performed at −35 °C to avoid the formation of solids identified as cadmium metal and NaCl by PXRD and the release of TEMPO radical assessed by UV-Vis absorbance spectroscopy (Fig. S27).[4,5]

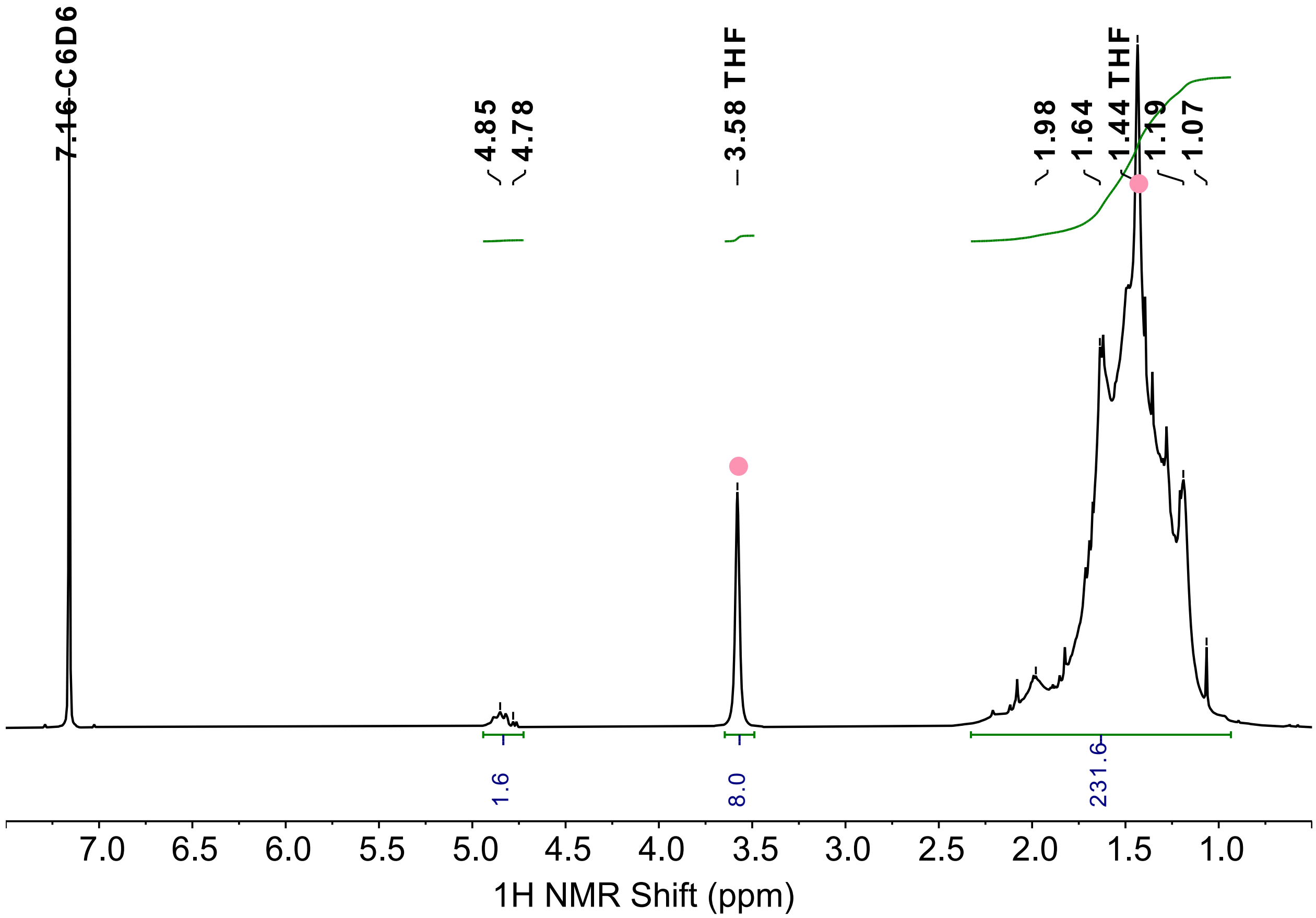

Figure S22. ^{1}H NMR (600 MHz, benzene-d_6, 25 °C) spectrum of **[Cd]**. The pink dots indicate resonances assigned to free THF.

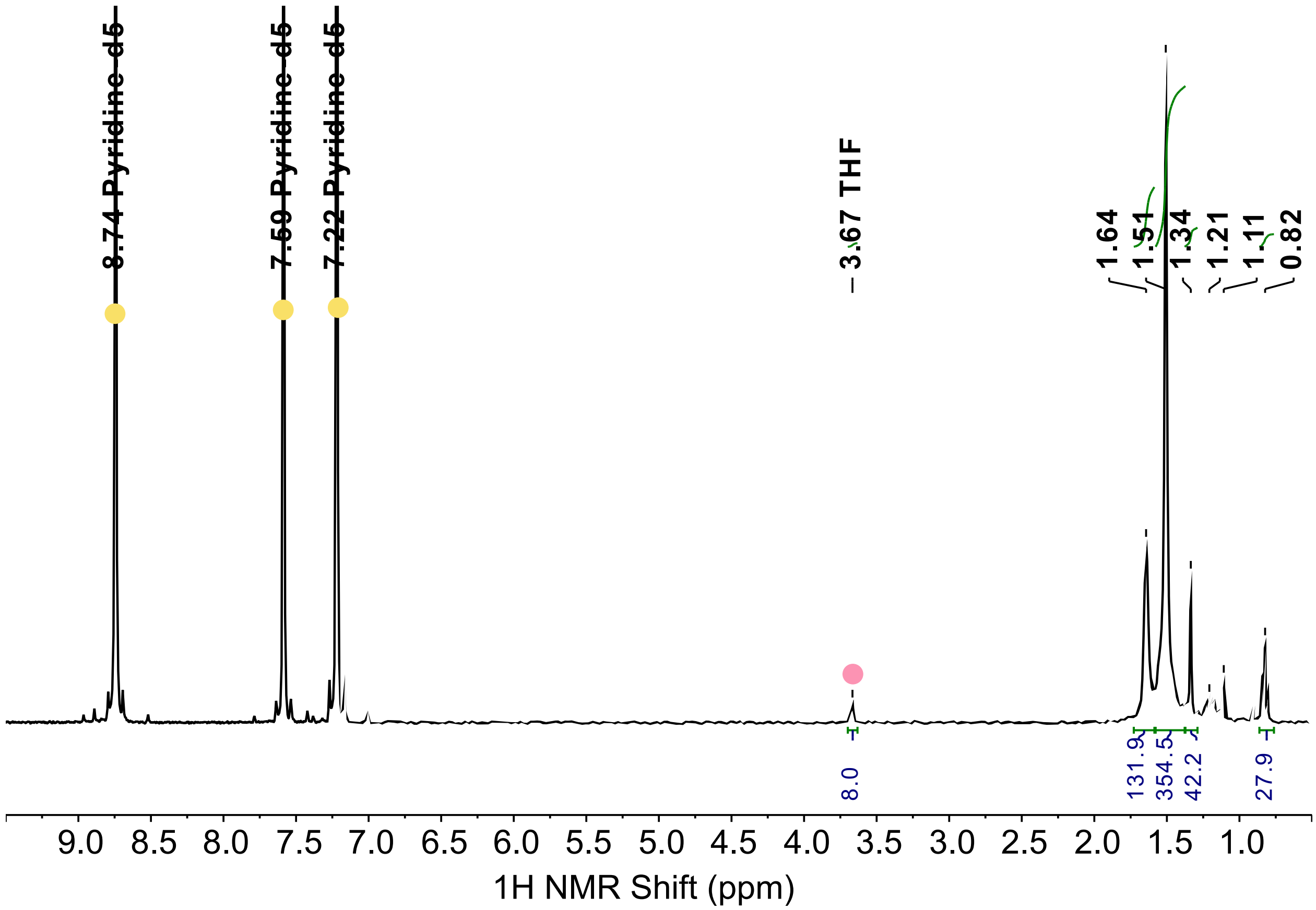

Figure S23. ^{1}H NMR (600 MHz, pyridine-d_5) spectrum of **[Cd]**. The yellow dots indicate resonances assigned to pyridine, the pink dots indicate resonances assigned to free THF.

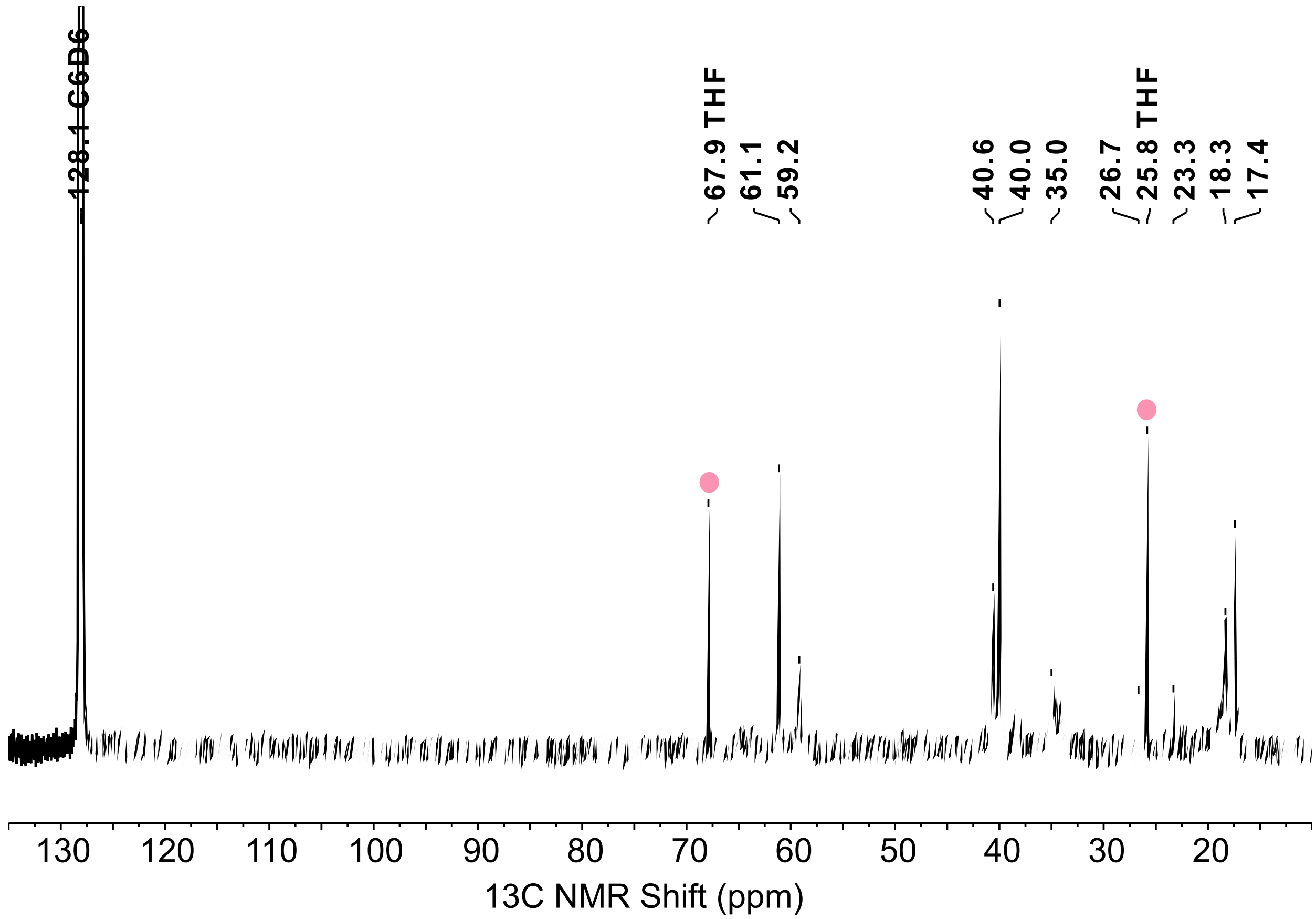

Figure S24. $^{13}C\{^{1}H\}$ NMR (101 MHz, benzene-d_6) spectrum of **[Cd]**. The pink dots indicate resonances assigned to free THF.

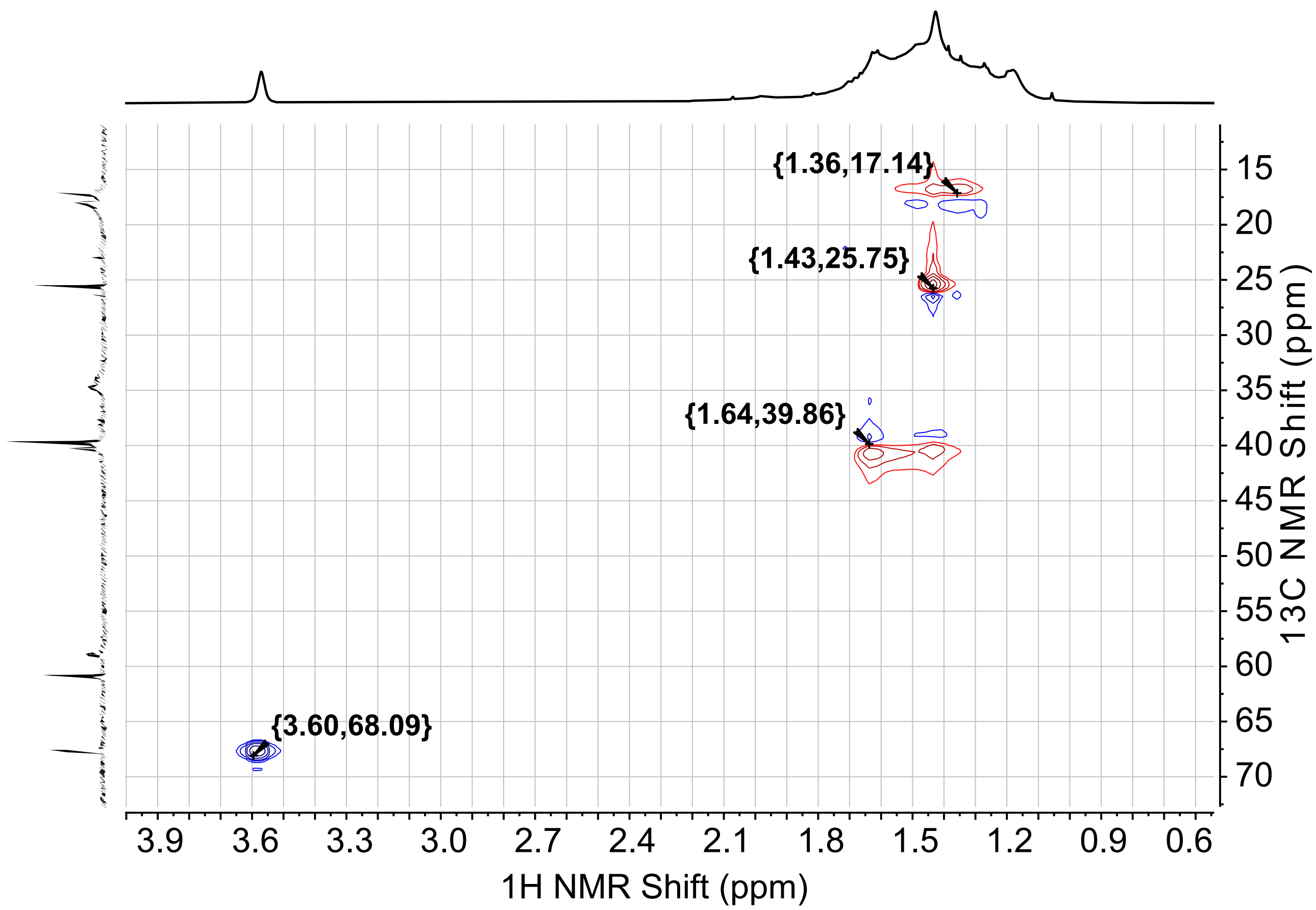

Figure S25. Multiplicity-edited ^{1}H, ^{13}C-HSQC NMR (400 MHz, benzene-d_6) spectrum of **[Cd]**.

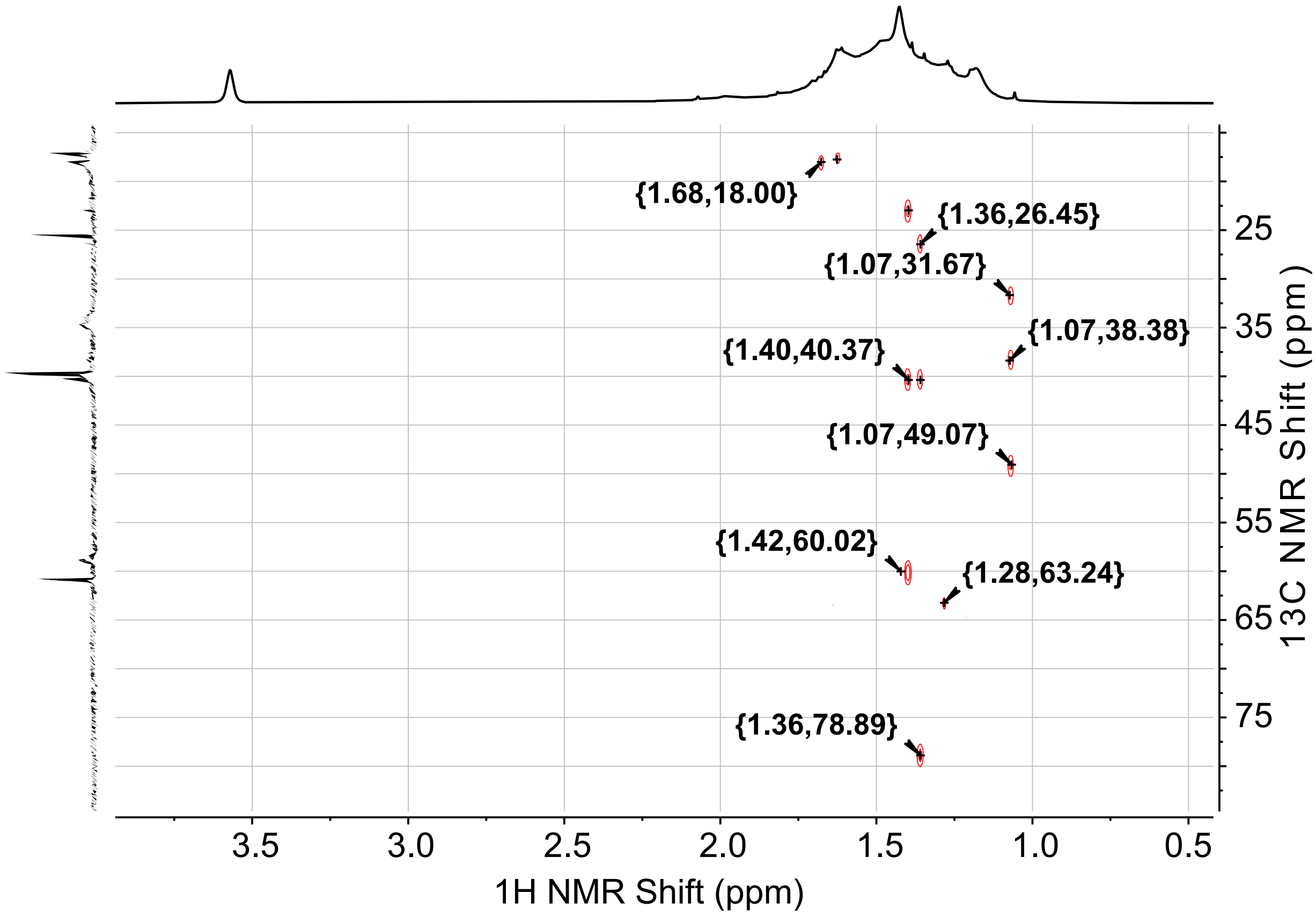

Figure S26. ^{1}H, ^{13}C-HMBC NMR (400 MHz, benzene-d_6) spectrum of **[Cd]**.

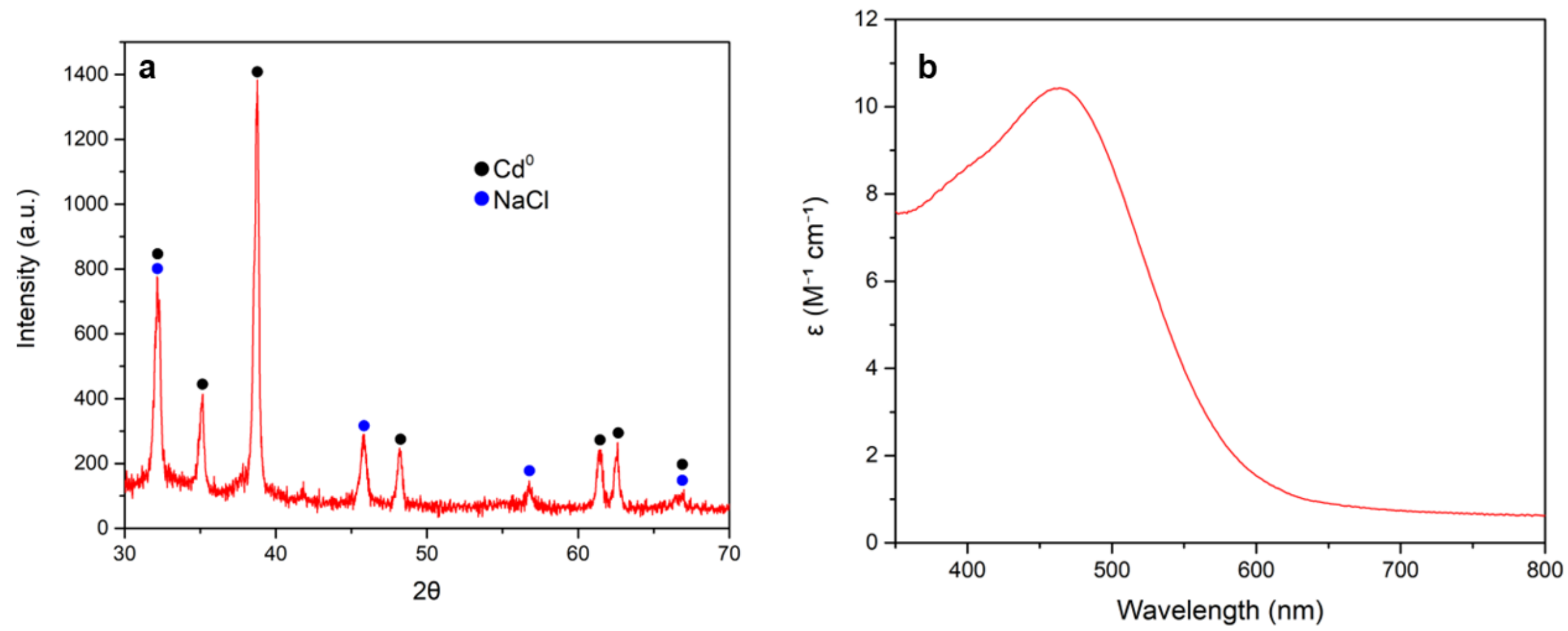

Figure S27. (a) PXRD pattern of solids obtained by treating $CdCl_2$ with NaTEMPO at room temperature (0.5° slit, 0.017° step size, and 40 s/step) and (b) UV-Vis absorbance spectra of the reaction mixture containing TEMPO at ~470 nm.

Table S5. ICP-MS data of crystalline **[Cd]**. The expected molar ratio of the sample is 0:1 sodium to cadmium. The observed molar ratio of the sample is 0.05029:1.000 sodium to cadmium. ICP-MS analysis for a heteroleptic alkaline earth metal aminoxide complex has been previously used to confirm the metal ratio present in a sample.[2]

	**Sodium Concentration (μg element/mg sample)**	**Cadmium Concentration (μg element/mg sample)**
Experimental **[Cd]**	2.716	264.051
Experimental standard deviation	0.019	2.854
Expected **[Cd]**	0	226.6

2.8 Synthesis of [Pb(TEMPO)$_2$]$_2$ ([Pb])

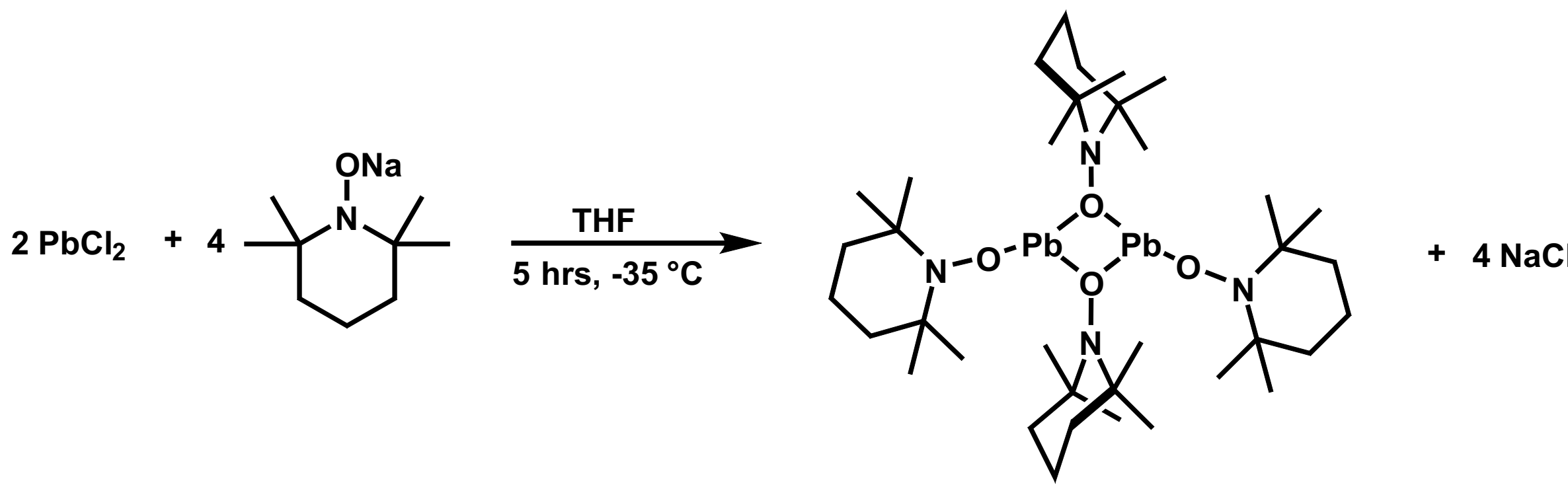

To a −35 °C stirring suspension of $PbCl_2$ (0.500 g, 1.80 mmol, 2.00 equiv.) in THF (6.0 mL) was added a −35 °C solution of NaTEMPO (0.645 g, 3.60 mmol, 1.00 equiv.) in THF (6.0 mL). After stirring for ca. 5 hours at −35 °C, the suspension was passed through a 15 mL medium sintered frit (pre-cooled at −35 °C to avoid the formation of lead metal and lead oxides) containing a half-inch plug of Celite. Volatile materials were removed from the collected filtrate under dynamic vacuum. The resulting solids were suspended in pentanes (5 mL), and the suspension was passed through a 15 mL medium sintered frit. The filtrate was concentrated to ca. 3 mL under reduced pressure and placed in a –35 °C freezer for crystallization providing **[Pb]** as a yellow crystalline solid with crystals suitable for SCXRD. NMR spectroscopic data and UV-vis absorbance data of the compound could not be obtained due to its thermal instability in benzene-d_6 and THF. ICP-MS data of **[Pb]** was collected and the data is shown in Table S6. It is important to note that the addition of NaTEMPO solution in THF to stirring suspension of $PbCl_2$ in THF is performed at −35 °C to avoid the formation of solids identified as lead metal, NaCl, PbO_2, and unreacted $PbCl_2$ by PXRD and the release of TEMPO radical as assessed by UV-Vis absorbance spectroscopy (Fig. S28).[5–8]

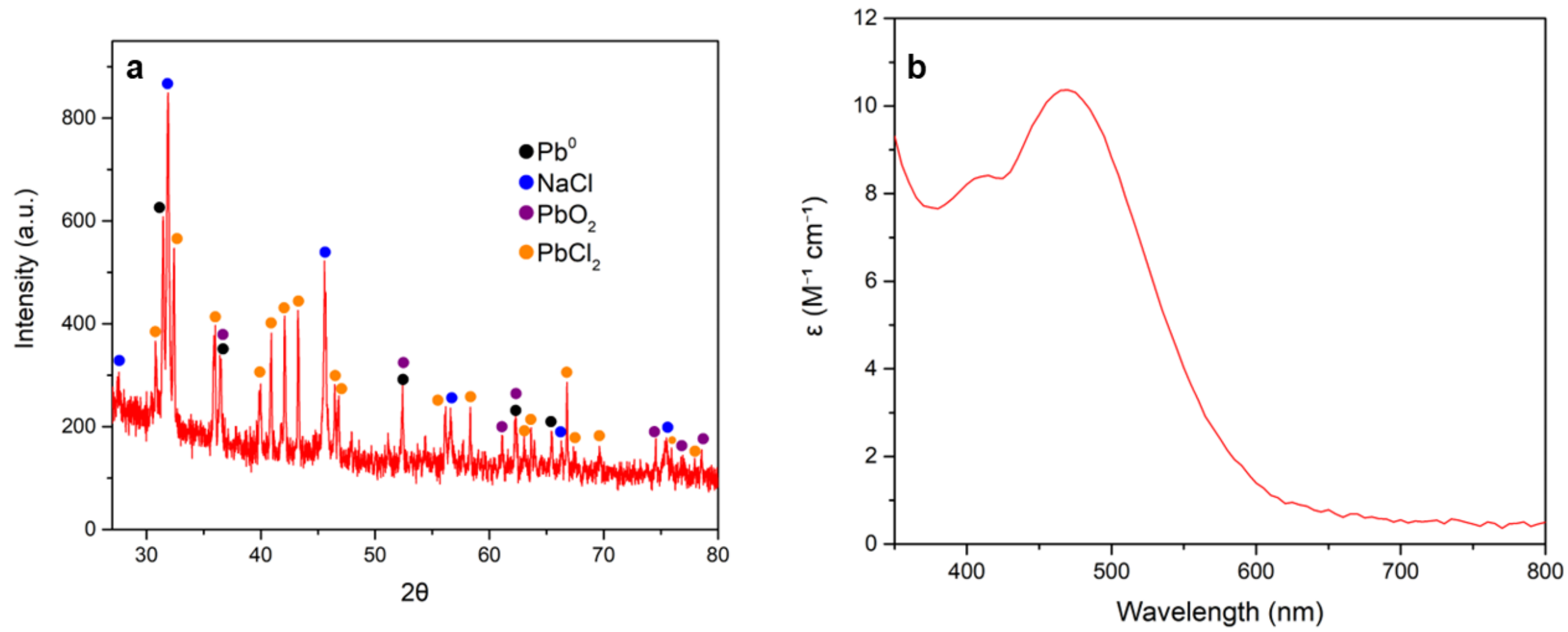

Figure S28. (a) PXRD pattern of solids obtained by treating $PbCl_2$ with NaTEMPO at room temperature (0.5° slit, 0.017° step size, and 40 s/step) and (b) UV-Vis absorbance spectra of the reaction mixture in which TEMPO can be observed at ~470 nm.

Table S6. ICP-MS data of crystalline **[Pb]**. The expected molar ratio of the sample is 0:1 sodium to lead. The observed molar ratio of the sample is 0.03481:1.000 sodium to lead. ICP-MS analysis for a heteroleptic alkaline earth metal aminoxide complex has been previously used to confirm the metal ratio present in a sample.[2]

	**Sodium Concentration (µg element/mg sample)**	**Lead Concentration (µg element/mg sample)**
Experimental **[Pb]**	1.613	417.576
Experimental standard deviation	0.012	9.292
Expected **[Pb]**	0	398.7

3. UV-Vis Absorbance Spectra

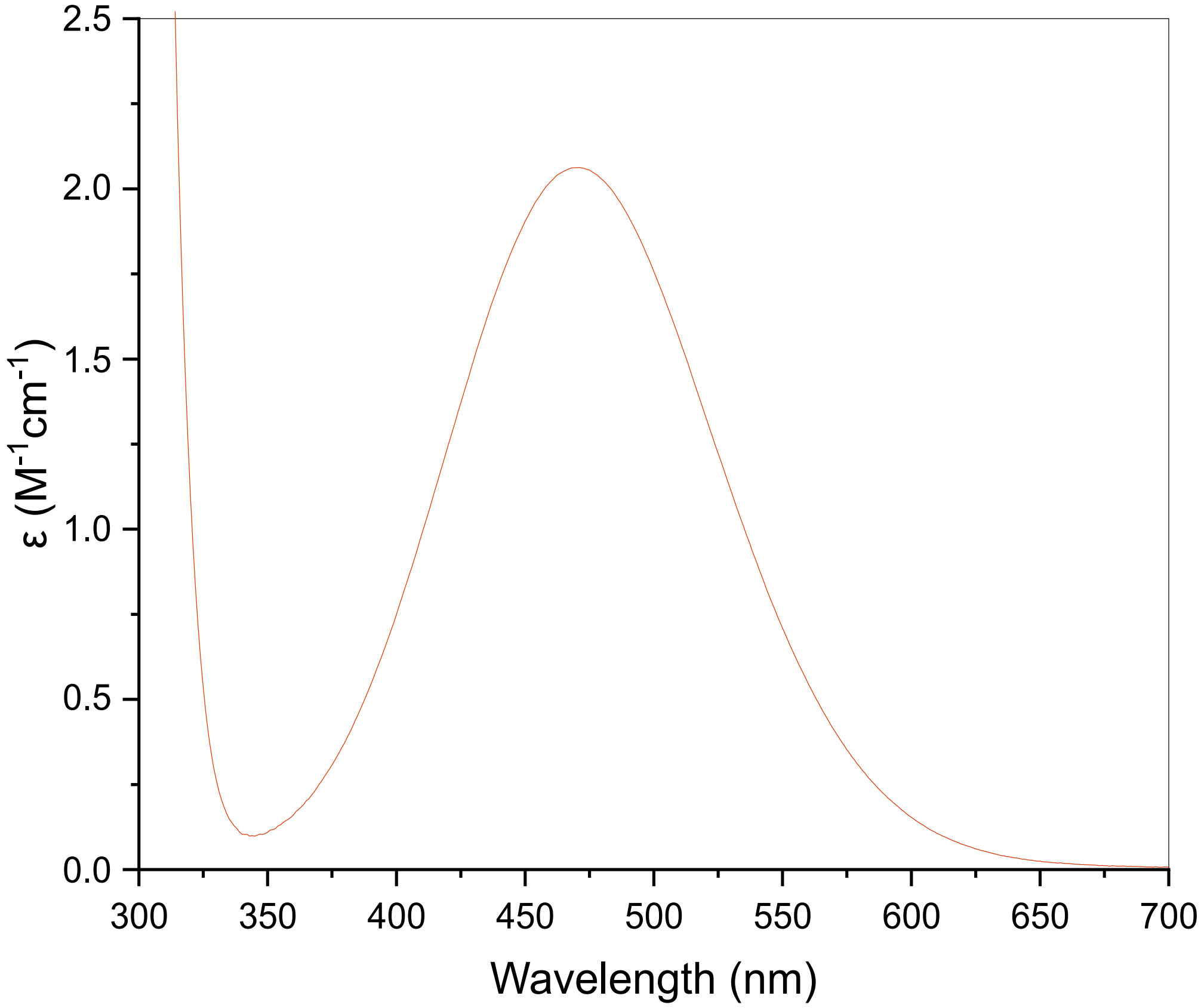

Figure S29. Absorbance spectrum of TEMPO (96.4 mM) obtained at 25 ºC in THF.

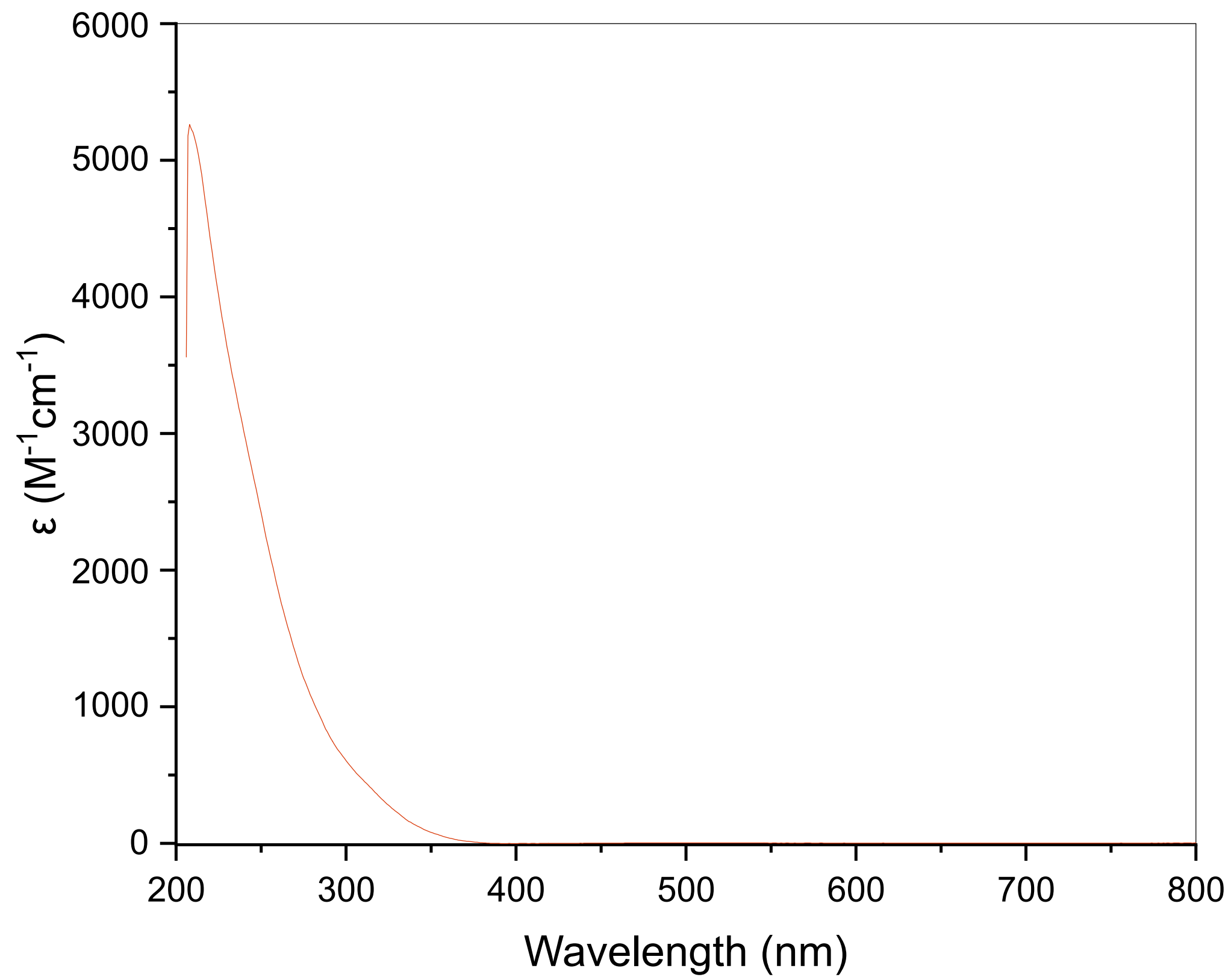

Figure S30. Absorbance spectrum of NaTEMPO (0.383 mM) obtained at 25 ºC in THF.

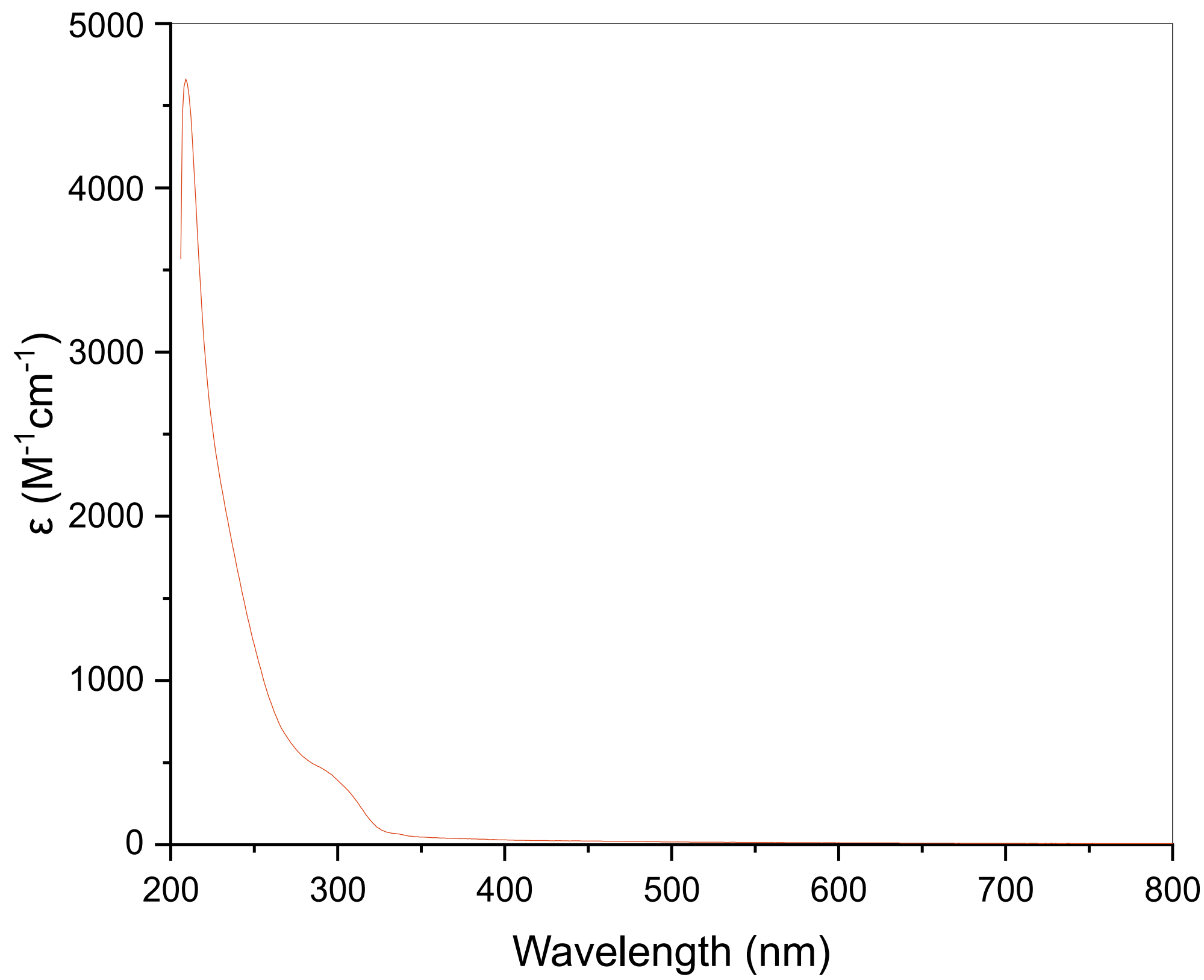

Figure S31. Absorbance spectrum of **[Mg]** (0.312 mM) obtained at 25 ºC in THF.

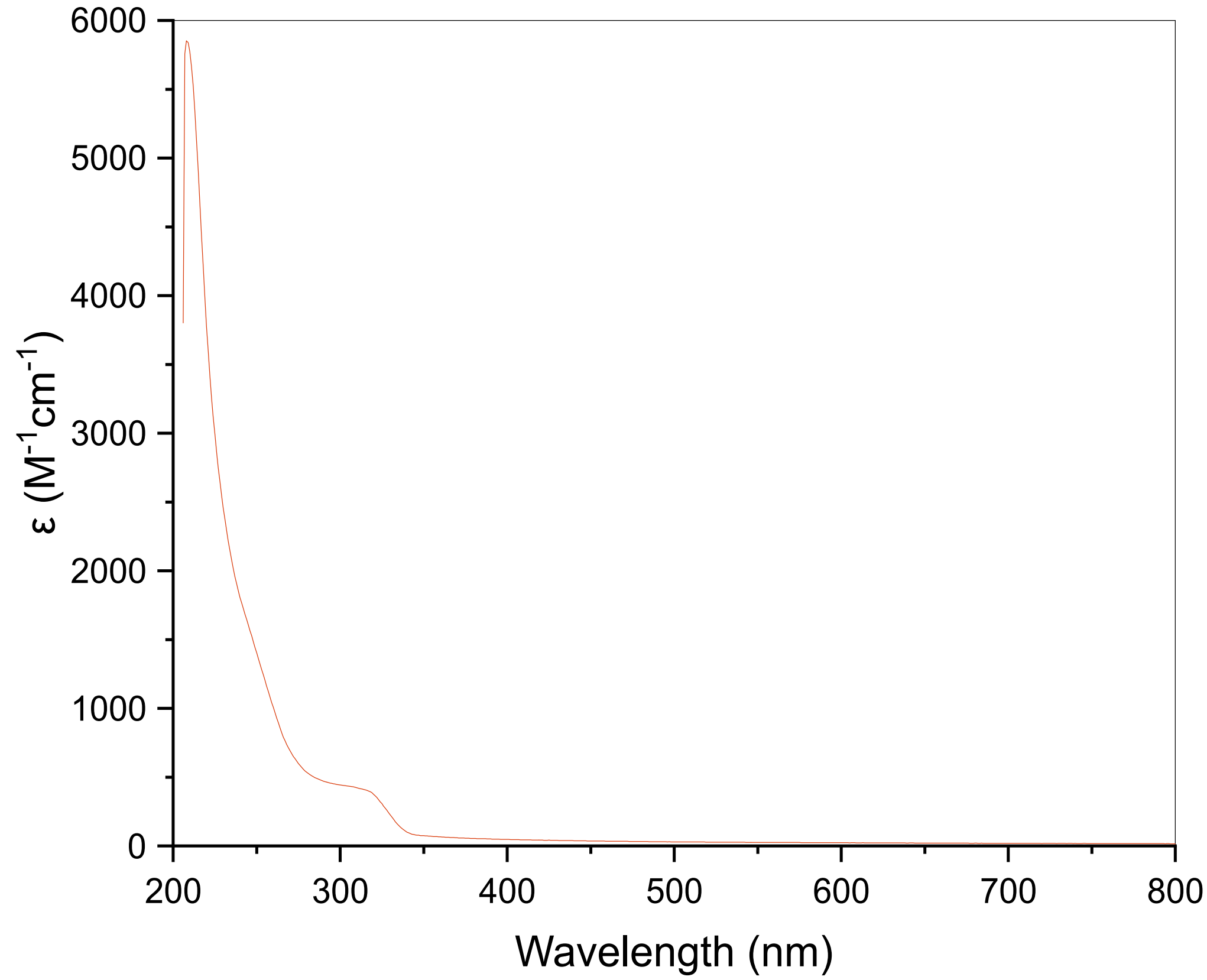

Figure S32. Absorbance spectrum of **[Ca·Na$_2$]** (0.300 mM) obtained at 25 ºC in THF.

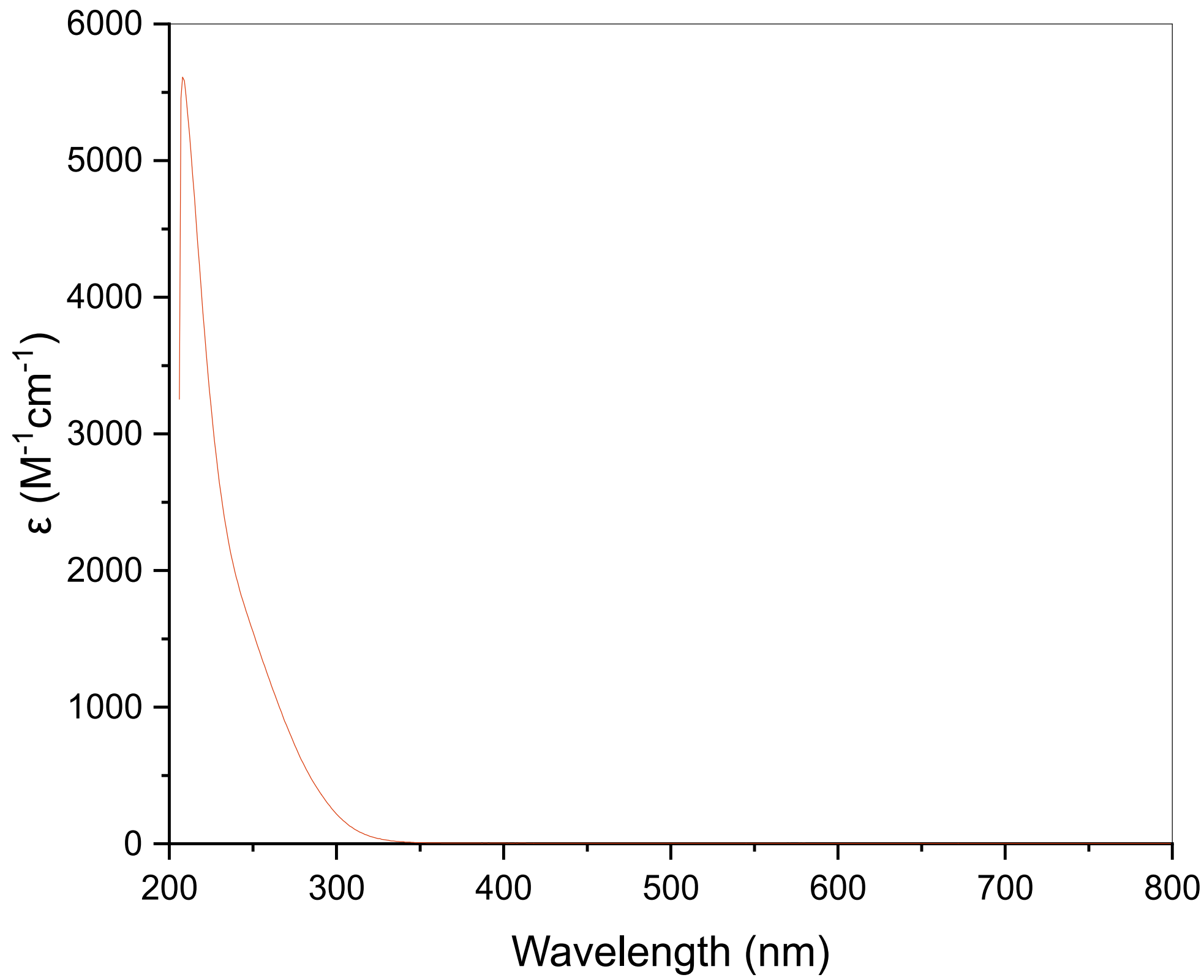

Figure S33. Absorbance spectrum of **[Zn]** (0.300 mM) obtained at 25 ºC in THF.

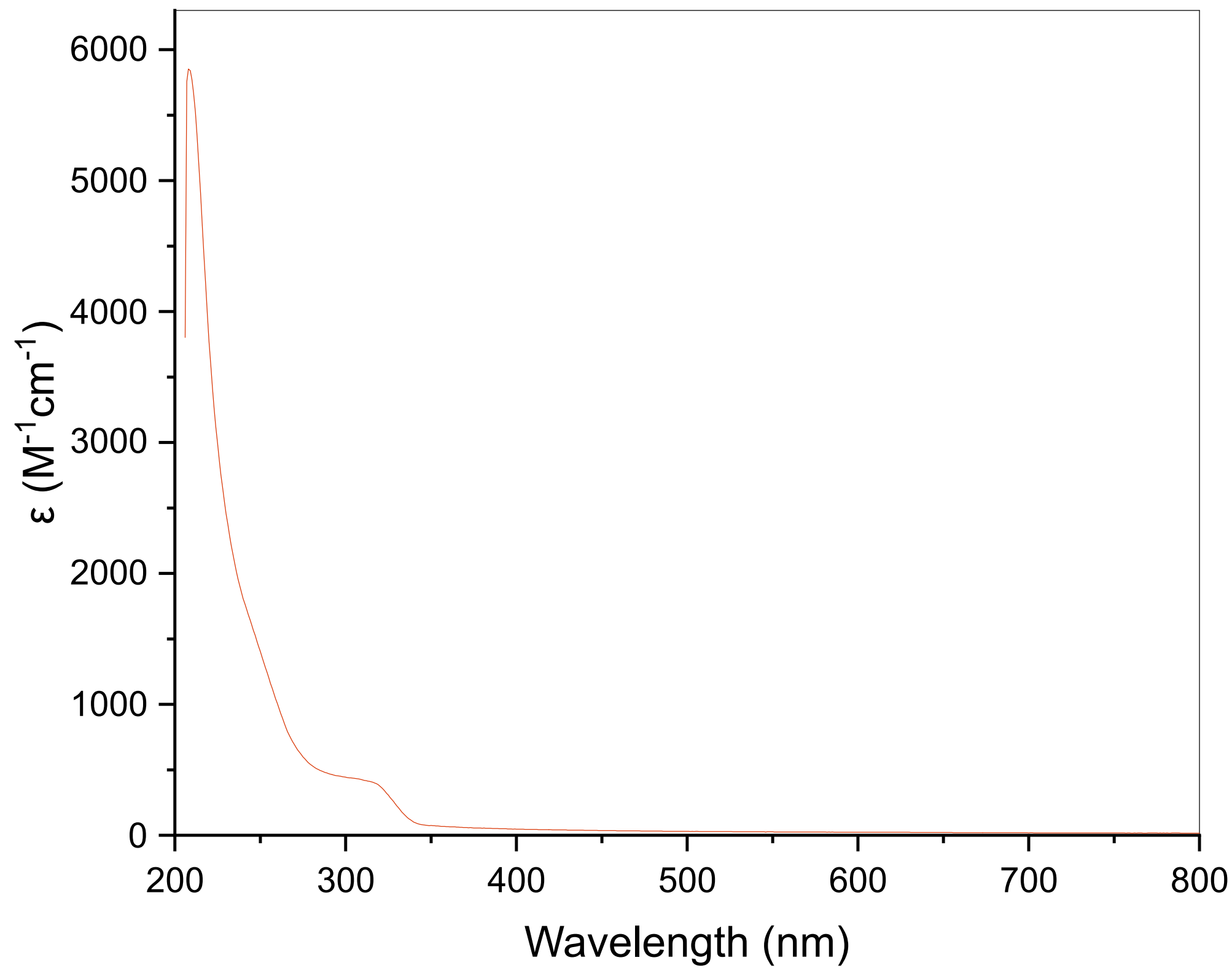

Figure S34. Absorbance spectrum of **[Cd]** (0.757 mM) obtained at 25 ºC in THF.

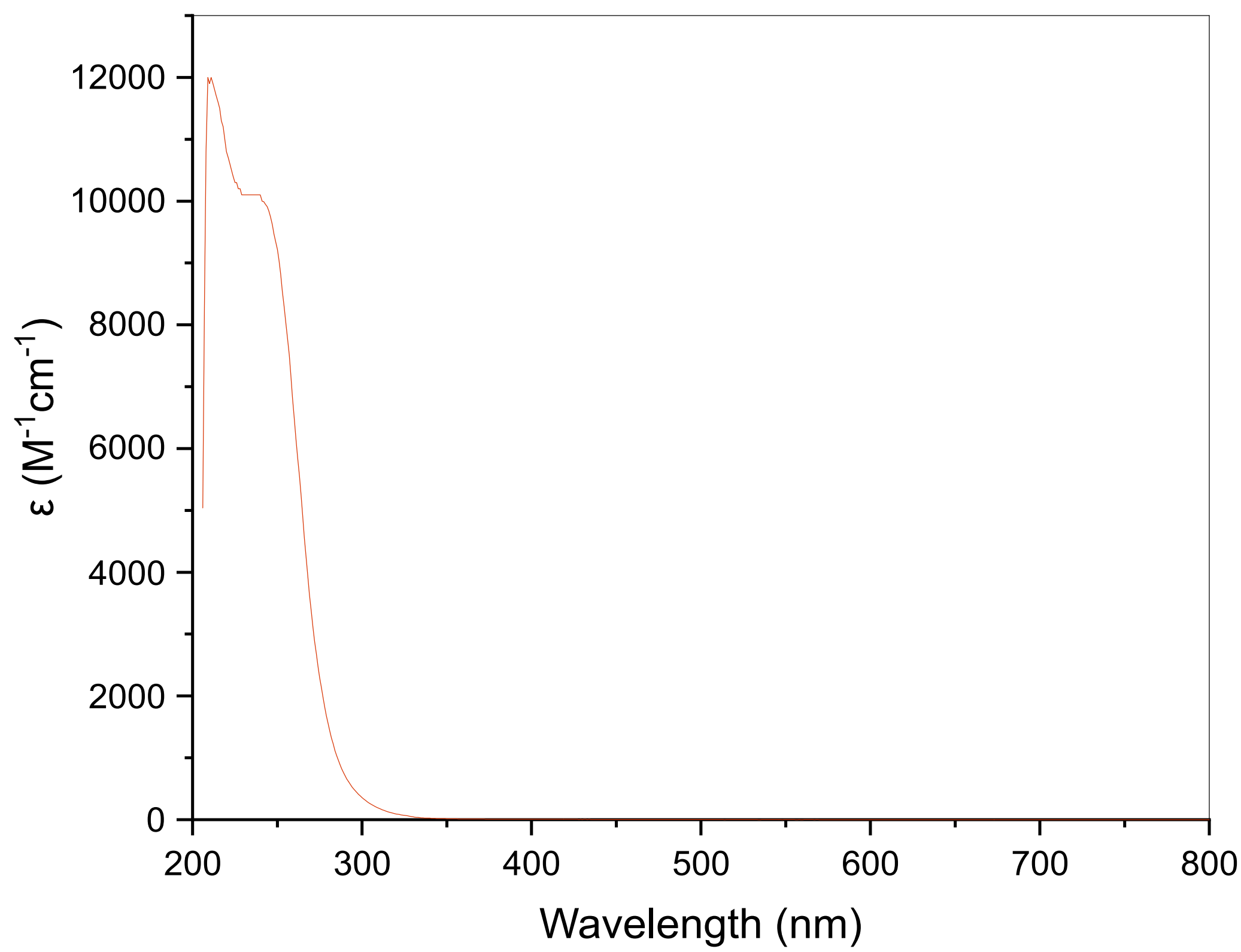

Figure S35. Absorbance spectrum of **[Sn]** (0.300 mM) obtained at 25 ºC in THF.

4. Energy dispersive X-ray spectroscopy (EDS) data

EDS data was obtained by removing a sample from the glovebox and letting it thoroughly react with air for 1 hour. During this time, it is assumed that the compound reacted with atmospheric oxygen and water to form metal oxides and TEMPO. The resulting TEMPO was then expected to sublime from the sample. Accordingly, each sample changed from its original white color to red (indicating TEMPO formation), then changed back to white (indicating TEMPO sublimation from the sample). The sample was then loaded onto carbon tape and analyzed using a scanning electron microscope equipped with EDS. For this reason, the following data contains only metal, oxygen, and carbon signals (from the carbon tape).

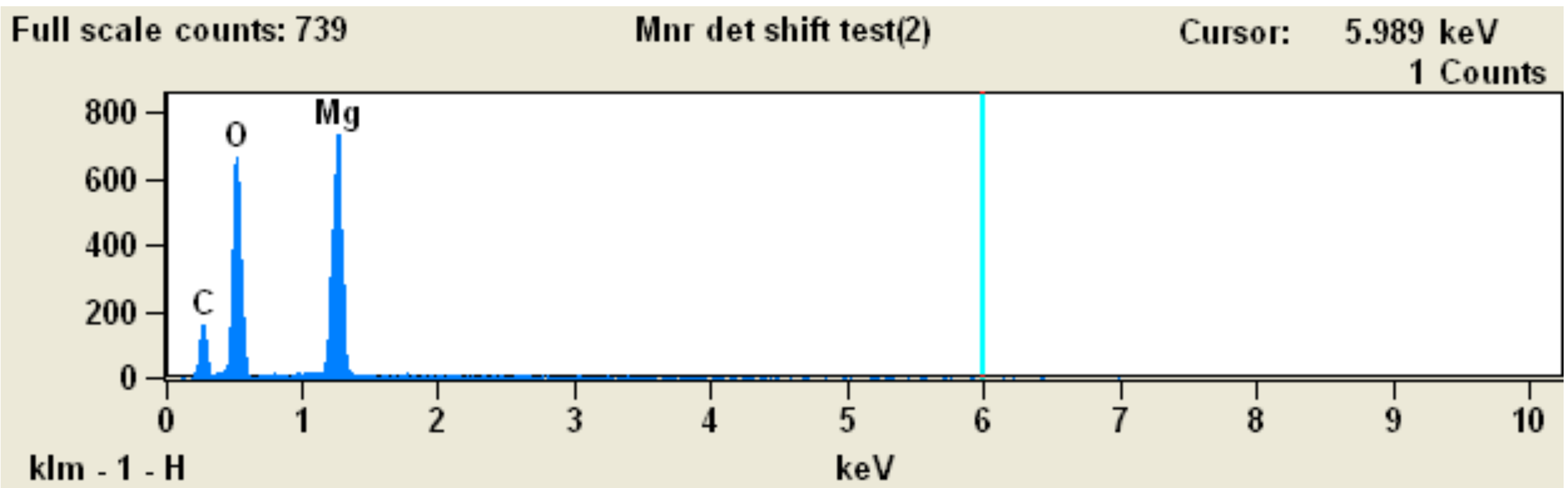

Figure S36. EDS data of **[Mg]**.

Table S7. EDS data of **[Mg]**. Sodium was not detected in the sample, placing an upper limit on the sodium composition of the sample.

Element Line	Net Counts	Source	Weight %	Weight % Error	Atom %	Atom % Error	Compound %
**C K**	975	EDS	24.28	± 0.97	33.17	± 1.33	24.28
**O K**	4593	EDS	44.89	± 0.74	46.03	± 0.76	44.89
**Mg K**	6003	EDS	30.83	± 0.42	20.81	± 0.28	30.83

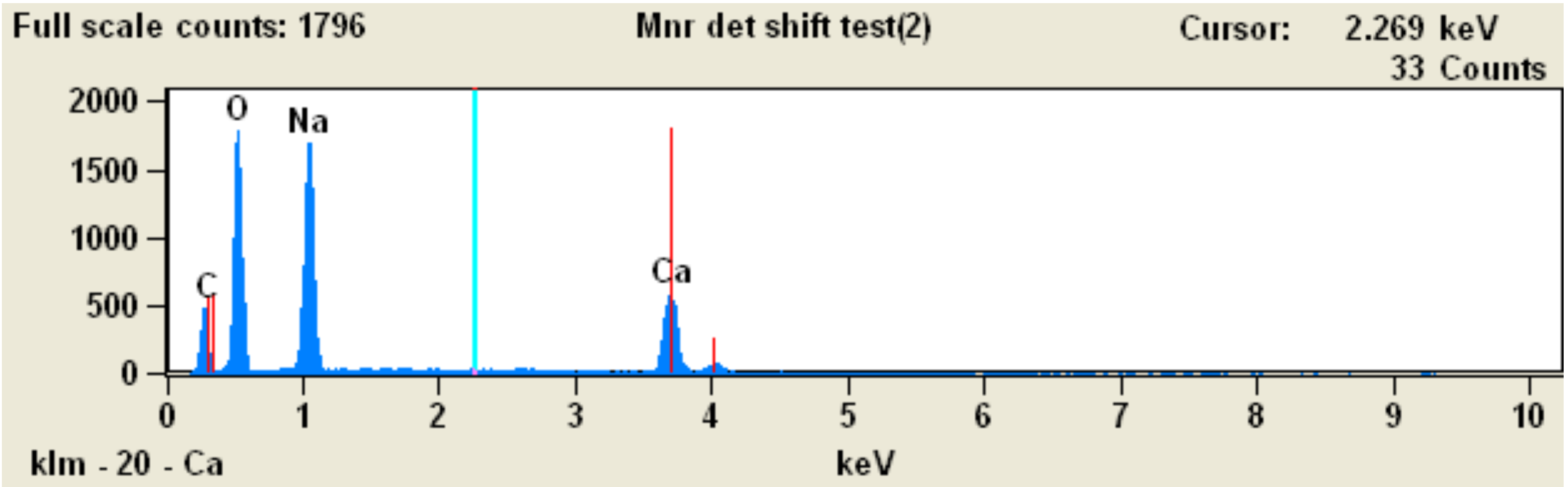

Figure S37. EDS data of **[Ca·Na$_2$]**.

Table S8. EDS data of **[Ca·Na$_2$]**.

Element Line	Net Counts	Source	Weight %	Weight % Error	Atom %	Atom % Error	Compound %
**C K**	3253	EDS	19.92	± 0.40	29.12	± 0.58	19.92
**O K**	12088	EDS	42.82	± 0.45	47.01	± 0.50	42.82
**Na K**	13335	EDS	23.15	± 0.23	17.68	± 0.18	23.15
**Ca K**	7756	EDS	14.12	± 0.26	6.19	± 0.11	14.12

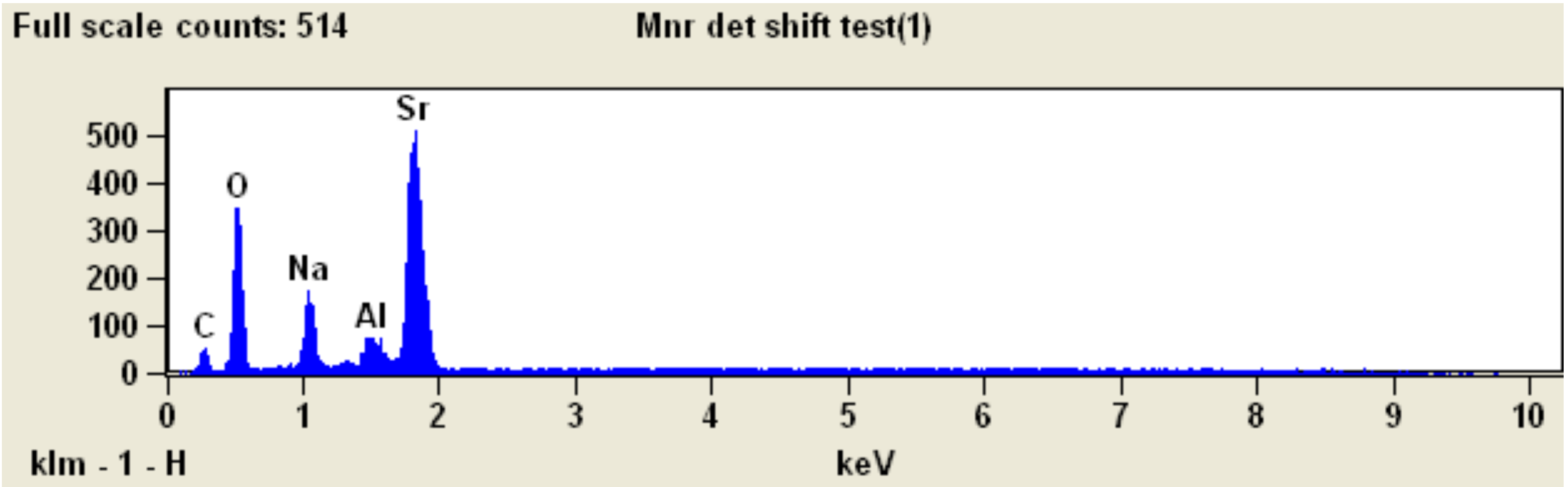

Figure S38. EDS data of **[Sr·Na]$_2$**. It is believed that the Al detected is actually Br.

Table S9. EDS data of **[Sr·Na]$_2$**. It is believed that the Al detected is actually Br.

**Element Line**	**Net Counts**	**Source**	**Weight %**	**Weight % Error**	**Atom %**	**Atom % Error**	**Compound %**
**C K**	250	EDS	12.28	± 0.98	27.29	± 2.18	12.28
**O K**	2457	EDS	27.86	± 0.67	46.46	± 1.12	27.86
**Na K**	1179	EDS	7.35	± 0.34	8.53	± 0.39	7.35
**Al K**	514	EDS	2.51	± 0.29	15.23	± 0.33	2.51
**Sr L**	6605	EDS	50.00	± 1.09	15.23	± 0.33	50.00

5. Reactivity with H_2

5.1 Treatment of NaTEMPO with H_2:

NaTEMPO (0.717 g, 4.00 mmol) was dissolved in diethyl ether (10 mL) and the solution was transferred to a Schlenk flask (50 mL) containing a stir bar. The solution was removed from the glovebox and degassed by one freeze-pump-thaw cycle, then evacuating the headspace under dynamic vacuum. The flask was then backfilled with H_2 (ca. 2 atm) for 10 minutes, then closed to H_2 and set to stir for 16 h. After ca. 16 h, the flask was again backfilled with H_2 (ca. 2 atm) for 20 minutes, then closed to H_2 and set to stir for 1 h. The flask was brought back into the glovebox where all volatile materials were removed under reduced pressure, providing a light pink solid. The ^{1}H NMR (400 MHz, benzene-d_6, Fig. S39) spectrum of the sample analyzed after isolation of the solids matched that of NaTEMPO reported above (Fig. S1), indicating that a reaction of NaTEMPO with H_2 had not occurred.

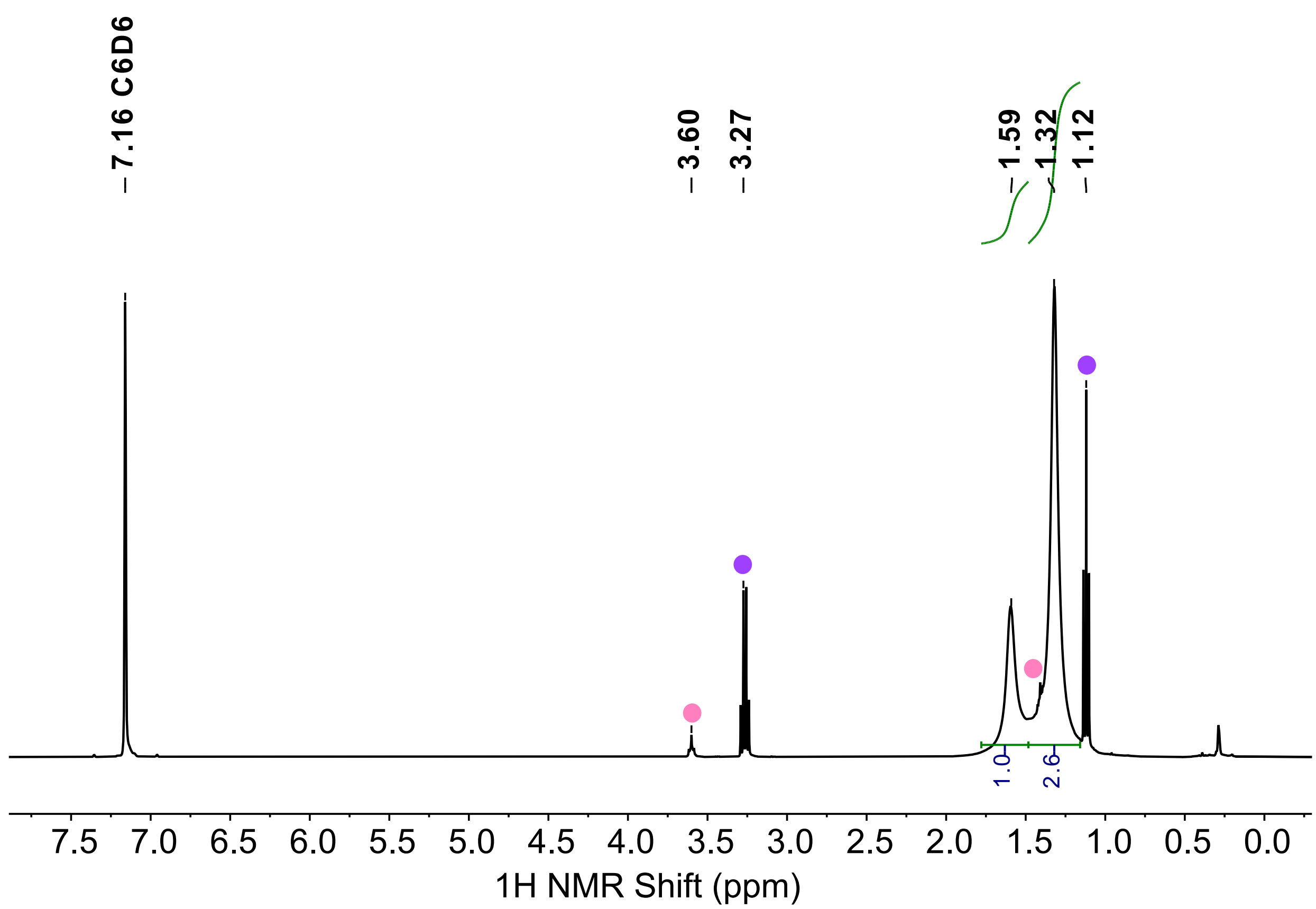

Figure S39. ^{1}H NMR (400 MHz, benzene-d_6, 25 °C) spectrum of the isolated solids obtained after workup of reaction mixture obtained by treatment of NaTEMPO with H_2 (ca. 2 atm). The pink dots indicate resonances assigned to THF, the purple dots indicate resonances assigned to diethyl ether.

5.2 Treatment of [Mg] with H_2

[Mg] (0.007 g, 0.009 mmol) was dissolved in benzene-*d*$_6$ (2 mL) in a 4 mL vial containing a stir bar, then the vial was loaded into a Parr reactor in the glovebox. The reactor was then sealed, removed from the glovebox, and connected to a vacuum gas manifold containing H_2. The line between the Parr reactor and the H_2 cylinder was cycled three times between vacuum and H_2. The reactor was filled with H_2 (ca. 8 atm.), sealed, and set to stir for 48 h. After ca. 48 h, the reactor was brought back into the glovebox where an aliquot of the solution was taken for ^{1}H NMR spectroscopic analysis. The ^{1}H NMR (400 MHz, benzene-*d*$_6$, Fig. S40) spectrum of the sample matched that of **[Mg]** reported above (Fig. S3), indicating that a reaction of **[Mg]** with H_2 had not occurred.

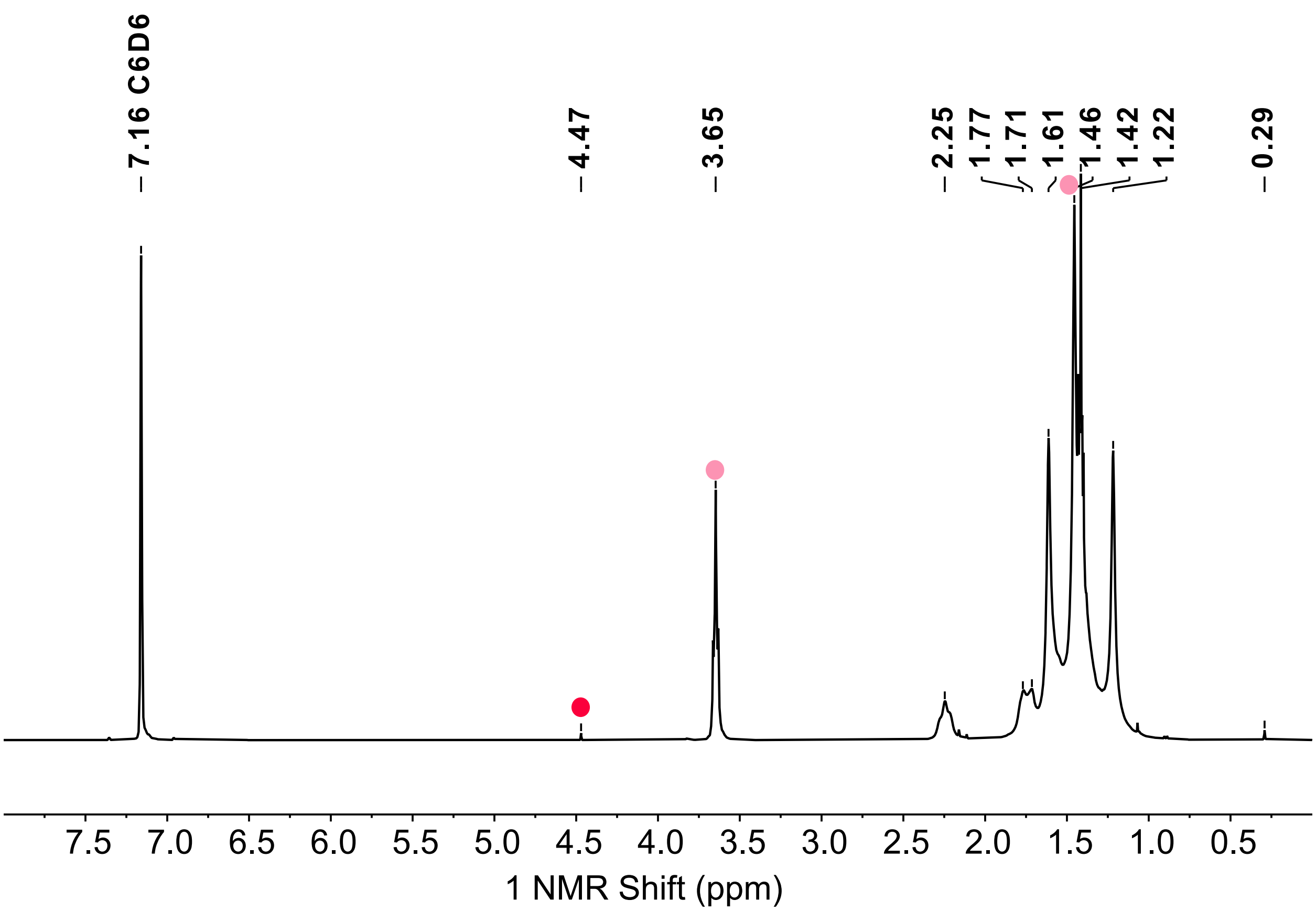

Figure S40. ^{1}H NMR (400 MHz, benzene-*d*$_6$, 25 °C) spectrum of the reaction mixture obtained by treatment of **[Mg]** with H_2 (ca. 8 atm). The red dot indicates a resonance assigned to H_2, the pink dots indicate resonances assigned to THF.

5.3 Treatment of [Ca·Na$_2$] with H_2

[Ca·Na$_2$] (0.015 g, 0.017 mmol) was dissolved in benzene-d_6 (1 mL) and 0.6 mL of the solution was transferred to a J Young tube (2 mL). The solution was removed from the glovebox and degassed by one freeze-pump-thaw cycle. The tube was backfilled with H_2 (ca. 3 atm) and after 48 h, NMR data was collected. The ^{1}H NMR (400 MHz, benzene-d_6, Fig. S41) spectrum of the reaction mixture matched that of **[Ca·Na$_2$]** reported above (Fig. S8), indicating that a reaction of **[Ca·Na$_2$]** with H_2 had not occurred.

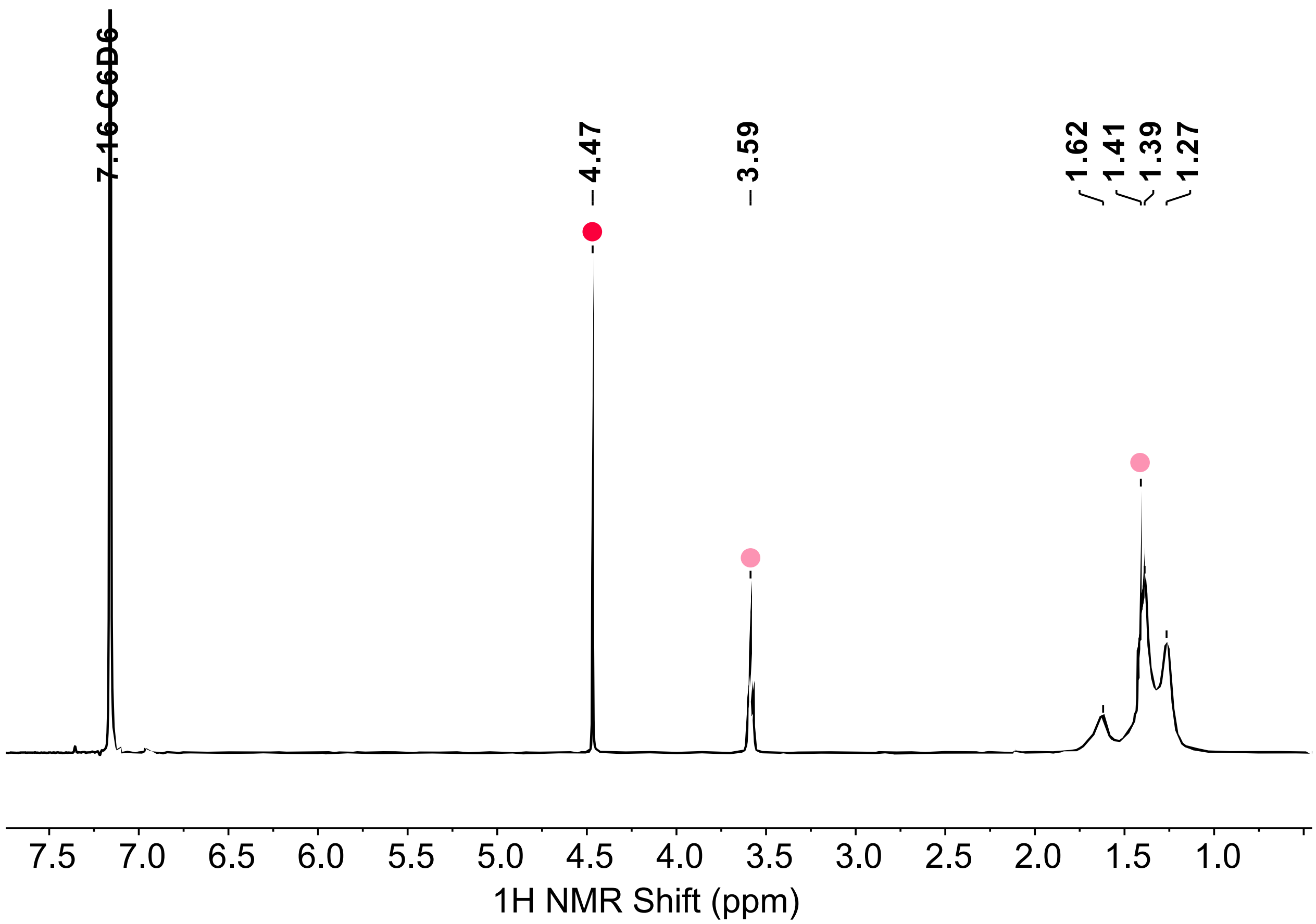

Figure S41. ^{1}H NMR (400 MHz, benzene-d_6, 25 °C) spectrum of the reaction mixture obtained by treatment of **[Ca·Na$_2$]** with H_2 (ca. 3 atm). The red dot indicates a resonance assigned to H_2, the pink dots indicate resonances assigned to THF.

5.4 Treatment of [Sr·Na]$_2$with H_2

[Sr·Na]$_2$ (0.004 g, 0.003 mmol) was dissolved in benzene-d_6 (1 mL) and 0.6 mL of the solution was transferred to a J Young tube (2 mL). The solution was removed from the glovebox and degassed by one freeze-pump-thaw cycle. The tube was backfilled with H_2 (ca. 3 atm) and after 48 h, NMR data was collected. The ^{1}H NMR (400 MHz, benzene-d_6, Fig. S42) spectrum of the reaction mixture matched that of **[Sr·Na]$_2$** reported above (Fig. S14), indicating that a reaction of **[Sr·Na]$_2$** with H_2 had not occurred.

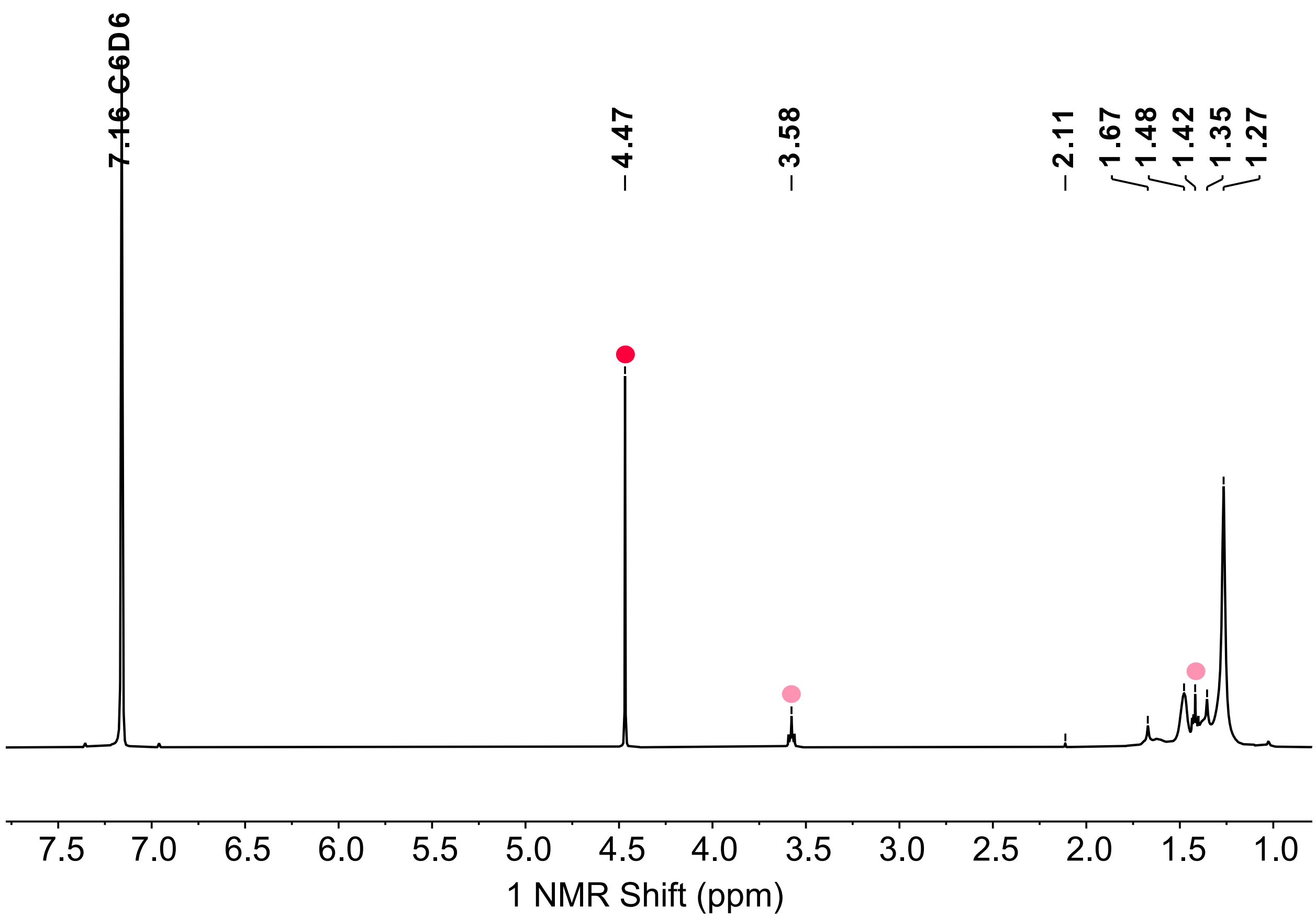

Figure S42. ^{1}H NMR (400 MHz, benzene-d_6, 25 °C) spectrum of the reaction mixture obtained by treatment of **[Sr·Na]$_2$** with H_2 (ca. 3 atm). The red dot indicates a resonance assigned to H_2, the pink dots indicate resonances assigned to THF.

5.5 Treatment of [Zn] with H_2

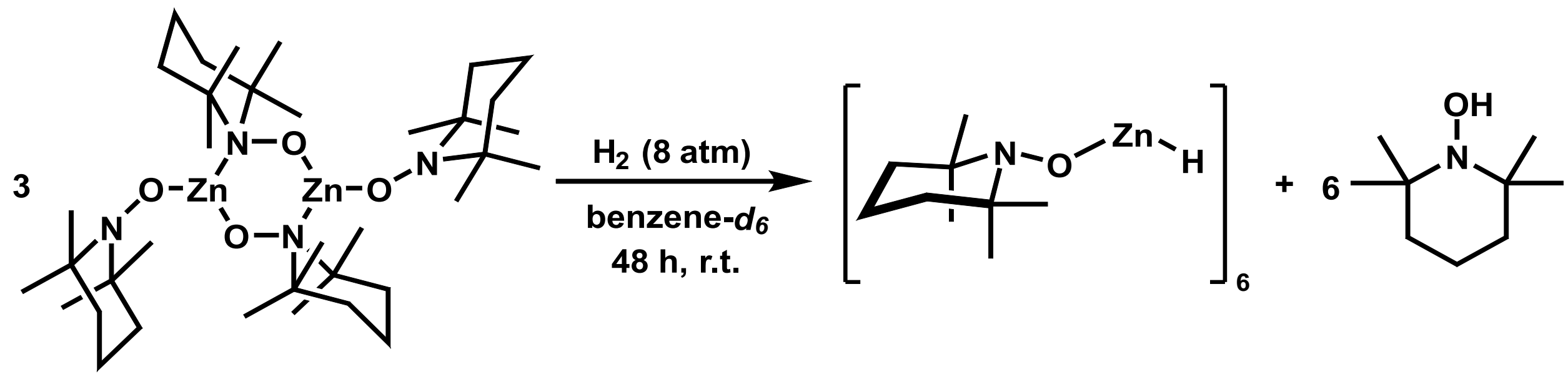

[Zn] (0.015 g, 0.020 mmol) was dissolved in benzene-*d*$_6$ (3 mL) and combined with a solution of 1,3,5-trimethoxybenzene (0.35 mL, 59 mM in benzene-*d*$_6$, 0.020 mmol) in a 20 mL scintillation vial containing a stir bar, then the vial was loaded into a Parr reactor in the glovebox. The reactor was then sealed, removed from the glovebox, and connected to a vacuum gas manifold containing H_2. The line between the Parr reactor and the H_2 cylinder was cycled three times between vacuum and H_2. The reactor was filled with H_2 (ca. 8 atm.), sealed, and set to stir for 48. After ca. 48 h, the reactor was brought back into the glovebox where an aliquot of the solution was taken for ^{1}H NMR spectroscopic analysis and the formation of colorless crystals was observed. NMR data was collected with a relaxation delay set to 30 s. Analysis of the ^{1}H NMR (600 MHz, benzene-*d*$_6$, Fig. S43) spectrum of the reaction mixture indicated 58% yield of TEMPOH. The UV-vis absorbance spectrum (Fig. S44) of the reaction mixture did not show any peak in absorbance around 475 nm corresponding to free TEMPO. A unit cell of the crystals grown from solution assessed by SCXRD confirmed the identity of Zn(μ-H)(μ^2-η^1:η^1-TEMPO)]$_6$ in accordance with literature.[2]

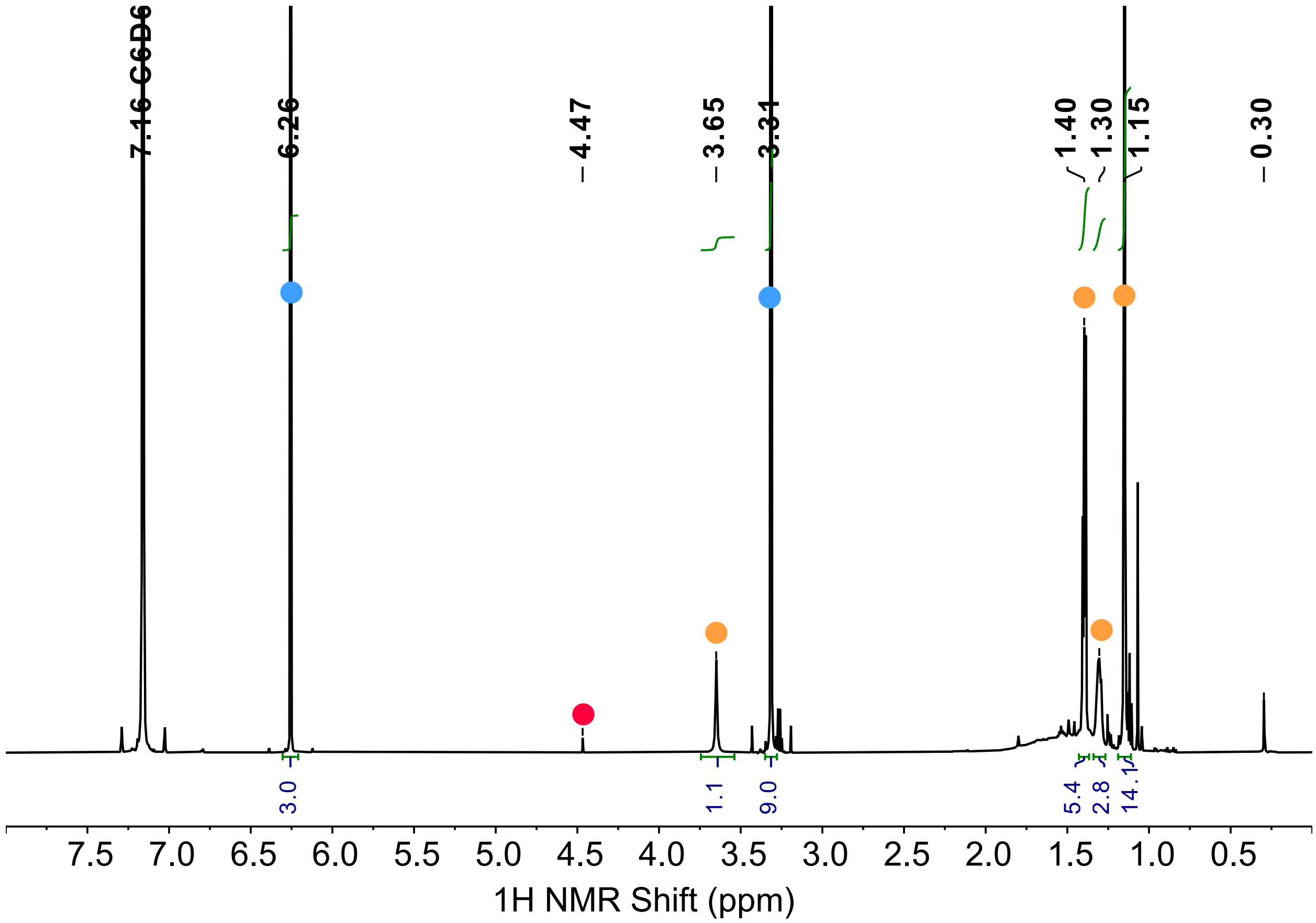

Figure S43. Quantitative ^{1}H NMR (600 MHz, benzene-d_6, 25 °C) spectrum of the reaction mixture obtained by treatment of **[Zn]** with H_2 (ca. 8 atm) using 1,3,5-trimethoxybenzene as an internal standard. The red dot indicates a resonance assigned to H_2, the blue dots indicate resonances assigned to 1,3,5-trimethoxybenzene, the orange dots indicate resonances assigned to TEMPOH.

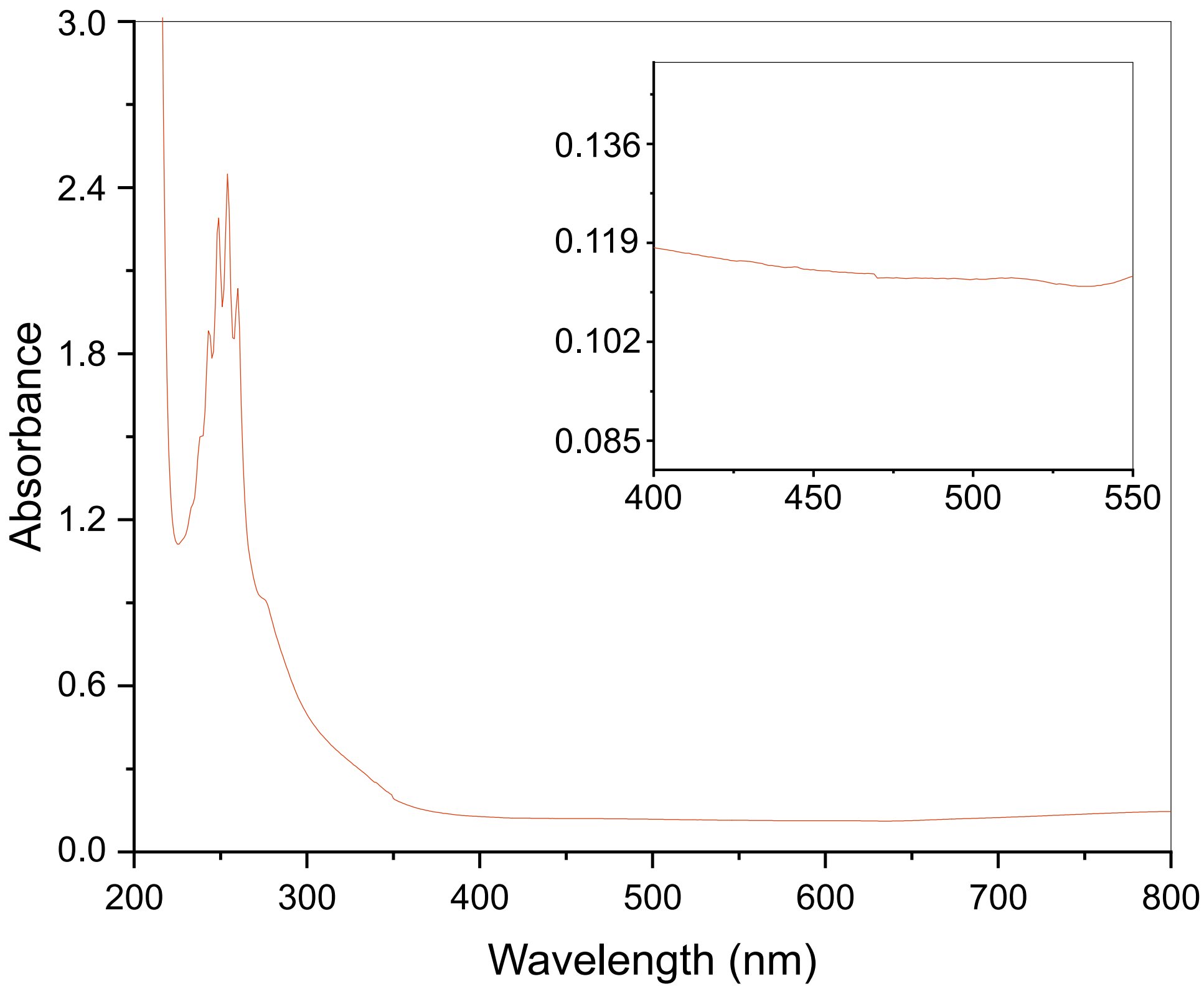

Figure S44. Absorbance spectrum of the reaction mixture obtained by treatment of **[Zn]** with H_2 (ca. 8 atm) in the presence of 1,3,5-trimethoxybenzene in benzene-d_6.

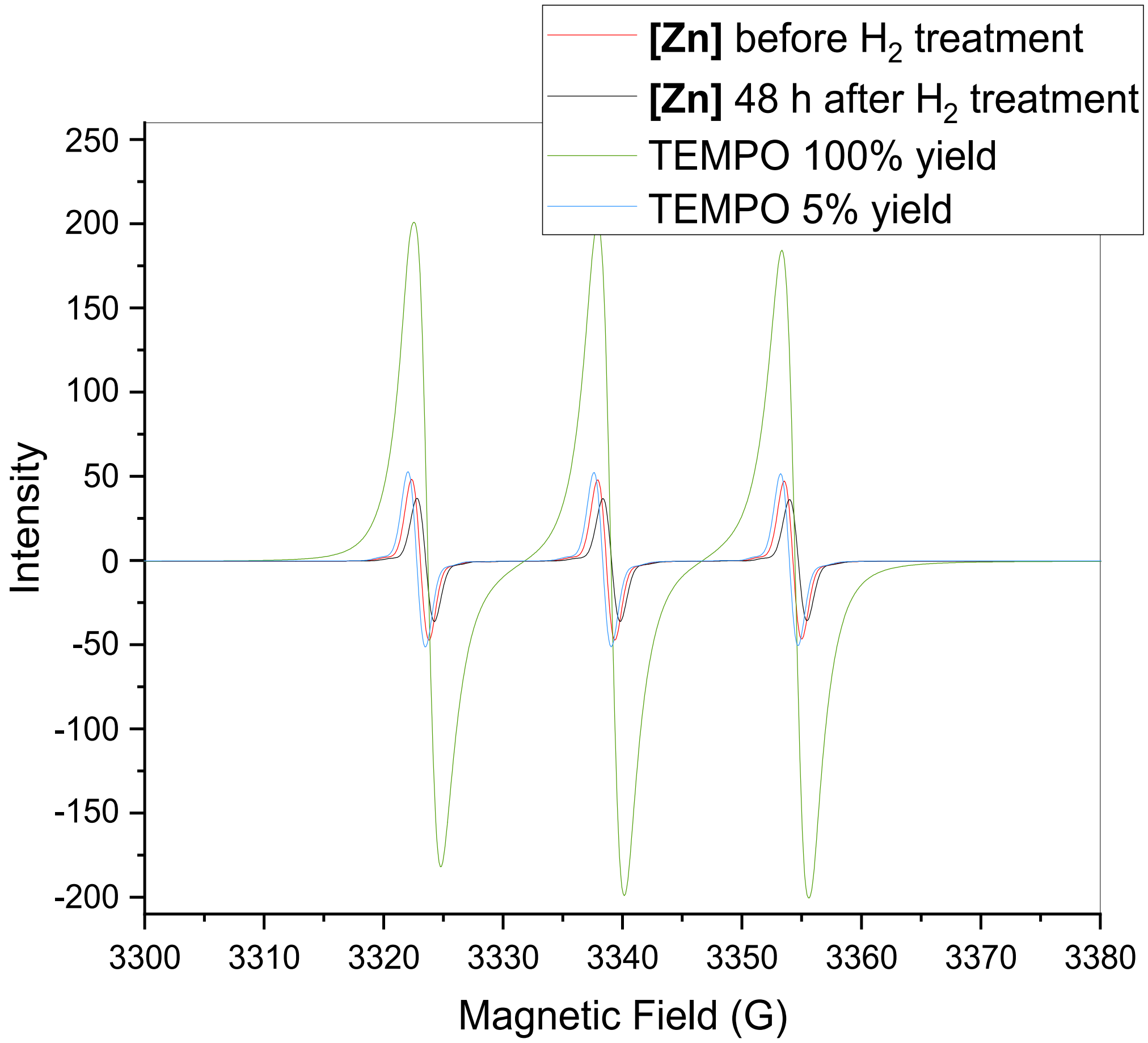

Figure S45. Stacked EPR spectra. **Red trace: [Zn]** (3.1 mM in benzene-d_6). A stock solution of **[Zn]** (0.051 g in 2.22 mL benzene-d_6) was prepared, and a 0.10 mL aliquot was diluted with 0.90 mL benzene-d_6. **Black trace: [Zn]** (3.1 mM in benzene-d_6) treated with H_2 (3 atm) for 48 h. A stock solution of **[Zn]** (0.051 g in 2.22 mL benzene-d_6) was prepared, and a 0.6 mL aliquot was transferred to a J Young tube (2 mL). The solution was removed from the glovebox and degassed by three freeze-pump-thaw cycles, backfilled with H_2 (ca. 3 atm), and after 48 h, 0.10 mL of this solution was removed and diluted with 0.90 mL benzene-d_6. **Green trace:** TEMPO (6.1 mM in benzene-d_6) representing a 100% theoretical yield of TEMPO in the reaction of **[Zn]** and H_2. A stock solution of TEMPO (19.2 mg in 2.0 mL benzene-d_6) was prepared, and a 0.10 mL aliquot was diluted with 0.90 mL benzene-d_6. **Blue trace:** TEMPO (0.31 mM in benzene-d_6), representing a 5% theoretical yield of TEMPO in the reaction of **[Zn]** and H_2. This solution was prepared *via* serial dilution of the TEMPO stock solution. EPR data was collected with the following parameters: Frequency = approx. 9.36 GHz, Power = 0.020000 mW, ModFreq = 100 kHz, ModAmp = 1.00 G, Attenuation = 40 dB, Time const. = 0.01 ms)

5.6 Treatment of [Sn] with H_2

[Sn] (0.010 g, 0.020 mmol) was dissolved in benzene-*d*$_6$ (0.6 mL) and transferred to a J Young tube (2 mL). The solution was removed from the glovebox and degassed by three freeze-pump-thaw cycles, backfilled with H_2 (ca. 3 atm), and after 18 h, NMR data was collected. The ^{1}H NMR (400 MHz, benzene-*d*$_6$, Fig. S45) spectrum of the reaction mixture revealed a new broad signal at 4.02 ppm and a sharp signal at 1.17 ppm indicating formation of TEMPOH. After repeating the experiment using D_2 (Fig. S60), we attribute this formation of TEMPOH to the reaction of **[Sn]** with trace oxygen or water, not to the reaction with H_2.

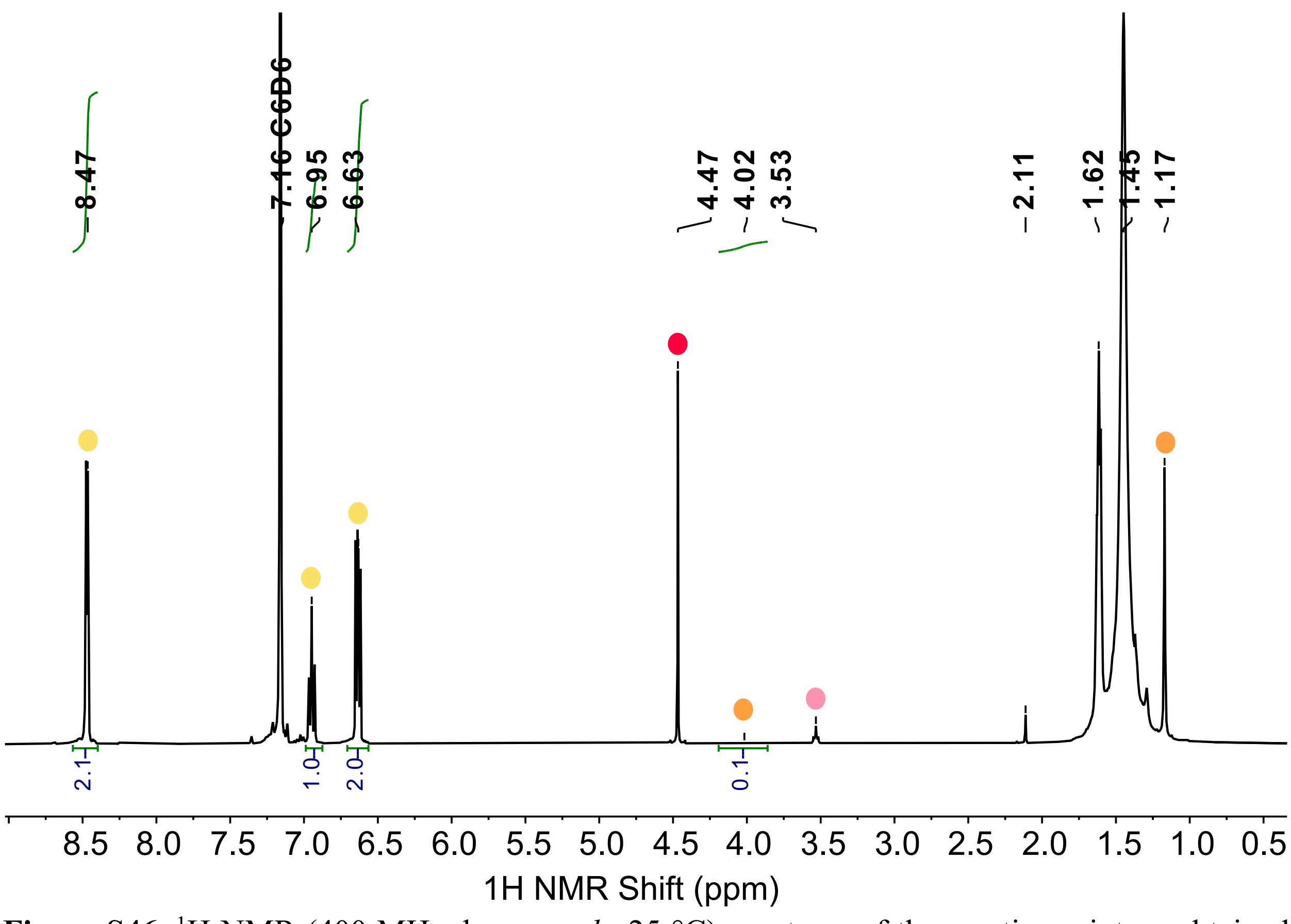

Figure S46. ^{1}H NMR (400 MHz, benzene-*d*$_6$, 25 °C) spectrum of the reaction mixture obtained by treatment of **[Sn]** with H_2 (ca. 3 atm). The red dot indicates a resonance assigned to H_2, the yellow dots indicate resonances assigned to pyridine, the orange dots indicate resonances assigned to TEMPOH.

5.7 Treatment of [Cd] with H_2

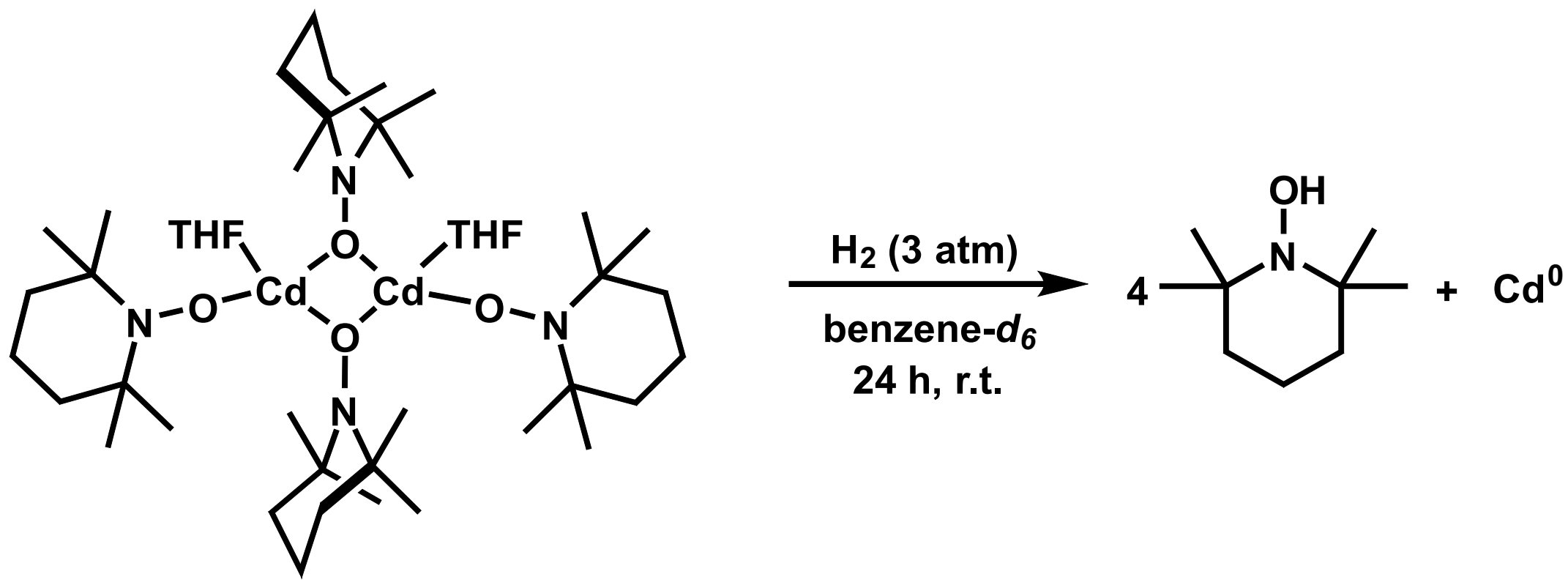

[Cd] (0.264 g, 0.266 mmol) was combined with a solution of 1,3,5-trimethoxybenzene (0.045 g, 0.27 mmol) in benzene-d_6 (4 mL) and the solution was transferred to a Schlenk flask (10 mL) containing a stir bar. The solution was removed from the glovebox and degassed by three freeze-pump-thaw cycle. The flask was then backfilled with H_2 (ca. 3 atm) and set to stir for 1 h during which time the formation of black solids was observed. After 1 h, the reactor was brought back into the glovebox where an aliquot of the solution was taken for ^{1}H NMR spectroscopic analysis. NMR data was collected with a relaxation delay set to 30 s to obtain quantitative ^{1}H NMR data.[3] Analysis of the ^{1}H NMR (600 MHz, benzene-d_6, Fig. S46) spectrum of the reaction mixture indicated 60% yield of TEMPOH.

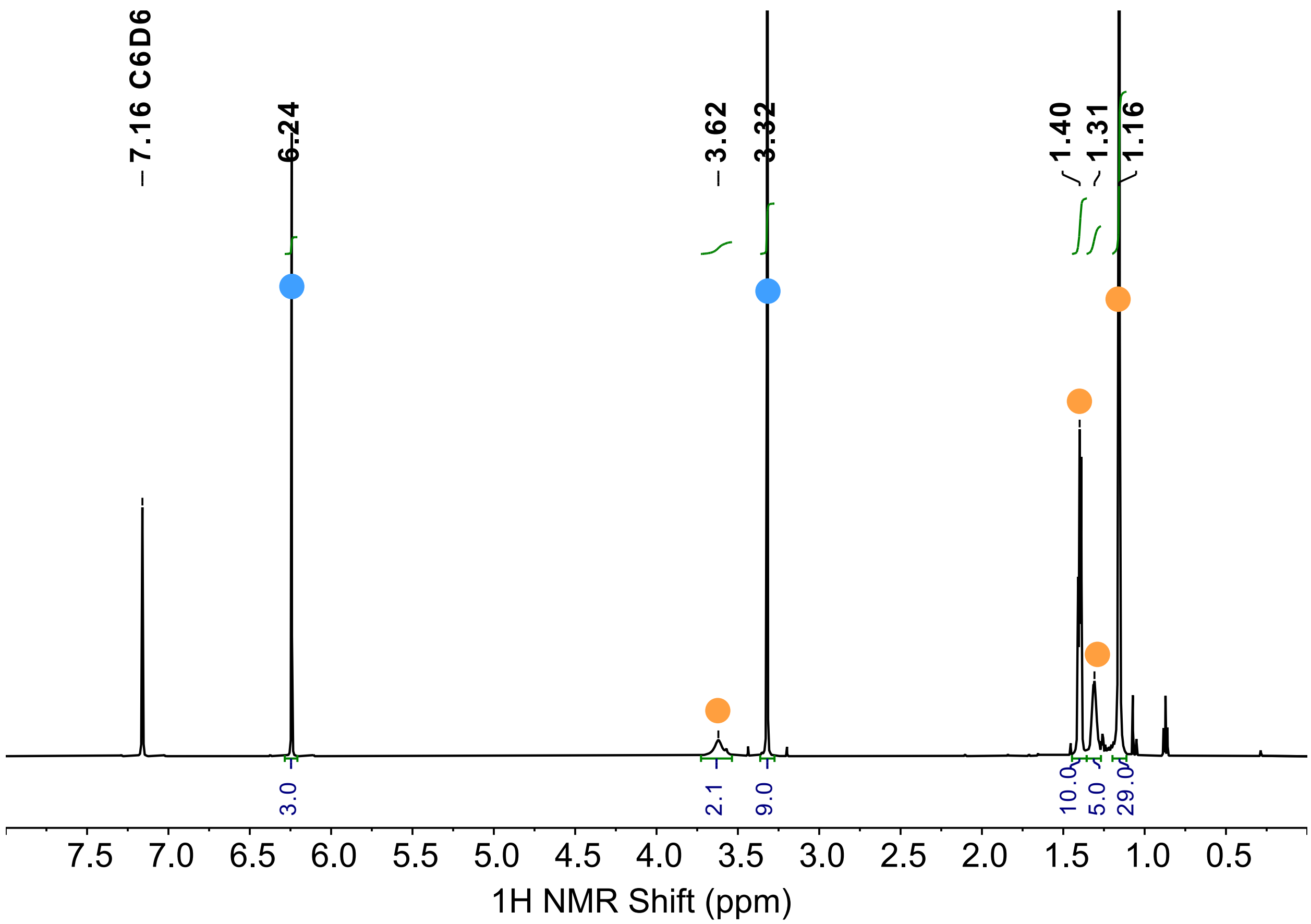

Figure S47. ^{1}H NMR (600 MHz, benzene-d_6, 25 °C) spectrum of the reaction mixture obtained by treatment of **[Cd]** with H_2 (ca. 3 atm). The blue dots indicate resonances assigned to 1,3,5-trimethoxybenzene, the orange dots indicate resonances assigned to TEMPOH.

To obtain solids for PXRD analysis, **[Cd]** (0.010 g, 0.023 mmol) was dissolved in benzene-d_6 (0.6 mL) and transferred to a J Young tube (2 mL). The solution was removed from the glovebox and degassed by three freeze-pump-thaw cycles, then backfilled with H_2 (ca. 3 atm). The formation of black solids was observed after 5 minutes. After 24 h, the sample was brought back into the glovebox, and all volatile materials were removed under dynamic vacuum. The resulting black solids were washed with toluene (2 x 2 mL), and all volatile materials were removed under dynamic vacuum. PXRD data was collected on the black solids (Fig. S47) in the 20-60° 2θ range which revealed a pattern corresponding to Cd^0.[4]

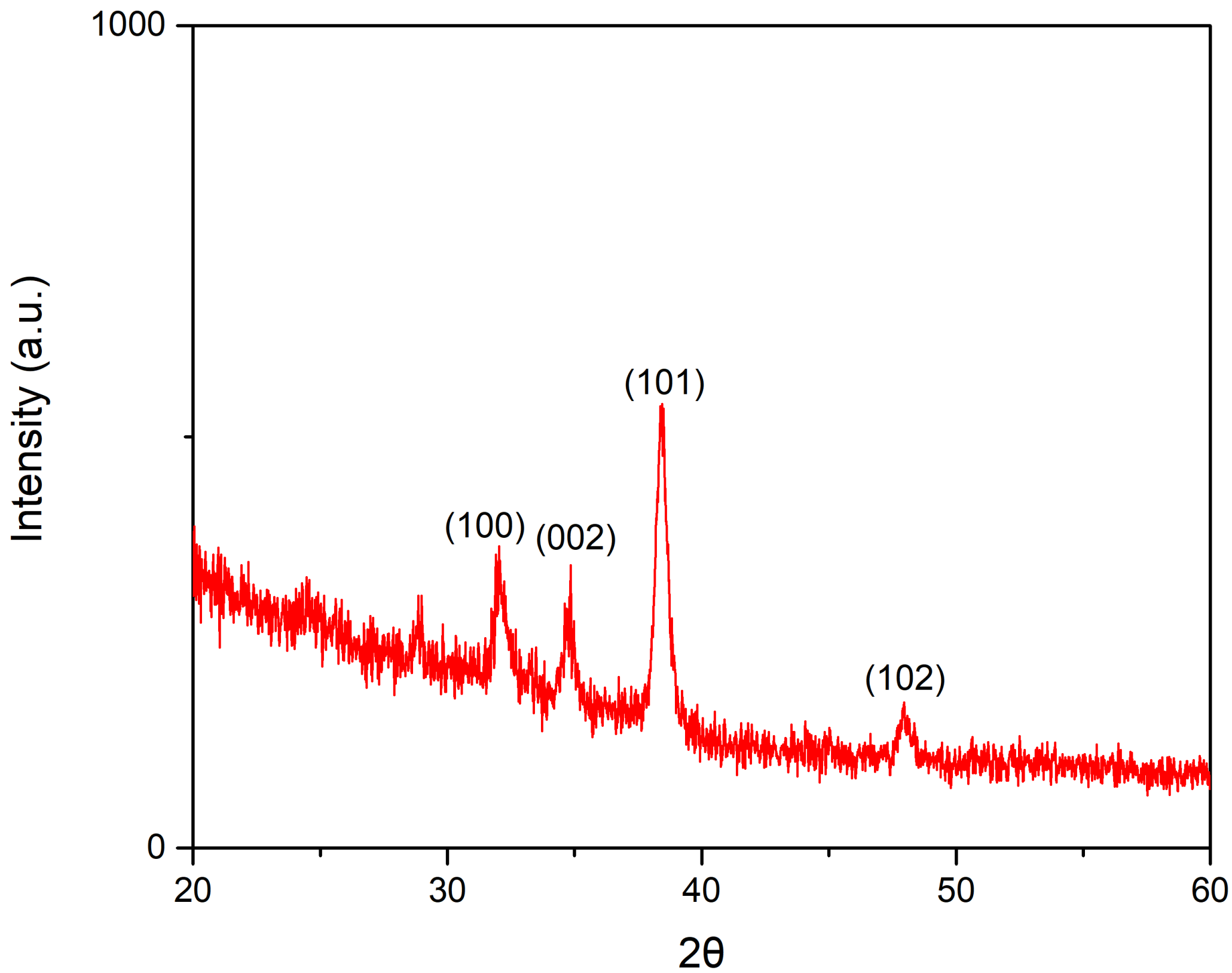

Figure S48. PXRD pattern of the solids obtained by treatment of **[Cd]** with H_2 (ca. 3 atm) (0.5° slit, 0.017° step size, and 35 s/step).

5.8 Treatment of $PbCl_2$ and NaTEMPO with H_2

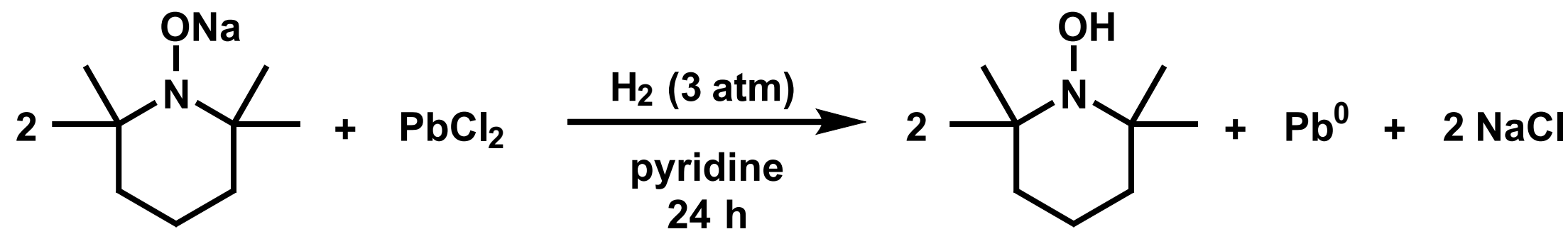

Cold pyridine (20 mL, −32 °C) was added to a cold (−32 °C) Schlenk flask containing a stir bar, $PbCl_2$ (0.200 g, 0.719 mmol, 1.00 equiv.), and NaTEMPO (0.258 g, 1.44 mmol, 2.00 equiv.). The solution was removed from the glovebox and degassed by three freeze-pump thaw cycles and allowed to warm to room temperature. After reaching room temperature the flask was backfilled with H_2 (ca. 3 atm) and set to stir for 24 h at room temperature during which time the formation of black solids was observed. After ca. 24 hours, the flask was brought back into the glovebox, the black solids were separated by centrifugation, washed with toluene (2 x 2 mL), and all volatile materials were removed from the solids under dynamic vacuum. An aliquot of the supernatant was removed for ^1H NMR spectroscopic analysis. NMR data was collected with a relaxation delay set to 30 s to obtain quantitative ^1H NMR data.[3] Analysis of the ^1H NMR (400 MHz, pyridine-d_5, Fig. S48) spectrum of the reaction mixture indicated 74% yield of TEMPOH. The ^1H NMR (300 MHz, benzene-d_6, Fig. S49) spectrum of TEMPOH in pyridine-d_5 is provided below for comparison. The solids were then taken for PXRD analysis (Fig. S50) in the 30-80° 2θ range which revealed a mixed powder pattern of NaCl and Pb^0.[5,6]

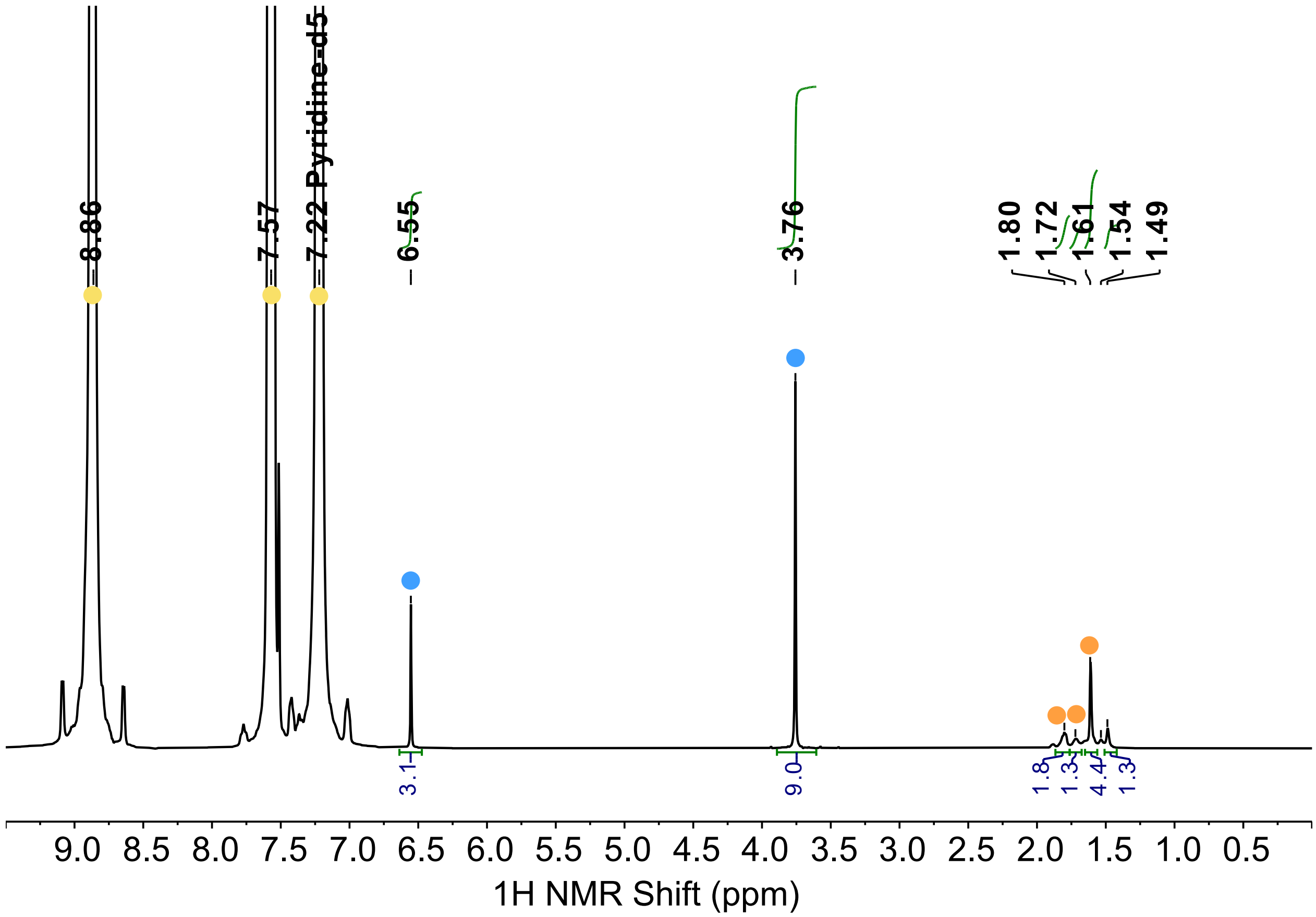

Figure S49. ^{1}H NMR (400 MHz, pyridine-d_5, 25 °C) spectrum of the reaction mixture obtained by treatment of PbCl with NaTEMPO and H_2 (ca. 3 atm). The blue dots indicate resonances assigned to 1,3,5-trimethoxybenzene, the orange dots indicate resonances assigned to TEMPOH.

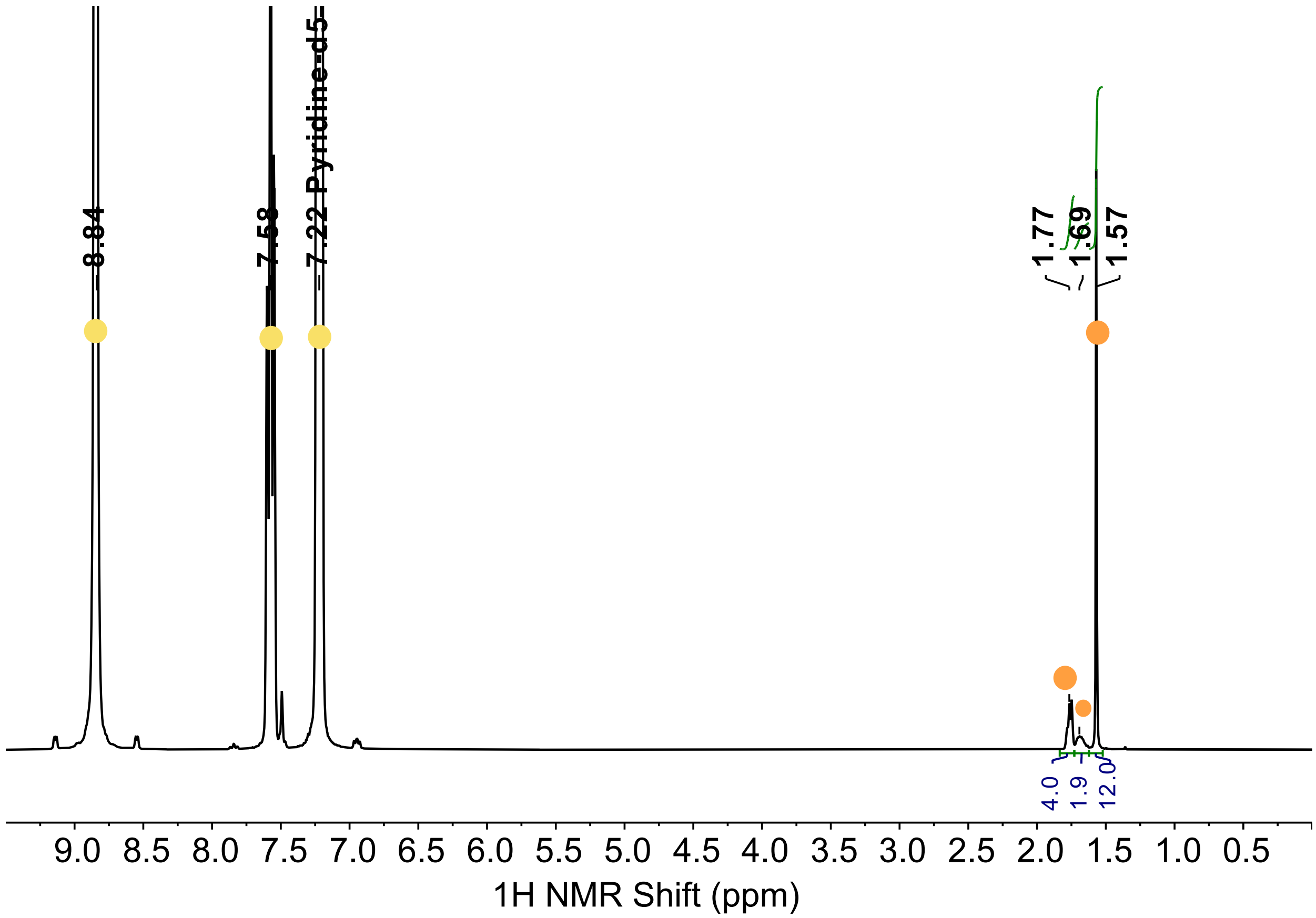

Figure S50. ^{1}H NMR (300 MHz, pyridine-d_5, 25 °C) spectrum of TEMPOH. The orange dots indicate resonances assigned to TEMPOH, the yellow dots indicate resonances assigned to pyridine.

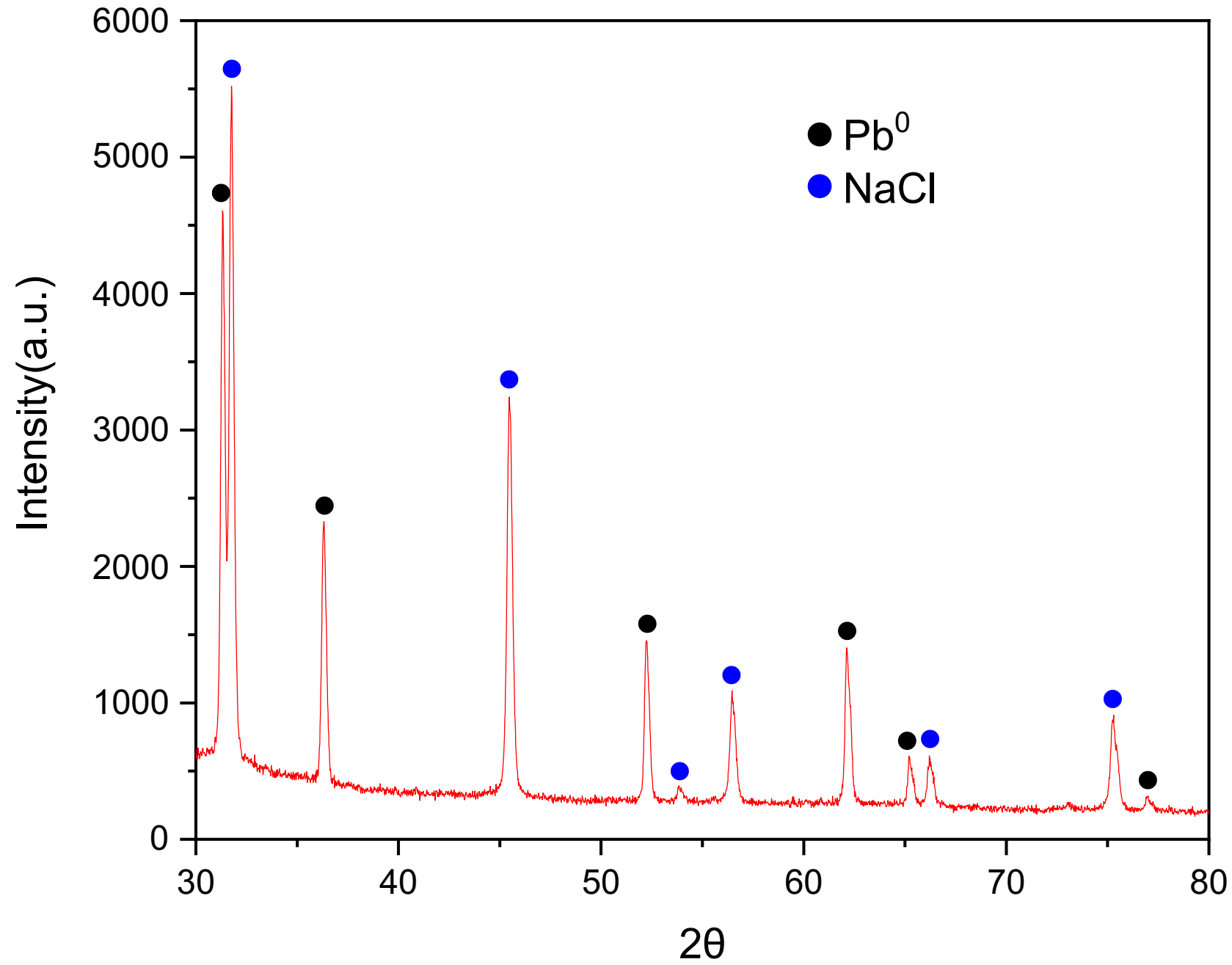

Figure S51. PXRD pattern of the solids obtained by treatment of NaTEMPO with $PbCl_2$ and H_2 (ca. 3 atm) (0.5° slit, 0.017° step size, and 65 s/step).

5.9 Treatment of $PbCl_2$ with H_2

A Straus flask containing a suspension of $PbCl_2$ (0.200 g, 0.719 mmol) in pyridine (20 mL) and a stir bar were prepared in the glovebox. The Straus flask was removed from the glovebox and degassed by three freeze-pump-thaw cycles. After being degassed, the Straus flask was charged with H_2 (ca. 3 atm) and allowed to stir for 24 hours. After 24 hours, the flask was brought back into the glovebox and all volatile materials were removed under dynamic vacuum, leaving behind white solids. The solids were then taken for PXRD analysis (Fig. S51) in the 20-50° 2θ range which revealed a pattern corresponding to $PbCl_2$.[7]

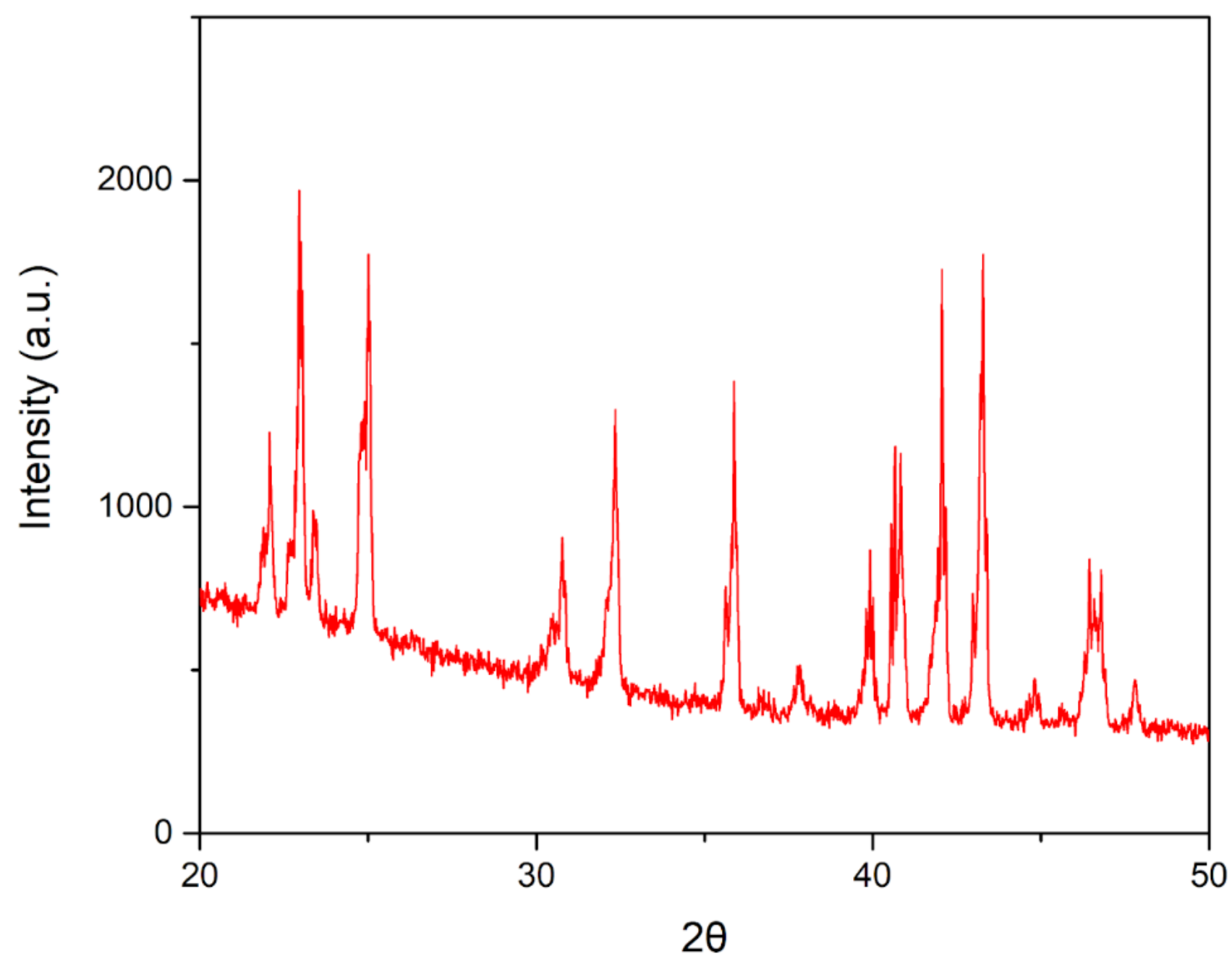

Figure S52. PXRD diffraction pattern of the solids obtained by treatment of $PbCl_2$ with H_2 (ca. 3 atm) (0.5° slit, 0.017° step size, and 50 s/step).[7]

6. Reactivity with D_2

6.1 Treatment of [Mg] with D_2

[Mg] (0.008 g, 0.009 mmol) was dissolved in benzene-d_6 (2 mL) in a 4 mL vial containing a stir bar, then the vial was loaded into a Parr reactor in the glovebox. The reactor was then sealed, removed from the glovebox, and connected to a vacuum gas manifold containing D_2. The line between the Parr reactor and the D_2 cylinder was cycled three times between vacuum and D_2. The reactor was backfilled with H_2 (ca. 5.45 atm.), sealed, and set to stir for 48 h. After ca. 48 h, the reactor was brought back into the glovebox where an aliquot of the solution was taken for ^1H and ^2H NMR spectroscopic analysis. The ^1H NMR (600 MHz, benzene-d_6, Fig. S52) spectrum of the sample showed that both **[Mg]** and TEMPOH were present which we attributed to undesired reaction with trace oxygen or water. The ^2H NMR (92 MHz, benzene-d_6, Fig. S53) spectrum of the sample showed no TEMPOD was formed.

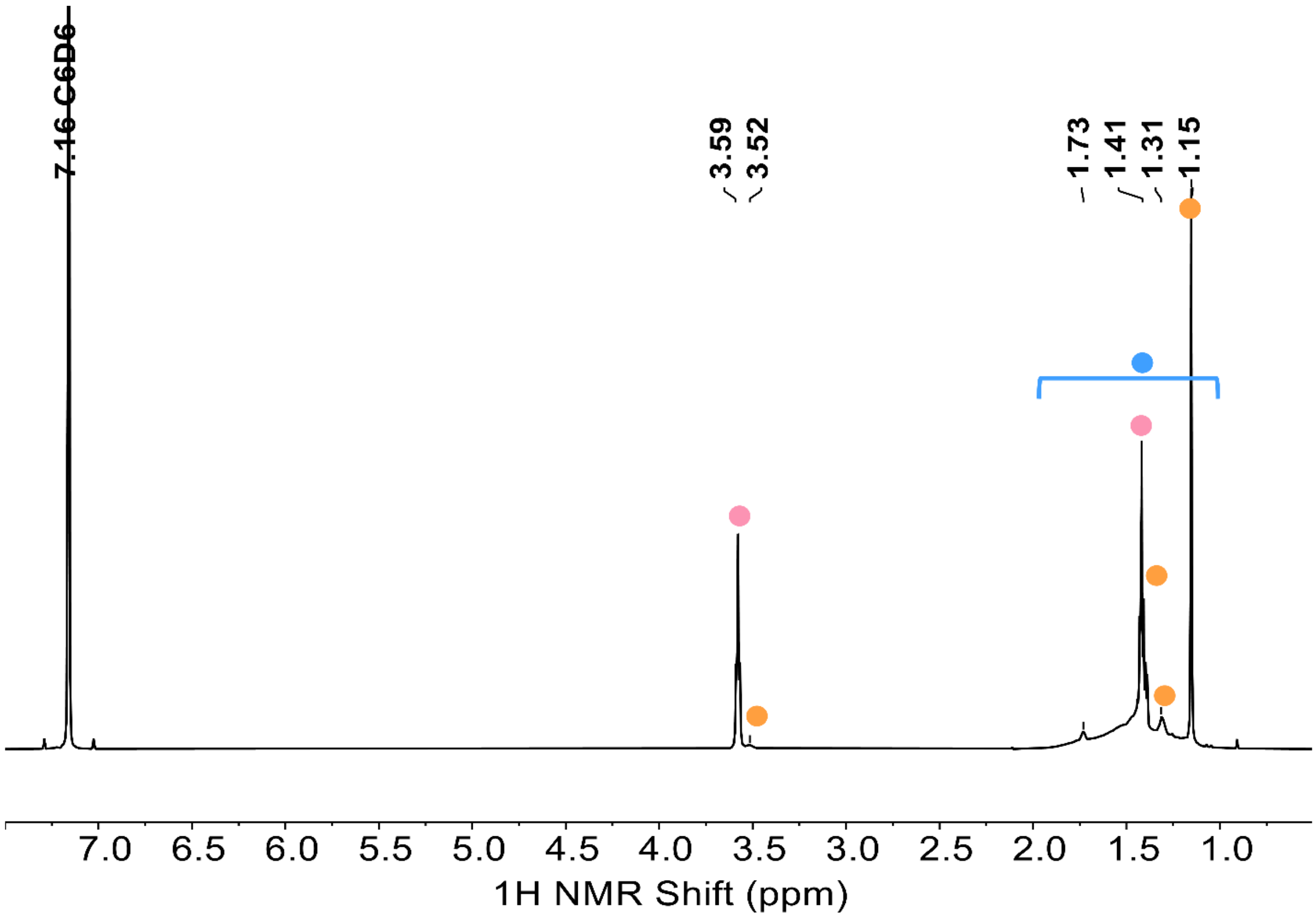

Figure S53. ^{1}H NMR (600 MHz, benzene-d_6, 25 °C) spectrum of the reaction mixture obtained by treatment of **[Mg]** with D_2 (ca 5.45 atm). The blue dot indicates broad resonances assigned to **[Mg]**, the orange dots indicate resonances assigned to TEMPOH, the pink dots indicate resonances assigned to THF.

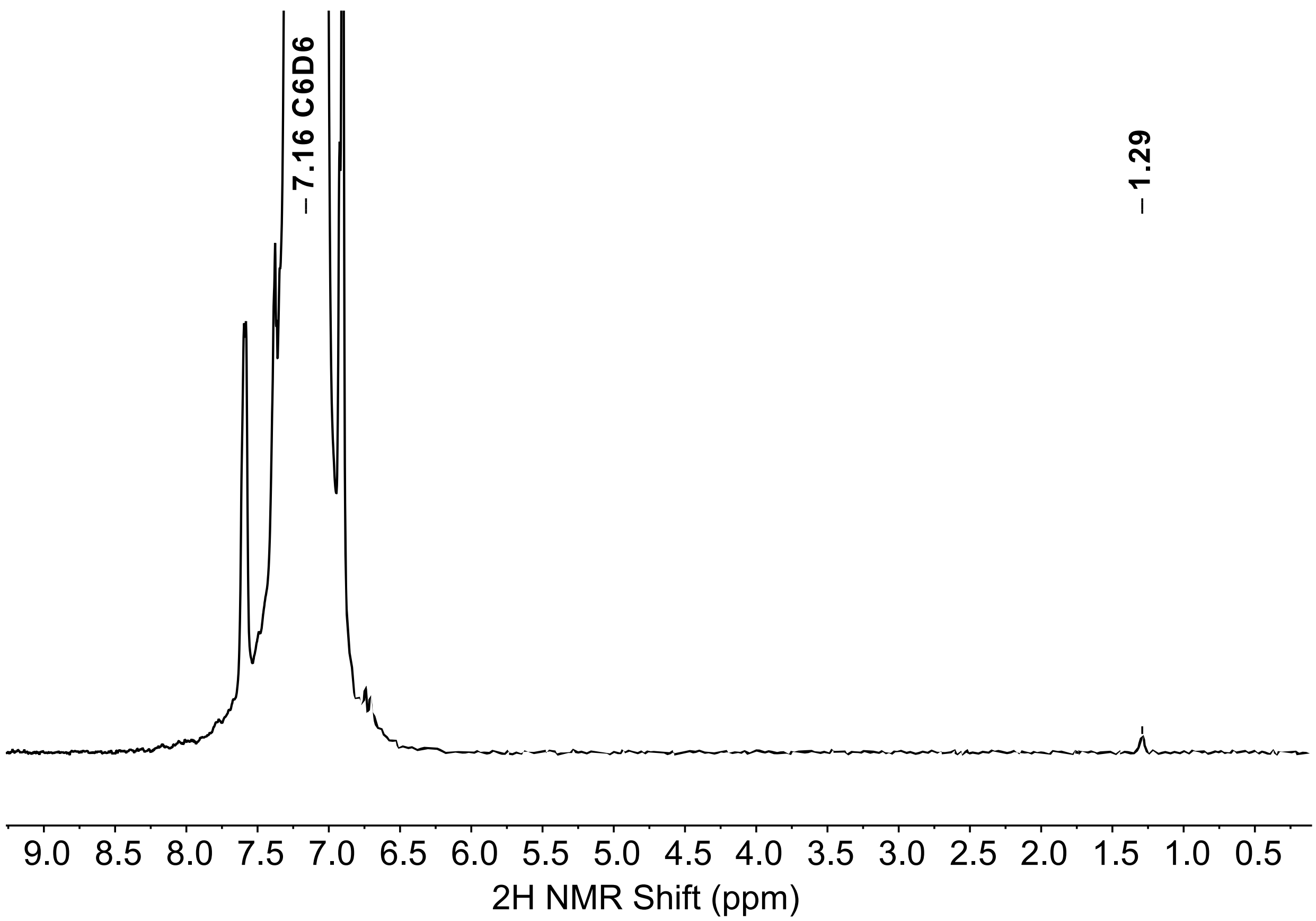

Figure S54. ^{2}H NMR (92 MHz, benzene-d_6, 25 °C) spectrum of the reaction mixture obtained by treatment of **[Mg]** with D_2 (ca. 5.45 atm). The resonance at 1.29 ppm is attributed to an impurity present in the NMR solvent.

6.2 Treatment of [Zn] with D_2

Treatment of **[Zn]** with D_2 in benzene-d_6:

[Zn] (0.007 g, 0.01 mmol) was dissolved in benzene-d_6 (2 mL) in a 4 mL vial containing a stir bar, then the vial was loaded into a Parr reactor in the glovebox. The reactor was then sealed, removed from the glovebox, and connected to a vacuum gas manifold containing D_2. The line between the Parr reactor and the D_2 cylinder was cycled three times between vacuum and D_2. The reactor was filled with H_2 (ca. 8 atm.), sealed, and set to stir for 48 h. After ca. 48 h, the reactor was brought back into the glovebox where an aliquot of the solution was taken for ^1H and ^2H NMR spectroscopic analysis. The ^1H NMR (600 MHz, benzene-d_6, Fig. S54) spectrum of the sample indicated formation of 10% TEMPOH and 90% TEMPOD based on the integration of the TEMPOH (O–H) resonance relative to the remaining TEMPOH/D resonances. We attributed the TEMPOH formation to deleterious reaction with trace oxygen or water. The ^2H NMR (92 MHz, benzene-d_6, Fig. S55) spectrum of the sample revealed a new resonance at 3.49 ppm which we attributed to TEMPOD formation.

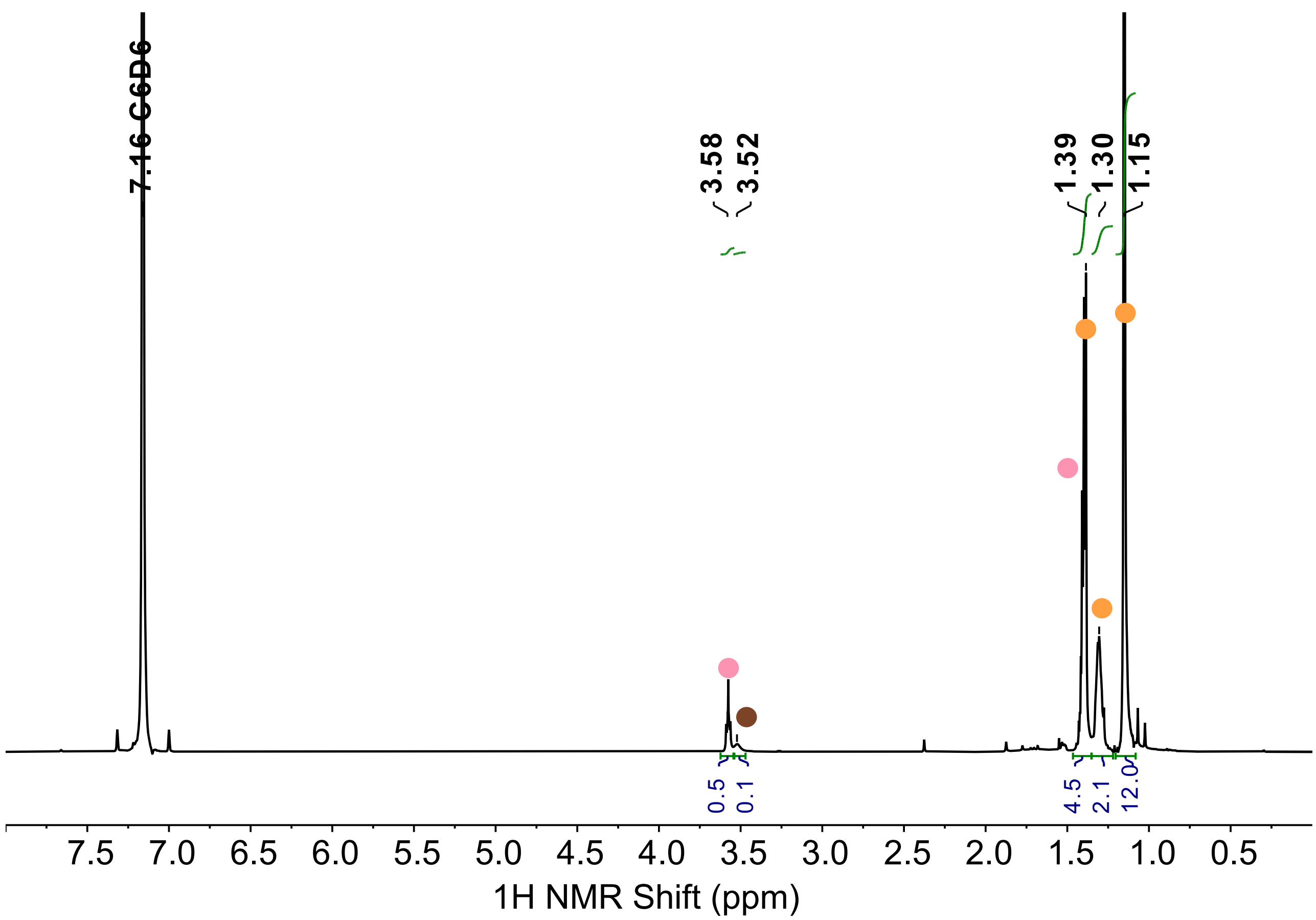

Figure S55. ^1H NMR (600 MHz, benzene-d_6, 25 °C) spectrum of the reaction mixture obtained by treatment of **[Zn]** with D_2 (ca. 8 atm) in benzene-d_6. The orange dots indicate resonances assigned to TEMPOH/TEMPOD, the brown dot indicates a resonance assigned to TEMPOH (O-H), the pink dots indicate resonances assigned to THF.

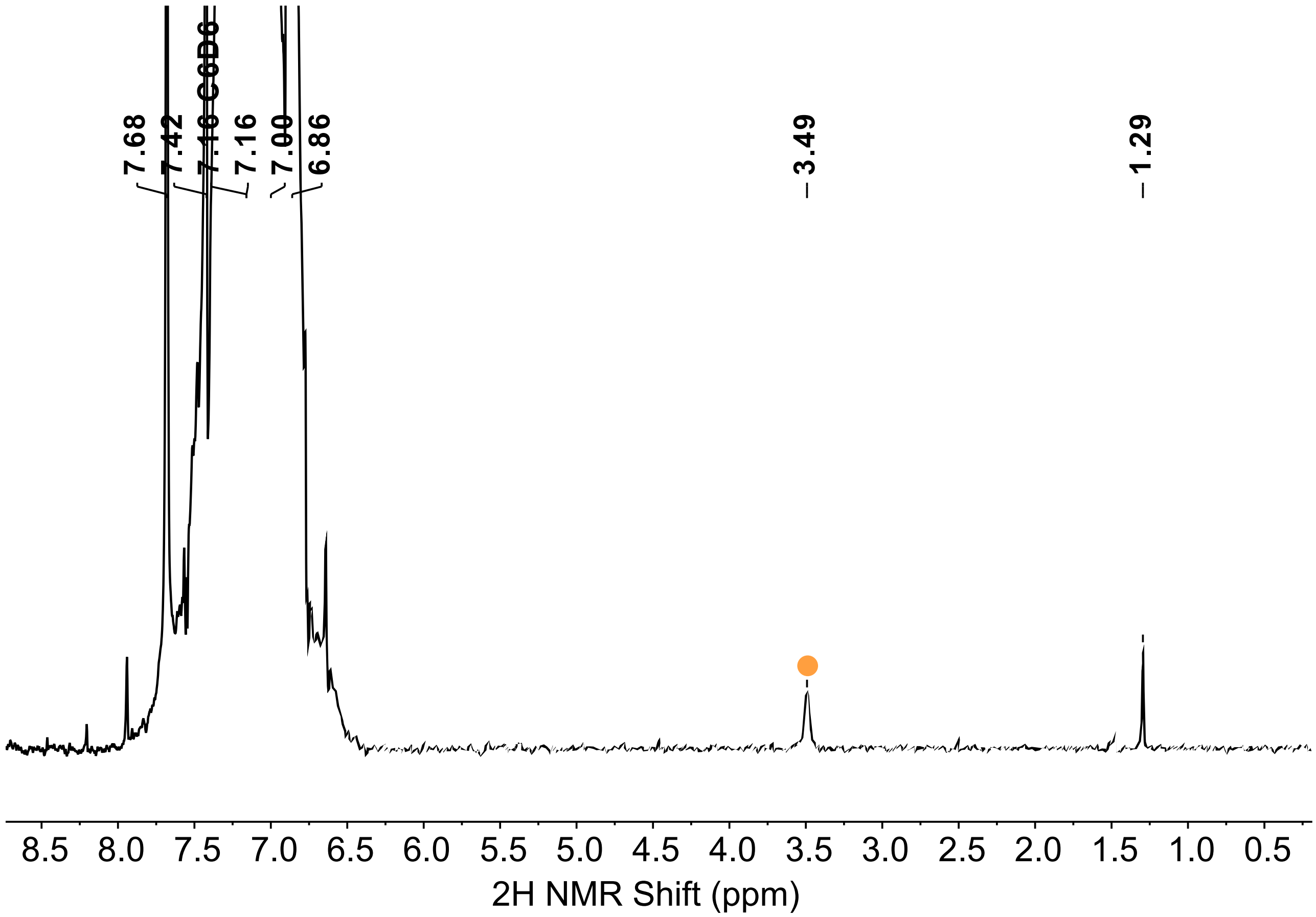

Figure S56. ^{2}H NMR (92 MHz, benzene-d_6, 25 °C) spectrum of the reaction mixture obtained by treatment of **[Zn]** with D_2 (ca. 8 atm) in benzene-d_6. The orange dot indicates a resonance attributed to TEMPOD, the resonance at 1.29 ppm is attributed to an impurity present in the NMR solvent.

Treatment of **[Zn]** with D_2 in pyridine-d_5:

[Zn] (0.008 g, 0.01 mmol) was dissolved in benzene-d_6 (0.6 mL) and transferred to a J Young tube (2 mL). The solution was removed from the glovebox and degassed by one freeze-pump-thaw cycle, backfilled with D_2 (ca. 3 atm), and after 48 h, NMR data was collected. The ^{1}H NMR (600 MHz, pyridine-d_5, Fig. S56) spectrum of the reaction mixture matched that of **[Zn]** reported previously,[3] indicating that a reaction of **[Zn]** with D_2 in pyridine-d_5 had not occurred. The ^{2}H NMR (92 MHz, pyridine-d_5, Fig. S57) spectrum of the sample showed no TEMPOD was formed.

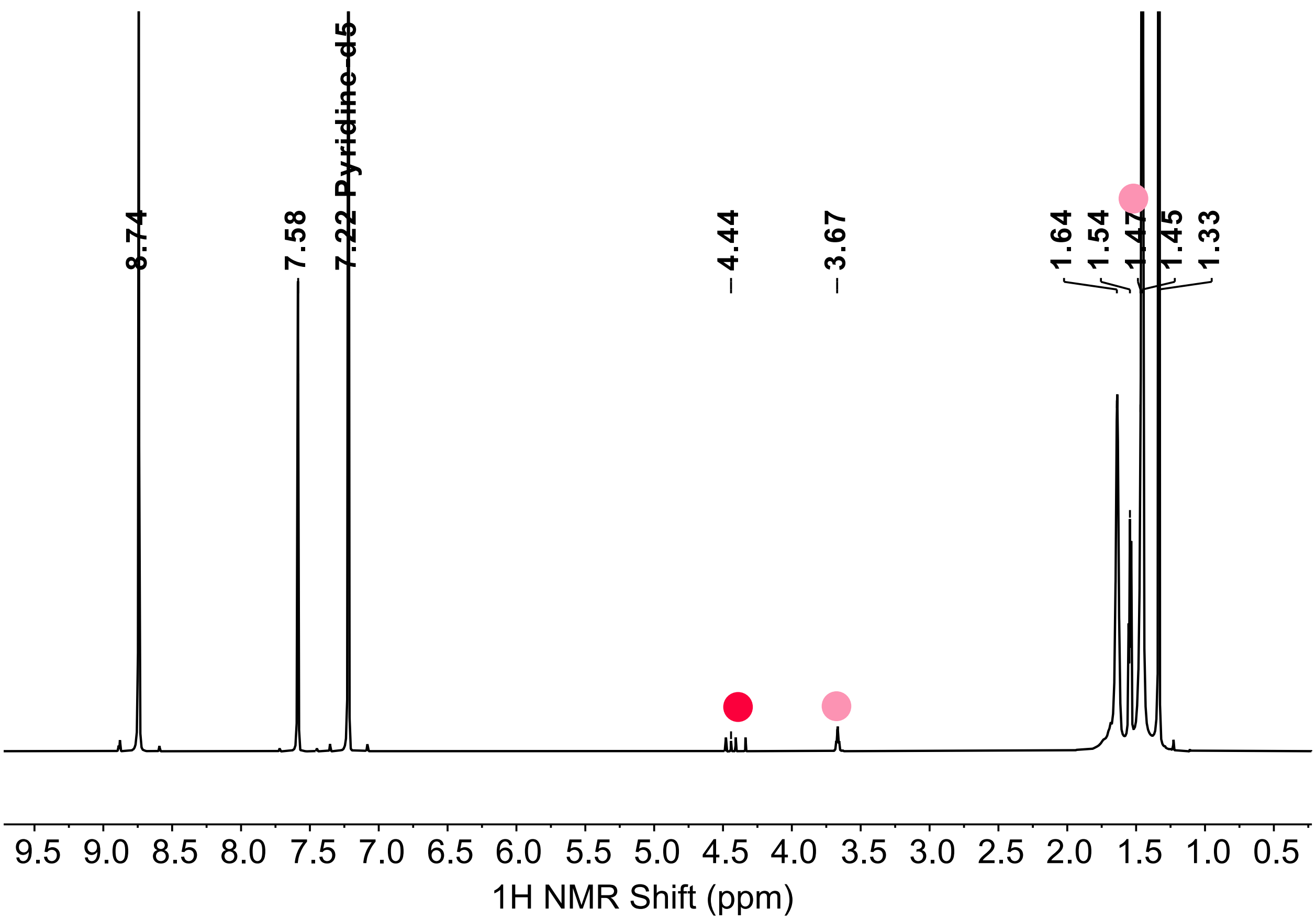

Figure S57. ^{1}H NMR (600 MHz, pyridine-d_5, 25 °C) spectrum of the reaction mixture obtained by treatment of **[Zn]** with D_2 (ca. 8 atm). The red dot indicates resonances assigned to H_2 and HD, the pink dots indicate resonances assigned to THF.

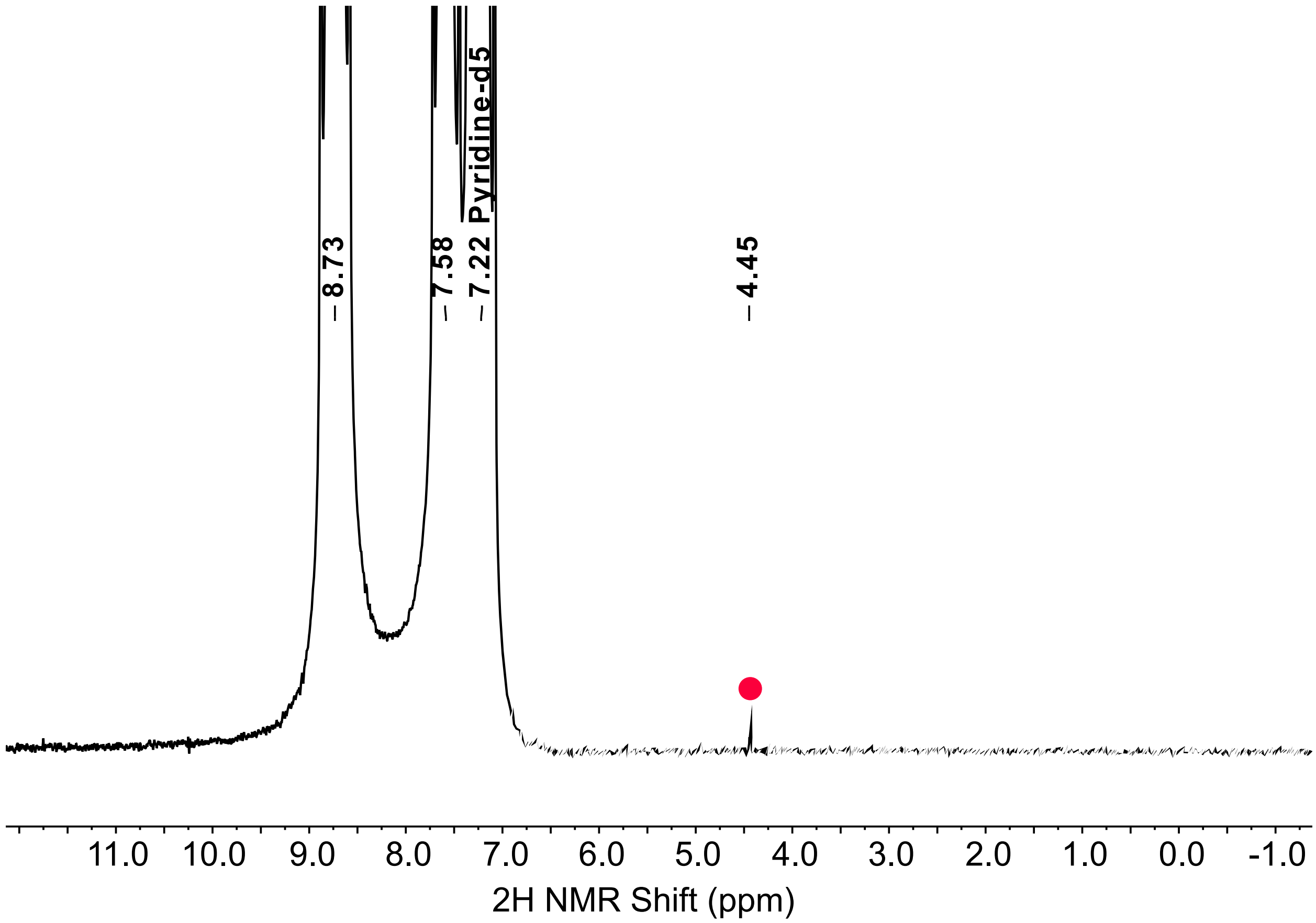

Figure S58. ^{2}H NMR (92 MHz, pyridine-d_5, 25 °C) spectrum of the reaction mixture obtained by treatment of **[Zn]** with D_2 (ca. 8 atm). The red dot indicates a resonance assigned to H_2.

6.3 Treatment of [Cd] with D_2

[Cd] (0.010 g, 0.023 mmol) was dissolved in benzene-d_6 (0.6 mL) and transferred to a J Young tube (2 mL). The solution was removed from the glovebox and degassed by three freeze-pump-thaw cycles, backfilled with D_2 (ca. 3 atm), and after 24 h, and NMR data was collected. The formation of black solids was observed 5 minutes after treatment with D_2. The ^{1}H NMR (600 MHz, benzene-d_6, Fig. S58) spectrum of the reaction mixture indicated formation of TEMPOD and no resonance corresponding to TEMPOH (O–H) was observed. The ^{2}H NMR (92 MHz, benzene-d_6, Fig. S59) spectrum of the sample revealed a new resonance at 3.76 ppm which we attributed to TEMPOD formation.

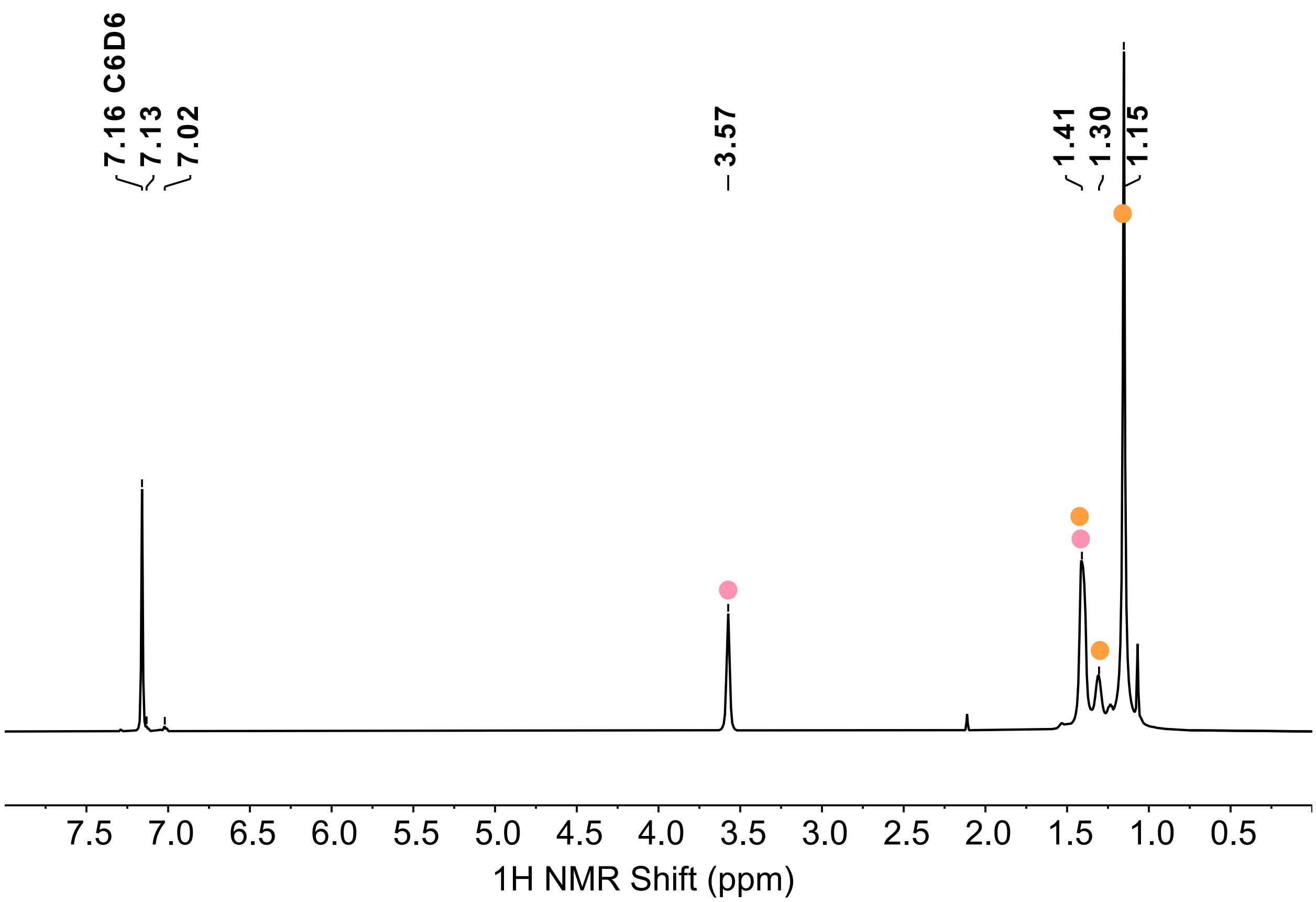

Figure S59. ^{1}H NMR (600 MHz, benzene-d_6, 25 °C) spectrum of the reaction mixture obtained by treatment of **[Cd]** with D_2 (ca. 3 atm). The orange dots indicate resonances assigned to TEMPOD, the pink dots indicate resonances assigned to THF.

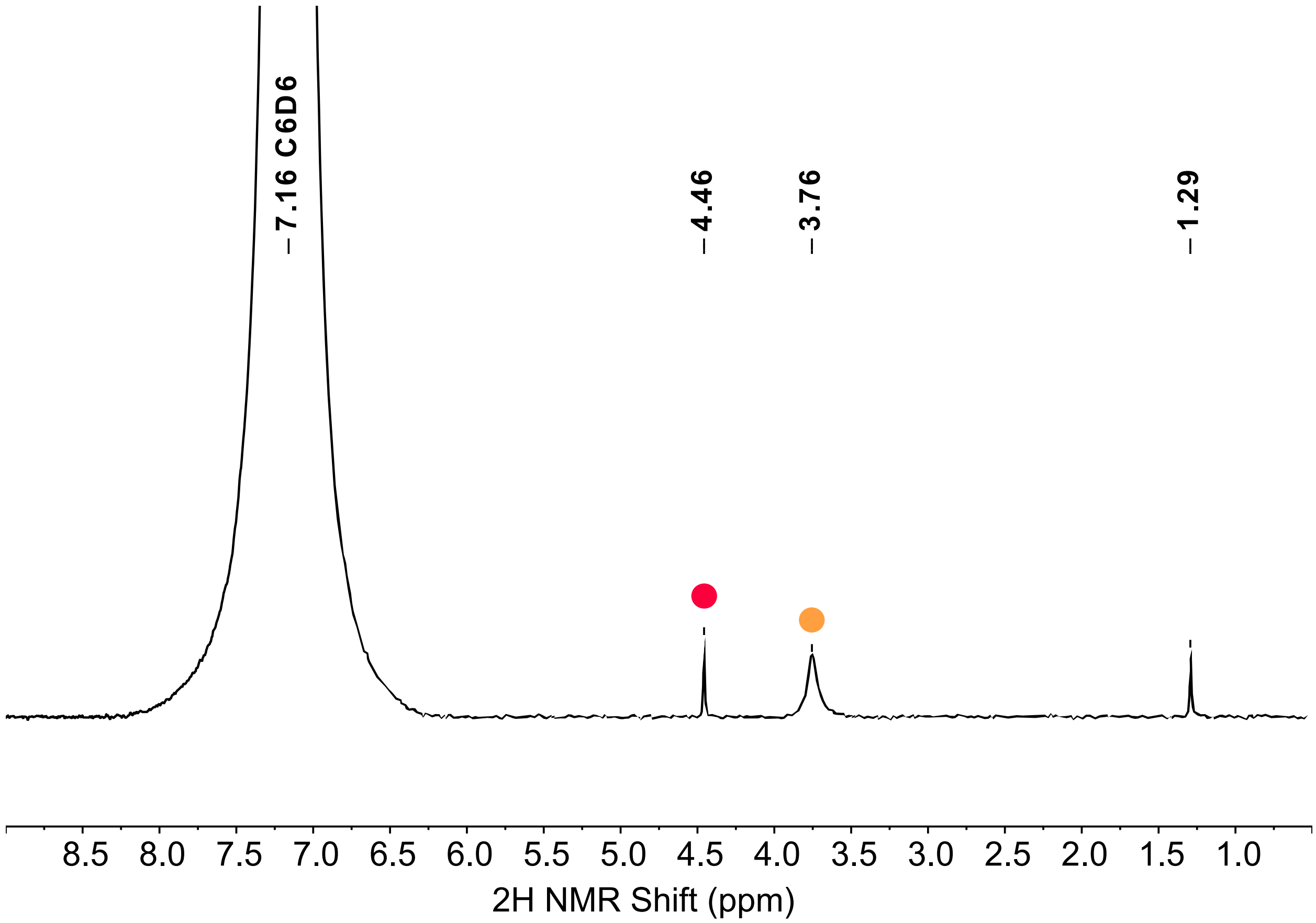

Figure S60. ^{2}H NMR (92 MHz, benzene-d_6, 25 °C) spectrum of the reaction mixture obtained by treatment of **[Cd]** with D_2 (ca. 3 atm). The red dot indicates a resonance assigned to D_2, the orange dot indicates a resonance attributed to TEMPOD, the resonance at 1.29 ppm is attributed to an impurity present in the NMR solvent.

6.4 Treatment of [Sn] with D_2

[Sn] (0.01 g, 0.020 mmol) was dissolved in benzene-d_6 (0.6 mL) and transferred to a J Young tube (2 mL). The solution was removed from the glovebox and degassed by three freeze-pump-thaw cycles, backfilled with D_2 (ca. 3 atm), and after 24 h, and NMR data was collected. The ^{1}H NMR (600 MHz, benzene-d_6, Fig. S60) spectrum of the reaction mixture showed that both **[Sn]** and TEMPOH were present. The ^{2}H NMR (92 MHz, benzene-d_6, Fig. S61) spectrum of the sample showed no TEMPOD was formed. We attributed the TEMPOH formation to deleterious reaction with trace oxygen or water

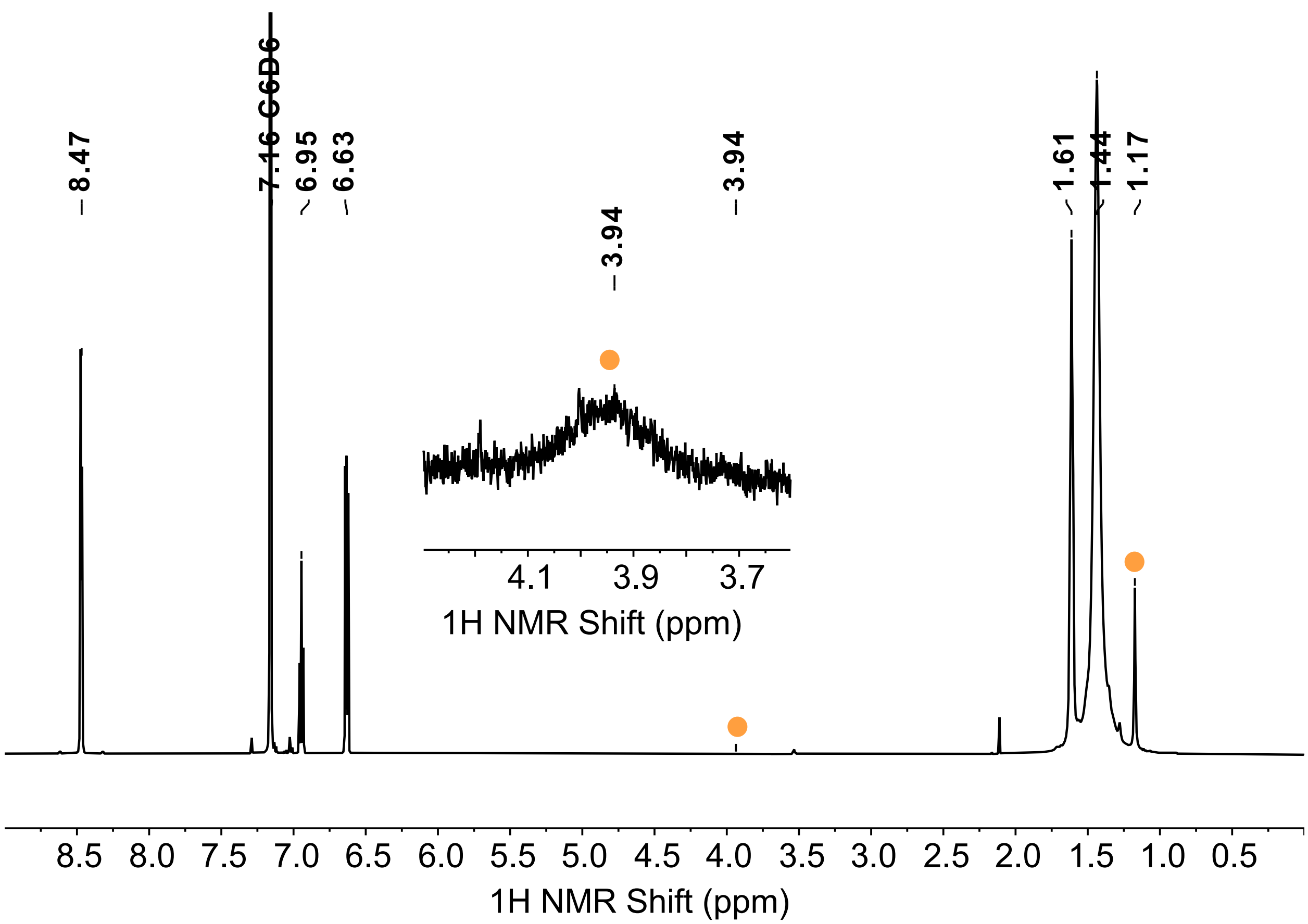

Figure S61. ^{1}H NMR (600 MHz, benzene-d_6, 25 °C) spectrum of the reaction mixture obtained by treatment of **[Sn]** with D_2 (ca. 3 atm). The orange dots indicate resonances assigned to TEMPOD.

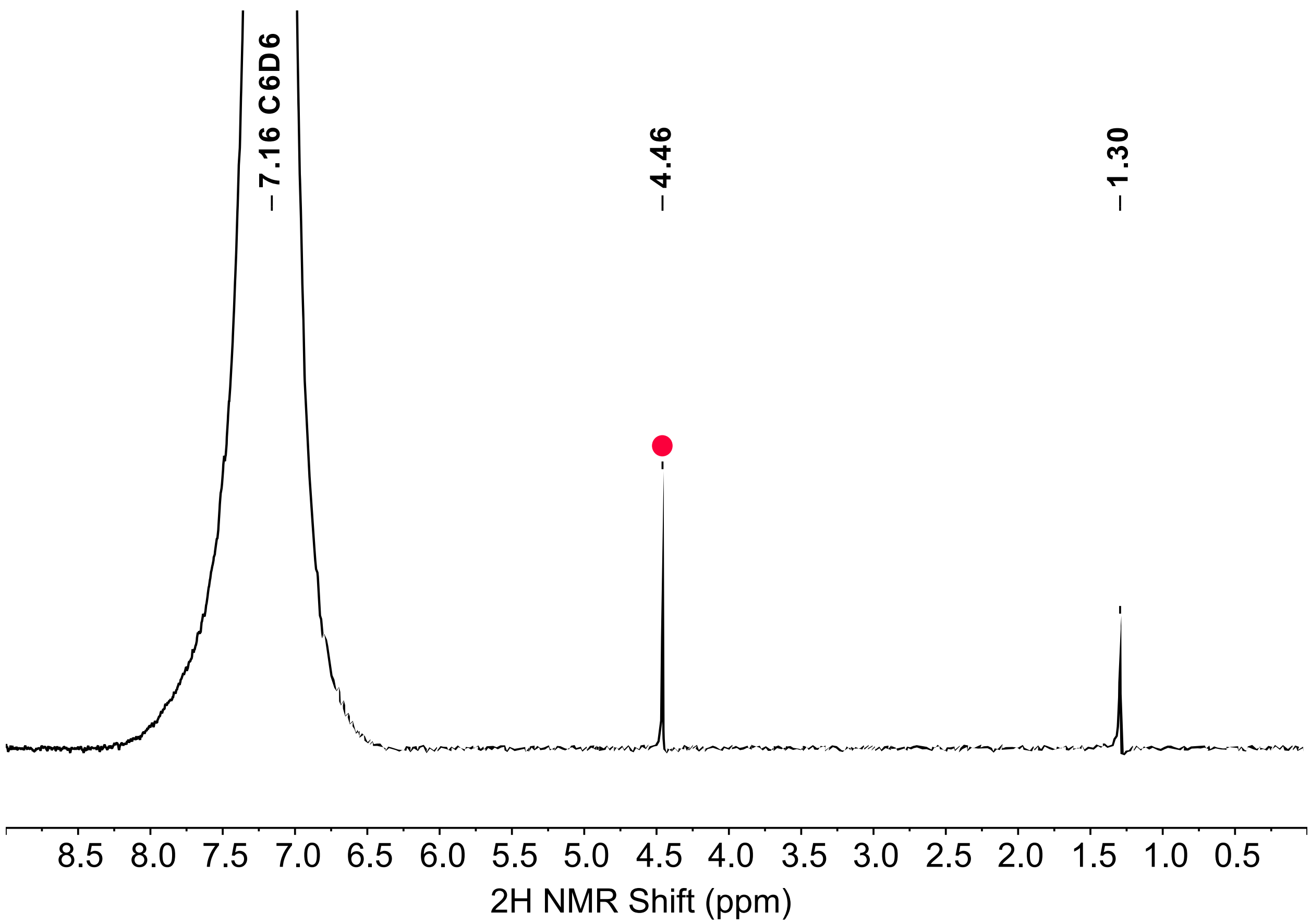

Figure S62. ^{2}H NMR (92 MHz, benzene-d_6, 25 °C) spectrum of the reaction mixture obtained by treatment of **[Sn]** with D_2 (ca. 3 atm). The red dot indicates a resonance assigned to D_2, the resonance at 1.30 ppm is attributed to an impurity present in the NMR solvent.

7. Gutmann-Beckett Studies

Separate solutions of the complexes (0.50 mL, 0.017 M) were combined with a stock solution of Et_3PO (0.10 mL, 0.033 M, 0.33 equiv) in NMR tubes and analyzed by $^{31}P\{^1H\}$ NMR spectroscopy. Sub stoichiometric amounts of Et_3PO were used to ensure more complete adduct formation.[9] A table of Gutmann-Beckett shifts is provided and shows the change in $^{31}P\{^1H\}$ NMR chemical shift of the Et_3PO adduct upon complexation. Notably, treatment of **[Cd]** with Et_3PO resulted in the rapid formation of black solids and the solution showed no resonances in the $^{31}P\{^1H\}$ NMR spectrum. Treatment of **[Zn]** with Et_3PO resulted in two resonances in the $^{31}P\{^1H\}$ NMR spectrum.

Table S10. Table of Gutmann-Beckett Shifts

Compound	Gutmann-Beckett shift, $\Delta\delta^{31}P\{^1H\}$ (ppm)
**[Mg]**	15.9
**[Ca·Na$_2$]**	5.3
**[Sr·Na]$_2$**	4.8
**[Zn]**	19.4 and 8.4
**[Cd]**	---
**[Sn]**	5.3

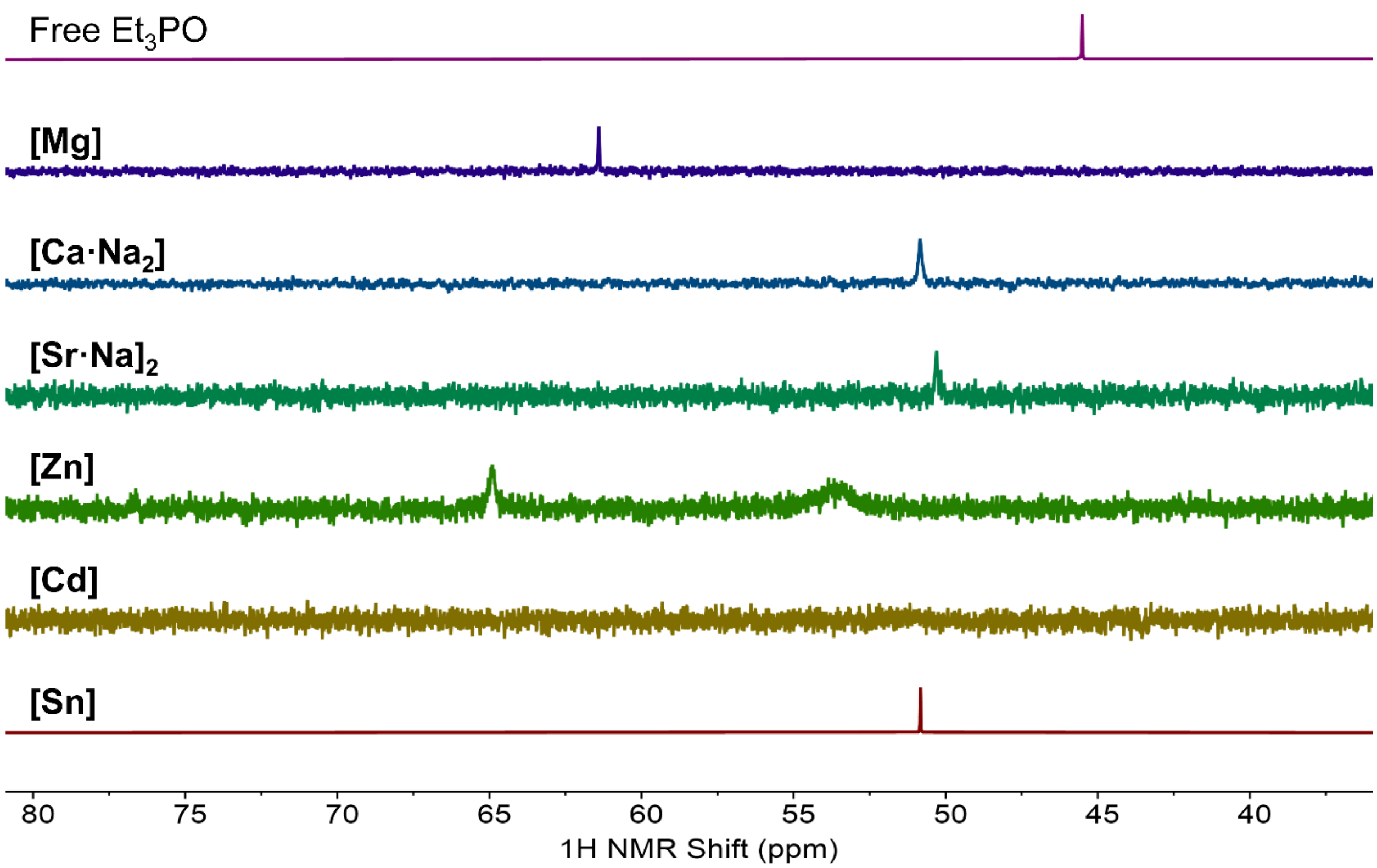

Figure S63. Stacked $^{31}P\{^1H\}$ NMR (162 MHz, benzene-d_6, 25 °C) spectra of Gutmann-Beckett experiments.

8. Reactivity with Ph_3PSe

8.1 Treatment of [Cd] with Ph_3PSe, followed by H_2

[Cd] (0.010 g, 0.020 mmol, 1.00 equiv.) was combined with Ph_3PSe (0.007 g, 0.02 mmol, 1.00 equiv.) in benzene-d_6 (0.6 mL) and transferred to a J Young tube (2 mL). The solution was removed from the glovebox and degassed by three freeze-pump-thaw cycles. The J Young tube was then backfilled with H_2 (ca. 3 atm) and set for 48 h, during which time the formation of yellow solids was observed. The ^1H NMR (400 MHz, benzene-d_6, Fig. S63) spectrum of the reaction mixture indicated formation of TEMPOH. The $^{31}P\{^1H\}$ NMR (162 MHz, benzene-d_6, Fig. S64) spectrum of the reaction mixture showed signals corresponding to Ph_3PSe and PPh_3 in a 1:10 molar ratio. The yellow powder that formed during the reaction was separated by centrifugation, washed with toluene (2 x 2 mL), and all volatiles were removed under dynamic vacuum. Analysis of the powder by PXRD (Fig. S65) in the 20-50° 2θ range revealed a broad featureless pattern, indicating formation of an amorphous material. EDS analysis (Table S11) indicated the presence of Cd and Se in an approximately 1:1 molar ratio. Raman analysis (Fig. S66) of the powder revealed two bands present at 178 and 250 cm^{-1} indicating the formation of CdSe. The yellow powder was annealed using an MTI OTF 1200 tube furnace at 400 °C for 4 hours under argon environment, leading to the formation of a black powder. Analysis of the annealed powder by PXRD (Fig. S65) in the 20-50° 2θ range revealed a pattern corresponding to hexagonal CdSe.[10] The annealed solids exhibited two bands at 173 and 250 cm^{-1} Raman data (Fig. S66).

8.2 Treatment of Cd Powder with Ph_3PSe, followed by H_2

Cadmium powder was prepared via hydrogenolysis of **[Cd]**, cadmium powder was collected on filter paper, washed with THF (10 mL) and hexane (10 mL), and was dried under dynamic vacuum. In the glovebox, cadmium powder (0.015 g, 0.133 mmol, 1.00 equiv.) was placed in a J Young NMR tube containing a solution of Ph_3PSe (0.045 g, 0.133 mmol, 1.00 equiv.) in benzene-d_6 (0.6 mL). The sample was removed from the glovebox and degassed by three freeze-pump-thaw cycles. After being degassed, the J Young NMR tube was backfilled with H_2 (ca. 3 atm). The ^1H NMR (400 MHz, benzene-d_6, Fig. S63) spectrum of the reaction mixture after 24 h showed resonances corresponding to Ph_3PSe and H_2. The $^{31}P\{^1H\}$ NMR (162 MHz, benzene-d_6, Fig. S64) spectrum of the reaction mixture after 24 h showed a resonance corresponding to Ph_3PSe.

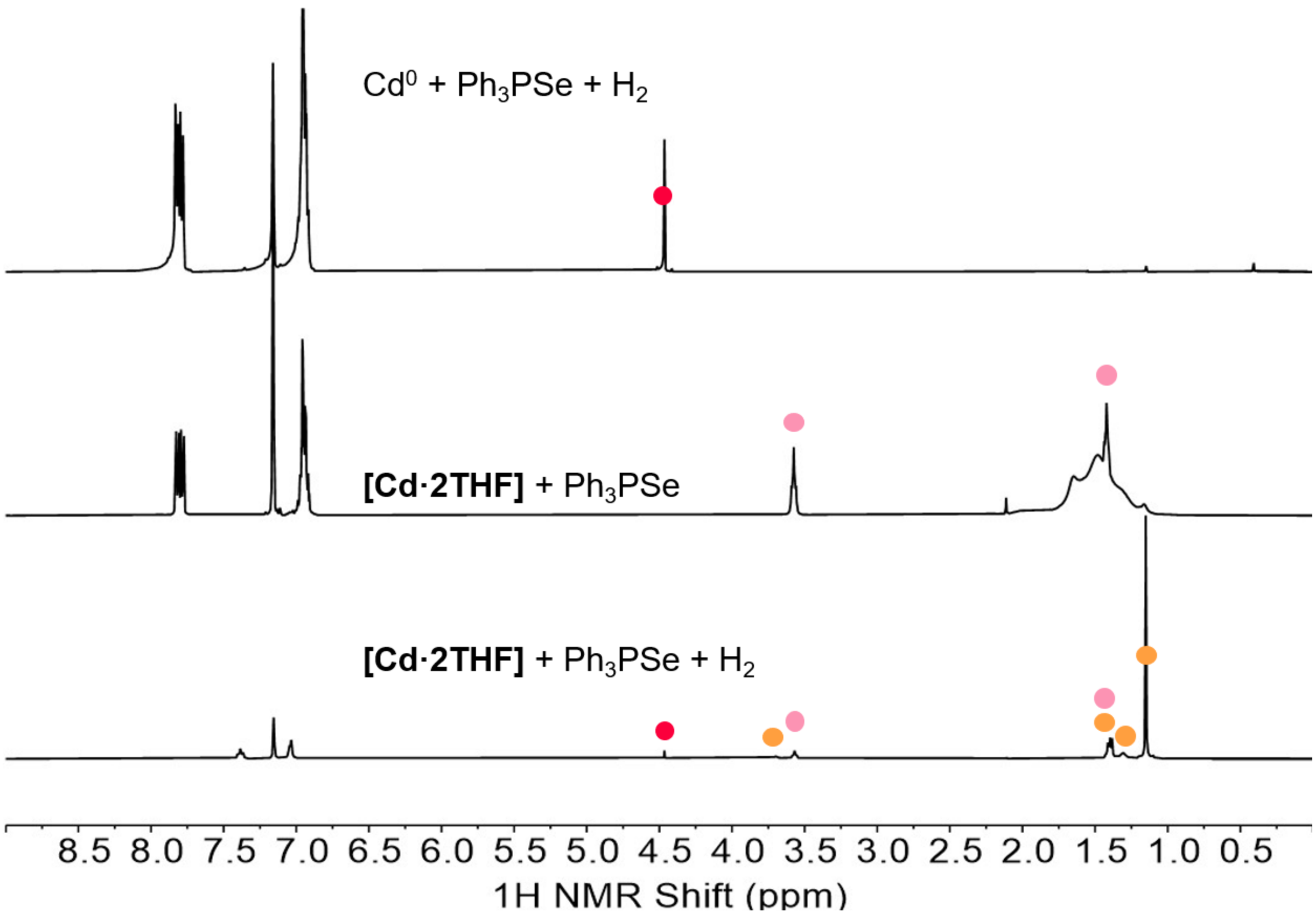

Figure S64. ^{1}H NMR (400 MHz, benzene-d_6, 25 °C) spectrum of the reaction mixture obtained by treatment of **[Cd]** with Ph_3PSe and H_2 (ca. 3 atm) (bottom spectrum), the reaction mixture obtained by treatment of **[Cd]** with Ph_3PSe (middle spectrum), and the reaction mixture obtained by treatment of Cd^0 with Ph_3PSe and H_2 (top spectrum). The orange dots indicate resonances assigned to TEMPOH, the pink dots indicate resonances assigned to THF, the red dots indicate resonances assigned to H_2.

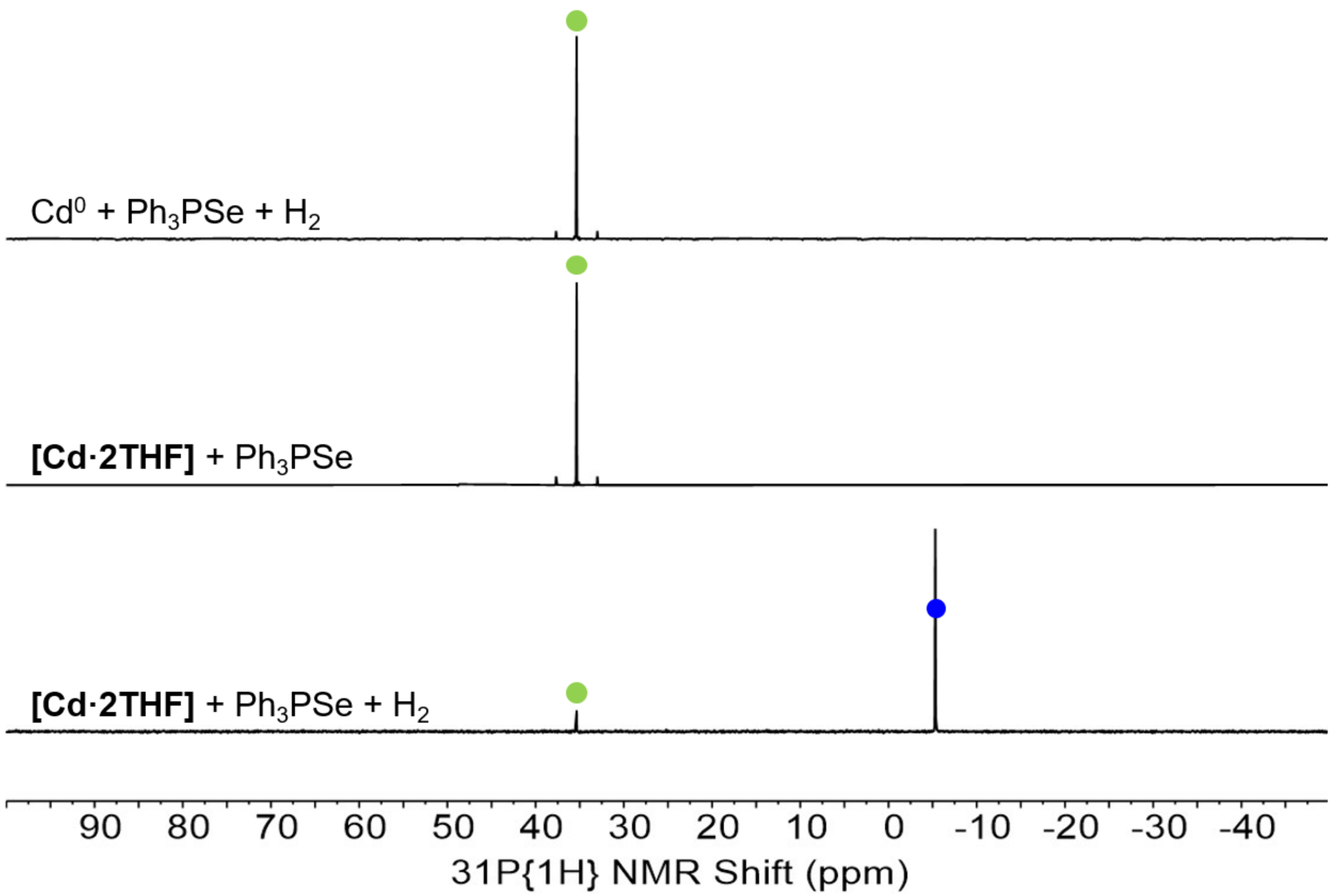

Figure S65. Stacked $^{31}P\{^1H\}$ NMR (162 MHz, benzene-d_6, 25 °C) spectrum of the reaction mixture obtained by treatment of **[Cd]** with Ph_3PSe and H_2 (ca. 3 atm) (bottom spectrum), the reaction mixture obtained by treatment of **[Cd]** with Ph_3PSe (middle spectrum), and the reaction mixture obtained by treatment of Cd^0 with Ph_3PSe and H_2 (top spectrum). The green dots indicate resonances assigned to Ph_3PSe, the blue dot indicates a resonance assigned to PPh_3.

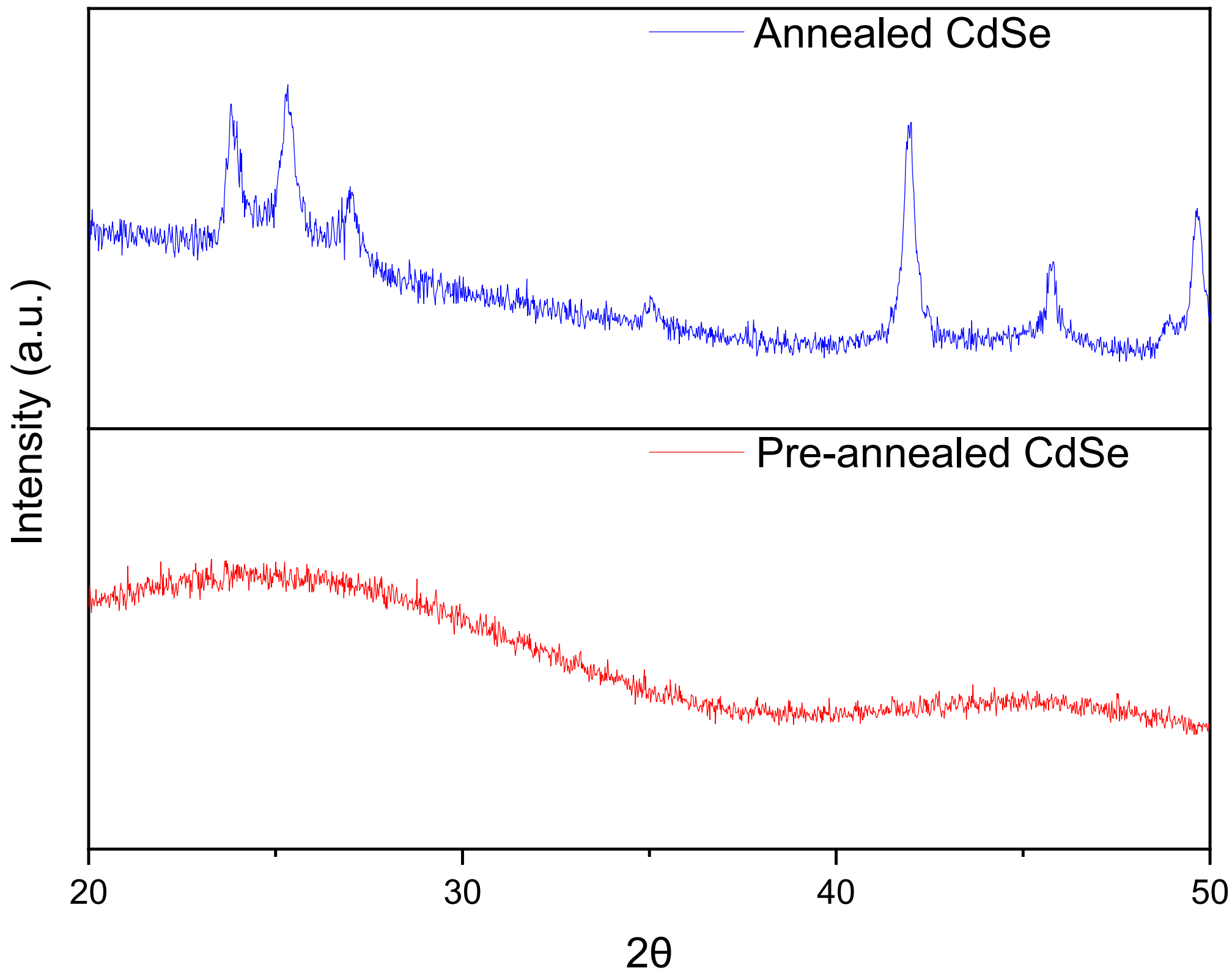

Figure S66. PXRD pattern of the solids obtained from the treatment of **[Cd]** with Ph_3PSe and H_2 (ca. 3 atm) before annealing (bottom, red trace), and after annealing (top, blue trace) (0.5° slit, 0.017° step size, and 65 s/step).

Table S11. EDS data of the treatment of **[Cd]** with Ph_3PSe and H_2 (3 atm)

**Element Line**	**Net Counts**	**Source**	**Weight %**	**Weight % Error**	**Atom %**	**Atom % Error**	**Compound %**
**C K**	28682	EDS	14.26	± 0.98	54.19	± 3.71	14.26
**O K**	0	EDS	0.00	---	0.00	± 0.00	0.00
**P K**	23497	EDS	3.51	± 0.07	5.17	± 0.11	3.51
**Se L**	285289	EDS	37.46	± 0.16	21.66	± 0.09	37.46
**Cd L**	119534	EDS	43.87	± 0.47	17.82	± 0.19	43.87

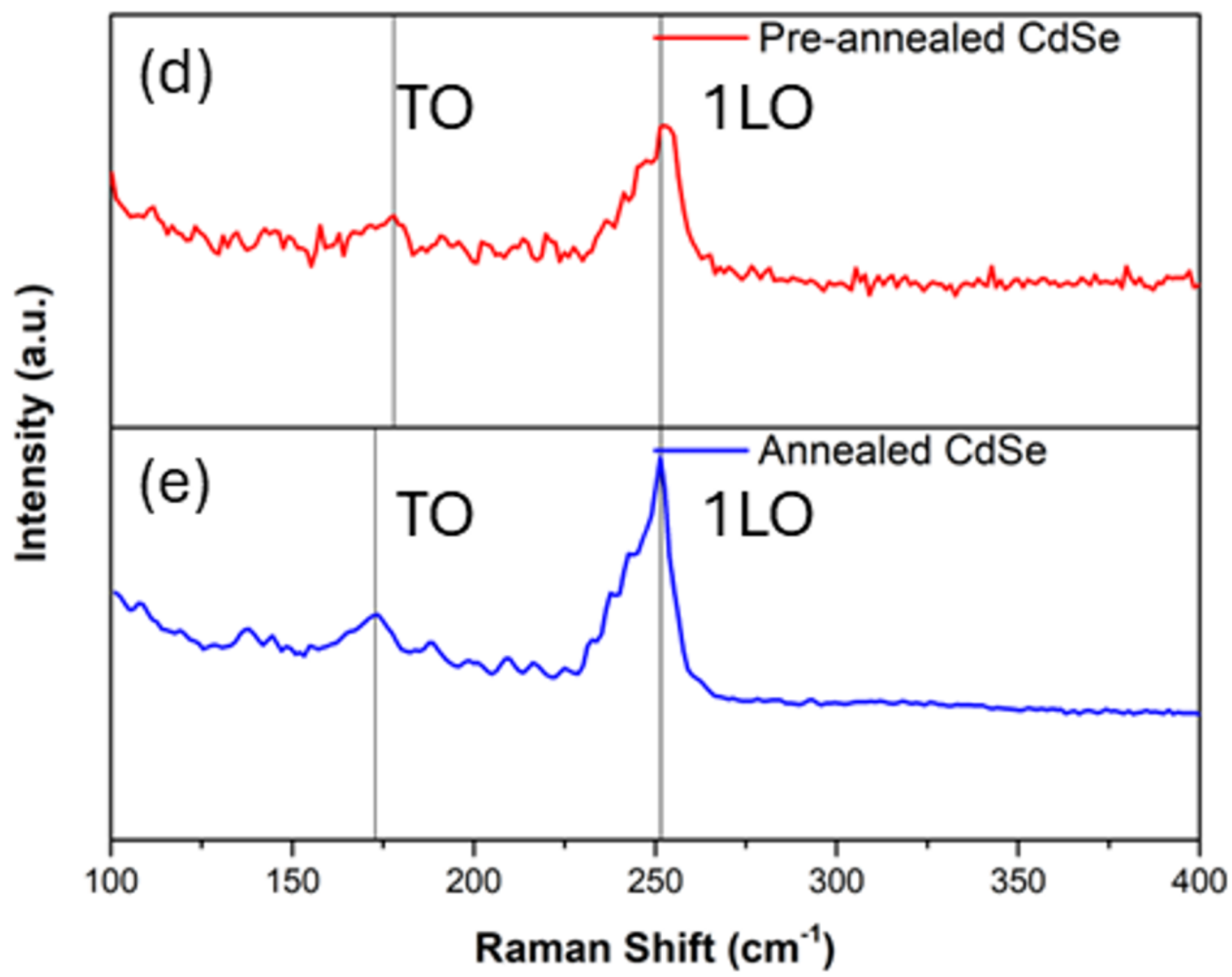

Figure S67. Raman spectrum of the solids obtained from the treatment of **[Cd]** with Ph_3PSe and H_2 (ca. 3 atm) before annealing (top, red trace), and after annealing (bottom, blue trace). The gray line indicates the position of the peaks.

8.3 Treatment of NaTEMPO and $PbCl_2$ and Ph_3PSe, followed by H_2

A cold (−32 °C) solution of Ph_3PSe (0.245 g, 0.719 mmol, 1.00 equiv.) in pyridine (20 mL) was transferred to a cold (−32 °C) Schlenk storage flask (50 mL) containing a stir bar, $PbCl_2$ (0.200 g, 0.719 mmol, 1.00 equiv.), and NaTEMPO (0.258 g, 1.44 mmol, 2.00 equiv.). The flask was brought out of the glovebox, degassed by three freeze-pump thaw cycles and allowed to warm to room temperature. After reaching room temperature the Schlenk flask was charged with H_2 (ca. 3 atm). The reaction mixture was then kept at 0 °C for 30 minutes before being allowed to stir at room temperature for 24 hours. After 24 hours, the black solids that had formed were separated by centrifugation, washed with toluene (2 × 2 mL), and all volatiles were removed under dynamic vacuum. An aliquot of the supernatant was analyzed using NMR spectroscopy. The ^{1}H NMR (400 MHz, benzene-d_6, Fig. S67) spectrum of the reaction mixture indicated formation of TEMPOH. The $^{31}P\{^{1}H\}$ NMR (162 MHz, benzene-d_6, Fig. S68) spectrum of the reaction mixture showed signals corresponding to Ph_3PSe and PPh_3 in a 1:2 molar ratio. PXRD analysis (Fig. S69) of the solids in the 20-70° 2θ range revealed a powder pattern mixture corresponding to NaCl, Pb^0, and PbSe.[5,6,11]

8.4 Treatment of Pb Powder with Ph_3PSe, followed by H_2

Lead powder was prepared via hydrogenolysis of *in situ* generated **[Pb]**, solids were collected on filter paper, washed with THF (10 mL) and hexane (10 mL), and brought out of the glovebox and washed with water (10 mL). The lead powder was then dried under a dynamic vacuum and brought back into the glovebox. In the glovebox, lead powder (0.029 g, 0.139 mmol, 1.00 equiv.) was placed in a J Young NMR tube containing a solution of Ph_3PSe (0.047 g, 0.139 mmol, 1.00 equiv.) in benzene-d_6 (0.6 mL). The sample was removed from the glovebox and degassed by three freeze-pump-thaw cycles. After being degassed, the J Young NMR tube was backfilled with H_2 (ca. 3 atm). The ^{1}H NMR (400 MHz, benzene-d_6, Fig. S67) spectrum of the reaction mixture after 24 h showed resonances corresponding to Ph_3PSe and H_2. The $^{31}P\{^{1}H\}$ NMR (162 MHz, benzene-d_6, Fig. S68) spectrum of the reaction mixture after 24 h showed a resonance corresponding to Ph_3PSe.

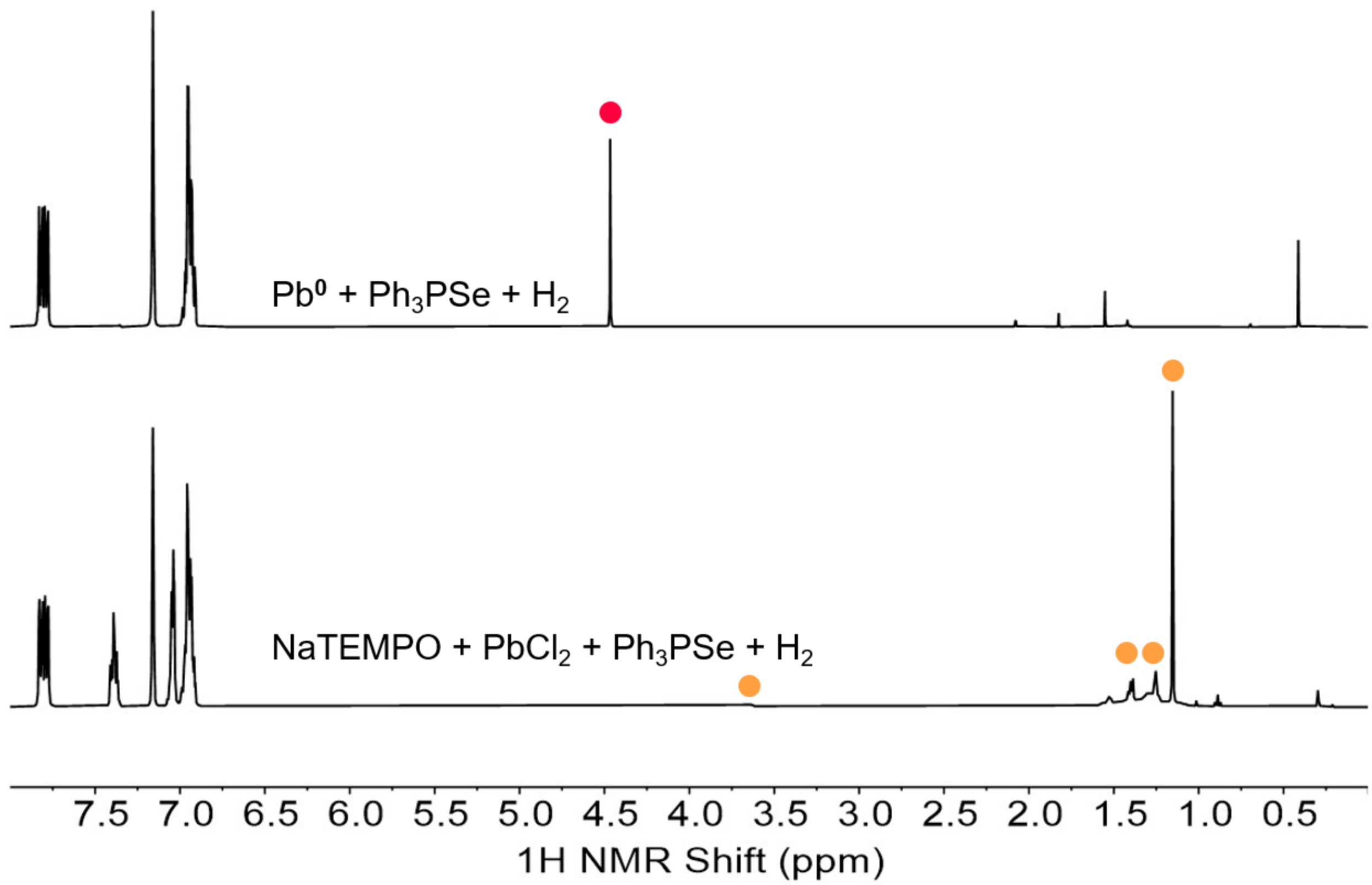

Figure S68. ^{1}H NMR (400 MHz, benzene-d_6, 25 °C) spectrum of the treatment of NaTEMPO with $PbCl_2$, Ph_3PSe, and H_2 (ca. 3 atm) (bottom trace) and control treatment of Pb^0 with Ph_3PSe and H_2 (3 atm) (top trace). The orange dots indicate resonances assigned to TEMPOH, the red dot indicates a resonance assigned to H_2.

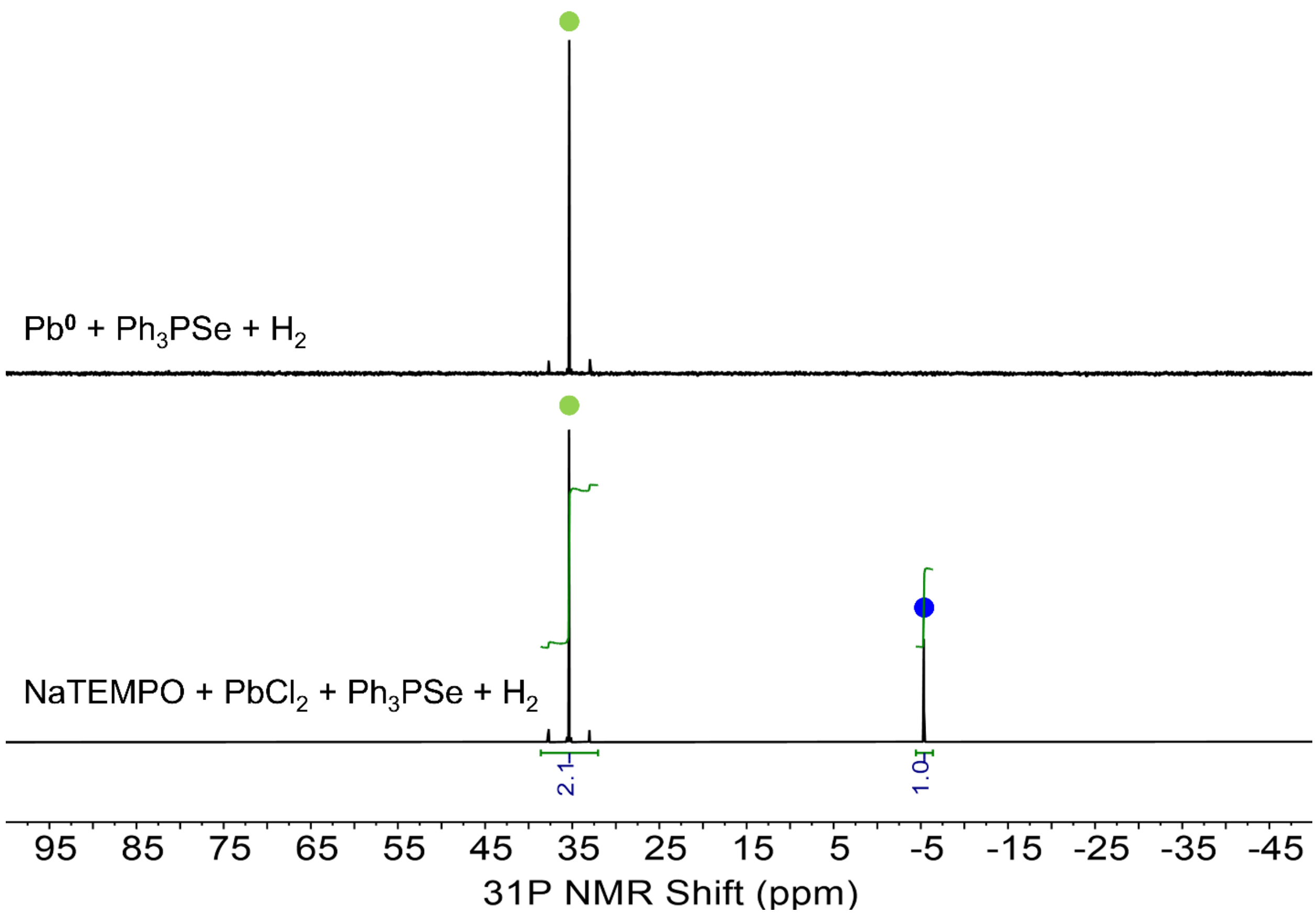

Figure S69. Stacked $^{31}P\{^{1}H\}$ NMR (162 MHz, benzene-d_6, 25 °C) spectra of the treatment of NaTEMPO with $PbCl_2$, Ph_3PSe, and H_2 (3 atm) (bottom trace) and control treatment of Pb^0 with Ph_3PSe and H_2 (ca. 3 atm) (top trace). The green dots indicate resonances assigned to Ph_3PSe, the blue dot indicates a resonance assigned to PPh_3.

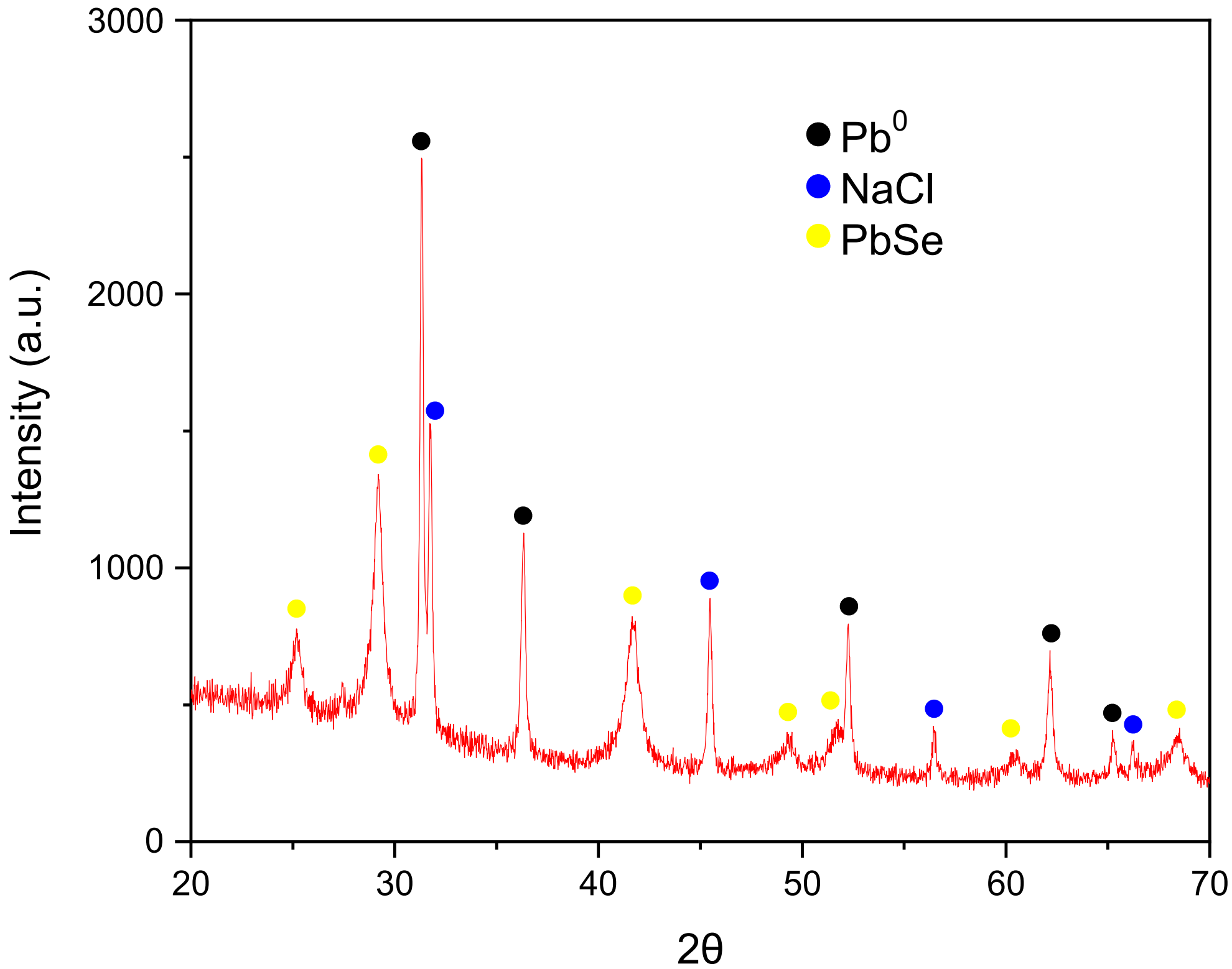

Figure S70. PXRD pattern of the solids obtained from the treatment of NaTEMPO with $PbCl_2$, Ph_3PSe, and H_2 (ca. 3 atm) (0.5° slit, 0.017° step size, and 50 s/step).

9. X-ray Diffraction Structures and Refinement Details

Data for **[Mg]**, **[Ca·Na$_2$]**, **[Sr·Na]$_2$**, **[Sn]**, **[Cd]**, and **[Pb]** were collected from shock-cooled single crystals at 100(2) K on a Bruker D8 three-axis goniometer with a sealed X-ray tube using a Triumph as monochromator and a Bruker Apex-II CCD detector. The diffractometer was equipped with an Oxford Cryostream 700 low temperature device and used CuK_α radiation (λ = 1.54178 Å) or MoK_α radiation (λ = 0.71073 Å). All data were integrated with SAINT V8.41 and a multi-scan absorption correction was applied using SADABS 2016/2 or TWINABS for twinned structures.[12,13] The structure was solved by dual space methods with SHELXT and refined by full-matrix least-squares methods against F^2 using SHELXL-2019/2 following established refinement strategies.[14,15] All non-hydrogen atoms were refined with anisotropic displacement parameters. All hydrogen atoms were refined with isotropic displacement parameters. Some of their coordinates were refined freely and some on calculated positions using a riding model with their U_{iso} values constrained to 1.5 times the U_{eq} of their pivot atoms for terminal sp^3 carbon atoms and 1.2 times for all other carbon atoms. Details of the data quality and a summary of the residual values of the refinement are listed in Tables S12-S17. Further details can be found in the form of .CIF available from the Cambridge Crystallographic Data Centre.[16] Parts of this report and the .CIF were generated using FinalCif.[17]

A colorless, block-shaped crystal of **[Mg]** was grown from a vapor diffusion setup using THF as the solvent and toluene as the antisolvent (Fig. S70). The crystal was twinned; therefore, the orientation matrices for two components were determined using CELL NOW.[18] The molecule exhibits no disorder. The structure of **[Mg(TEMPO)$_2$]$_2$** has been previously reported crystallized in the $P2_12_12_1$ space group and including benzene in the unit cell.[19] A colorless, block-shaped crystal of **[Ca·Na$_2$]** was grown from a vapor diffusion setup using THF as the solvent and toluene as the antisolvent (Fig. S71). The molecule exhibits no disorder. A colorless, block-shaped crystal of **[Sr·Na]$_2$** was grown from a vapor diffusion setup using THF as the solvent and toluene as the antisolvent (Fig. S72). The compound crystallizes as 2 molecules within the unit cell. One THF on each molecule exhibits disorder, requiring refinement in negative parts and strict SIMU and SADI restraints. Many reflections were omitted due to poor handling of spot overlap. A colorless, block-shaped crystal of **[Sn]** was grown from a saturated solution in hexanes cooled to −38 ˚C (Fig. S73). The crystal was twinned; therefore, the orientation matrices for two components were determined using CELL NOW.[18] The molecule exhibits no disorder. A yellow, block-shaped crystal of **[Cd]** was grown from a saturated solution in hexanes cooled to −38 ˚C (Fig. S74). The crystal was twinned; therefore, the orientation matrices for two components were determined using CELL NOW.[18] The molecule is disordered over a special position and the THF ligands are disordered, requiring refinement in negative parts. Strict SIMU restraints were applied and a global RIGU was applied. To refine heavily disordered electron density which has been treated as a diffuse contribution to the overall scattering without specific atom positions, the SQUEEZE procedure in PLATON was used.[20] A colorless, block-shaped crystal of **[Pb]** was grown from a saturated solution in hexanes cooled to −38 ˚C (Fig. S75). The molecule exhibits no disorder.

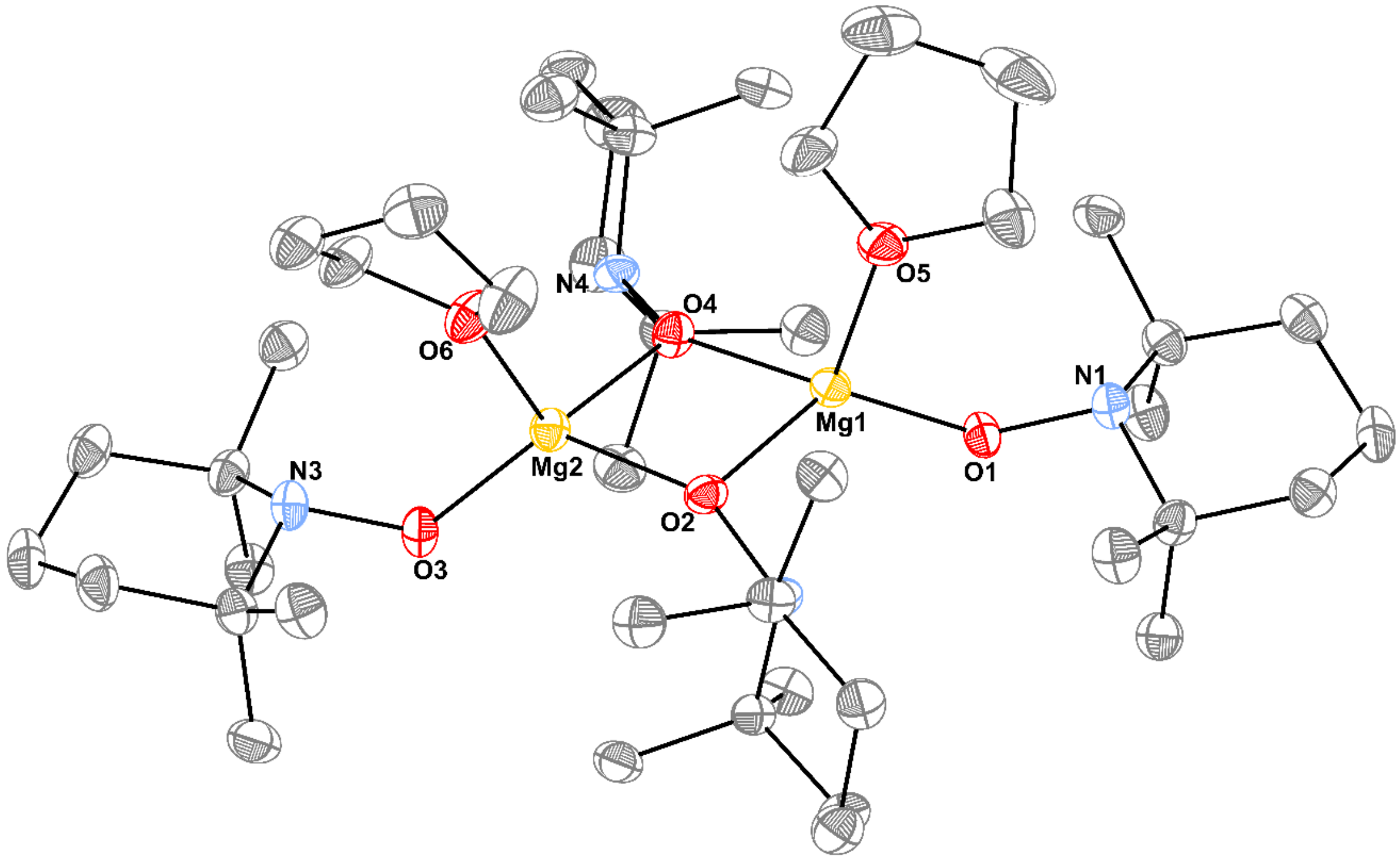

Figure S71. Molecular structure of **[Mg]** with thermal ellipsoids shown at the 50% probability level. Hydrogen atoms are omitted for clarity.

Table S12. Crystal data and structure refinement for **[Mg]**

CCDC	CSD 2514574	
Empirical formula	$C_{14.67}$ $H_{29.33}$ $Mg_{0.67}$ $N_{1.33}$ O_2	
Formula weight	272.60	
Temperature	100(2) K	
Wavelength	1.54178 Å	
Crystal system	Monoclinic	
Space group	P21	
Unit cell dimensions	a = 14.1999(7) Å	**α**= 90°.
	b = 18.9154(9) Å	**β**= 90.708(2)°.
	c = 17.8063(8) Å	**γ** = 90°.
Volume	4782.3(4) $Å^3$	
Z	2	
Density (calculated)	1.136 g/cm^3	
F(000)	1808	
Crystal size	0.599 x 0.409 x 0.344 mm^3	
Theta range for data collection	3.112 to 72.441°.	
Index ranges	-17<=h<=17, 0<=k<=23, 0<=l<=22	
Reflections collected	10163	
Independent reflections	10163 [*R*(int) = 0.0755]	
Completeness to theta = 67.679°	99.8 %	
Absorption correction	Semi-empirical from equivalents	
Max. and min. transmission	0.753578 and 0.635182	
Refinement method	Full-matrix least-squares on F^2	
Data / restraints / parameters	10163 / 1 / 1042	
Goodness-of-fit on F^2	1.177	
Final R indices [I>2sigma(I)]	R_1 = 0.0526, wR_2 = 0.1467	

R indices (all data)	R_1 = 0.0567, wR_2 = 0.1489
Largest diff. peak and hole	0.499 and -0.382 e.Å^{-3}

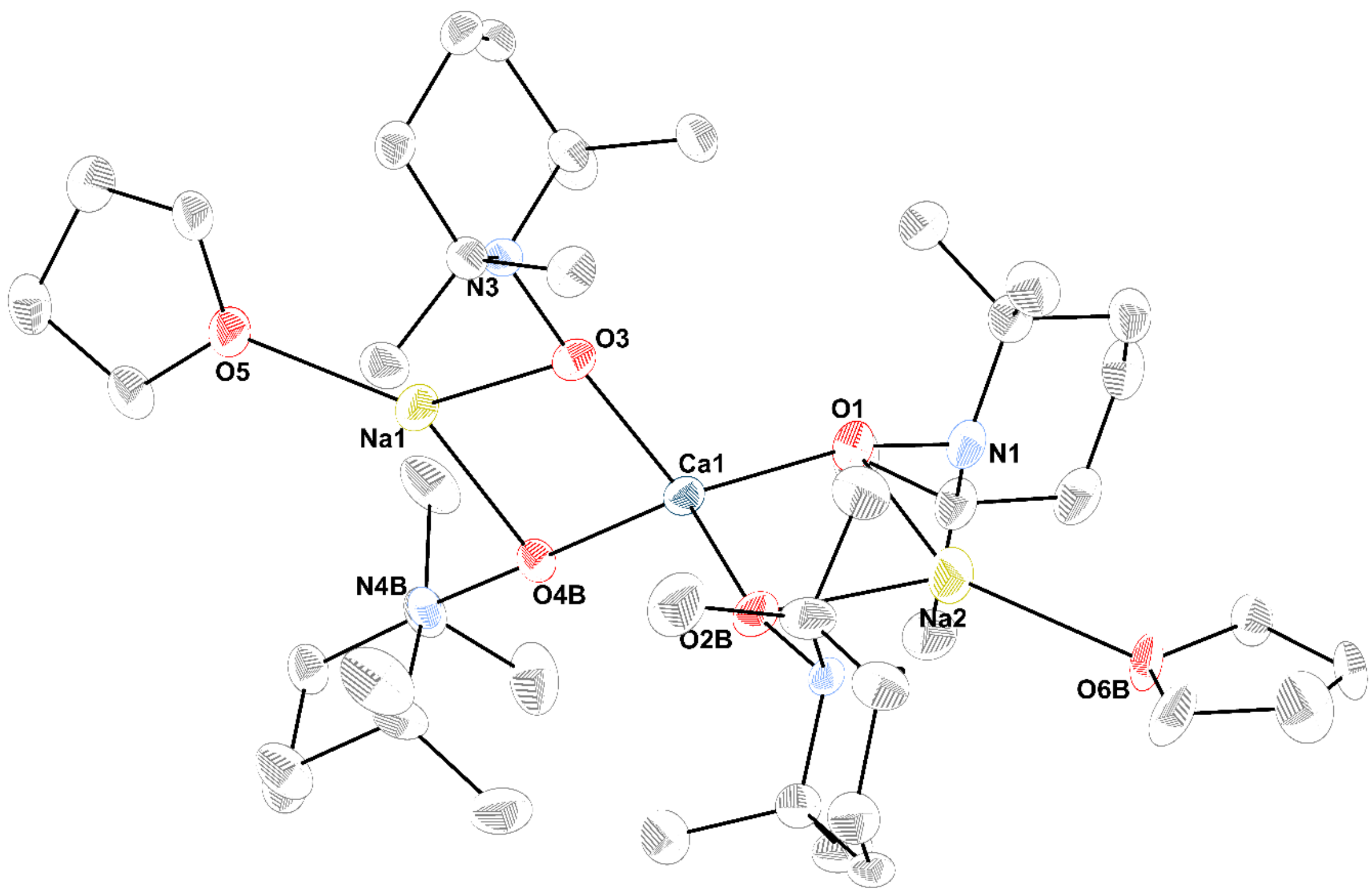

Figure S72. Solid-state structure of **[Ca·Na$_2$]** with thermal ellipsoids represented at the 50% probability level. Hydrogen atoms omitted for clarity.

Table S13. Crystal data and structure refinement for **[Ca·Na$_2$]**

CCDC	CSD 2514633	
Empirical formula	$C_{13.04} H_{26.07} Ca_{0.30} N_{1.19} Na_{0.59} O_{1.78}$	
Formula weight	253.39	
Temperature	100(2) K	
Wavelength	1.54178 Å	
Crystal system	Orthorhombic	
Space group	Pbca	
Unit cell dimensions	a = 13.6683(5) Å	**α**= 90°.
	b = 24.6605(8) Å	**β**= 90°.
	c = 29.8174(11) Å	**γ** = 90°.
Volume	10050.5(6) Å^3	
Z	8	
Density (calculated)	1.130 g/cm^3	
F(000)	3760	
Crystal size	0.424 x 0.390 x 0.208 mm^3	
Theta range for data collection	2.964 to 65.131°.	
Index ranges	-16<=h<=15, -28<=k<=29, -35<=l<=34	
Reflections collected	65343	
Independent reflections	8567 [R(int) = 0.0530]	
Completeness to theta = 67.679°	99.8 %	
Absorption correction	Semi-empirical from equivalents	
Max. and min. transmission	0.7526 and 0.6170	
Refinement method	Full-matrix least-squares on F^2	
Data / restraints / parameters	8567 / 1595 / 831	
Goodness-of-fit on F^2	1.173	
Final R indices [I>2sigma(I)]	R_1 = 0.0546, wR_2 = 0.1082	

R indices (all data)	R_1 = 0.0610, wR_2 = 0.1104
Largest diff. peak and hole	0.499 and -0.382 e.Å^{-3}

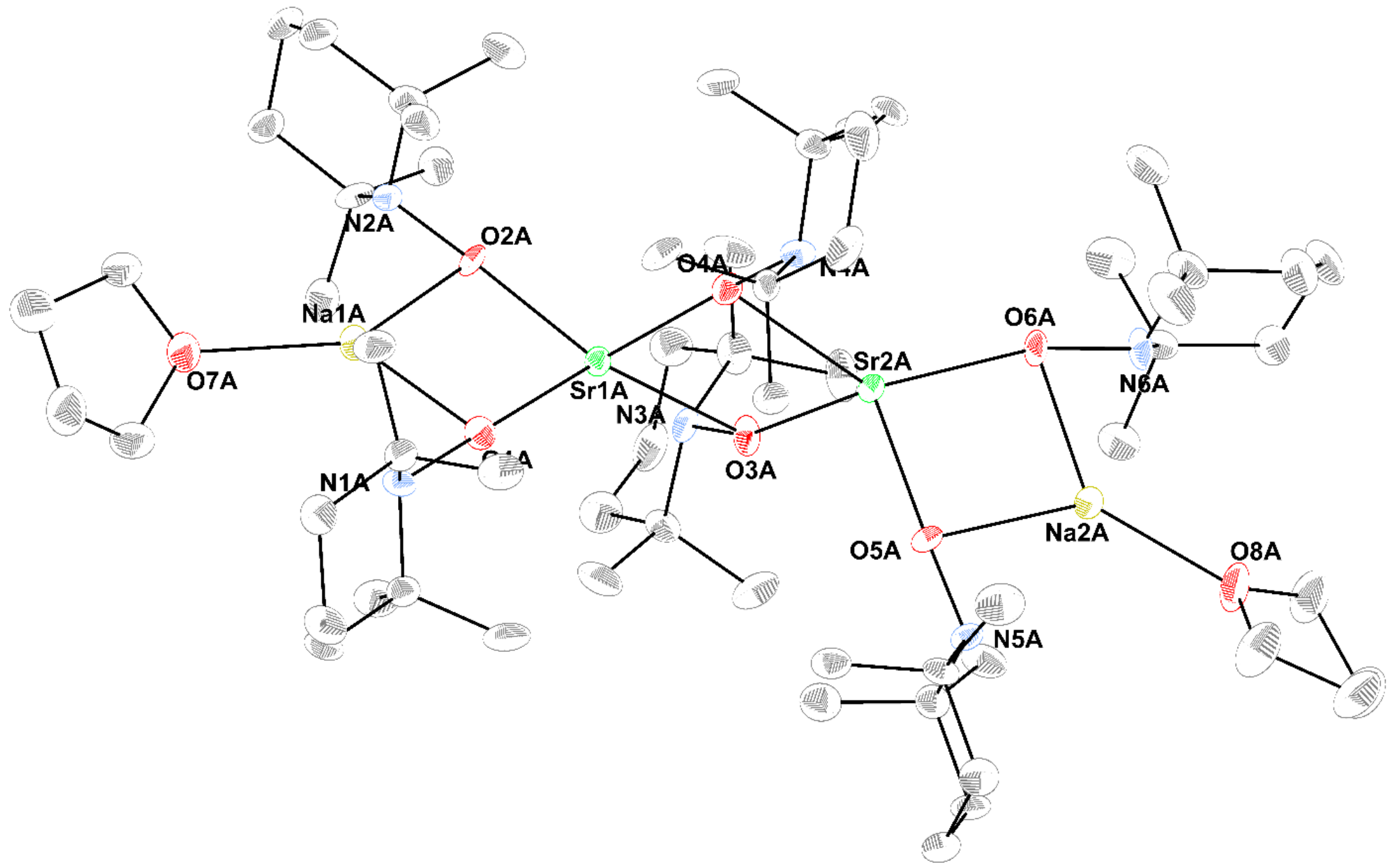

Figure S73. Solid-state structure of **[Sr·Na]$_2$** with thermal ellipsoids represented at the 50% probability level. Hydrogen atoms omitted for clarity.

Table S14. Crystal data and structure refinement for **[Sr·Na]$_2$**

CCDC	CSD 2542829	
Empirical formula	$C_{62}H_{124}N_6Na_2O_8Sr_2$	
Formula weight	1302.88	
Temperature	100(2) K	
Wavelength	0.71073 Å	
Crystal system	triclinic	
Space group	$P\underline{1}$ (2)	
Unit cell dimensions	a = 12.2715(11) Å	**α**= 108.028(2)°.
	b = 22.219(2) Å	**β**= 91.108(2)°.
	c = 28.034(3) Å	**γ** = 103.916(2)°.
Volume	7018.6(11) $Å^3$	
Z	4	
Density (calculated)	1.233 g/cm^3	
F(000)	2800	
Crystal size	0.283 x 0.156 x 0.149 mm^3	
Theta range for data collection	2.00 to 50.22°.	
Index ranges	-43<=h<=14, -26<=k<=25, 0<=l<=33	
Reflections collected	49674	
Independent reflections	49674 [R(int) = 0.125]	
Completeness to theta = 67.679°	99.3 %	
Absorption correction	Semi-empirical from equivalents	
Refinement method	Full-matrix least-squares on F^2	
Data / restraints / parameters	49674 / 214 / 1569	
Goodness-of-fit on F^2	1.019	
Final R indices [I>2sigma(I)]	R_1 = 0.0583, wR_2 = 0.1083	

R indices (all data)	R_1 = 0.1219, wR_2 = 0.1285
Largest diff. peak and hole	0.86 and -0.56 e.Å^{-3}

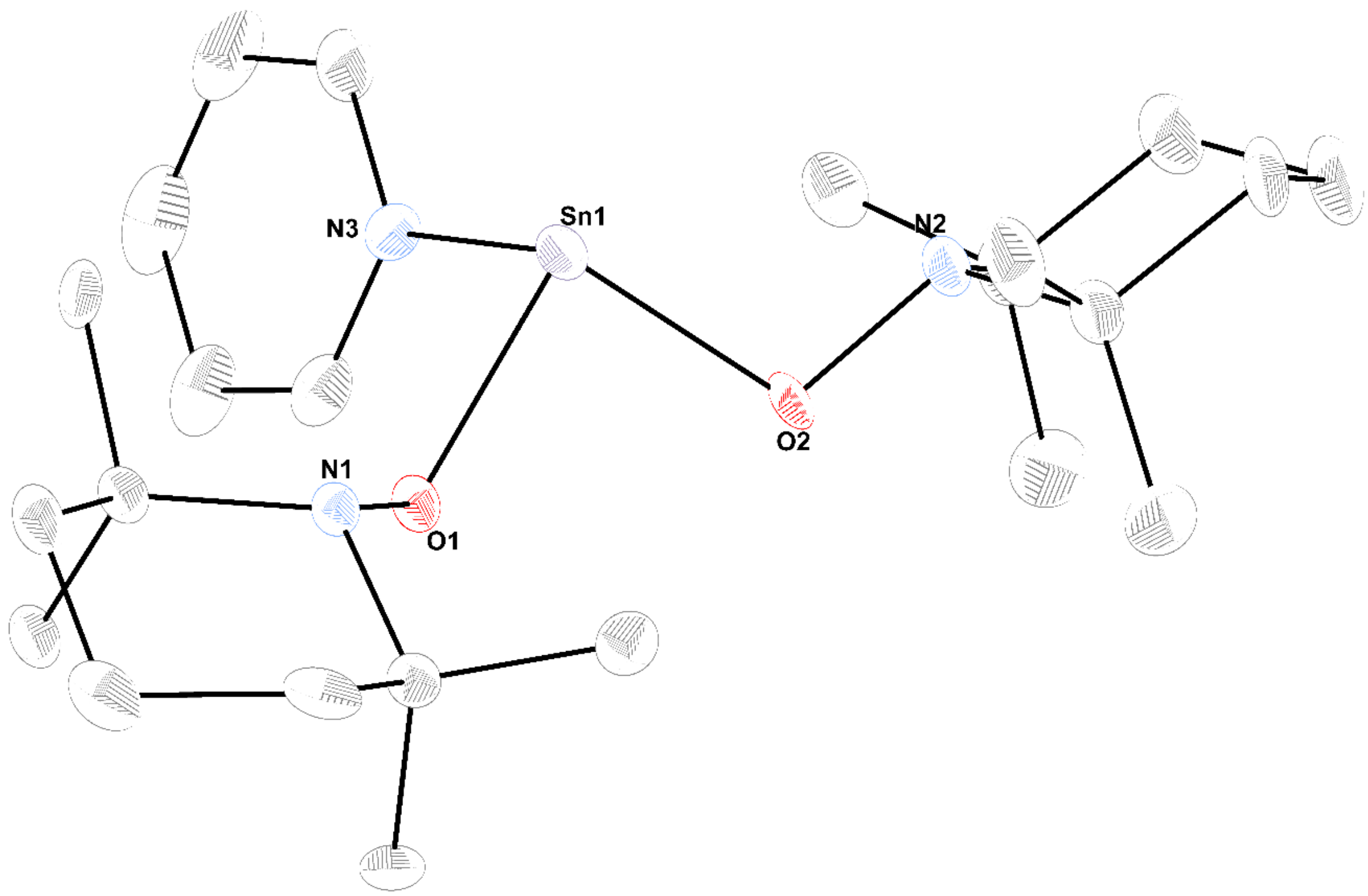

Figure S74. Solid-state structure of **[Sn]** with thermal ellipsoids represented at the 50% probability level. Hydrogen atoms omitted for clarity.

Table S15. Crystal data and structure refinement for **[Sn]**

CCDC	CSD 2504184	
Empirical formula	$C_{23}H_{41}N_3O_2Sn$	
Formula weight	510.28	
Temperature	100(2) K	
Wavelength	0.71073 Å	
Crystal system	Orthorhombic	
Space group	$Pbcn$	
Unit cell dimensions	a = 22.454(3) Å	**α**= 90°.
	b = 20.287(3) Å	**β**= 90 °.
	c = 10.8685(15) Å	**γ** = 90°.
Volume	4950.9(12) $Å^3$	
Z	8	
Density (calculated)	1.369 g/cm^3	
F(000)	2128	
Crystal size	0.188 x 0.14 x 0.12 mm^3	
Theta range for data collection	3.63 to 50.13°.	
Index ranges	0<=h<=26, -24<=k<=0, 0<=l<=12	
Reflections collected	14034	
Independent reflections	14034 [R_{int} = 0.1041, 0.0939]	
Completeness to theta = 67.679°	99.7 %	
Max. and min. transmission	0.6623 and 0.7452	
Refinement method	Full-matrix least-squares on F^2	
Data / restraints / parameters	14034 / 0 / 271	
Goodness-of-fit on F^2	1.039	
Final R indices [I>2sigma(I)]	R_1 = 0.0599, wR_2 = 0.0784	
R indices (all data)	R_1 = 0.1371, wR_2 = 0.0972	

Largest diff. peak and hole	0.58 and -0.45 e.Å^{-3}

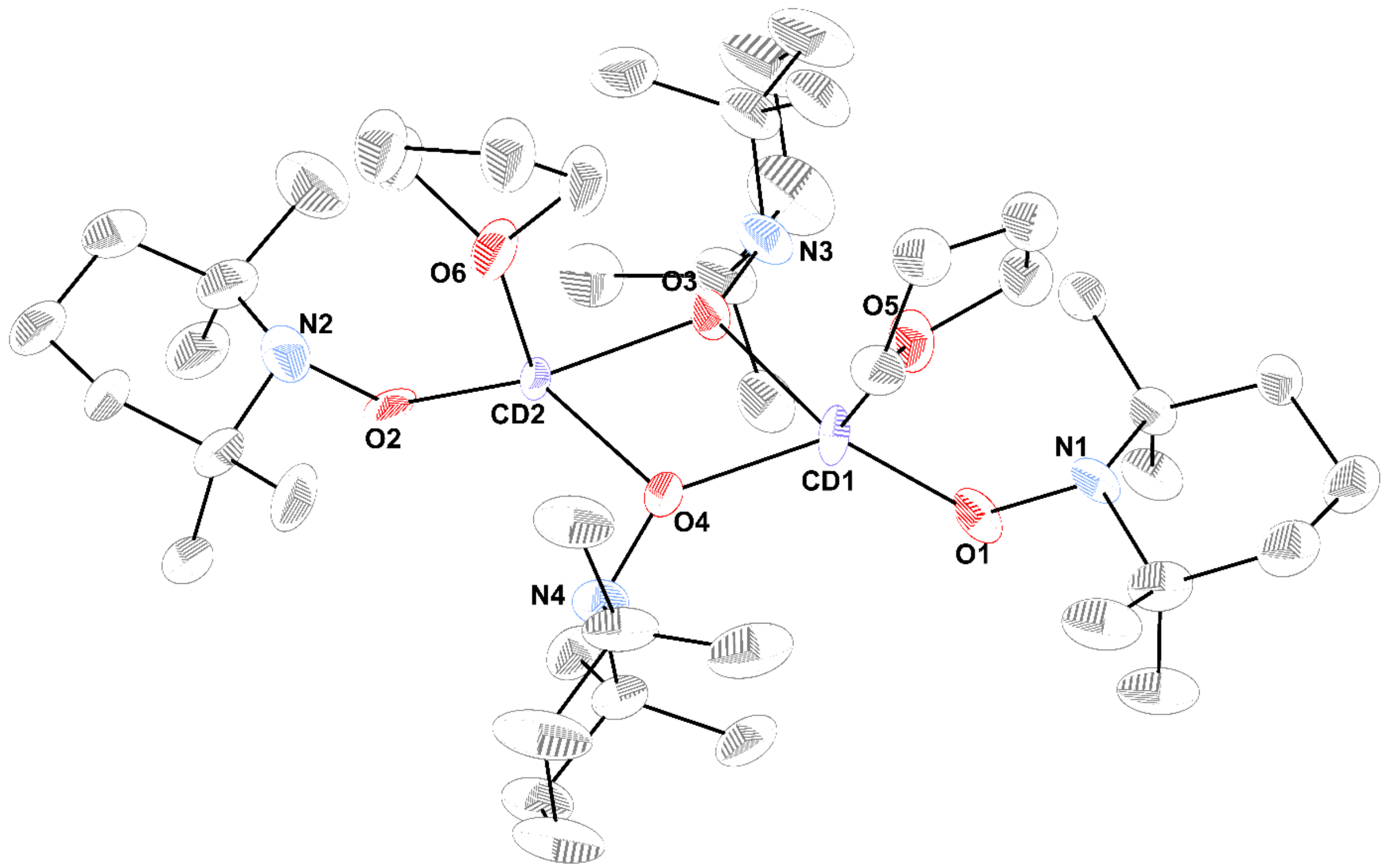

Figure S75. Solid-state structure of **[Cd]** with thermal ellipsoids represented at the 50% probability level. Hydrogen atoms omitted for clarity.

Table S16. Crystal data and structure refinement for **[Cd]**

CCDC	CSD 2514638	
Empirical formula	$C_{44}H_{88}Cd_2N_4O_6$	
Formula weight	993.98	
Temperature	100(2) K	
Wavelength	0.71073 Å	
Crystal system	monoclininc	
Space group	$C2/c$ (15)	
Unit cell dimensions	a = 21.112(2) Å	**α**= 90°.
	b = 9.3900(9) Å	**β**= 103.922(4) °.
	c = 27.140(3) Å	**γ** = 90°.
Volume	5222.2(9) Å^3	
Z	4	
Density (calculated)	1.264 g/cm^3	
F(000)	2096	
Crystal size	0.34 x 0.309 x 0.298 mm^3	
Theta range for data collection	3.09 to 52.87°.	
Index ranges	−26<=h<=25, 0<=k<=11, 0<=l<=33	
Reflections collected	5369	
Independent reflections	5369 [R_{int} = 0.059, 0.0214]	
Completeness to theta = 67.679°	100.0 %	
Max. and min. transmission	0.5995 and 0.7454	
Refinement method	Full-matrix least-squares on F^2	
Data / restraints / parameters	5369 / 870 / 691	
Goodness-of-fit on F^2	1.099	
Final R indices [I>2sigma(I)]	R_1 = 0.0315, wR_2 = 0.0741	
R indices (all data)	R_1 = 0.0466, wR_2 = 0.0858	

Largest diff. peak and hole	0.85 and −0.54 e.Å^{-3}

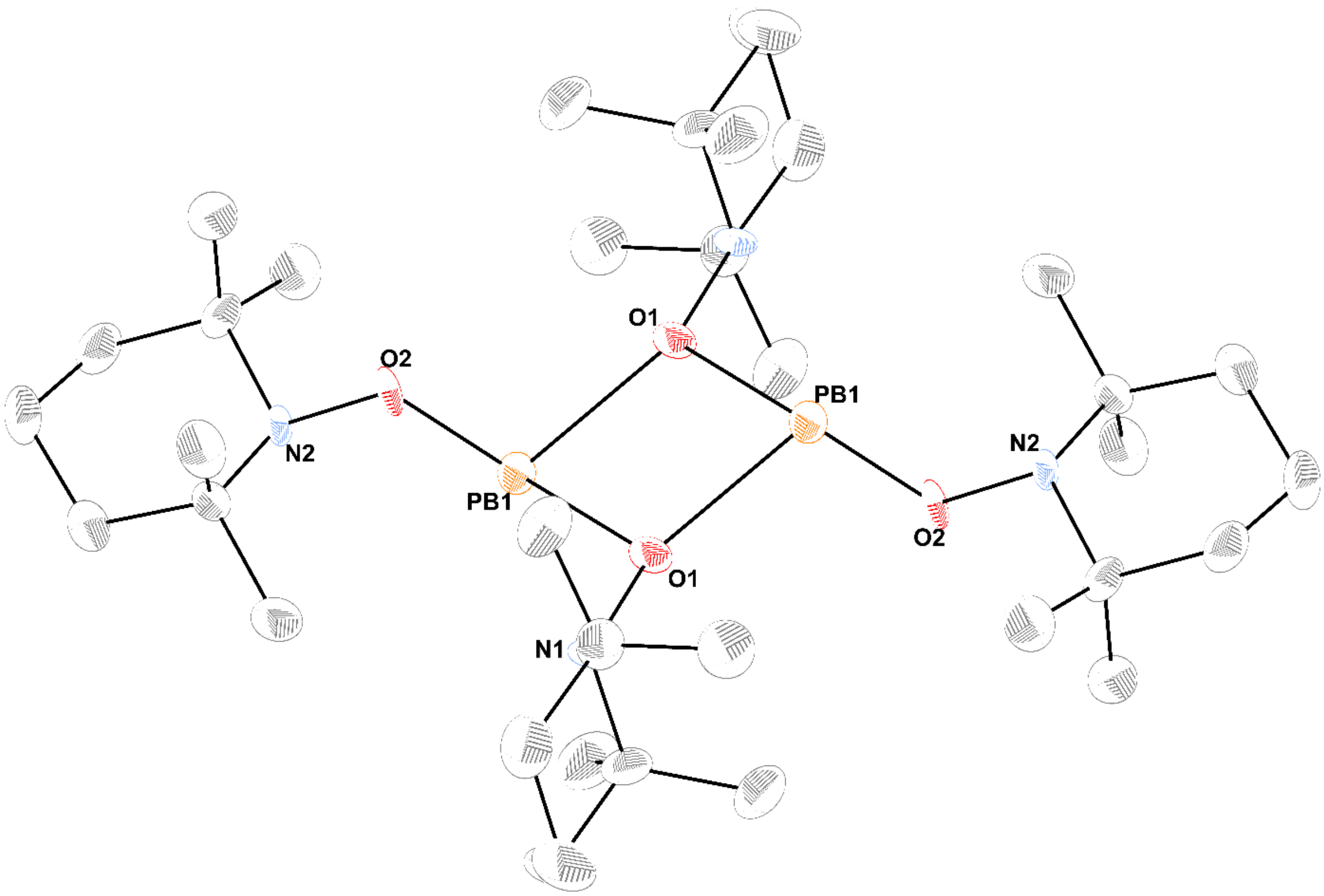

Figure S76. Solid-state structure of **[Pb]** with thermal ellipsoids represented at the 90% probability level. Hydrogen atoms omitted for clarity.

Table S17. Crystal data and structure refinement for **[Pb]**

CCDC	CSD 2514576	
Empirical formula	$C_{36}H_{72}N_4O_4Pb_2$	
Formula weight	1039.35	
Temperature	100(2) K	
Wavelength	0.71073 Å	
Crystal system	monoclininc	
Space group	$P2_1/c$ (14)	
Unit cell dimensions	a = 11.9118(13) Å	**α**= 90°.
	b = 21.487(2) Å	**β**= 103.753(2) °.
	c = 7.9618(9) Å	**γ** = 90°.
Volume	1979.4(4) Å^3	
Z	2	
Density (calculated)	1.744 g/cm^3	
F(000)	1024	
Crystal size	0.619 x 0.324 x 0.286 mm^3	
Theta range for data collection	3.52 to 50.22°.	
Index ranges	−14<=h<=14, −25<=k<=25, −9<=l<=9	
Reflections collected	42569	
Independent reflections	3528 [R_{int} = 0.0389, 0.0164]	
Completeness to theta = 67.679°	99.9 %	
Max. and min. transmission	0.1368 and 0.2586	
Refinement method	Full-matrix least-squares on F^2	
Data / restraints / parameters	3528 / 0 / 217	
Goodness-of-fit on F^2	1.185	
Final R indices [I>2sigma(I)]	R_1 = 0.0151, wR_2 = 0.0321	
R indices (all data)	R_1 = 0.0163, wR_2 = 0.0324	

Largest diff. peak and hole	0.71 and −0.47 e.Å^{-3}
Extinction Coefficient	0.00105(6)

10. Computational Studies

10.1 General Information

Density functional theory (DFT) calculations were performed using ORCA 6.0.0.[21] The B3LYP functional, the Ahlrich's def2-SVP, def2-TZVPP, and the auxiliary def2/J basis sets were utilized,[22,23] along with Grimme's D4 empirical dispersion were used to account for van der Waals interactions where indicated.[24] The proton affinity is defined by the following equation:[25]

$$B + H^+ \rightleftharpoons BH^+$$

Where H^+ is defined as the proton in the gas phase, B is the neutral base, here explicitly defined and evaluated as the nitrogen of the aminoxide ligand of the metal complex, and BH^+ is the N-protonated complex. By calculating the Gibbs free energy change of the reaction above, the intrinsic basicity of the nitrogen atom of the aminoxide complex can be estimated. Using the Gibbs free energy of the species above calculated at 298.15 K, corrected for zero-point energy, a thermodynamic cycle can be constructed. The gas-phase Gibbs free energy value of the proton in the gas phase is –6.28 kcal/mol.[26]

Geometry optimizations were performed on coordinates derived from the SCXRD structures and by optimizing all atoms using B3LYP-D4/def2-SVP level of theory in the gas phase. Frequencies were computed at the same level of theory to determine whether the structures are minima (with no imaginary frequency). Note: the calculations of **[Sr·Na]$_2$** and protonated **[Sr·Na]$_2$** resulted in one imaginary frequency each at -9.49 and -9.92 cm^{-1}, respectively. The cartesian coordinates of the structures are provided in section 10.2. Gibbs free energies were obtained by combining B3LYP-D4/def2-TZVPP single-point electronic energies with thermal free-energy corrections calculated at the B3LYP-D4/def2-SVP level. The following ORCA input file was used:

```
%pal  nprocs 4
      end

%maxcore 4000

%Compound

# SVP Geometry Opt Freq
New_Step
! B3LYP D4 def2-SVP RIJCOSX TightSCF Opt Freq

%base "opt"

* xyzfile 0 1 model.xyz
Step_End


# TZVPP SPE
New_Step
! B3LYP D4 def2-TZVPP RIJCOSX TightSCF

%base "SPE"
```

```
* xyzfile 0 1 opt.xyz
Step_End

EndRun
```

Calculations were attempted on B and BH by optimizing only the positions of the hydrogen atoms from coordinates derived from the SCXRD structures, however, these calculations yielded significant imaginary frequences in all cases and physically unmeaningful basicity values.

10.2 Cartesian Coordinates of def2-TZVPP Optimized Structures Relevant to Proton Affinity Calculations

Table S18. Determination of the proton affinity of the nitrogen atoms of the aminoxide ligands (species B of the proton affinity equation) of model metal aminoxide complexes.

Species (metal)	B (Hartree)	HB^+ (Hartree)	Proton Affinity (kcal/mol)
Mg	-2798.68664752	-2799.09943377	-265.30337284
Ca	-3400.69554026	-3401.11320201	-268.36274348
Sr	-3751.55015394	-3751.96583714	-267.12121076
Zn	-5492.43503633	-5492.84503097	-263.55163975
Sn	-1429.38034083	-1429.78278610	-258.81440846
Cd	-2733.86293334	-2734.27280105	-263.47198622
Pb	-2319.53906396	-2319.94162043	-258.88418283

Cartesian coordinates for the model of **[Mg]**

Mg	6.54805982501257	8.03745987154574	10.07202219264048
Mg	6.62002854622064	7.84595118733541	7.04180533109997
O	6.66747946223925	7.02230712916714	11.65792533098609
O	7.85403125654933	8.20037153833477	8.57952636231562
O	6.82508335842756	6.71485415253796	5.54522097543690
O	5.33423693721910	7.67836342179319	8.54146659874602
O	6.31536912951024	9.97929653600268	10.80832629807106
O	6.18596959904550	9.66583285563909	6.16107563848364
N	6.36042303201034	7.28155995356949	13.00925006386983
N	9.19675048347756	7.95109708398133	8.17883541549451
N	7.05866037843931	6.89532626513982	4.16669791972247
N	4.03521224791210	7.23536055070383	8.90893975783118
C	7.60307044931258	7.18205398281185	13.82335557503515
C	8.35647481395631	5.84468923487708	13.65394078065128
H	8.34991223521697	5.55468190446527	12.59596574125106
H	7.91041717137209	5.02885359175718	14.23759092805928
H	9.40305005123144	5.94881607051688	13.98290564950058
C	8.53652621745442	8.32129272451224	13.37801463472838
H	8.86499880329874	8.18911867914467	12.34122220998527
H	9.43188043128873	8.35102950473921	14.01939116868202
H	8.02730483573303	9.29209238391359	13.46173726537851

C	7.24224901628247	7.42912558227616	15.30211346938064
H	8.15216070838170	7.29468655474817	15.91116645505990
H	6.93866369800830	8.48556750845501	15.41444518512287
C	6.10996650537489	6.53959583614845	15.80206476556033
H	5.85974565248153	6.78955737643628	16.84714223901680
H	6.42477342197183	5.48278497084820	15.80889641209238
C	4.89153903658815	6.72965942141496	14.90619495392347
H	4.53639872127473	7.77031295705297	15.01223055200178
H	4.05904781715595	6.07830461956510	15.22189711414921
C	5.18080635419713	6.46431576134594	13.41472251131742
C	5.34999344211138	4.95234275296572	13.14737807028611
H	4.36704891755180	4.45616333843335	13.11105921994227
H	5.94368024518311	4.44644835426502	13.92026089640336
H	5.85043948441112	4.80583776462611	12.18218276334053
C	3.97193793357938	6.97292155302362	12.60862048462897
H	4.10178401881453	6.83304305492611	11.52787648076779
H	3.80710321459709	8.04516144227599	12.79597445269679
H	3.06028805192216	6.43653544645850	12.91823180368676
C	10.02304087659464	9.16690162838865	8.35037405773657
C	10.12635446535528	9.67138403097528	9.80940131021950
H	10.39682743441292	10.73933153265525	9.82927947950777
H	10.88374017320153	9.13441554008909	10.39397553496190
H	9.16528808773250	9.55539467465544	10.32874611898713
C	9.39795485729582	10.27693944374613	7.49058737489701
H	9.23824496430807	9.92257792771128	6.46367296142563
H	10.06411745450718	11.15363966937026	7.45895275306274
H	8.43046512170803	10.59417354435693	7.89399088695798
C	11.42135956250367	8.86887263053409	7.76530295141437
H	11.32420659441612	8.80352219235906	6.66909588672395
H	12.07674958190815	9.73071306001660	7.97703800924231
C	12.03843993943604	7.57189447383827	8.27827707586095
H	12.99156431986087	7.37925058269597	7.75779575595709
H	12.29080037740536	7.65810123172686	9.34867936674516
C	11.07017328844926	6.41432297055111	8.05264047268783
H	11.48566891701073	5.47285217631380	8.44807164307744
H	10.92018613367042	6.27002287258839	6.96966272985425
C	9.69340385494532	6.65212362374080	8.70647067938894
C	9.79791299500000	6.54841327692758	10.24088815277333
H	9.98874349638822	5.50084034459240	10.52193669515774
H	8.85806502780904	6.83478236178343	10.73188538089685
H	10.61057432233418	7.14932868018087	10.66630749930081
C	8.72876554063993	5.55414037998959	8.23477922137798
H	8.44292237394776	5.69166841973477	7.18311907538772
H	7.81473012270956	5.53656765962558	8.84328999117819
H	9.20359442459671	4.56669607447240	8.34134950766724
C	8.49565664364713	6.64124435351876	3.86421474139329

C	9.00952691493484	5.26178643823424	4.33290943012225
H	8.74350466417127	4.45098967481750	3.64231992500786
H	8.58523184988256	5.02531195152379	5.31549235517133
H	10.10814597930347	5.27088071232717	4.41978824535663
C	9.30720024166701	7.72928068890213	4.58510640087789
H	10.36816647708987	7.66804379099152	4.29219700969370
H	9.24974353787922	7.62993911305276	5.67728675352228
H	8.93834458546830	8.72841107770283	4.30330814543030
C	8.71269666004154	6.81329198762078	2.34773300166337
H	8.55878406578633	7.87685667888715	2.09153768033672
H	9.76615011286346	6.58102866204323	2.11662947636981
C	7.76463236909898	5.96519971228758	1.50803226202156
H	7.96602748146114	4.89193648182752	1.66181769844368
H	7.92913640343181	6.15505725220230	0.43368438267838
C	6.32777063094140	6.30077134913238	1.88865202951244
H	5.61061749493560	5.68089841163472	1.32449403865009
H	6.12812824820275	7.35002118194918	1.60694637043790
C	6.03150192480968	6.13994194680284	3.39446649933418
C	5.93126044058371	4.65012416811247	3.78759232228804
H	4.98268351373164	4.22182825952086	3.42590835292591
H	5.96158375993234	4.55989900295639	4.88132935716585
H	6.74508219527207	4.04377359774521	3.36921157794578
C	4.66948790826029	6.80228899759081	3.66467391482498
H	4.68949214155062	7.86272408084789	3.37439149452684
H	4.39663632041112	6.73537560439814	4.72296447350062
H	3.88369997604100	6.30504354188481	3.07362305313424
C	3.83943138893299	5.80138219431032	8.56469041504443
C	4.92741812596271	4.99153142681369	9.28460556642568
H	4.65933098943849	3.92343335389421	9.28491656801081
H	5.05539548441272	5.32348836952063	10.32160651388870
H	5.89474155512343	5.08475661562097	8.77541345359620
C	3.92490147527164	5.45752320201948	7.06461687928043
H	3.06581570325363	5.81584377983857	6.48449430138127
H	3.96258620635100	4.36335605570037	6.94341367854547
H	4.84078822604396	5.85684267643458	6.60714546051500
C	2.46723963496799	5.38847488293099	9.13679294924924
H	2.27502181445236	4.34063888331972	8.85339583214325
H	2.52723857288069	5.41808690467517	10.23752218298069
C	1.33265013230762	6.29630613323160	8.67334065579967
H	0.38180899620155	5.98855151875181	9.14001637262567
H	1.18217164837859	6.20016840501755	7.58483013063739
C	1.65127693128905	7.73996239695040	9.04633508317446
H	1.66724689736302	7.82299010950516	10.14632619542523
H	0.86678650172454	8.42641102008810	8.68545293013092
C	3.01424483008876	8.23140702074973	8.51168291736849
C	3.34399677393018	9.54341240335117	9.24147480656792

H	4.28964714662031	9.97302114298615	8.89060488821666
H	3.42808338067238	9.35864749655108	10.32169404059660
H	2.54319895528020	10.28183543302782	9.07744277153372
C	2.93659761219499	8.52200237308088	6.99520411213468
H	3.93817741231116	8.67638510114673	6.57248755387492
H	2.35261090491529	9.43950043601699	6.81759625091073
H	2.45416473652712	7.71571981306039	6.42970938238687
C	5.64309099464795	10.19246679298424	12.08286206394541
H	5.76200017584545	9.25720817070131	12.65711965715021
H	4.57762500020465	10.38186382915960	11.87557050340062
C	6.32510872905908	11.42340226086975	12.71209536186522
H	6.80779620602721	11.17449075705376	13.66824750181982
H	5.58822166145453	12.21583096284598	12.91330508428467
C	7.35725731672363	11.87681440670601	11.65779543067949
H	7.44618835117289	12.97044363243525	11.57975759254490
H	8.35371794170272	11.46796644369851	11.88504142091642
C	6.83288154937693	11.24220579806236	10.37576357047883
H	6.01468097546723	11.83989709024771	9.93077404496685
H	7.60045491780978	11.05363766160571	9.61794127969021
C	5.91554850918887	10.93488532911154	6.76849089340014
H	6.28298009033621	10.89420293507457	7.79848226355308
H	4.82323076820365	11.08951432205814	6.78926603775139
C	6.62081924885540	11.97255819403293	5.87449322812145
H	7.54835392418232	12.33425119050828	6.34007228624990
H	5.97180600240595	12.84456597046441	5.70578228917538
C	6.91923788137279	11.19912890850759	4.56286772274223
H	6.59428147435252	11.74399733379231	3.66459079798945
H	7.99808288872735	11.00596979667096	4.46442230036361
C	6.16321076766328	9.88332219536911	4.73165181325468
H	5.10702408780451	9.98304238073666	4.42100147514658
H	6.60072614615618	8.99069184881888	4.25288825593193

Cartesian coordinates for the model of protonated **[Mg]**

Mg	6.53743674557197	8.03329326824034	10.02669624683353
Mg	6.63977604254260	7.88480078385032	7.03761363492281
O	6.65132333706476	7.02136160264145	11.60424952494895
O	7.88901383779208	8.04077558158049	8.52839113498414
O	6.89415893467582	6.77241314179286	5.47505359785438
O	5.30010960172756	7.80627296069742	8.44558044030402
O	6.29579586916891	9.97978767030516	10.66358230802951
O	6.33668052985547	9.70202178397711	6.05239517745669
N	6.36330838757404	7.31001693501456	12.95915217389235
N	9.26897141969833	7.89816325632100	8.17934006001420
N	7.12424535234373	6.85038496181661	4.13969729775243
N	4.08980351001394	7.22413694685615	8.92707177634844
C	7.60160209850631	7.14577784106098	13.77243043546533

C	8.27648732374142	5.76501556748983	13.61974000544445
H	8.25816443188271	5.45692750972090	12.56697553981917
H	7.78428907162798	4.98439574271125	14.21361585720154
H	9.32545562585986	5.81448045284274	13.95243178891749
C	8.59646274355788	8.22511378742206	13.31466239244720
H	8.89982459552216	8.07238393888449	12.27396492274958
H	9.50020459743937	8.20157415832947	13.94367347265309
H	8.14971927253792	9.22573940165635	13.40481564612078
C	7.25694459523041	7.42949732292769	15.24882385973261
H	8.15863664330875	7.25031981344236	15.85780989161722
H	7.01547510506975	8.50259902741282	15.34998653983283
C	6.07589840959224	6.61382711240655	15.76132740259101
H	5.84203445787002	6.89319508208252	16.80200562128801
H	6.32718197347729	5.54060992639390	15.78435931970509
C	4.86955538445672	6.86296218017737	14.86388755075081
H	4.57301856727538	7.92295059641724	14.95636351934372
H	4.00191937820950	6.26378185752764	15.18701195843020
C	5.14245005143234	6.56195659157854	13.37608533342802
C	5.22295209920001	5.03919243369849	13.13110923945174
H	4.21346598643609	4.59761580791438	13.12952935330395
H	5.80521619257082	4.51410081364682	13.89883367570372
H	5.69077367818769	4.84152883174204	12.15849279169553
C	3.96559945426556	7.12280043182584	12.56103484623650
H	4.08665579094827	6.93700553669883	11.48648450761119
H	3.87242567433538	8.20891820687603	12.71584778278113
H	3.02255924558688	6.65694528723379	12.88926505962616
C	9.99108710003717	9.17122051614792	8.44414176897912
C	9.90224432600734	9.67389534081671	9.90063103949735
H	10.20107953794773	10.73257880786378	9.95294430448516
H	10.55938845490368	9.12061797293335	10.58159644006049
H	8.87931158313601	9.59507689286721	10.29013242675416
C	9.38658287776339	10.23517279328473	7.51609027996253
H	9.45231670617665	9.89718622625369	6.47133802073043
H	9.93743511011414	11.18395202292241	7.60515101958254
H	8.33331044420174	10.42319707879431	7.75367592329273
C	11.46484449943238	8.98776547742321	8.02155001931280
H	11.50213882215621	8.91117478357113	6.92074268211041
H	12.01812743264228	9.90192903005901	8.29258841262655
C	12.11965472465685	7.74722174180756	8.61455372635954
H	13.14790264285765	7.64375582746023	8.23148381159695
H	12.21170742372441	7.83777128239432	9.70913996845498
C	11.29037594710963	6.52364767332054	8.24547126099182
H	11.72752576707295	5.60533283443647	8.66879697602020
H	11.30078348352571	6.40263015191318	7.14796523112123
C	9.82471074475645	6.61996969652180	8.71908659682599
C	9.74354372268804	6.44852742274780	10.24576663252510

H	10.03758346983930	5.42091040499485	10.50949699481764
H	8.72406705589981	6.59167318967049	10.62917341163065
H	10.41411821898935	7.12026275015800	10.79406568899058
C	9.04309516109866	5.46592891479793	8.07908502433397
H	8.99037600689770	5.58424949863958	6.99023221090279
H	8.02197737718107	5.40619588914958	8.47397888414950
H	9.53636537132205	4.50760272937707	8.30076444221872
C	8.52964856243415	6.28559087848680	3.77557579723735
C	8.66190729606453	4.84207207917379	4.25959390365009
H	8.20599335251742	4.11676352343144	3.57606643898604
H	8.21398765272891	4.72332654573367	5.25295993772150
H	9.73036652497025	4.59488339832568	4.33601163893244
C	9.53429841693450	7.18856413710880	4.49891139775547
H	10.55302408960125	6.85475240755121	4.25416915219592
H	9.41719577719666	7.17129921042309	5.59129854745968
H	9.45208282871687	8.23225535336107	4.15070707627448
C	8.70692488710242	6.42473183760824	2.25375861519613
H	8.81934958035154	7.49575576248478	2.00251801409158
H	9.66644786598630	5.95202594582922	1.99154385568270
C	7.56082729342561	5.84139412699195	1.43209813316540
H	7.50477841403739	4.74866565328948	1.55805776045991
H	7.74887512419293	6.01229420190726	0.36093580683699
C	6.24359725177079	6.49956854512780	1.83420089002740
H	5.40059096045388	6.08674688646038	1.25829880113808
H	6.28468574484781	7.57569767909304	1.58174547463685
C	5.89890999622289	6.34874983170719	3.32508031533008
C	5.54544697387630	4.91334621857887	3.71134432648989
H	4.51154903831305	4.71434080263109	3.39403164707221
H	5.59880763271302	4.78118131625367	4.79795976632791
H	6.18608551336651	4.16762216746873	3.22802939628338
C	4.74394042127480	7.28719532435202	3.69067904135783
H	5.01554690471247	8.34485004424018	3.54148369821477
H	4.41565734002143	7.14929339288786	4.72694741118180
H	3.89120557412653	7.07410013130675	3.03003649235285
C	3.99783458850137	5.78656692306121	8.56270480409388
C	5.22790334368185	5.07356011114941	9.14189200835203
H	5.07109159686413	3.98435247077150	9.13430662741774
H	5.42867180178437	5.39272874595644	10.17394124430875
H	6.12606276406879	5.27192078424544	8.54129916171255
C	3.95175549426558	5.50111524705115	7.04744701941734
H	2.99833260615175	5.77696458414131	6.58138123513717
H	4.10523423064482	4.42525439557048	6.87073431460617
H	4.75741758397027	6.03004203587086	6.52134445151204
C	2.73805528284874	5.22225091173517	9.25134064110777
H	2.62401981175429	4.16741949960625	8.95299873061835
H	2.90761455144554	5.22932162018068	10.33982808438479

C	1.47820773496655	6.02024588656845	8.93379774722466
H	0.61912179418665	5.60910151233135	9.48822976284695
H	1.21698301324871	5.92863399087221	7.86560449919288
C	1.68976252895654	7.48022530466020	9.31725058994750
H	1.80243519047725	7.54392322058048	10.41155521693665
H	0.81199663650381	8.09233954309035	9.05181556574323
C	2.93856004127846	8.12097154873661	8.67138314853395
C	3.20161009252172	9.43987757713217	9.41403141930380
H	4.05254949398938	9.98234601063196	8.98695416232124
H	3.42216693509278	9.24063980093685	10.47117199599685
H	2.31516717117760	10.09015657401362	9.35942610328416
C	2.68893045556657	8.42173908329376	7.17487579461129
H	3.64019082685824	8.55951891293010	6.64170397171609
H	2.10133508890647	9.34608875188751	7.06133441106019
H	2.13312683974273	7.62119701925593	6.67005445435892
C	5.81754229455916	10.28697381724620	12.00329772931455
H	5.96298111181920	9.37731452766215	12.61290655943009
H	4.74163828698327	10.51983686644782	11.92537159185068
C	6.62793155482073	11.51746363330060	12.42251603898069
H	7.48629006639059	11.22560778726553	13.04369187899624
H	6.01368625931942	12.21198201846747	13.01318120936806
C	7.11055427966641	12.14439113219644	11.08687936647478
H	6.78479207963883	13.18713992595818	10.96189231527671
H	8.20801727984738	12.12755536976240	11.02363719123906
C	6.50062585910978	11.23818123676284	10.01174680453230
H	5.52384525544066	11.62403507773473	9.66692025338418
H	7.14845772597713	11.06656905479735	9.14452325778018
C	5.40637340415263	10.73374454637616	6.45943485301720
H	5.10858457776392	10.51568145601824	7.48931578211624
H	4.51382853403954	10.67373418575879	5.81396115296920
C	6.17134722894818	12.04141357413603	6.26537622779164
H	6.69420677734017	12.32344887662915	7.18982726819041
H	5.49301252055024	12.86575729258161	6.00432141726194
C	7.18883084822661	11.72687152865151	5.14000111624855
H	6.97642192299136	12.28402459289988	4.21677739325631
H	8.21110531591526	11.97802204452911	5.45233183118428
C	7.04271490726379	10.21404221412422	4.90745490513894
H	6.44311707897012	10.00731724206517	4.00247845966974
H	8.00234658520568	9.68608851177818	4.84434746198165
H	7.19527969934590	7.84157291523038	3.86195254711386

Cartesian coordinates for the model of **[Ca·Na2]**

Ca	8.27889138874210	15.42632269747831	11.18795759315456
O	10.28359758084516	15.13598028469598	11.95161163827574
N	11.34411386772095	15.36914560466131	12.84179651194344
C	10.84473335739034	15.36270655787703	14.24719121319468

C	10.07168305623163	14.08559597290412	14.63854452530606
H	9.35244839000160	13.83370950926143	13.84738746780606
H	9.50862080512763	14.25318317033648	15.57012423033697
H	10.72849496526564	13.22110166796901	14.79782330036777
C	9.88164867220681	16.55560683971287	14.39172357898124
H	10.35918506472921	17.48841501384978	14.04666189685094
H	9.60277347594337	16.69586550029269	15.44789339790094
H	8.95284589505756	16.40215406912920	13.82510902558211
C	12.04431428551544	15.59815034234687	15.18623933367429
H	12.40199970320605	16.63288132114585	15.03904167366940
H	11.69501597514497	15.52987540925280	16.23023967673869
C	13.20175568513391	14.63715254407375	14.93183819096791
H	14.04699765987460	14.87064732709307	15.60156007784940
H	12.89974112189296	13.60376834192828	15.17066522445427
C	13.63870335294736	14.73688456417679	13.47345871517985
H	14.45811561305777	14.03119443739834	13.25537494947701
H	14.03767702339863	15.75079610935656	13.29235774771093
C	12.48939462361247	14.48528708512428	12.47920623435048
C	12.11884390705427	12.98843641172526	12.41401613729007
H	11.14978944883340	12.88281451200146	11.90807453258714
H	12.04922690028210	12.52114257279082	13.40525879213398
H	12.87727660669001	12.43106157399365	11.84111948150492
C	12.95374502526020	14.91919800534331	11.07696841328466
H	12.16808309626591	14.72380663457601	10.33418750164966
H	13.85925227019086	14.36498162162457	10.78247080348845
H	13.19451345164273	15.99499244697722	11.06710414245846
O	8.69072815337775	17.46504619851191	10.58574248865751
N	9.35692625451156	18.42560397736777	9.80783982053146
C	9.35175100929964	18.02025766843051	8.37293486830806
C	10.17933697985261	16.72653125711435	8.25955440860025
H	10.33915111327026	16.46883548431345	7.20066014553176
H	9.67429246965954	15.87130527334105	8.73006614436887
H	11.17107688706750	16.84959250087808	8.72632669434496
C	7.94701850679668	17.73249510216249	7.80222040092832
H	7.38625573716363	17.09447075254912	8.49897818238199
H	8.02850784610366	17.19879094066663	6.84211609624761
H	7.36388554752674	18.64537298069943	7.62577521863005
C	10.06780076674730	19.11706633084360	7.55954116304301
H	11.13854454975521	19.10755879900048	7.83130310991333
H	10.01098491350597	18.85666017160196	6.48924679133856
C	9.51175609684885	20.51466039936658	7.81464083380222
H	10.08393073989554	21.26331947413695	7.24049446157705
H	8.47133062658028	20.58621198566751	7.45607830685770
C	9.58222942643552	20.82668312891069	9.30636369980544
H	9.16255754362659	21.82308278341175	9.52510948825615
H	10.64372121667943	20.85700307557818	9.61003231344810

C	8.85980382414947	19.78212947261776	10.17805378375885
C	7.32708187846166	19.92954054911014	10.07635877828692
H	6.97797378612170	20.04714215056619	9.04203250521955
H	6.99036862513533	20.81468677260897	10.63955520020732
H	6.85123106681836	19.03771480149437	10.50665427604440
C	9.26185541913407	20.01079386475578	11.64642926011114
H	10.35093762590959	19.89157843879721	11.76936750859484
H	8.74653732677335	19.29422556786202	12.30098713622064
H	8.99500050851500	21.03062863551926	11.96661085078619
O	7.49480361782888	14.06301309534397	9.70075769003024
N	7.12808295803174	12.79472470244825	9.22236049956936
C	6.61143926761559	12.92830648079416	7.83043353771713
C	7.59345487042578	13.62732709010535	6.86795022613201
H	8.41393963139167	12.97490969839762	6.54056221711687
H	8.02419171100029	14.50425574435985	7.36938761329749
H	7.06347902185547	13.96584111530817	5.96311988618453
C	5.33854479428715	13.79055468482943	7.91399376532584
H	4.85375892834992	13.85905696326861	6.92704793303254
H	5.58459066072988	14.80614839081275	8.25322921031356
H	4.61701011311226	13.34366956830973	8.61750361511380
C	6.22080925223588	11.52969642425231	7.31645162109580
H	5.33653186837718	11.18821104763101	7.88309464684490
H	5.90720896408000	11.61338329683141	6.26214918847402
C	7.33393473273179	10.49901301754062	7.47808305428224
H	6.98578817387203	9.50608279144309	7.14567290962990
H	8.18990991581710	10.75267258735451	6.83050096753333
C	7.77246497218930	10.44041841260627	8.93833404434926
H	8.59702080158563	9.72055645343753	9.07505637105569
H	6.92764973814160	10.07342861264205	9.54793869347244
C	8.21201334478836	11.80821544405877	9.49807815275642
C	9.59551939268371	12.21964287499589	8.95146016104727
H	9.68726092531795	12.07458515213485	7.86705057929367
H	10.38807178669475	11.62521394107231	9.43301400451053
H	9.78041461383298	13.27923243390185	9.17534465969689
C	8.33053802606404	11.67854597255726	11.02790118992299
H	7.35313108943478	11.43497530662462	11.47792203909370
H	8.71191157615226	12.59933080821354	11.49074483694408
H	9.02737334745961	10.86675675246003	11.28917402024967
O	6.66594046685651	15.02260659717221	12.58174387946311
N	5.31526554379858	15.04641211225440	12.96524092708779
C	5.18125822743375	14.43775350777988	14.32032094386627
C	6.07991367806035	15.09769836787877	15.38640353924490
H	7.07566093851662	15.26711721859821	14.95633644271292
H	6.18316200056765	14.43686434342882	16.26204535722824
H	5.68663206162744	16.05804773502811	15.74518592959400
C	5.61033749635212	12.96526875301672	14.18710364186253

H	5.01111660818326	12.45778046070756	13.41291502334366
H	6.67368027371485	12.89629076346112	13.91877437775609
H	5.45464498420570	12.43134957163854	15.13806072302972
C	3.69747182476394	14.47810154643544	14.73197729344474
H	3.13923554405692	13.77724713131045	14.08590939707583
H	3.60394855595702	14.09884776448319	15.76367492699697
C	3.07730615615435	15.86564711668094	14.59970391967131
H	2.00533815384845	15.83158714781530	14.85884084879283
H	3.54222171032407	16.56235171513820	15.31699454805246
C	3.25937838086259	16.37489099330304	13.17299882243161
H	2.83692203184205	17.38721639671184	13.05700103672488
H	2.69786328203472	15.71637054225343	12.48664248180403
C	4.73293759415909	16.39762319307156	12.72129363957447
C	5.50553571340973	17.55204870493966	13.39401522467007
H	6.58449425444323	17.41833234133188	13.23438677246387
H	5.32231135102842	17.61839142935074	14.47419544347323
H	5.21201929243389	18.51598651563401	12.94871212796107
C	4.76508693543187	16.63861375914214	11.20109617182081
H	5.79139860299018	16.76990465773505	10.83048896997710
H	4.20653513524313	17.55327311955285	10.94718050789477
H	4.29692764049546	15.79975137738808	10.65916944537700
Na	5.75661927340755	13.57246657616228	11.08177343321396
O	3.91095762671616	12.24978193758280	10.94677603322143
C	2.55737569113216	12.71998382600083	11.04966822012532
H	2.43168144254784	13.58390660879521	10.37761589197919
H	2.37463703569007	13.05993507274820	12.08406650271095
C	1.67977027908359	11.52401874257849	10.67966028756603
H	1.53307753859300	11.47661845720573	9.58778678397330
H	0.68815194356545	11.56451009830027	11.15420780975288
C	2.53900404035816	10.34425824388880	11.15307117259593
H	2.44275419395821	10.21257408760771	12.24373322472819
H	2.28060628797642	9.38990149861124	10.67067983507872
C	3.94562314206774	10.82236471013256	10.79639648450332
H	4.73143856200600	10.41876542589215	11.45434899048323
H	4.21061272708658	10.57500853186848	9.75313716136652
Na	10.80592307248546	17.27177189253275	11.39730437727522
O	12.66055333520877	18.57700849004098	11.52819223169349
C	13.31436037246005	19.17455048773312	10.39719008391517
H	12.68910983198939	20.00189811710323	10.01811485522186
H	13.40292393769050	18.41892877964598	9.60040218241314
C	14.66025719618826	19.67353395190758	10.92237138995148
H	15.05088982003669	20.52415761132721	10.34444773628155
H	15.40957563802164	18.86520327109374	10.88935119236393
C	14.31860408084903	20.02332237136456	12.37681990662492
H	15.19642791810445	20.06038096717321	13.03890128105508
H	13.81187248314390	21.00148093057814	12.42666783976996

C	13.34970310771983	18.90083008333925	12.74592962791859
H	13.88159184511405	18.00362613228918	13.10860102790558
H	12.60511632692277	19.18827266252926	13.50507479485042

Cartesian coordinates for the model of protonated **[Ca·Na$_2$]**

Ca	8.21060221836246	15.12738744284062	11.17152744495005
O	9.97353152883592	15.12399885797760	12.35100501610227
N	11.20835992393355	15.41153203189719	12.95968313110119
C	11.03832169721863	15.46453605074749	14.44021362374636
C	10.41517518841925	14.18426274864045	15.03600987626370
H	9.62569829377297	13.81380851637186	14.36892611645417
H	9.97343881545070	14.39347570763877	16.02316177841886
H	11.15014287156148	13.38050286519338	15.17272401244934
C	10.10424892996621	16.65245120990380	14.73382890505605
H	10.52950635833965	17.58834438496382	14.33331856430522
H	9.97719862203597	16.78719918150593	15.81934478092519
H	9.11475452429975	16.50003300796347	14.28293799310936
C	12.40665022905021	15.75915900485668	15.08468524532298
H	12.69732608940248	16.79307800210407	14.83042471588519
H	12.29314216562131	15.72569914337082	16.18106319222393
C	13.50677569940515	14.81228255506735	14.61587899099366
H	14.47090998177653	15.09787590194784	15.06793905330041
H	13.30578654398753	13.78401349791806	14.95928528640764
C	13.60458523840438	14.85960347248722	13.09466546434810
H	14.37562821293190	14.16382468081935	12.72512189584337
H	13.92446420123062	15.87233535124165	12.79206826544674
C	12.27472373981776	14.53740495658369	12.38566717325056
C	11.95181250231884	13.03217532913390	12.46529483736584
H	10.91592983969443	12.85994693314031	12.14631221837495
H	12.07572677558100	12.62242404838984	13.47570127914810
H	12.61792560489746	12.46532717214565	11.79633381269982
C	12.42055508808012	14.92404244407216	10.90360252149630
H	11.50704853333859	14.69756011678077	10.33710750083765
H	13.25057759485283	14.36806683998765	10.44115828312093
H	12.64607589403092	15.99877461848029	10.80546546449441
O	8.38818767341501	17.21778142603285	10.70739272952084
N	8.96701439057990	18.25217399689385	9.94203501210361
C	8.98763314282669	17.86755507561626	8.50087926990994
C	9.94477783059667	16.67041809839118	8.36237184282065
H	10.11771208916616	16.43994288953401	7.29989663823584
H	9.54573075175238	15.75603976787307	8.82526716869189
H	10.92032225072269	16.89726353536258	8.82010086293910
C	7.61049746572615	17.45005421728195	7.94222795395321
H	7.11961265349729	16.75404047658868	8.63739922093335
H	7.73193366414128	16.93846140097473	6.97476140071119
H	6.94037042155030	18.30432228905928	7.78306666934766

C	9.58041603152169	19.03690833330822	7.68875269400770
H	10.65226970480048	19.12437590927041	7.93711034392068
H	9.52600772969999	18.78236213586006	6.61752998087784
C	8.90394153597338	20.37385429381618	7.97207379046397
H	9.39469337104743	21.17684118453404	7.39791920818597
H	7.85479458429293	20.35753855655926	7.63339375330062
C	8.98097002346229	20.67161794551151	9.46547503503931
H	8.48246423512067	21.62452612269141	9.70862973959756
H	10.04118919771700	20.79066754062468	9.74861061301891
C	8.36624419410554	19.55992480033620	10.33680226308334
C	6.82651085691521	19.58328298804361	10.25270091680018
H	6.45945909484210	19.69821741080574	9.22463064634703
H	6.42311239444818	20.42362337512735	10.83978544791773
H	6.42880794353259	18.64580970490795	10.65898141158640
C	8.77872831994778	19.81503216359563	11.79861872010482
H	9.87671139138362	19.80806657101112	11.89581498505168
H	8.35522120019077	19.05298733673995	12.46791842233564
H	8.42208868963133	20.80104888919853	12.13473882421126
O	7.70607562255188	13.62338439314099	9.71956801056236
N	7.48378627158661	12.35464547569707	9.14817065898882
C	7.24408169589016	12.49701671794551	7.68008606719916
C	8.36615520445880	13.24443750556339	6.93459921216561
H	9.26474980982144	12.63045539828316	6.79795404881106
H	8.64516761085618	14.14657974115769	7.49310082934883
H	8.02032145131498	13.54719453192850	5.93398182292799
C	5.94591623829881	13.30848052401294	7.52186323224346
H	5.66833792481049	13.39387911682691	6.46039999101310
H	6.06235551664141	14.32312039801912	7.92994968605594
H	5.10861809616099	12.79735990182348	8.03211437023232
C	7.01443508229348	11.09337813680713	7.08720194730926
H	6.05049113674808	10.70591372474855	7.46448975210518
H	6.91050326444842	11.18619050259738	5.99371584735025
C	8.11337997227751	10.10065426275189	7.45301003488160
H	7.87817050905088	9.10557494556968	7.04155688341526
H	9.07090590493866	10.39911298766645	6.99562845070501
C	8.24756780854862	10.02309270180456	8.97085463486476
H	9.05093131222118	9.32715337637962	9.26304902213447
H	7.30939697510150	9.61545084368071	9.38901333361484
C	8.52227090977803	11.39169877596746	9.62415759517247
C	9.96969379178894	11.85061473370459	9.35881033709156
H	10.27031394183994	11.72049975136984	8.31192473552103
H	10.67174794139489	11.27132571228454	9.97716602661856
H	10.08561360925708	12.91103722168259	9.61881771868896
C	8.33875342002210	11.24436920141203	11.14579982204797
H	7.29818944380879	10.96377182293040	11.39058460087893
H	8.59544055099177	12.16878603026119	11.68383935518166

H	8.98944840230567	10.44618524316900	11.53403856108549
O	6.15748213663302	14.64055225861476	12.10389171129524
N	5.18364736255825	14.95367195393995	13.00248647363291
C	5.41966237484221	14.25106524347535	14.36534327067110
C	6.75336060803263	14.66764374290027	14.98213964533332
H	7.54573752794295	14.69958116988613	14.22440283555890
H	7.04450143593745	13.91390789085995	15.72762391368378
H	6.71702233849758	15.63492450399283	15.49482598348660
C	5.45943759874306	12.75039816394050	14.05581808310721
H	4.57359677895482	12.42849212994122	13.48621867760332
H	6.36568602450710	12.48012625835541	13.49737169682152
H	5.46165466556463	12.19040824166543	15.00214166854136
C	4.21591811269041	14.57594913518513	15.26492802970464
H	3.32356443468510	14.05575361779629	14.86960907534417
H	4.40995417251952	14.13673343069767	16.25612608398349
C	3.91617247709668	16.06949852340029	15.37232927322006
H	3.02949618366572	16.22538699390443	16.00625827097701
H	4.74362598378301	16.59609931779749	15.87392343653040
C	3.66812372745833	16.65703043524610	13.98512958695908
H	3.45442980584669	17.73557473625410	14.04323245137451
H	2.76319949676726	16.19115377547042	13.55259820310559
C	4.83996541643342	16.46600620866950	13.00550465527138
C	6.04769262794968	17.33188304198804	13.36132425159939
H	6.89984382184383	17.15428132875620	12.69111922909157
H	6.37288912250045	17.22886593964266	14.40119827014063
H	5.75648211539767	18.38168372971702	13.21308374396518
C	4.38804395965150	16.78921603248466	11.57874401299453
H	5.21145488298728	16.68188841701879	10.86144415540537
H	4.03535244871921	17.82964620013197	11.54224721367023
H	3.55174767046844	16.14473208704355	11.26734968421085
Na	5.75719640254647	13.03795117773254	10.56444569129243
O	3.48170368682796	13.15939884959572	10.97992359341606
C	2.75255145828194	13.83147171510654	9.92554841461208
H	3.48031557660241	14.34850811026934	9.28245074137302
H	2.07776496938026	14.58625679274882	10.36845171409435
C	1.97115858023559	12.72492999176373	9.23276121468611
H	2.62803931843185	12.17180524942087	8.54106488688695
H	1.11544034294101	13.10543139692281	8.65679886664713
C	1.56000926966895	11.84785249403926	10.42326915638600
H	0.65958019478556	12.25844135686961	10.90699538052355
H	1.34120614377645	10.80780836132796	10.14254263251321
C	2.76875625451390	11.95300010168420	11.36258308861606
H	2.47985747952881	12.02504947611926	12.42338745084263
H	3.46061692216804	11.10029078816833	11.25184094345109
Na	10.48234993480378	17.28018581980845	11.61627404744752
O	12.27740607100159	18.67589600238219	11.54009558321313

C	12.82789915819541	19.20448180214117	10.31878319530006
H	12.18132427999635	20.02390804973758	9.95893761393271
H	12.82758482913516	18.40811299563045	9.55816223639259
C	14.22436100381301	19.70286837009754	10.68385242617027
H	14.57260646553781	20.50818074870763	10.02074421664746
H	14.95376130289114	18.87785180027608	10.62983314694518
C	14.02731617305035	20.14783153796561	12.13857493060230
H	14.96404706362153	20.20513071315539	12.71188151221240
H	13.54485451979353	21.13850413902091	12.17542610522755
C	13.08122223078526	19.07135197556897	12.66722849920802
H	13.63298292958435	18.19042057105113	13.03961214700984
H	12.41392807786780	19.42526242703445	13.46934661878028
H	4.31095888911857	14.51539448796191	12.65481176160535

Cartesian coordinates for the model of **[Sr·Na]₂**. This calculation resulted in one imaginary frequency at -9.49 cm^{-1}.

Sr	-3.55825126374131	14.26193721242542	6.46791395360239
Sr	-1.74011697331842	15.37606357153943	3.49459000367078
Na	-5.69521537354552	12.74410625141905	8.47132647314160
Na	0.51359255763861	15.63304364858095	1.09547773939592
O	-4.54938013807357	12.15515029875270	6.64869464400012
O	-4.79532390576616	14.81905324310415	8.35472638488584
O	-1.21743636937589	14.64398535273139	5.68815233728328
O	-4.07838664377243	15.22884606467760	4.37364193081674
O	-0.75877780258461	13.98433877332769	1.90523239229462
O	-0.44125694889701	17.06577289014948	2.58111741268177
O	2.09101879151156	15.63001582986432	-0.53962198717659
N	-5.39780900433856	11.03108075536234	6.59858286267094
N	-5.68694959109353	15.03706512529410	9.42086694646881
N	-0.95461531154647	14.53502274652315	7.07027270662089
N	-4.32865801465108	16.08733647517655	3.28326091310035
N	0.01101436219151	13.06076597160665	1.17252667664853
N	0.48833147865708	17.90626647524956	1.94060230437883
C	2.34460551464321	14.39928536494891	-1.23431106597026
H	1.93491598971494	14.45642286395498	-2.26139351026039
H	1.81886737951466	13.60588715322093	-0.68589401901305
C	3.86273325153591	14.27593364760033	-1.25141061388785
H	4.22190000840643	13.89675582680139	-0.27973863073310
H	4.22996000460844	13.60058396500850	-2.03843802731936
C	4.28287457208723	15.73862849675293	-1.45695585781187
H	4.22912923191990	16.00070738141208	-2.52629218640066
H	5.30478634537118	15.95438077047026	-1.11143031306153
C	3.22153181272628	16.51056754962745	-0.65819905284791
H	3.56883415119380	16.76305151514469	0.35712480704692
H	2.90830772495516	17.44622245118623	-1.14961160241986
C	-6.71894216991714	16.03678049971076	9.01709741628379

C	-7.67279814079068	16.26744210054627	10.20557276688215
H	-8.38749622706732	17.06236794854531	9.93394132281976
H	-8.26926385572760	15.35088668694786	10.35702694875605
C	-6.94773379119641	16.60501443361853	11.50498159188789
H	-7.67221839815876	16.70219171744285	12.33132586306476
H	-6.45104659066791	17.58602428584997	11.42187610783742
C	-5.92438011439251	15.51904130842502	11.82617182612440
H	-6.45577398471128	14.57523811128445	12.03918427201882
H	-5.35937862075217	15.76918095664786	12.73989391810157
C	-4.92295660042626	15.27289315301409	10.68102215671503
C	-7.52288761956702	15.40359076430422	7.86990209515969
H	-8.37893839228436	16.04052239459789	7.59650179296404
H	-7.91552380195013	14.41859781264293	8.17002347138771
H	-6.89766263409381	15.26914880505132	6.97819363163119
C	-6.13147648051255	17.37091306785677	8.50927012589927
H	-5.27300521794885	17.16438634470541	7.85562306202718
H	-5.79746264651980	18.02822808306935	9.32234896441979
H	-6.88352901839514	17.92374567672387	7.92491560943699
C	-4.14862544514507	13.97656266013583	10.97839959530783
H	-4.83397319658195	13.11334741770184	11.02873310136600
H	-3.63276135518003	14.04833490480740	11.94893055544911
H	-3.39547745866102	13.78578905490443	10.20064724384840
C	-3.90372461498226	16.42615306998831	10.58780263948091
H	-3.37250352478452	16.35745338080286	9.63147340763377
H	-3.16533752861161	16.35365053968951	11.40197142095482
H	-4.36964308574011	17.41764173620114	10.65354930327729
C	-0.17057638117020	13.29233414814111	7.35985920380995
C	0.02748421143908	13.20053430232938	8.88545723875718
H	-0.95897713455387	13.02214117425370	9.34952700567595
H	0.64586837137427	12.31400049664953	9.10515783326275
C	0.63623602986333	14.45852350382603	9.49415866161974
H	0.69951166640837	14.35809370184336	10.59081906819395
H	1.67244555261809	14.59444987313482	9.14144411497111
C	-0.21752240183748	15.66716822293062	9.12690086788645
H	0.22702875485102	16.59817420771856	9.51736645196430
H	-1.20615170980997	15.56536683322112	9.60402592115219
C	-0.42431267682018	15.82769788977705	7.60803568501267
C	1.17048915947670	13.20983102000436	6.60451299806116
H	1.53168599348503	12.16941229111577	6.59353935740362
H	1.96106508482567	13.82488405004180	7.05160188492941
H	1.02491691897147	13.52943057259963	5.56504478048828
C	-1.02547139576275	12.09622981751397	6.92481727855419
H	-1.16440192805014	12.07765023594921	5.83673707081294
H	-2.01564372001906	12.10729377401157	7.40472517623433
H	-0.53830173949414	11.15323552823453	7.21729701747587
C	0.86347469680907	16.31355291898328	6.91382952080467

H	0.78224671744832	16.14968587794895	5.83208291660911
H	1.76803747796655	15.80781807095470	7.27276216356039
H	1.00070921586658	17.39281297227868	7.08260088933666
C	-1.49831076071489	16.90109197038508	7.38144353264521
H	-1.59206978088567	17.15293678969018	6.31864689882997
H	-1.22825790239985	17.82412978006524	7.91787467260242
H	-2.48025524908783	16.57958873110283	7.76521542704867
C	-4.59466856553466	9.78861138997526	6.79590053909646
C	-5.53325962806748	8.56777548048143	6.70664640314987
H	-4.92098769666044	7.65241365393068	6.77057351460020
H	-6.19319892503347	8.56014754738525	7.59143020984065
C	-6.39336469315528	8.56055288154714	5.44640396720556
H	-7.08335693486624	7.69979670405115	5.46008790427530
H	-5.76179986214806	8.42915321121408	4.55220763991792
C	-7.17831338312128	9.86501087066576	5.35061188132049
H	-7.89710537921339	9.91168039317213	6.18717218200603
H	-7.77768322461295	9.89859407716235	4.42527355268283
C	-6.28169528272038	11.11870938178498	5.39994102940112
C	-4.02627851084848	9.85170005519704	8.22462496306933
H	-3.53585597283773	8.90028352831578	8.48584815985627
H	-4.83217813733930	10.02960802531602	8.95539248534897
H	-3.28496456226230	10.65551912315420	8.32119339662436
C	-3.41812059466954	9.64090602869785	5.80688747005114
H	-3.72711177447703	9.23367564229464	4.83506270126096
H	-2.65405369830617	8.96171994071946	6.21746879242479
H	-2.95461845296130	10.62017641612001	5.63962290405533
C	-7.18597668177644	12.34519641659033	5.60569299771478
H	-6.60223666443541	13.27462139831867	5.59144001422082
H	-7.71454201540803	12.27869232018920	6.57068042210910
H	-7.94372385656967	12.40857840735750	4.80932799848457
C	-5.52006855119180	11.29788538039442	4.07124124805271
H	-5.04046686442871	10.37387568481599	3.72516901836888
H	-4.74208726588668	12.06228197193547	4.19475337343646
H	-6.20584391490048	11.63252110599996	3.27781047890680
C	-4.79910723079251	17.42420834239870	3.76415414482784
C	-4.98982298212713	18.33304684700731	2.53377502563813
H	-5.39101352073538	19.30255016381202	2.87436343676068
H	-3.99844622948652	18.53749677275909	2.09701515651111
C	-5.88372003674849	17.71904115824316	1.46187456599143
H	-6.91942734117887	17.62410939731880	1.82962748181070
H	-5.93395577027422	18.38428304584889	0.58334813334788
C	-5.33480375664309	16.35515971596571	1.05816463574763
H	-4.34896563654439	16.49481003822078	0.57967891596191
H	-5.98485466753810	15.87174203537680	0.30946960260166
C	-5.16019960285973	15.39314375300562	2.24872609967545
C	-3.68574742271049	18.02493412520238	4.63479400387620

H	-2.71080036752265	17.99793311698491	4.12163921498843
H	-3.60081353218715	17.49884000983678	5.59276298119957
H	-3.90833087670373	19.08071990700000	4.85449519254179
C	-6.07626435956451	17.36706838300326	4.62656690867306
H	-6.99391120757498	17.25518928024989	4.03683721301422
H	-6.17523490427643	18.29209592694817	5.21563863499381
H	-6.01185161814052	16.52522604094381	5.32874668202923
C	-6.51838130164985	14.88644249800623	2.77323138748825
H	-7.27917529666528	15.67361848557437	2.84295537023408
H	-6.38383482910506	14.44164224956626	3.76616039481617
H	-6.91439571535064	14.10796621623190	2.10209831816133
C	-4.36453089597218	14.17615512189105	1.76410719023030
H	-4.87616649445648	13.69802320560657	0.91447356124702
H	-4.26101690686635	13.42513355428170	2.55706841904254
H	-3.36055439161964	14.46034559721680	1.41466921994770
C	-0.87045784163077	12.29746143274700	0.24156882494638
C	-0.01194196655924	11.27955010854115	-0.53660632354626
H	-0.68085812134739	10.66617957739205	-1.16369058274404
H	0.64806667078214	11.82699771125437	-1.23155929466052
C	0.84406855946104	10.40001220807081	0.36964779965585
H	1.47769924157224	9.72971487675067	-0.23579066978489
H	0.20392483842455	9.74220047014505	0.98074904006239
C	1.71138202357548	11.27720154424456	1.26661286603647
H	2.42966019421987	11.82961605967878	0.63538895209914
H	2.31279792600421	10.66362028444953	1.95826111403946
C	0.90036688153178	12.29554999679292	2.09394641816935
C	-1.43562590510999	13.31644798286610	-0.76342620308485
H	-0.61882932031390	13.88694387704119	-1.23381200526047
H	-2.10985089299637	14.02645485407435	-0.26827055754861
H	-1.99793067891599	12.80239109909943	-1.55936052453712
C	-2.05150767824156	11.58625018003778	0.93780279078839
H	-2.86056464999066	11.38963842310356	0.21662120201877
H	-2.44862217953930	12.22680215334809	1.73329910601412
H	-1.76765411202283	10.62171242460727	1.37909971193478
C	1.88693384658478	13.30963541426027	2.69853387087725
H	2.63949667187966	12.80071892057694	3.32052300373152
H	1.36596790935182	14.04015884269042	3.33142527718411
H	2.41919955943570	13.85082391727983	1.89866249873189
C	0.15765817117291	11.59735186911249	3.25046279964469
H	-0.40831867810880	10.71614420888264	2.92426722414281
H	-0.54251826114036	12.30271228836947	3.71676288682376
H	0.87102879266250	11.26737740757138	4.02097275963905
C	-0.22672243076449	18.91319129563965	1.10318842171611
C	0.81806593115591	19.82388816838738	0.43085798845697
H	1.38487098263734	19.22493357155128	-0.30397841777160
H	0.28948866479896	20.60608544548320	-0.13971479293590

C	1.79906231614550	20.43720740801477	1.42503413792098
H	1.27375067409453	21.12392266996106	2.10940201953698
H	2.54777496311366	21.05018046774789	0.89499090687939
C	2.48799433612812	19.32524264147710	2.20882504358856
H	3.18615671270308	19.73947829233206	2.95566148462533
H	3.09462298661925	18.72751708560217	1.50685187111754
C	1.50292797126296	18.38138566662495	2.92580059948576
C	-0.97031834451928	18.13111536522312	0.00510930024601
H	-1.46094198517624	18.82336969944976	-0.69705320259894
H	-1.74131839222250	17.48214193955329	0.44447715681711
H	-0.26796411953267	17.51106876268978	-0.57772026366622
C	-1.26529720713998	19.74692145356504	1.88083950114060
H	-1.96434186199160	20.23676087401116	1.18443880131771
H	-0.81038907804695	20.53411001392652	2.49571711338037
H	-1.83861615976364	19.08347137604835	2.53891791990809
C	2.28285470232554	17.14016795210489	3.39162828378577
H	2.73077843243395	16.61505657280726	2.53191580448604
H	1.62371773855672	16.43936933041901	3.91850183855276
H	3.09807146782021	17.42539815067546	4.07497096994293
C	0.88912203476666	19.05803716163801	4.16970654604055
H	0.53672834801398	20.07783044909394	3.96864389952810
H	1.63192154077923	19.11783373163982	4.98075992238416
H	0.03786900937527	18.46130336219342	4.52423875795702
O	-7.21692838869998	11.64692480300157	9.75241314685619
C	-8.10933522488999	12.27380850217332	10.67109269061094
H	-7.73397783175985	13.28725779282786	10.85974792932541
H	-8.11546111265838	11.70950372513078	11.62386006685509
C	-9.48198405809660	12.22232686755556	9.98213033364870
H	-10.30051565041265	12.11889048250183	10.70967645419998
H	-9.65854244335178	13.14797135106966	9.41393316527133
C	-9.36578376382830	11.00404310515605	9.02620844197165
H	-10.09030851655740	10.21057951161553	9.26190751070819
H	-9.53753696987454	11.30710020849055	7.98250164009631
C	-7.91742216336390	10.52050885198246	9.22178481161752
H	-7.86725952378665	9.68371336199044	9.94463206733314
H	-7.40386899031013	10.22843902853163	8.29761353520455

Cartesian coordinates for the model of protonated **[Sr·Na]$_2$**. This calculation resulted in one imaginary frequency at -9.92 cm^{-1}.

Sr	-3.57104666845328	14.50543404154236	6.57142146177771
Sr	-1.75819487545272	15.44052590934316	3.58061886890499
Na	-5.66963672745912	12.90133723211750	8.68556373305722
Na	0.48846813326295	15.31067097818634	1.16577370625060
O	-4.73271172695540	12.22154110858612	6.73151879717824
O	-4.84341872334613	14.91630567628406	8.44464973197328
O	-1.27962551696452	14.83869926454833	5.85276964328992

O	-4.15763653893879	15.27606944271390	4.41229004395096
O	-0.86970192457235	13.81303605162808	2.19634032899071
O	-0.35938059872394	16.90037723450594	2.54191449845722
O	1.99542131419056	15.18383058913144	-0.51790092683441
N	-5.29945357080419	11.08946789964570	6.21901705444189
N	-5.72188629568477	15.07607159564223	9.53809967282632
N	-0.99894284090202	14.80580220283675	7.24024065112150
N	-4.34577249840321	16.27532749556187	3.42017473881885
N	-0.11330483275696	12.84707842393413	1.50589263836233
N	0.53132870290680	17.70193872100604	1.80772625313605
C	2.28363858376929	13.93043571807827	-1.15724685078940
H	1.76342387650220	13.87909107992174	-2.13285787639148
H	1.89207050509547	13.13397060318458	-0.51040420979556
C	3.79632245274226	13.92710926992295	-1.33652449169920
H	4.28993978243568	13.64833351457366	-0.39018791009785
H	4.13457712916520	13.22968597118060	-2.11703645636548
C	4.06198479935748	15.40014946279889	-1.67719513653457
H	3.86294819259907	15.58309488226408	-2.74560874262262
H	5.09422818401369	15.71885179449594	-1.47073603893006
C	3.03776876318932	16.13773202131247	-0.80403616323958
H	3.47032885843498	16.47075852191285	0.15350971692422
H	2.60017819098940	17.01736067361205	-1.30224044497788
C	-6.83943545272656	15.99390242417314	9.16250163460959
C	-7.77665180964800	16.14092055803252	10.37723459035278
H	-8.56092472367973	16.87496772279573	10.12954557981592
H	-8.29337625203841	15.17761775235249	10.54147490194772
C	-7.04635911156230	16.53124877097627	11.65810344559191
H	-7.75490843319747	16.57075606970210	12.50153909301210
H	-6.62761363399764	17.54695949644638	11.56890687838799
C	-5.94081112632823	15.52128180136398	11.94921253729826
H	-6.40227681827271	14.53989290201297	12.16238915160774
H	-5.37771947065903	15.80181035048069	12.85439480355937
C	-4.94559248676206	15.35534528822022	10.78389001529450
C	-7.62138144091395	15.30440186798501	8.03141679179184
H	-8.52309866533265	15.88219679167696	7.77725321649885
H	-7.95151443193868	14.29608190401193	8.33837402815160
H	-7.00946114986513	15.21678417483756	7.12378277766207
C	-6.36930299052836	17.37097098150856	8.65281960412674
H	-5.52072331196693	17.24512427808962	7.96716545400309
H	-6.06036749449983	18.04228916619839	9.46346669837048
H	-7.18185443519176	17.87139273553187	8.10415362571960
C	-4.08169530274199	14.11134740116356	11.05756990441653
H	-4.70878654706533	13.20386159714007	11.13122395541578
H	-3.55179877515485	14.21214893648097	12.01681051599901
H	-3.33036595709518	13.96805509290458	10.26760724688796
C	-4.00784258947751	16.57293570821210	10.67553129206005

H	-3.47933258249512	16.54469542466136	9.71470148796410
H	-3.25972846709448	16.54998015677630	11.48272773311090
H	-4.53894061242022	17.52995168802380	10.74914063240099
C	-0.23742831661826	13.56298876960876	7.59142678282479
C	-0.01836835412425	13.54793025266404	9.11641382321316
H	-1.00186656590796	13.42613576766161	9.60520800810860
H	0.57753333208341	12.65681152726320	9.37516851097237
C	0.63519756302144	14.81911402966127	9.64411191448485
H	0.71299705140376	14.77853471888425	10.74299733072885
H	1.66871385126119	14.90502874821911	9.27008535468552
C	-0.19085757610220	16.02730355103589	9.21917744805298
H	0.28393604060714	16.96542925311891	9.55030722663999
H	-1.17647803133977	15.98020302492017	9.71231633909895
C	-0.41560075686623	16.11104275224258	7.69617606910445
C	1.08772631332420	13.39868417555848	6.82266159719708
H	1.41878284628574	12.34929296869531	6.86305767562136
H	1.90138185242775	14.01028535101567	7.22905978200754
H	0.94082627422550	13.66795939634274	5.76965743436982
C	-1.12935394673318	12.37091698901674	7.23139334076853
H	-1.27963719238502	12.31232151429628	6.14742780479915
H	-2.10870148274665	12.43550438627731	7.73528641903851
H	-0.66386465141448	11.42825732889537	7.55733290073225
C	0.87493478196263	16.51345927913322	6.95543715441320
H	0.77540135946063	16.30264957647363	5.88257182671092
H	1.76789649084222	15.99817933368141	7.32794081136403
H	1.05079907106655	17.59387006821048	7.07005903482588
C	-1.45473611647019	17.20578008460725	7.43147001334885
H	-1.54787737706794	17.41746488108791	6.36080893942150
H	-1.15249945689786	18.14045479622598	7.92803730849011
H	-2.44288643070426	16.93068099416073	7.83676450859517
C	-4.29742832441557	9.90516865247352	6.13157880335597
C	-5.08852009557269	8.66765508213954	5.67197829934083
H	-4.36815127270237	7.84483080378970	5.54086657147899
H	-5.76405646982859	8.35173152220189	6.48853035179886
C	-5.90784145636483	8.89041557508909	4.40326273734818
H	-6.46727441675775	7.97435671101528	4.15784018561152
H	-5.24931081326903	9.08324485799686	3.54169302457397
C	-6.87918380613222	10.05043889213025	4.60454346952704
H	-7.60726358201906	9.78037381059681	5.39242167858471
H	-7.47322452966497	10.23371635891630	3.69582706807054
C	-6.19732814268257	11.37585904228037	4.98864304021125
C	-3.79881277645243	9.69601837634575	7.56512398611818
H	-3.22145397014603	8.76110439149183	7.61154444892172
H	-4.64010793388210	9.60096826783520	8.27322399034836
H	-3.15362552620745	10.51873202137253	7.89325579161328
C	-3.13075450938790	10.23321620049602	5.20129539522403

H	-3.34198966920256	10.01534192253425	4.14873788338866
H	-2.25984138685602	9.63005311238335	5.49422251826137
H	-2.84192967252819	11.28529985121696	5.28796852813679
C	-7.24705176588418	12.38250734319630	5.47050867399309
H	-6.80322098924011	13.36238633148980	5.68271765920230
H	-7.76350134322491	12.02825334258689	6.37887659458350
H	-8.00797609365713	12.51472567485052	4.68809848249661
C	-5.39196780791522	11.97566560533728	3.84328333982445
H	-4.68900446609367	11.27677591383904	3.38051419018361
H	-4.85140797985573	12.87578822353329	4.16253563620677
H	-6.09842576201916	12.29023474925397	3.06388617873955
C	-4.86075721343042	17.54076860414355	4.03464033783331
C	-5.00436094123772	18.60167617905888	2.92439643304827
H	-5.43224991842266	19.51350338337886	3.37258427275977
H	-3.99577109648539	18.86650636746549	2.56866160905083
C	-5.83512458344325	18.13181129652394	1.73709054269861
H	-6.88848232177808	17.98461634628987	2.02942420197634
H	-5.84442917464608	18.90524549232377	0.95192247503999
C	-5.23860320242245	16.84043459072671	1.19280070886765
H	-4.23189058704533	17.05665245124469	0.79512366789610
H	-5.83375359972779	16.44824877333568	0.35140773206019
C	-5.10981347518785	15.72765883067691	2.25339328789047
C	-3.80129414177087	18.04550456162379	5.01874113089272
H	-2.80649480478440	18.09254664528428	4.54741709689030
H	-3.74192122531112	17.41368233910671	5.91251714178964
H	-4.05088815626557	19.06178658199401	5.35979483174505
C	-6.18034040917591	17.37165533708399	4.81387109552330
H	-7.05883627449335	17.28682417049462	4.16415002163878
H	-6.34077414940013	18.24127548513791	5.46914267465024
H	-6.13482261169780	16.47638106152010	5.44902016089676
C	-6.49462499600498	15.15803163318471	2.62823775804941
H	-7.26996271772599	15.93152508275826	2.68877344345355
H	-6.44053630714365	14.64990359597774	3.59837222001558
H	-6.82390648830818	14.43046430621213	1.86966459236512
C	-4.26481152460486	14.60873349389820	1.63457894994804
H	-4.75392856717123	14.21300666853382	0.73154296469225
H	-4.11123003460097	13.77387910324298	2.32698452939761
H	-3.27941861509932	14.98337271180203	1.31873731511236
C	-1.00799956969925	12.03958306838060	0.62776369711001
C	-0.17909253300355	10.94665843547723	-0.07719153793211
H	-0.86674036583616	10.30370929879891	-0.65156313244421
H	0.48250786964346	11.42873256213966	-0.81656026682006
C	0.67279366155224	10.12015244260946	0.88011821233302
H	1.28339182178430	9.39409480089185	0.31810191398729
H	0.03239118006264	9.52209741024254	1.55027844830828
C	1.57064933874495	11.04868252755269	1.69109267755819

H	2.28003654962853	11.54531661103786	1.00636075762586
H	2.18218699169684	10.48196284928282	2.41286460725105
C	0.78849124633798	12.13461557879618	2.45655321083383
C	-1.55118616411861	12.99185140975570	-0.45115034871939
H	-0.72057333634291	13.48908912724211	-0.97682497421635
H	-2.19374339796144	13.76394550035667	-0.01210287362225
H	-2.14037961282379	12.43507448846251	-1.19712466154997
C	-2.20512540227423	11.41248395792594	1.37769194828783
H	-3.00131335566520	11.13779417451047	0.66760963695449
H	-2.60773917620910	12.14558742651307	2.08886304778675
H	-1.92821452101580	10.50363680254829	1.92957146968356
C	1.79787976094786	13.17530023881584	2.97177051332132
H	2.54561086066756	12.70487564648413	3.62846865956294
H	1.29529482809399	13.96690063396687	3.54364808594755
H	2.33840465129167	13.63644947163321	2.12860476736035
C	0.05417089479113	11.52755366044551	3.66923938387298
H	-0.49906502147745	10.61343254925648	3.41760034227580
H	-0.65372903677673	12.26721798140900	4.06607919735841
H	0.77056238939200	11.27250649643946	4.46467939447010
C	-0.23744387014786	18.63502535767064	0.93370388531640
C	0.76079022266148	19.51757305545477	0.16134880590179
H	1.30833635829421	18.87837365225431	-0.55319504256284
H	0.19503824542000	20.24967069820678	-0.43864192105148
C	1.76971083424938	20.21203147301269	1.07076918578311
H	1.26093430177861	20.93776744688411	1.72682757600921
H	2.48519069713378	20.79685828644334	0.46922703890064
C	2.51044109237023	19.16722862065131	1.89970099169770
H	3.22908624368044	19.64265377877564	2.58781433030068
H	3.10086930949191	18.53231459078865	1.21702677721855
C	1.57134953904098	18.26067906041518	2.71978813864709
C	-1.00143455267681	17.76546713984888	-0.08174330509524
H	-1.51554991655553	18.39754881874692	-0.82236062134536
H	-1.75770483400348	17.14619498797921	0.42235245306265
H	-0.30736490560010	17.10750399593397	-0.63112478832901
C	-1.26678083560685	19.49448506378214	1.69594838093980
H	-2.00951059291434	19.91419420393730	0.99927293905152
H	-0.81166003948625	20.33603585448876	2.23361270113207
H	-1.79198291167301	18.86291042424147	2.42480101994786
C	2.38901183887830	17.06857656462755	3.24614998736292
H	2.79719552595567	16.47684983331603	2.41031678635386
H	1.77072569447787	16.40695296838482	3.86735515330875
H	3.23666010058489	17.41980024470182	3.85505555122811
C	0.99281184252117	19.01870365472149	3.93371682751616
H	0.62183109205536	20.01870101449813	3.67528547556700
H	1.76526174597515	19.14545517140305	4.70848536493730
H	0.16301933068107	18.44233193959463	4.36556014960112

O	-7.17076259924670	11.35388853585118	9.32527650108814
C	-7.78471356660389	11.84616014899561	10.53399240886753
H	-7.46837548179073	12.89056590282791	10.67123993547426
H	-7.42200840621901	11.24810563291409	11.38982381095394
C	-9.29124636317079	11.66740030552626	10.31141043476546
H	-9.80979457924572	11.42711393251808	11.25025367275397
H	-9.73516517827740	12.59241558827058	9.91510486394685
C	-9.38111726244835	10.52155211993033	9.26803361130442
H	-9.92205027367500	9.64290362424835	9.64727081679940
H	-9.89900222640160	10.86047792339517	8.35888319234371
C	-7.91201936974290	10.18373855816221	8.96604779786729
H	-7.55802774077292	9.32664172502490	9.56822694894418
H	-7.71926289839787	9.96191378034914	7.90496736676969
H	-5.97992509185108	10.73871949962986	6.91224364994579

Cartesian coordinates for the model of **[Zn]**

Zn	-0.61547027032393	11.81010017251812	13.38343259391127
O	1.00027565385195	11.45357770837559	15.66663450472048
O	-1.78043573437046	10.94665593159345	12.21682231434195
N	0.59605150917795	10.63725875099870	14.59220256808025
C	-0.24679162078128	9.50407827235220	15.17804144372607
C	-0.62922429601751	8.53962792165459	14.03873616113049
H	-1.18032890238459	7.69653864456419	14.48693675184097
H	-1.32661924490060	9.05236964133935	13.35531076562962
C	0.57034291120926	8.04241021862236	13.23809298512991
H	0.22491898208903	7.41130126041151	12.40331777209756
H	1.22050201687545	7.40174381430098	13.85724972156829
C	1.35390320191581	9.23217676286304	12.69336813601713
H	0.71115944226783	9.77365065160177	11.97730813143655
H	2.24305759330502	8.90004543996645	12.13284988745120
C	1.82161487558292	10.21038883264193	13.78747152497725
C	2.39221067418243	11.46605667114903	13.11652246485356
H	2.88493816474502	12.12342825234284	13.84466118826787
H	1.62782243642226	12.05200052341894	12.58308340464951
H	3.15449151193314	11.17327147949277	12.37945825313696
C	2.93232291282022	9.62111811118977	14.67143633829539
H	3.01986756640514	10.21491219641052	15.59107137578997
H	3.88949983399729	9.67258582601860	14.13040624357557
H	2.76612622705441	8.57174142134479	14.93811422518169
C	-1.52673571388710	10.13958057918133	15.73240736068747
H	-2.07204670890525	10.68900819564515	14.94803922297817
H	-1.30859884325576	10.81369687686819	16.56961427299995
H	-2.20443092429481	9.34835233352350	16.08622907643436
C	0.45563441903132	8.77709914482148	16.33666915369538
H	-0.29963095812751	8.23120908585614	16.92220377902054
H	0.94861462680904	9.50167034557986	16.99692350230741

H	1.19633807059117	8.04210292075741	16.00203353123979
N	-2.48452186284786	11.93804452951562	11.46591251790975
C	-3.93866282031703	11.74293861887164	11.66296892343579
C	-4.44360306154271	10.32381597393157	11.32266717750607
H	-3.75939758529971	9.57861168945330	11.75194330376720
H	-4.51709670842128	10.14406354363012	10.24219156470619
H	-5.44636571348068	10.16311990800674	11.75080334788102
C	-4.23499797587758	12.01183444005305	13.14866207938296
H	-3.80099325551718	12.97664356084034	13.45236198721531
H	-3.82310928822468	11.21941141722573	13.78850983271166
H	-5.32267330446691	12.05252012285938	13.31975784656545
C	-4.67536920021342	12.81364755659790	10.83267575627235
H	-5.76091875772429	12.63284475154762	10.90828570782589
H	-4.48231021252368	13.79824642838598	11.29313882181495
C	-4.22562704804956	12.85827310336912	9.37571859852669
H	-4.74471480991487	13.67255865449193	8.84242116587235
H	-4.50760891325492	11.92672887477943	8.85656796206870
C	-2.71576641860458	13.06727295340024	9.31427918832222
H	-2.47521672744743	14.05342836143078	9.74826915708691
H	-2.35883479208890	13.07861094449194	8.27068575476811
C	-1.92682030855588	11.99623008410688	10.09355820026911
C	-0.46866453834344	12.47752286454537	10.21342774594551
H	-0.40595503007439	13.34621299711442	10.88658651255492
H	-0.08125988896494	12.76631406837512	9.22318361438804
H	0.18747243298055	11.68200259208871	10.59750575207147
C	-1.92513690332935	10.64506013175610	9.34611234383002
H	-2.91786136589033	10.35842075389436	8.97519707252228
H	-1.56802363947479	9.85523670141524	10.02192599221384
H	-1.25053354811479	10.69210008478402	8.47580495266760
Zn	1.34919212509008	13.31635458680819	15.63773218290217
O	-0.27341350454938	13.67379789836945	13.35788441608056
O	2.51941244521043	14.17943243140751	16.79898274278054
N	0.13512724814254	14.48938700805385	14.43120364131796
C	0.97684455664416	15.62225357881798	13.84295185429799
C	1.36340872968904	16.58629132012749	14.98121811296110
H	1.91335944461997	17.42926859012477	14.53138191942802
H	2.06291841761054	16.07312616800347	15.66215161765339
C	0.16685716808495	17.08390553557119	15.78610646839623
H	0.51567416008631	17.71406035602491	16.62019598512299
H	-0.48482257438671	17.72568190011999	15.16968797987311
C	-0.61595280643644	15.89448936486804	16.33272778465333
H	0.02860748639657	15.35220804276567	17.04655201384317
H	-1.50304692775628	16.22707298590381	16.89622754666535
C	-1.08781491042137	14.91713952814439	15.23966146206940
C	-1.65804195666916	13.66178964263452	15.91152958591291
H	-2.15200538188247	13.00475236303448	15.18395543747167

H	-0.89319549495222	13.07523705323172	16.44360610830581
H	-2.41896767973180	13.95517291318301	16.64976027939814
C	-2.20036479528171	15.50804596695539	14.35904934286683
H	-2.28983091412959	14.91586329226067	13.43857230646814
H	-3.15643962004692	15.45579403999566	14.90193663747616
H	-2.03462660287946	16.55785451799075	14.09380055391537
C	2.25481481017279	14.98625102377935	13.28463639783782
H	2.80227457227284	14.43675690615344	14.06741964328813
H	2.03395055287175	14.31203393725762	12.44820895961321
H	2.93163191187612	15.77718446202259	12.92849813597274
C	0.27151016629130	16.34987964355204	12.68649336421460
H	1.02553556915859	16.89507597041121	12.09871140663881
H	-0.22405763564319	15.62577352893598	12.02765569266752
H	-0.46754845769604	17.08558103840106	13.02319975636297
N	3.22653167485747	13.19022451154426	17.54966413450829
C	4.67996158014626	13.38721286302228	17.34916688402236
C	5.18343377778194	14.80770265748073	17.68586196664650
H	4.49729831782342	15.55114040617451	17.25659835420862
H	5.25873289659090	14.98955434391579	18.76586143202922
H	6.18511700813409	14.96912879016121	17.25549783956798
C	4.97335121501706	13.11605584452420	15.86331365836822
H	4.54162090280730	12.14918463496105	15.56302333216613
H	4.55708876952024	13.90569377205201	15.22286529807796
H	6.06068571430835	13.07854916068928	15.68933688630320
C	5.42009631389282	12.31904316959590	18.17969396988537
H	6.50519689348975	12.50138786008802	18.10138505077640
H	5.22755832507043	11.33337416702160	17.72130991763530
C	4.97358169646544	12.27604786684837	19.63770031242884
H	5.49499433027065	11.46330438371638	20.17107972908225
H	5.25537028082545	13.20875295306025	20.15487884949533
C	3.46415486963345	12.06494559098878	19.70265656466712
H	3.22415748507421	11.07778249932858	19.27065993846104
H	3.10941375579969	12.05471873299737	20.74700056580182
C	2.67202302335321	13.13363186792562	18.92337565518817
C	1.21413906100882	12.65041464087669	18.80792418079967
H	1.15044408740478	11.78105530710520	18.13574999121622
H	0.82983044241015	12.36209432091016	19.79950291166396
H	0.55587237132969	13.44478405913637	18.42507872039010
C	2.67054098755844	14.48603266929541	19.66860527009803
H	3.66396974099615	14.77485678521656	20.03590096748640
H	2.30993023180030	15.27423061286833	18.99274474462386
H	1.99880197744099	14.43930623521546	20.54114800266780

Cartesian coordinates for the model of protonated **[Zn]**

Zn	-0.44007419092661	11.96366268038450	13.35895875698412
O	1.45922303025650	11.78383817979910	15.54716521201652

O	-1.70495169660548	11.00658776737917	12.41885443081042
N	0.92303258324298	10.89293856699594	14.57677347924192
C	0.14266076837256	9.79486102382852	15.30143210789202
C	-0.37208367347529	8.78811528186095	14.25003618476168
H	-0.85701189210638	7.96350429247869	14.79643206709126
H	-1.15613432728052	9.27352818497326	13.64626968790425
C	0.70818018243949	8.25889492805400	13.31333916524527
H	0.24627271865327	7.62138437616035	12.54348046378470
H	1.42305692785431	7.61399404782009	13.85073322351911
C	1.42848007864429	9.42524459963946	12.64595190483751
H	0.70913132682645	9.95756878706297	12.00003165073793
H	2.23853742527012	9.07154058484916	11.98836037674636
C	2.04252702949207	10.41899497037898	13.65267152830361
C	2.56207102522394	11.64460179693659	12.89063250725817
H	3.08967595284130	12.34121949551120	13.55423951903795
H	1.75964731763256	12.20164766256988	12.38252486996724
H	3.27123109712347	11.31888294065071	12.11602869736491
C	3.22492490989402	9.78522616432801	14.40632356690392
H	3.41915452232341	10.31273098020720	15.34775069361930
H	4.13027383730648	9.85405794708706	13.78549873620043
H	3.07033344263502	8.72545266001239	14.63487494467111
C	-1.07894864185866	10.45248845225037	15.95382599019234
H	-1.70700848566798	10.95630128978456	15.20304077580903
H	-0.80103774686670	11.16814938177745	16.73687195831423
H	-1.70877574311530	9.67636991664962	16.41219226974075
C	0.96192492137227	9.09526420611737	16.39822862913941
H	0.27122802803143	8.57049072871001	17.07452096748615
H	1.52925180136032	9.82205999896807	16.99362642275360
H	1.66008777179154	8.34630060703011	16.00940840243286
N	-2.49849793017874	11.91665332822746	11.64522152460748
C	-3.93075716452096	11.64786828298073	11.93090215852970
C	-4.36424554730095	10.18263739029076	11.71118388205580
H	-3.62809538338813	9.50197155646426	12.16085876163084
H	-4.47085883565947	9.92029263137825	10.65127699338847
H	-5.33944494618289	10.00225592717566	12.19044018402684
C	-4.17215594125774	11.99911118958441	13.40832972842728
H	-3.81991789782427	13.02033696570125	13.61640549712689
H	-3.65536309669839	11.29536755079943	14.07612011350713
H	-5.24765005459026	11.95514158933272	13.64059644417893
C	-4.76792551467539	12.61616492883469	11.07043431131603
H	-5.83526909336994	12.38298283139194	11.21800476460416
H	-4.60927337727441	13.64049976854429	11.45095396239287
C	-4.39728287244873	12.58156809450573	9.59118410375775
H	-4.98791476402886	13.32793335512178	9.03501436141582
H	-4.65312268434795	11.60361794662044	9.15104031510416
C	-2.90724395134774	12.86620264266456	9.43116522594820

H	-2.70152609498850	13.89475262874456	9.77609903916969
H	-2.60575301853728	12.81803391746917	8.37178602018939
C	-2.01796514491596	11.89862877086251	10.23794757735740
C	-0.58454653921136	12.46252030076231	10.23515490176994
H	-0.52543675804204	13.37435258476660	10.84941974685777
H	-0.27746112183446	12.71374918261956	9.20793038218114
H	0.14307896556835	11.72523872613519	10.60926742430941
C	-1.98180653858330	10.49912303814692	9.58932066539358
H	-2.97426000606690	10.13890639224920	9.29163982087137
H	-1.54909536828825	9.77371048834078	10.29271079696301
H	-1.35670553899552	10.51904770717340	8.68234773630394
Zn	1.30088479525210	13.67880501620798	15.49387454146529
O	-0.04556904680756	13.82777734014065	13.23791831420611
O	2.45647531706367	14.14368737292180	16.95350180527487
N	0.17732496976225	14.75911056150646	14.27054168134051
C	1.02595062457068	15.89390999484745	13.69147037767944
C	1.23835769047108	16.93925893759136	14.80074798386373
H	1.80748692103733	17.77802472385403	14.36969359956341
H	1.88146217542261	16.50182572373751	15.58718748336288
C	-0.06113713245473	17.42338417542068	15.43569633560893
H	0.15999425398538	18.13346717308362	16.24800284760181
H	-0.66872543153797	17.97973179212961	14.70425293954021
C	-0.84037976807398	16.23382067790291	15.98542259148527
H	-0.25793610152000	15.77894553151729	16.80944798695737
H	-1.79757861820351	16.55361770762938	16.42672044327804
C	-1.14318620962162	15.14900067048569	14.93558152314331
C	-1.66432730002236	13.90340533767849	15.65869239151581
H	-2.02562132908273	13.13912690939306	14.95956430343910
H	-0.90593992111570	13.45147912481977	16.32354824204599
H	-2.51670022238725	14.17321643564759	16.29885386762134
C	-2.20449780610606	15.57987136838379	13.91541986304248
H	-2.21664580366426	14.88077080391280	13.06796328699659
H	-3.19316103703832	15.55350649205058	14.39739104898145
H	-2.05441728052252	16.59701627025786	13.53897868177137
C	2.37863980283893	15.27797774753003	13.31611429143320
H	2.86251679180022	14.79536406875038	14.18751563728081
H	2.27592034720525	14.54229696092141	12.51002665113787
H	3.06906887390036	16.06870026568894	12.98859570350258
C	0.40938648456112	16.50087314390803	12.42424491040251
H	1.18417051598356	17.06813344823675	11.88770030968945
H	0.04225449360781	15.70505048165439	11.76423602671391
H	-0.41224339220987	17.19348722646713	12.63512970084260
N	3.01635360851618	13.02890392680047	17.56648603580019
C	4.55488140854480	13.05206357613784	17.43099489268621
C	5.14802885682741	14.31875433729022	18.04841949018188
H	4.55274365988198	15.19690570712881	17.76637886010470

H	5.23170814179844	14.27696743678331	19.13952600894848
H	6.16376445387867	14.45277667275536	17.64853333679383
C	4.85114340049388	13.04203250910497	15.92822306265757
H	4.30856309638189	12.23909556499200	15.41112042863555
H	4.59857485655421	14.00376877382129	15.46335042045378
H	5.92654624763618	12.86597143553814	15.78145508026627
C	5.07619194526249	11.75813851055037	18.08089364562288
H	6.17653655028211	11.79160326563977	18.05246715259539
H	4.78040678604693	10.90053951232367	17.44934296290750
C	4.57028686735896	11.53494246825649	19.50484815333591
H	4.95043808901350	10.57472017084336	19.88628552618808
H	4.96775001484023	12.30723287212023	20.18242844997736
C	3.04324914734296	11.52966954496100	19.53386027720565
H	2.67029959133208	10.66623186457092	18.95356402403745
H	2.66687389472929	11.39319655109138	20.55972747288302
C	2.40613342203884	12.81176667050541	18.97058717935949
C	0.90450395702354	12.59903623407661	18.75272674793146
H	0.71232016771863	11.85379426288610	17.96890158678984
H	0.45756722361946	12.23504798001130	19.68919530754270
H	0.40296363137871	13.53897577021562	18.48440477248199
C	2.61316224493975	14.03424533507288	19.86461733305946
H	3.61704388435068	14.09781441057409	20.29749670929573
H	2.40383896947171	14.95581582613614	19.30582297406433
H	1.90088323992698	13.97170965630250	20.70030804456228
H	2.71550090402066	12.19422345208499	17.02141305868610

Cartesian coordinates for the model of **[Sn]**

Sn	16.94066437654504	7.19043772078279	6.01160883994630
O	18.08036405507203	7.52083629519088	4.28873982702952
O	15.26006831538180	6.88572518177548	4.81395054800223
N	18.79540280869556	8.74891295828365	4.38821242453413
N	14.07215854999491	6.68622793637121	5.58121247643685
N	17.76704326586502	4.85578468329279	5.51342624564724
C	20.24869956948309	8.45715980009535	4.45721993006538
C	20.99788487883277	9.80329943702544	4.52400176058833
H	20.77676370028709	10.27327498853506	5.49814061486913
H	22.08255492363354	9.60365916238327	4.50458473854535
C	20.59652242462634	10.76853560878352	3.41276852659142
H	20.89913664893730	10.37180969649383	2.42915590862788
H	21.12837095253681	11.72749867296295	3.53217609575993
C	19.08811145935262	10.99269919386267	3.45595035966943
H	18.76320090165533	11.66931318751573	2.64782770859618
H	18.83028094204352	11.48654705095636	4.40907463677808
C	18.27852966580977	9.68521746023102	3.35636928678041
C	20.77899434565064	7.58292618368384	3.29947543108216
H	20.10561710598180	6.72925063384775	3.14420183679749

H	21.78013231881122	7.19473353544678	3.54717383507769
H	20.86323508927764	8.13062508501064	2.35231913146343
C	20.50448364370593	7.70170483209630	5.77299629330407
H	20.08633940820680	6.68543129252844	5.74054012379007
H	20.05127966628163	8.24345123684322	6.61801708887452
H	21.58715899415090	7.61479508768078	5.95763050443002
C	18.30308762900273	9.11679972747398	1.92106191811446
H	17.67094320749170	9.73223153425601	1.26120965099503
H	17.90233927972428	8.09393652043046	1.92799264927926
H	19.30960199848224	9.09538025695015	1.48295013712277
C	16.81594774499480	9.98884390042561	3.71654039666219
H	16.42041983839248	10.78933246930309	3.07126712652456
H	16.74798555798616	10.32943202717615	4.76270782218818
H	16.18456493711016	9.09852990841192	3.59183105379710
C	13.23594488840239	7.91672120540534	5.51832652121500
C	11.93671975449495	7.64967373160128	6.30561188724392
H	12.19623612168458	7.55498334347801	7.37451015228196
H	11.28407776357013	8.53389150211255	6.21288214257268
C	11.21211937688326	6.38332053422928	5.86516587462682
H	10.84469037927874	6.48748606202074	4.83052961371055
H	10.31915698846001	6.21790931235746	6.49124933877454
C	12.16277165398263	5.19748529667629	5.97780455889190
H	11.67992969664743	4.26613921348789	5.63699902278466
H	12.42473336429982	5.05206492522638	7.04057956739770
C	13.46938537587594	5.38715574520113	5.18153156810056
C	12.93230603269901	8.40588740451549	4.08587586934522
H	12.56654779388290	9.44498097200447	4.11115406044224
H	12.16756127468094	7.80214434471783	3.58078491233918
H	13.84944823210824	8.37556460725414	3.48229226488182
C	13.99347305216376	9.03670740913461	6.25093954600134
H	13.33898640842011	9.91333481022399	6.38019494410402
H	14.87426833576463	9.36785227113385	5.68503693496385
H	14.31418591443303	8.69547491780074	7.24771176247673
C	13.22757957329541	5.24732413494844	3.66233385229127
H	13.07950914454256	4.18778983728411	3.39735672236119
H	14.10625710646031	5.62515699557866	3.12156097447978
H	12.34325529000029	5.79750651806717	3.31592902967908
C	14.44305389176574	4.27633012893293	5.60406353080859
H	13.94932973701034	3.29348590012405	5.53604451108710
H	14.76755191502344	4.43071436268035	6.64542048350924
H	15.32684778642930	4.25825463050971	4.95650407134900
C	17.81764748333163	3.87385719028717	6.41853206968241
H	17.56797781404365	4.14453832877721	7.44967740116034
C	18.15965136334476	2.56586873802412	6.07814062368650
H	18.19068071268273	1.78967004227367	6.84526060355088
C	18.45262754170886	2.28210130963214	4.74202313478688

C	18.39991818595641	3.31415389815457	3.80271793726565
C	18.05388611681110	4.59624451804278	4.23030263219447
H	18.71945124089635	1.26684055684096	4.43711613807779
H	18.62328430667370	3.13446493226217	2.74923470317733
H	18.00828017828636	5.45695710289076	3.55650611072672

Cartesian coordinates for the model of protonated **[Sn]**

Sn	17.18261280486319	7.23130983237685	6.04038281882868
O	18.02799242105732	7.54380164511379	4.20345445594390
O	15.28208434257185	6.79516076530853	4.98247281734811
N	18.81194293479646	8.74492004771389	4.36301610375162
N	14.06213696988368	6.63968876190427	5.59656491454654
N	17.81574684403589	4.91406550477760	5.60389436583449
C	20.25786168354739	8.39188392455397	4.30472291986852
C	21.05874234813027	9.70366585110616	4.43971071148385
H	20.92417047836355	10.08746991272202	5.46575608525197
H	22.12942971683666	9.46863823179688	4.32594390808240
C	20.62374759423126	10.78029827863510	3.45095605689185
H	20.84780149202879	10.47155489012480	2.41666805916377
H	21.19822667327983	11.70455387315679	3.62343711361977
C	19.13242110064551	11.05188596949383	3.61810023397615
H	18.78236734150886	11.80935451610701	2.89812720750519
H	18.95673096222875	11.46463255029416	4.62666154242257
C	18.26412905456301	9.78864780102610	3.45351012853964
C	20.66939722562683	7.61162327486452	3.03963318105572
H	19.95112650563242	6.80468908824594	2.84060642828139
H	21.66251520270481	7.15949825190470	3.18727188593817
H	20.72979727851484	8.24430987677696	2.14581715559685
C	20.57496935358912	7.51656342206199	5.52981755716030
H	20.15177443065932	6.50535455828218	5.43677086867743
H	20.19464146343528	7.98886893252484	6.45108337661989
H	21.66389302354577	7.40217915293232	5.64157783205392
C	18.17599507362268	9.35167679005891	1.97695448120580
H	17.53270390491062	10.05009771969197	1.41932311224041
H	17.73577368671826	8.34737315755065	1.90739437707314
H	19.15156138657211	9.33907481643072	1.47596899213256
C	16.84280805154425	10.11257803152781	3.93981137851788
H	16.44723017131886	10.98963104095103	3.40518577356175
H	16.85426486668556	10.35125033862238	5.01687386128183
H	16.16253875185954	9.26859475618158	3.75927071073277
C	13.20026939487121	7.92935972146836	5.47640416385381
C	11.84951888133105	7.65012100608446	6.15698283912112
H	12.00603834464755	7.58096667403983	7.24937084397805
H	11.21440525417054	8.53567181861642	5.99878791782585
C	11.15508219487702	6.38087808031663	5.67119613175145
H	10.87276530198958	6.47088363533734	4.61027437506887

H	10.21360105340506	6.23993977989539	6.22373858254973
C	12.06325264095298	5.17359636713958	5.88853102520224
H	11.58501245004007	4.24712482117441	5.53399397786493
H	12.22789742821830	5.03446908659051	6.97311503016966
C	13.42669400128543	5.28232793093402	5.18629362938164
C	13.06317517391740	8.34487537806394	4.01239353476312
H	12.74602643078346	9.39731403003220	3.97980146538712
H	12.31531797629871	7.76005384166905	3.46535852524295
H	14.02886783354934	8.26140872788792	3.49832541614279
C	13.95659853040882	9.01037664680610	6.25367650610151
H	13.31260280800308	9.89676425047274	6.34697112862612
H	14.87428869180827	9.31781328369772	5.73829269925821
H	14.20672899826079	8.68192159440776	7.27697392709370
C	13.32964243692080	5.17816939190328	3.66479563760516
H	13.17221794977317	4.12218560477164	3.40033663025387
H	14.26457400257987	5.51684544501074	3.20022693920013
H	12.49444332546195	5.74932217504188	3.24484138162901
C	14.37941886206753	4.20669235913955	5.71425034178127
H	13.89568998563959	3.22381982221822	5.61857144591310
H	14.61033658713639	4.35411118279597	6.78341870664395
H	15.31677363114207	4.18959542035602	5.14783500712972
C	17.89631811540276	3.98636972354126	6.56868127554837
H	17.69795874627141	4.31973203301111	7.59254641052961
C	18.21883953829116	2.65966627306473	6.29359851894749
H	18.28133453617539	1.93214226252187	7.10500652562056
C	18.46200085888130	2.29416430444110	4.96661142847139
C	18.38078081092124	3.26773817496547	3.96780314262352
C	18.05848349791813	4.57551513705133	4.32639398789125
H	14.19621370087936	6.53980283471996	6.61693574645167
H	18.00295886131471	5.39199273960763	3.60069850001133
H	18.57203907515024	3.02374941638762	2.92113494176462
H	18.71784694974057	1.26182345999553	4.71531129741196

Cartesian coordinates for the model of **[Cd]**

Cd	0.86667951649887	0.10930567027595	1.41568217569371
Cd	-1.01856659844141	0.23540002905756	-1.46007450480035
O	1.42952745496580	1.11649559793338	3.14014206725465
N	1.80766543552576	0.33413577538591	4.25697336335953
C	3.25950182450068	0.54253466428563	4.50705247830488
C	3.66954882793600	2.02511237879306	4.64298950358829
H	4.76092285095420	2.12934283677824	4.53074886554472
H	3.39698254679909	2.45624401669231	5.61471190370396
H	3.17696059264497	2.60898685554924	3.85354416662964
C	4.01400517961122	-0.04285697302317	3.30099490592125
H	3.67764100689874	-1.07029063605731	3.09833493903302
H	5.09706083190658	-0.06584824526636	3.50127282763239

H	3.84868348725348	0.56183975776501	2.39989916234424
C	3.65505524839982	-0.26055449169353	5.76130411670547
H	3.58120838506997	-1.33776647631840	5.52572435910433
H	4.71551700032310	-0.05916567262797	5.98891938491965
C	2.76823859554805	0.04174898878877	6.96510224957187
H	3.06135053180975	-0.58618795428739	7.82356433348586
H	2.90747188975640	1.08624397081640	7.29041152370730
C	1.30977969659035	-0.21262148699011	6.59876242831318
H	0.64041021033439	0.02414798744601	7.44295158895119
H	1.18185906759905	-1.28971315210031	6.38903104376274
C	0.84003874122226	0.58138569074210	5.36278301376853
C	0.64238285564454	2.07450447210392	5.70258996247790
H	0.53333447690731	2.65037485135944	4.77362887113178
H	1.47894141307429	2.49810434019776	6.27356801680487
H	-0.26856306714141	2.20755368166432	6.30787517807072
C	-0.51875792276586	0.01007198484905	4.92081201889708
H	-0.91384641616975	0.51208364121811	4.02878110512735
H	-1.25703235810711	0.11777205307029	5.73183946921856
H	-0.43235264936136	-1.06175260761198	4.69020460099700
O	-1.74230145815035	1.31511164462888	-3.07504108440045
N	-2.10905043788778	0.52170706748891	-4.19241997742557
C	-3.56134410724967	0.71131325019499	-4.44877970867573
C	-3.98429636233203	2.18816730984676	-4.60366205654107
H	-3.71201688360307	2.61060186709410	-5.57953159461881
H	-3.49968501323825	2.78484348772581	-3.81881464229412
H	-5.07683029049828	2.28425321994752	-4.49625556917801
C	-4.31343803774207	0.13031855571368	-3.23871169950118
H	-5.39657234595509	0.10569140352282	-3.43915866256930
H	-4.14617863965143	0.73946207316217	-2.34171156143279
H	-3.98118137508953	-0.89735338849308	-3.02818223804100
C	-3.94057391876722	-0.11139556991529	-5.69619623950350
H	-3.86528278386886	-1.18455580751899	-5.44471690609212
H	-4.99989548137240	0.08127762392433	-5.93620949638465
C	-3.04565787825323	0.17661739650172	-6.89794027812879
H	-3.32686145416497	-0.46905230088957	-7.74720559975855
H	-3.19043499443285	1.21398783267723	-7.24293630797416
C	-1.58703221882822	-0.05714249171267	-6.51725430110399
H	-0.91432351005753	0.17167149972414	-7.36104494827601
H	-1.44776846130206	-1.12866589460583	-6.28629413904320
C	-1.13884057075256	0.76620961978111	-5.29295539541665
C	0.22176108036009	0.22599929402670	-4.82192454312919
H	0.95698186454668	0.27324081588559	-5.64159123380468
H	0.12892353229126	-0.82685154913570	-4.51526626904158
H	0.62329488969360	0.79282390907608	-3.97109148924872
C	-0.95510894807581	2.25446786860689	-5.66065148813840
H	-0.04217991246899	2.38433497245059	-6.26402947023085

H	-0.85478353149367	2.84889570314059	-4.74241464619031
H	-1.79280024379463	2.65917308829300	-6.24338184835363
O	-1.21449531527943	-0.01897531170279	0.70766308001543
N	-2.23715804569149	0.23068249958103	1.64778620209001
C	-3.17454665357566	-0.91329686003428	1.70261273154903
C	-2.35655420368523	-2.14837612574958	2.11342637362495
H	-3.02102096778789	-3.01453936141886	2.26256763173783
H	-1.82676362555388	-1.94730893369482	3.05506914514890
H	-1.61415865435718	-2.40173318633513	1.34821827348558
C	-3.89311435386834	-1.22824089635969	0.37160083772669
H	-3.17037137466687	-1.35729926788681	-0.44574095888651
H	-4.61065464318569	-0.4523555792525	0.07662919814381
H	-4.45201137494889	-2.17322952262825	0.46437117985373
C	-4.19727435456204	-0.63821878876811	2.82562374829869
H	-4.95660719303608	-1.43780894270475	2.80882343826301
H	-3.67228578458333	-0.71551942899793	3.79285662783856
C	-4.84216210944478	0.73982953399479	2.73588911502913
H	-5.52029104637184	0.89715517331052	3.59146349425650
H	-5.46948838220701	0.81904882001086	1.83179805631760
C	-3.74993199913701	1.80382189311596	2.72708420368153
H	-3.19521407918593	1.75575732018617	3.67935350750336
H	-4.18170304968323	2.81595556553499	2.65831095039865
C	-2.74104958668062	1.62359361956741	1.57302685522390
C	-1.55386617088088	2.56684705285867	1.82945650636875
H	-0.88094249481034	2.61036397396792	0.96373391844174
H	-0.96486730033173	2.24667402259640	2.70041950906526
H	-1.91820180374001	3.59059008075889	2.00802606554532
C	-3.40213554979606	2.03976005181756	0.23866301192053
H	-2.75975845068373	1.86276638407899	-0.63703585485053
H	-3.60092651968148	3.12267792168157	0.25435230475912
H	-4.35870071997140	1.53566515043553	0.05273464281465
O	1.09039828136589	0.24943887083126	-0.76537090634036
N	2.00049310523252	0.85252675522519	-1.65972738355443
C	3.17081297567368	-0.02528509773890	-1.88165273558841
C	2.63412631065992	-1.37501027377214	-2.38251036671422
H	2.00076736499228	-1.22730137933557	-3.26702110342868
H	2.02655427502908	-1.85770567354726	-1.60931210306683
H	3.46753514607191	-2.04330814416572	-2.65183904979954
C	4.03462571776630	-0.27197083482220	-0.62374488875600
H	4.65877696277541	-1.16975621566665	-0.75583143998097
H	3.40189098531863	-0.43540235402511	0.25861878790862
H	4.70934364066173	0.56310762827305	-0.39687788367762
C	4.01858923537349	0.60112538291567	-3.00721588960260
H	3.44729854271261	0.52409448888801	-3.94755026320572
H	4.93398245824584	-0.00121251835752	-3.13539799057725
C	2.82774391948618	2.64376489671817	-0.01640669333471

H	3.89020102016569	2.37888538491383	0.05237268744281
H	2.31192537064463	2.16103190004540	0.82762841716647
H	2.75106928749938	3.72830709471888	0.15803085529069
C	4.35490302876034	2.06730957922110	-2.75592517198660
H	5.02684539509597	2.16794021030404	-1.88673609498069
H	4.90834593237560	2.48167555300558	-3.61523179233837
C	3.06896726270170	2.85599779461655	-2.53134505081205
H	2.46867746182800	2.83410059681802	-3.45680251710343
H	3.28856452095268	3.91516209541872	-2.31779580132806
C	2.19721847165725	2.29716693348585	-1.38541886351582
C	0.82504640109127	2.98517969407147	-1.46676225380042
H	0.95489825156678	4.07835195543627	-1.48245718033758
H	0.20589559641726	2.74957966753184	-0.59110380492567
H	0.27519775738363	2.68697659579370	-2.37179422820369
O	1.50149350667344	-2.22260321871493	1.74819632228644
C	2.47913946548503	-3.04080254132512	1.07989348317070
H	3.46587936298992	-2.55066998456009	1.13315060158015
H	2.19760931687857	-3.12815590652906	0.02206002950365
C	2.47515811848822	-4.36096415735286	1.84085154719294
H	1.64754466858534	-5.00301676881264	1.49507226867837
H	3.41512677869099	-4.91915623353113	1.71737302671325
C	2.22557329291978	-3.88622011896227	3.27797885634069
H	3.16117123798241	-3.50977908200079	3.72390384043975
H	1.83138005184680	-4.67357274519096	3.93722259600095
C	1.23182258419649	-2.74469360030697	3.07990791686133
H	0.18925116177493	-3.10015343659321	3.09628962486345
H	1.35898761775252	-1.91277196223048	3.78965102605597
O	-1.23082400986242	-2.08624800999961	-1.91995094743343
C	-0.63911423496261	-3.13190180086361	-1.14379570580017
H	0.04592759248472	-2.66765046868041	-0.42402557115114
H	-1.43439313725711	-3.64994319701420	-0.57862797135814
C	0.02800733423261	-4.07697898234128	-2.16485639861084
H	-0.20194400627451	-5.12748425829275	-1.93211333412874
H	1.12185544437600	-3.96913053633839	-2.16000702421872
C	-0.56492272762899	-3.63858171877880	-3.52982695530601
H	-0.93235540603674	-4.48521460341596	-4.12804844943374
H	0.19367921210790	-3.11096289819323	-4.12713343814137
C	-1.68842665312729	-2.67442029332665	-3.14544703664977
H	-1.87409355299565	-1.84673908539126	-3.84445075527239
H	-2.63614305498679	-3.21302587995702	-2.95162236312557

Cartesian coordinates for the model of protonated **[Cd]**

Cd	0.89038915847555	0.05451693250422	1.47099039189972
Cd	-0.97370734193586	0.04860037260300	-1.36284139891638
O	1.38067376963921	1.16976978275388	3.13483226413463
N	1.80418850862336	0.41491962028011	4.25865523986871

C	3.26254683664703	0.63868794802434	4.45912861023195
C	3.66661745589084	2.12519328458688	4.55259098855643
H	4.75496722965196	2.23060899456697	4.41766796899806
H	3.41215460659722	2.57613237341571	5.51966717887283
H	3.15858278660761	2.69346140345426	3.76146107264366
C	3.98609172527769	0.03049377819843	3.24435688429577
H	3.64642336740962	-1.00150012709382	3.06850431775646
H	5.07292942038383	0.00743013944902	3.41949017548131
H	3.80701453259405	0.62067609536047	2.33545807649298
C	3.69821128752919	-0.13711239195230	5.71745876591646
H	3.62740231311815	-1.21938736247320	5.50497124928961
H	4.76246687249540	0.07665648700294	5.91095616510124
C	2.84301213078533	0.18119009453209	6.93976895856140
H	3.16536458991494	-0.42910968000921	7.79960748475744
H	2.98366717336310	1.23153287601349	7.24403441701003
C	1.37659871396682	-0.09150243271083	6.62139157573527
H	0.73080787848657	0.15644681555561	7.48002293768292
H	1.25081515995596	-1.17341258378552	6.43646080762999
C	0.86467144942383	0.67685851402156	5.38602612288243
C	0.66680243935100	2.17414111368408	5.70474341495529
H	0.52947373583033	2.73861622198536	4.77242391134568
H	1.51436998994823	2.61080040581883	6.24823871959516
H	-0.22810302172922	2.31086079894350	6.33221250436705
C	-0.50173275183446	0.08787120816188	4.99222224338364
H	-0.92988834486658	0.58362688531652	4.11199181985221
H	-1.21288247538951	0.19296416781382	5.82678339594895
H	-0.41329598717490	-0.98462551847809	4.76404929061518
O	-1.73286203126640	1.11687547634616	-3.05243403563935
N	-2.06377409318395	0.60365879013533	-4.27688663214866
C	-3.59695375804637	0.67577691805279	-4.49628488848065
C	-4.09953807670437	2.10629996198394	-4.30864370095502
H	-3.89235583430969	2.75389856231775	-5.16800819059065
H	-3.65453541722417	2.55163472542373	-3.40968526491886
H	-5.19080067677128	2.07459510032578	-4.17556580993084
C	-4.20429439409504	-0.22754990862024	-3.41700709258471
H	-5.27709227981742	-0.35843510936324	-3.61949208462635
H	-4.09387067710320	0.20949527544345	-2.41697198847192
H	-3.74558567430997	-1.23055513136867	-3.41540738036481
C	-3.89173938930932	0.11439532531038	-5.89646117023420
H	-3.70241448693665	-0.97550398386541	-5.89516797081883
H	-4.97095698411312	0.23270283168612	-6.08127517572176
C	-3.06683123337273	0.76368274635844	-7.00557764538467
H	-3.31764251543837	0.30172671684672	-7.97291484623261
H	-3.32084187234286	1.83072937325333	-7.10587924815801
C	-1.57683929803368	0.58577177366855	-6.72361076843271
H	-0.96398339913567	1.05447239194259	-7.50956200364419

H	-1.33269225009772	-0.49233791555584	-6.75351356247902
C	-1.11633421872602	1.16249930938742	-5.37393175427243
C	0.27353771690373	0.62570328151101	-5.01876245204047
H	0.98594238411189	0.93242419228815	-5.79836716730650
H	0.27803061043167	-0.47664191184062	-4.99795946188986
H	0.64086653418690	0.99140051267313	-4.04968769251919
C	-1.10869752882888	2.68976247930455	-5.34573787346537
H	-0.23321035720564	3.04110236413676	-5.91082378322532
H	-1.02119092533646	3.05329515152308	-4.31446216989283
H	-1.99734661467452	3.13580270424134	-5.80706305432207
O	-1.21559455330266	-0.17459313305829	0.75745689092485
N	-2.25671386825688	0.12601431532578	1.65572054934705
C	-3.20243523396104	-1.00934371561501	1.75060025120363
C	-2.40194516889729	-2.22949465866784	2.23328927237466
H	-3.07650595424657	-3.08139775366268	2.41221313730340
H	-1.88876073490989	-1.98770392329575	3.17442081409769
H	-1.64479739701212	-2.52726151854530	1.49931867121808
C	-3.90346841344636	-1.36694678044742	0.41986534152977
H	-3.17634338234704	-1.47321140285760	-0.39980367721960
H	-4.65078237944301	-0.62110683936300	0.11965611317309
H	-4.42472079380451	-2.33206273205530	0.51538471903947
C	-4.24438608531409	-0.67621875554432	2.83925274957898
H	-5.00954998194892	-1.46979911728631	2.84324703567149
H	-3.73782959510561	-0.71718713204035	3.81780668429036
C	-4.87517886948893	0.70152421435501	2.67866419503345
H	-5.56370627549132	0.90042781409687	3.51579213300966
H	-5.49134524828748	0.74800315783802	1.76415022618878
C	-3.77473572215113	1.75580636744010	2.64433242306865
H	-3.23774004074739	1.74651786331077	3.60720937175829
H	-4.19569082525604	2.76718270757362	2.52382365711121
C	-2.74357891434198	1.52057079357925	1.52092384547108
C	-1.55466935480616	2.46451975583691	1.76066535885934
H	-0.86401940236297	2.47092631684751	0.90728422890579
H	-0.98273292197043	2.17673956177405	2.65364000682972
H	-1.91390174751876	3.49668396461168	1.89158370526750
C	-3.37623365871724	1.88049010023781	0.15611942537045
H	-2.71309364586759	1.66432155968723	-0.69736054733365
H	-3.56246151289421	2.96438731980941	0.11514028174102
H	-4.33284606180098	1.37547621465327	-0.02865851685197
O	1.09324398477265	0.20066290339610	-0.74158729053769
N	1.97856750583986	0.78565998923721	-1.68396043800261
C	3.12176636741391	-0.12783866801283	-1.93258165803661
C	2.53266445407523	-1.45122688223876	-2.44616955376357
H	1.87506546174227	-1.26075225787546	-3.30720844658516
H	1.94503005680873	-1.94662771762978	-1.66394071937012
H	3.33770096605453	-2.12997143078914	-2.76755084566687

C	3.98986018185597	-0.42464378117749	-0.69103898606064
H	4.57974075768324	-1.34076467019041	-0.84850609350267
H	3.36354088749420	-0.58140251884656	0.19644950226985
H	4.69793062547001	0.38034558706449	-0.45981568329678
C	3.97950330246441	0.48683377190771	-3.05699060249937
H	3.40351070099415	0.44169330736527	-3.99754365855980
H	4.87198317352681	-0.14437283146788	-3.20054528571066
C	2.88426960101016	2.52963517687830	-0.02561423477318
H	3.92213316763032	2.18412549224369	0.04697650648280
H	2.32980163554626	2.10187596541830	0.82305996247606
H	2.89295217150967	3.61844047473512	0.13534673614192
C	4.36800695704227	1.93639843564215	-2.78628009475900
H	5.04969976117281	2.00035182675368	-1.92224696133943
H	4.92866765446543	2.34473518987651	-3.64279531090100
C	3.11223030580002	2.76506824948313	-2.53852438627298
H	2.50590186892222	2.77984843520474	-3.46110386983043
H	3.36891919630806	3.81271494313132	-2.31334390685371
C	2.23202477518229	2.22232907303536	-1.39107481849809
C	0.88254343517585	2.95408588308004	-1.44959074094227
H	1.04052123354770	4.04281938010498	-1.42474031729194
H	0.24944133442628	2.70137985365594	-0.58955028931650
H	0.33538085175927	2.70599059165902	-2.37094912494272
O	1.43071685947749	-2.25156646831740	1.81152058496836
C	2.36853039097137	-3.11131981805341	1.13127674180728
H	3.35320960856115	-2.61768762488813	1.09083662872760
H	2.01752965708341	-3.26307992206462	0.10080216518129
C	2.41966359616470	-4.38241938893141	1.96945300859285
H	1.57822082611207	-5.04863696806733	1.71481367472245
H	3.35461102607474	-4.94287071081088	1.82296269175238
C	2.25559193814142	-3.82296375135744	3.38834340157897
H	3.21147929075189	-3.40932246580378	3.74896158185127
H	1.91580706607030	-4.57284999472629	4.11716054284007
C	1.23494973642067	-2.70866314635341	3.18720945802813
H	0.20037078724496	-3.07673524823042	3.27151487878817
H	1.38297804477317	-1.83760137867765	3.84467429005803
O	-1.09221410532083	-2.21374931217672	-2.11210515941576
C	-0.83392773759923	-3.19919784222121	-1.07092903695533
H	-0.22280828237524	-2.72469710900176	-0.29194355815450
H	-1.79890840704147	-3.49201406192261	-0.62805050707479
C	-0.15824778272075	-4.35985207313393	-1.78809337894508
H	-0.30856800926170	-5.31655678119475	-1.26771301078102
H	0.92563661408843	-4.18331626667609	-1.87536417265380
C	-0.81898512131508	-4.31305827199141	-3.17133362026006
H	-1.81810552935546	-4.77643454715596	-3.13857421339901
H	-0.23530422821167	-4.81926982336930	-3.95350484493339
C	-0.93357165657052	-2.80986814221861	-3.41579305369701

H	-0.02055790942836	-2.40432023589422	-3.88218322357979
H	-1.80656928069653	-2.54463011365834	-4.03642421988901
H	-1.87110849232189	-0.40927509037626	-4.29829607237169

Cartesian coordinates for the model of **[Pb]**

Pb	3.76036842727993	12.20036226708285	3.67119483256038
O	6.02804394148737	11.66194671020138	3.84667968034357
O	3.57398657137505	12.36647150728387	1.47517827986815
N	6.79068683511409	12.72700209828011	3.30951974632370
N	2.73840851118315	13.51531609034243	1.34851302134373
C	7.34698188503967	13.58325697305072	4.38759261755950
C	6.18047192686079	14.09828596463500	5.24783232105163
H	5.40672366599691	14.56604077850163	4.61990770412761
H	6.54476318103458	14.86311100178913	5.95113213348027
H	5.72930132318038	13.28881590284913	5.83907905956680
C	8.81411494798225	11.32372049503190	2.58393073308906
H	9.16547591812537	10.78094136852159	1.69245390558038
H	8.50713108010395	10.56963363041107	3.32163369612297
H	9.67878981360189	11.84633325121693	3.01156701455985
C	6.77140843983594	11.56996638928317	1.15510070974849
H	5.81796575178379	12.09447024176553	1.00740441453599
H	6.53118338475764	10.53760616616462	1.45155062046852
H	7.30687300680556	11.50990951467859	0.19450389000327
C	3.51619406887027	14.62766459312707	0.73099048214031
C	2.57423423685403	15.82421736271286	0.48835556872422
H	2.28341816863112	16.24165628293789	1.46831720593423
H	3.14452472405949	16.61029928889007	-0.03397461715519
C	1.30929342960852	15.45379674320280	-0.27413447573682
H	0.66093626018467	16.33830180395663	-0.39192070232668
H	1.55225253020965	15.11380387986703	-1.29474256931846
C	0.57919186636811	14.36382337856385	0.49937621572315
H	-0.34491680821424	14.05445850617880	-0.01681111025810
H	0.27438765790605	14.77404223355496	1.47830320248130
C	4.23802237695790	14.22967582221056	-0.57335073172119
H	3.54922039452497	14.10804072197609	-1.41958650916098
H	4.77441232785898	13.28290939408383	-0.43309390646446
H	4.96811348684850	15.00688018221164	-0.85099509958163
C	1.43694637414335	13.10698992581554	0.74738640781297
C	4.57028080563133	15.07347565584179	1.75592996281079
H	5.30098586568041	14.28379418503971	1.97460079273119
H	4.07744397951422	15.36396858320450	2.69860686559124
H	5.12120545872327	15.95116078246378	1.38128146651675
C	0.69884348932951	12.25036792125625	1.78770654895160
H	-0.32450012317239	12.02819040877350	1.44578168741525
H	0.62833632485377	12.79139290315868	2.74524935986358
H	1.20635935352949	11.29331076907216	1.95290483413021

C	1.59741534773676	12.27709013019860	-0.54257357794995
H	1.88352669353408	12.89045712497271	-1.40750761673119
H	0.64916604927805	11.77504362324178	-0.79333879050048
H	2.37246193824676	11.51377832199298	-0.38916882214376
C	7.99303447759593	14.80786473281280	3.70277197269844
H	7.18227013591778	15.43412021873000	3.29495717903510
H	8.50414658093609	15.40973871071032	4.47257904484627
C	8.94944069272769	14.45190140535423	2.56870873071307
H	9.30564393657319	15.37240143821129	2.07669847591759
H	9.85055260632031	13.95573222061762	2.96733635926527
C	8.25024568218146	13.55224102983094	1.55226439726180
H	8.94737605503670	13.24961663546497	0.75312388134581
H	7.43170580007110	14.10967667345392	1.06837160773222
C	7.65849259838609	12.27899425697267	2.19315289554729
C	8.32321011474173	12.86458388101735	5.33749750725662
H	7.85367344168650	11.94831122311905	5.72181496012124
H	8.54954638204620	13.50765348977054	6.20262688460557
H	9.27753549911032	12.59956486813006	4.86692198920287
Pb	6.26664973413730	9.29056860754287	4.09185393301506
O	4.00064485279768	9.83066437365406	3.90858313783683
O	6.43797761194843	9.12333488151949	6.28864715424743
N	3.23071894368321	8.75694401639688	4.41654048861503
N	7.27759184716983	7.97726984950670	6.41228191547740
C	2.69063656966129	7.91912172349070	3.31628969237065
C	3.87012847941827	7.41436332608606	2.46770277683027
H	4.63266250053476	6.93640345938444	3.10183155642685
H	3.51594061122558	6.66019609639655	1.74791462235092
H	4.33203949875688	8.23102582049606	1.89486855341439
C	1.19807044013978	10.14899826352284	5.13903076998636
H	0.82691612477123	10.66589796457942	6.03784454393907
H	1.51983197599925	10.92493871009356	4.43098548877681
H	0.34390705404735	9.63763060719918	4.67811550507986
C	3.22058823119217	9.87801041301674	6.58942081694280
H	4.17189916810169	9.35108327180772	6.74115012107491
H	3.46556128705980	10.91464911048221	6.31227601303800
H	2.67273019949757	9.92297330817674	7.54385606411761
C	6.50255731654359	6.85894746161235	7.02251100294506
C	7.44848077068227	5.66430034987856	7.25892693942754
H	7.74137355802382	5.25338474082374	6.27682208227409
H	6.88062123392131	4.87325568773673	7.77644548922365
C	8.71153888081610	6.03472686799267	8.02467075926355
H	9.36314228679499	5.15191302670535	8.13712207540391
H	8.46648845570413	6.36685622046271	9.04736769536802
C	9.43850399714361	7.13278505557148	7.25957784549909
H	10.36051228731523	7.44229705618913	7.77943740170534
H	9.74667186146715	6.73060293710561	6.27835955159011

C	5.77869154328316	7.24770875598362	8.32849934911467
H	6.46617070408152	7.36155433009948	9.17688705568992
H	5.24444351783249	8.19661208823648	8.19430692328491
H	5.04697611645738	6.46954957447415	8.59904941792860
C	8.57593366002743	8.38763093391945	7.01873713841226
C	5.45064753714539	6.41552064951656	5.99400978135211
H	4.71673978275421	7.20327144415843	5.77895205751424
H	5.94506088668719	6.13222299683018	5.04994538660333
H	4.90289226768903	5.53351995919035	6.36313745004788
C	9.31189252892510	9.25540500216359	5.98601730149742
H	10.33406589550348	9.47852246754386	6.33073946305500
H	9.38544065738987	8.72284847798183	5.02394197495949
H	8.80085217419422	10.21206203552485	5.82867724805200
C	8.40924392286190	9.20858640646291	8.31353974322962
H	8.12240301560154	8.58917476275053	9.17388086909291
H	9.35527451971333	9.71188535365793	8.57012069727223
H	7.63231910300581	9.97049469367755	8.16241618179422
C	2.03159789634122	6.68473431678314	3.97060164021548
H	2.83530911669336	6.05044936245865	4.38037391196611
H	1.53170132555522	6.09656713340587	3.18306700470851
C	1.05902187808865	7.02309762971214	5.09625862716157
H	0.69365771542402	6.09492101197354	5.56663900702046
H	0.16510879355108	7.52873244847684	4.69342557844675
C	1.74598888911822	7.90280763726965	6.13827704606358
H	1.03925354070577	8.19180048613249	6.93396746515116
H	2.55737077380103	7.33472676555675	6.62178841570801
C	2.34753056656769	9.18640324977124	5.52817613762372
C	1.73239438459466	8.65571912456367	2.36174645682199
H	2.21143133679553	9.57575751587382	1.99851398202667
H	1.51740312713116	8.02645140147942	1.48366195748567
H	0.77175215134819	8.91693711314714	2.82141839679729

Cartesian coordinates for the model of protonated **[Pb]**

Pb	4.08523082903985	12.11865113853505	4.13110820694623
O	6.27657650801277	11.62119737478459	3.92805920533207
O	3.37441579567113	12.52557374636249	1.90240955606139
N	6.89866309035084	12.64732151387301	3.15917319518086
N	2.60283022158354	13.62707840594545	1.62046055132516
C	7.71744469303982	13.53638605330411	4.03071518705260
C	6.79892868001971	14.11063762389530	5.12118049448452
H	5.89126319195772	14.55559320851093	4.68042608552566
H	7.32180289736644	14.90941613566890	5.66822561563490
H	6.51016886813262	13.34289777532871	5.85379387423313
C	8.69425665544469	11.12870533132395	2.11612541021341
H	8.84907180164490	10.54195099378891	1.19778877122495
H	8.51646504497065	10.41285356280616	2.93056396712421

H	9.63646150915551	11.64210357692670	2.33774276496620
C	6.40393348919279	11.33351250029762	1.15253328843232
H	5.46335826472196	11.89828255636969	1.12960587107001
H	6.19051810649314	10.35400209003347	1.60367101851945
H	6.73351782116057	11.14918562573724	0.11855314699815
C	3.42814297787785	14.73110529503962	0.90267210474905
C	2.48312652698324	15.90979787210875	0.61196853700933
H	2.21529494242596	16.39896663582508	1.56655539128086
H	3.05637089907081	16.65642476080001	0.04041264866880
C	1.20426728014020	15.51362048625444	-0.12008617239733
H	0.57455007549976	16.40318277294327	-0.27471645391741
H	1.43247106152979	15.12494218752285	-1.12518679738736
C	0.43986706183368	14.47992636636761	0.70163187889549
H	-0.48764106838871	14.17219365535576	0.19380207783017
H	0.12466983271192	14.94216622143059	1.65526395212672
C	4.08396074380896	14.17928981987352	-0.36147129497649
H	3.38722668639039	14.08316678283399	-1.20174111036972
H	4.54350934698889	13.20333168763263	-0.16954550039431
H	4.87687836637959	14.87579399302033	-0.67122117726132
C	1.23900718423668	13.20212582190872	1.00936344844653
C	4.49828540474675	15.15583486346182	1.91047217693044
H	5.22805484219122	14.35914069955700	2.10840517328492
H	4.04525025932326	15.47144508547693	2.86708887941837
H	5.03918713541571	16.02675565587528	1.51252333251243
C	0.53391539072224	12.40224235176943	2.10667216968444
H	-0.50280716713344	12.20473215898146	1.79763949542749
H	0.48814220051919	12.96619798983948	3.05456048762349
H	1.02865181545291	11.44240716721501	2.28599983648332
C	1.45712199748654	12.31967874920528	-0.21820050204824
H	1.73573694751707	12.88675701642929	-1.11369922237582
H	0.51508095238772	11.79702443594513	-0.43974181410963
H	2.22947203139955	11.56681373594171	-0.01579951996541
C	8.19940571598045	14.70543468627979	3.14410166976591
H	7.32929458296550	15.33904352274498	2.90253462780951
H	8.88613346950540	15.33096157656098	3.73729652600492
C	8.86386453619806	14.25739918771574	1.84460631068869
H	9.10260511891769	15.13624260520652	1.22393625061743
H	9.83032356962683	13.77195045285269	2.05700164004356
C	7.95011355764906	13.30614661080703	1.07470622427974
H	8.44929550982639	12.93580617432370	0.16450764004678
H	7.05016183929027	13.84998056015688	0.74358711631316
C	7.50119016721741	12.09238132446117	1.91588671001223
C	8.89786933412792	12.83912258573441	4.73375628107255
H	8.57497077330414	11.88633935353436	5.17124312041794
H	9.26857154319599	13.46888914366593	5.55702497929519
H	9.74629636275351	12.64586991514897	4.06757460524126

Pb	6.50971951126147	9.19284125210331	4.39141191191631
O	4.23001281516831	9.84682212190812	4.05429915143577
O	6.21113327252364	9.04999892372184	6.55431035044690
N	3.37991252549585	8.75645510204122	4.35412928481964
N	7.20218323341246	8.03982693878582	6.74638195160069
C	2.96306654943136	8.06028922770045	3.10891739242248
C	4.22809710763627	7.63851693731184	2.34280206161730
H	4.91771541028519	7.08640334341824	3.00054256627196
H	3.95645791541771	6.96411755426706	1.51663548792995
H	4.74838689921715	8.50246580593608	1.90522440243661
C	1.28788779355605	10.09008983582685	5.00678284453882
H	0.78279090845578	10.46250348688729	5.91105611772785
H	1.70150092438403	10.96964101345369	4.48933442145906
H	0.51846480137639	9.67014264184016	4.34835685475393
C	3.11867912870194	9.62620428391773	6.63719699645470
H	4.05510733767288	9.09033952068936	6.83757990484773
H	3.37773655059030	10.69341373237939	6.52702434623511
H	2.46069163867429	9.56111981160605	7.51712616660951
C	6.55030697768390	6.73966040246838	7.08827989956813
C	7.66495019987208	5.69524714314202	7.29970839455801
H	8.14339460011641	5.49720170758195	6.32458008096861
H	7.19810363313902	4.74990523653841	7.62123886217089
C	8.73560066055247	6.14350425175374	8.28860698038365
H	9.52435121250716	5.37745380143663	8.36364652435306
H	8.31163135003575	6.24232400421821	9.30147818268564
C	9.33389732985399	7.46736994015767	7.82501903870758
H	10.09216400822366	7.83212538260320	8.53712704919950
H	9.85222461073667	7.30673083454356	6.86335125702885
C	5.61359143485580	6.81238051615192	8.31097005001323
H	6.15599654406425	6.88197795068050	9.26136769298401
H	4.94661930766106	7.68125780104666	8.23389640217434
H	4.99131014725791	5.90485971083861	8.35724028372671
C	8.27965990994491	8.57352968526438	7.63021610701828
C	5.71256580787179	6.29984913147534	5.87600394492736
H	4.87123792858515	6.98112174221345	5.68417475053707
H	6.34413718160158	6.23890359801360	4.97232671973529
H	5.29867419642168	5.29426370283233	6.04702423182140
C	8.93808122607809	9.74628453046146	6.88993793942960
H	9.84179331046844	10.07599090269505	7.42458656904768
H	9.24664472501568	9.44410779435673	5.87371346277838
H	8.25471416320416	10.60340752661316	6.82144404611105
C	7.75534631986597	9.10414089983803	8.98023077006132
H	7.51751753795632	8.30438564835720	9.69240734214741
H	8.51559352699251	9.74563100464604	9.45263372978616
H	6.84940788448468	9.70332768505036	8.81394268966068
C	2.23214747480957	6.76975469478028	3.54056718877797

H	2.98397106555144	6.08032291158714	3.95981553842992
H	1.82288238777715	6.28364917387283	2.63999329686442
C	1.14296601596372	6.99383759964443	4.58409674663170
H	0.72355040129444	6.02510344665595	4.89999774571482
H	0.29982098480736	7.55771525975006	4.15035665640356
C	1.71667435803611	7.73264288303574	5.79016742565996
H	0.93197698625101	7.93297641842245	6.53772761651943
H	2.47400944210206	7.10135770443458	6.28334143288215
C	2.38211471967232	9.07189957253143	5.40767162374471
C	2.10941797417670	8.91531128328564	2.15033968845713
H	2.58435115131432	9.89432908219265	1.98732517135167
H	2.03594054382758	8.41693068948933	1.17134760856837
H	1.08505277620783	9.07231819304143	2.50975572482703
H	2.32226638385955	14.08945583719872	2.50297938295469

References

1 L. Balloch, A. M. Drummond, P. García-Álvarez, D. V. Graham, A. R. Kennedy, J. Klett, R. E. Mulvey, C. T. O'Hara, P. J. A. Rodger and I. D. Rushworth, *Inorg. Chem.*, 2009, **48**, 6934–6944.

2 A. Kornowicz, T. Pietrzak, K. Korona, M. Terlecki, I. Justyniak, A. Kubas and J. Lewiński, *J. Am. Chem. Soc.*, 2024, **146**, 18938–18947.

3 P. Jochmann and D. W. Stephan, *Chem. Commun.*, 2014, **50**, 8395–8397.

4 W. S. Khan, C. Cao, T. Mahmood, M. Ahmad, F. K. Butt, Z. Ali, Z. Usman, F. Wang, and Qurrat Ul Ain, *Mater. Lett.*, 2011, **65**, 1896–1899.

5 K. Ahmad, M. B. Kakakhel, S. Hayat, M. Wazir-ud-Din, M. M. Mahmood, S. Ur Rehman, M. T. Siddique and S. M. Mirza, *Radiat. Environ. Biophys.*, 2021, **60**, 365–375.

6 A. Banerjee, M. K. Ravikumar, A. Jalajakshi, P. S. Kumar, S. A. Gaffoor and A. K. Shukla, *J Chem. Sci.*, 2012, **124**, 747–762.

7 R. Sakamoto, N. Shirai, A. Inoishi and S. Okada, *ChemElectroChem*, 2021, **8**, 4441–4444.

8 J. Burbank, *J. Electrochem. Soc.*, 1957, **104**, 693.

9 P. Erdmann and L. Greb, *Angew. Chem. Int. Ed.*, 2022, **61**, e202114550.

10 P. Sanjay, K. Deepa, J. Madhavan and S. Senthil, *IOP Conf. Ser.: Mater. Sci. Eng.*, 2018, **360**, 012010.

11 T. S. Bhat, A. V. Shinde, R. S. Devan, A. M. Teli, Y. R. Ma, J. H. Kim and P. S. Patil, *Appl. Phys. A*, 2018, **124**, 34.

12 Bruker, SAINT, V8.41, Bruker AXS Inc., Madison, Wisconsin, USA.

13 L. Krause, R. Herbst-Irmer, G. M. Sheldrick and D. Stalke, *J. Appl. Crystallogr.*, 2015, **48**, 3–10.

14 G. M. Sheldrick, *Acta Crystallogr. A Found. Adv.*, 2015, **71**, 3–8.

15 P. Müller, *Crystallogr. Rev.*, 2009, **15**, 57–83.

16 C. R. Groom, I. J. Bruno, M. P. Lightfoot and S. C. Ward, *Acta Crystallogr. B Struct. Sci. Cryst. Eng. Mater.*, 2016, **72**, 171–179.

17 D. Kratzert, FinalCif, V152.

18 G. M. Sheldrick, CELL NOW, University of Göttingen: Germany, 2008.

19 D. J. Liptrot, P. M. S. Hill and M. F. Mahon, *Angew. Chem. Int. Ed.*, 2014, **53**, 6224–6227.

20 A. L. Spek, *Acta Crystallogr C Struct Chem*, 2015, **71**, 9–18.

21 Neese, F. Software update: the ORCA program system, version 5.0 WIRES Comput. Molec. Sci. 2022, 12, e1606.

22 F. Weigend and R. Ahlrichs, *Phys. Chem. Chem. Phys.*, 2005, **7**, 3297.

23 F. Weigend, *Phys. Chem. Chem. Phys.*, 2006, **8**, 1057.

24 S. Grimme, J. Antony, S. Ehrlich and H. Krieg, *J. Chem. Phys.*, 2010, **132**, 154104.

25 V. Gold, A. McNaught, and The International Union of Pure and Applied Chemistry (IUPAC), Eds., *The IUPAC Compendium of Chemical Terminology: The Gold Book*, International Union of Pure and Applied Chemistry (IUPAC), Research Triangle Park, NC, 5th edn., 2025.

26 A. Moser, K. Range and D. M. York, *J. Phys. Chem. B*, 2010, **114**, 13911–13921.
